# Convergent Total Syntheses of (−)‐Rubriflordilactone B and (−)‐*pseudo*‐Rubriflordilactone B

**DOI:** 10.1002/anie.201908917

**Published:** 2019-10-31

**Authors:** Mujahid Mohammad, Venkaiah Chintalapudi, Jeffrey M. Carney, Steven J. Mansfield, Pollyanna Sanderson, Kirsten E. Christensen, Edward A. Anderson

**Affiliations:** ^1^ Chemistry Research Laboratory University of Oxford 12 Mansfield Road Oxford OX1 3TA UK; ^2^ Department of Molecular Biology and Chemistry Christopher Newport University 1 Avenue of the Arts Newport News VA 23606 USA

**Keywords:** cyclotrimerization, natural products, schinortriterpenoids, structure elucidation, total synthesis

## Abstract

A highly convergent strategy for the synthesis of the natural product (−)‐rubriflordilactone B, and the proposed structure of (−)‐*pseudo*‐rubriflordilactone B, is described. Late stage coupling of diynes containing the respective natural product FG rings with a common AB ring aldehyde precedes rhodium‐catalyzed [2+2+2] alkyne cyclotrimerization to form the natural product skeleton, with the syntheses completed in just one further operation. This work resolves the uncertainty surrounding the identity of *pseudo*‐rubriflordilactone B and provides a robust platform for further synthetic and biological investigations.

## Graphical abstract

**Rubri B or not Rubri B?** A convergent late‐stage fragment coupling and rhodium‐catalyzed [2+2+2] alkyne cyclotrimerization strategy provides access to the natural product (−)‐rubriflordilactone B, and the proposed structure of (−)‐*pseudo*‐rubriflordilactone B. Thus, the uncertainty surrounding the identity of *pseudo*‐rubriflordilactone B is resolved, and a synthetic platform that offers broad scope for the exploration of this natural product family is established.

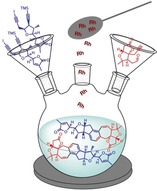

Plants of the genus *Schisandra* produce an array of nortriterpenoids characterized by densely functionalized polycyclic skeletons.^1^ Extracts from these plants feature prominently in traditional Chinese medicine, and many of their natural products have been found to display antiviral and anticancer activities. Stimulated by their structures and properties, syntheses of a number of these molecules have been achieved.^2^ Among the many family members, rubriflordilactones A and B (isolated by Sun and co‐workers from *Schisandra rubriflora*)^3^ are of interest due to their contrasting anti‐HIV bioactivities and, from a synthetic perspective, the challenge of efficient assembly of their polysubstituted arene cores. Syntheses of rubriflordilactone A were described by the Li group,^4^ and by ourselves.^5^ However, the subsequent completion of rubriflordilactone B (**1**, Scheme 1) by Li et al.^6^, ^7^ revealed a structural ambiguity: the NMR spectroscopic data of synthetic **1** (the form of the natural product characterized by X‐ray crystallographic analysis) did not match that recorded for rubriflordilactone B by the Sun group.^3^ This finding appeared to imply the existence of two rubriflordilactone B natural products—one corresponding to the X‐ray structure, and the other to the NMR spectroscopic data.

**Scheme 1:**
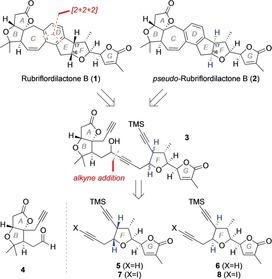
Rubriflordilactone B natural products, and our synthetic strategy.

Subsequent computational work by Kaufman and Sarotti^8^ suggested that the difference between the two forms of rubriflordilactone B lies at C16 and C17 in the EF rings, leading to the proposal of **2**, dubbed *pseudo*‐rubriflordilactone B, as the most likely candidate to fit the NMR data reported in the isolation paper. Herein, we describe our efforts to solve this stereochemical puzzle, first by establishing a robust synthetic route to rubriflordilactone B, and second by modification of this route to access the proposed structure of *pseudo*‐rubriflordilactone B. In combination with studies from the Li group,^9^ we demonstrate that this proposed structure is indeed the likely identity of this natural product.

Our strategy towards these targets derived from our studies on rubriflordilactone A, with the central arene D ring being a focal point for scaffold disconnection.5a Specifically, we viewed the [2+2+2] cyclotrimerization^10^ of suitable triynes **3** as offering a convergent and stereochemically flexible approach to the natural products. These triynes conveniently derive from the common AB ring aldehyde **4**,5a and diynes such as **5** (required for rubriflordilactone B, **1**) and **6** (required for *pseudo*‐rubriflordilactone B, **2**). Mindful of the potential sensitivity of these fragments under the basic conditions required for their union with **4**, we also contemplated equivalent iodoalkynes **7** and **8** which would enable a milder Nozaki–Hiyama–Kishi connection.^11^

Diyne **7** was selected as our initial target. A particular challenge in this fragment is the need to install four contiguous stereocenters on the tetrahydrofuran ring, three of which we envisaged could be created using a dianionic Ireland–Claisen rearrangement of the (*Z*)‐enolate of a suitable ester.^12^ Starting from aldehyde **9 (**Scheme 2), enantioselective [2+2] cycloaddition^13^ with ketene afforded β‐lactone **10**. Ring‐opening of this lactone with the magnesium alkoxide of enantioenriched alcohol **11** (obtained by enzymatic resolution)^14^ afforded ester **12**. Dianionic Ireland–Claisen rearrangement followed by treatment with TMS diazomethane gave ester **13**, featuring three of the required F ring stereocenters;^15^ this ring was formed by oxidative cleavage of the alkene, and cyclization to the methyl acetal **14**. Manipulation of the two oxygen‐bearing sidechains gave diyne **15**, acetoxylation of which afforded acetal **16**. Reaction of the terminal alkyne in **16** with iodine and morpholine^16^ in turn generated iodoalkyne **17**. Finally, the butenolide G ring was introduced through Lewis acid promoted addition of siloxyfuran **18**. As observed by Peng and co‐workers, bismuth(III) triflate proved highly efficient in this process,7c, 17 affording the butenolide diastereomers **7 a** and **7 b** in good yields. As expected on steric grounds, these adducts were formed with good control over the new F ring stereocenter, but as a mixture of stereoisomers on the G ring.^15^ The identity and absolute configuration of **7 b** (and therefore by inference **7 a**, required for rubriflordilactone B) was confirmed by X‐ray crystallographic analysis.^18^

**Scheme 2:**
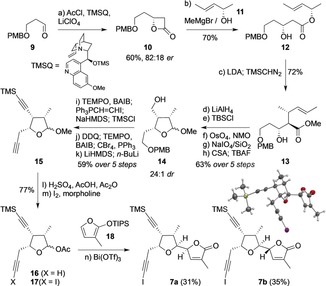
Synthesis of rubriflordilactone B diyne fragments. Ac=acetyl; BAIB=PhI(OAc)_2_; CSA=(±)‐camphorsulfonic acid; DDQ=2,3‐dichloro‐5,6‐dicyano‐1,4‐benzoquinone; LDA=LiN(*i*‐Pr)_2_; LiHMDS=LiN(SiMe_3_)_2_; NaHMDS=NaN(SiMe_3_)_2_; NMO=*N*‐methylmorpholine‐*N*‐oxide; PMB=4‐methoxybenzyl; TBAF=*n*‐Bu_4_NF; TEMPO=(2,2,6,6‐tetramethylpiperidin‐1‐yl)oxyl; Tf=SO_2_CF_3_; TIPS=Si(*i*‐Pr)_3_; TMS=SiMe_3_.

Synthesis of the diyne diastereomer required for *pseudo*‐rubriflordilactone B necessitated a change of strategy, as the configuration of this F ring was now not readily suited to a Claisen rearrangement approach. This route instead began with enantioenriched β‐hydroxy ester **19 (**Scheme 3), prepared by Noyori hydrogenation^19^ of the corresponding known β‐ketoester.^20^ Allylation of the enolate derived from **19** afforded **20** with high selectivity (10:1 *dr*) which,^21^ after further transformations according to the previous route, led to lactone **21**. With two substituents positioned on the β‐face of this ring, methylation of the lactone enolate gave trisubstituted lactone **22** as a single stereoisomer, featuring three of the F‐ring stereocenters required for the predicted natural product. After conversion of this lactone to acetal **23**, completion of diyne **8** entailed an equivalent sequence of steps as employed for its diastereomer **7**. In this case however, the final butenolide addition proceeded without stereocontrol, delivering the four diastereomeric FG diynes **8 a**–**d** in an approximately equimolar ratio. The formation of all four isomers greatly aided assignment of stereochemistry at the new F and G ring stereocenters by a combination of ^1^H NMR NOE enhancements around the tetrahydrofuran framework, and coupling constant analysis.^15^ **8 b** and **8 c** (the identity of the latter being confirmed by X‐ray analysis) were isolated as single stereoisomers, but **8 a** and **8 d** proved inseparable.

**Scheme 3:**
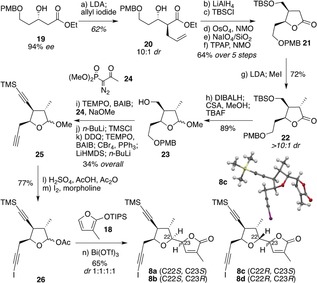
Synthesis of *pseudo*‐rubriflordilactone B diyne fragments. DIBALH=*i*‐Bu_2_AlH; TBS=SiMe_2_ *t*‐Bu; TPAP=tetrapropylammonium perruthenate.

In our previous work,5a we had constructed the arene D ring through palladium‐ and cobalt‐catalyzed cyclizations of bromoendiynes and triynes, respectively. The former proved high yielding but required a non‐terminal alkyne (which in the present context would necessitate later stage arene desilylation), and also temporary protection of the propargylic alcohol. Under cobalt catalysis, the challenge of 7‐membered C‐ring formation called for microwave heating, which limited scalability, and alternative catalyst systems were therefore considered. Rhodium‐catalyzed [2+2+2] alkyne cyclotrimerization also has a rich history in synthetic contexts,^22^ and subsequent to our work on rubriflordilactone A, was reported to promote efficient cyclization to a truncated rubriflordilactone B fragment.7a

To model the cyclization and synthesis endgame, diynes **16** and **17** were first added to hept‐6‐ynal (Scheme 4). This union proved far more efficient under Nozaki–Hiyama–Kishi conditions (with **17**, 73 %) than through the alkynyllithium (**16**, 35 %), presumably reflecting the base‐sensitivity of the acetoxy acetal. To our delight, subjection of the desilylated triyne derived from **27** to rhodium‐catalyzed cyclotrimerization using Wilkinson's catalyst (10 mol %) gave CDEF rings **28** in 73 % yield. Dehydration of the benzylic alcohol was achieved on treatment with Martin sulfurane^23^ (**29**), and finally bismuth(III)‐promoted addition of furan **18** gave the CDEFG model **30** in 27 % yield, along with its separable C23 epimer.

**Scheme 4:**
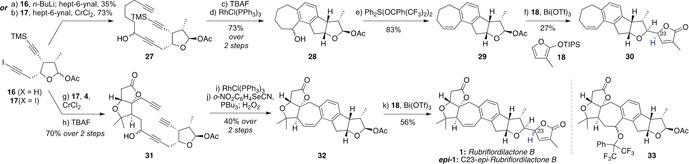
Late‐stage introduction of the butenolide G ring towards rubriflordilactone B.

Encouraged by these results, an equivalent sequence was pursued using the AB ring aldehyde **4**. Rhodium‐catalyzed cyclotrimerization of adduct **31** again proceeded efficiently to give the corresponding hexacycle; due to co‐elution with residual catalyst, this was carried directly to the next step after a short silica gel filtration. However, attempted dehydration of the resulting benzylic alcohol using Martin sulfurane surprisingly yielded the substitution product **33**, likely reflecting the increased steric hindrance towards elimination (or subtle conformational effects) in this substrate compared to **28**. Fortunately, elimination could be effected using the Grieco protocol,^24^ which gave **32** in 40 % yield over the two steps. Bismuth‐promoted reaction of **32** with furan **18** delivered rubriflordilactone B (**1**), as a 1:1 inseparable mixture with its C23 epimer ***epi*** **‐1**.^15^

Although the natural product had been successfully accessed, the problem of C23 isomer separation directed us to install the butenolide ring at an earlier stage, which in any event would represent a more convergent route (Scheme 5). Separate treatment of **4** with iodoalkynes **7 a** and **7 b** led, after alkyne desilylation, to adducts **34** and **35** as inconsequential mixtures at the propargylic alcohol stereocenters. To our delight, cyclotrimerization of these compounds indeed tolerated the butenolide ring, giving the full natural product frameworks (65 % and 68 % yield, respectively). With the more acid‐stable butenolide ring now in place, dehydration could now be effected using *p*‐TsOH (20 mol %) in warm toluene,7a which again afforded rubriflordilactone B **1**, and its C23 diastereomer ***epi*** **‐1**. The former not only matched the spectroscopic data reported by the Li group, but X‐ray crystallographic analysis of **1** enabled unambiguous confirmation of its structure and absolute configuration (Flack(*x*) parameter=−0.09(2)).^18^

**Scheme 5:**
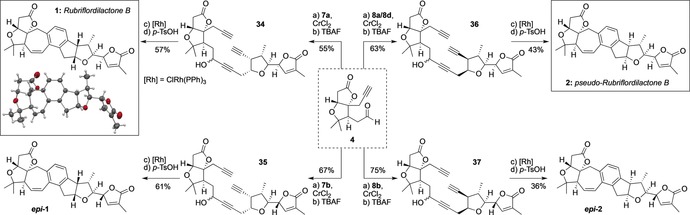
Synthesis of rubriflordilactone B and *pseudo*‐rubriflordilactone B. *p*‐Ts=*p*‐toluenesulfonyl.

Completion of *pseudo*‐rubriflordilactone B (**2**) proved more challenging. As iodoalkynes **8 a** and **8 d** were inseparable, this necessitated addition of this 1:1 mixture to **4**, which fortunately gave separable homopropargylic alcohols. Desilylation of the adduct destined for conversion to *pseudo*‐rubriflordilactone B (**36**) proved somewhat capricious, and both the subsequent cyclotrimerization and acid‐mediated dehydration required higher loadings of their respective catalysts, and/or extended reaction times, compared to rubriflordilactone B. This route nonetheless delivered **2**, the predicted structure of *pseudo*‐rubriflordilactone B. Diyne **8 b** was also carried through the sequence, giving the C23 epimer of *pseudo*‐rubriflordilactone B, ***epi*** **‐2**. Comparison of the NMR spectroscopic data of **2** (and ***epi*** **‐2**) with data from the isolation paper revealed a high level of consistency for the former diastereomer, thus providing strong support for the computationally‐predicted stereochemistry;^8^ further communications with the Li group supported this conclusion.^[9, 25}^

In summary, we have synthesized four diastereomers of rubriflordilactone B, one corresponding to the isolation crystal structure of the natural product, and another to the computationally‐predicted structure of *pseudo*‐rubriflordilactone B, which corresponds to the isolation NMR spectroscopic data. Key to this work was the stereoselective synthesis of the requisite FG ring diynes, and a highly convergent late‐stage coupling/ cyclotrimerization/ dehydration strategy. These results resolve the stereochemical ambiguity surrounding this intriguing natural product and offer a strategy suitable for natural product diversification.

## Conflict of interest

The authors declare no conflict of interest.

## Supporting information

As a service to our authors and readers, this journal provides supporting information supplied by the authors. Such materials are peer reviewed and may be re‐organized for online delivery, but are not copy‐edited or typeset. Technical support issues arising from supporting information (other than missing files) should be addressed to the authors.

Supplementary
