# Supplementary material for: Convergent Total Syntheses of (−)‐Rubriflordilactone B and (−)‐pseudo‐Rubriflordilactone B
Source: Angew Chem Int Ed Engl. 2019 Oct 31;58(50):18177–81. doi: 10.1002/anie.201908917 (PMC6973266; doi:10.1002/anie.201908917)

Angewandte Chemie
Eine Zeitschrift der Gesellschaft Deutscher Chemiker

Supporting Information

# Convergent Total Syntheses of (−)-Rubriflordilactone B and (−)-*pseudo*-Rubriflordilactone B

*Mujahid Mohammad, Venkaiah Chintalapudi, Jeffrey M. Carney, Steven J. Mansfield, Pollyanna Sanderson, Kirsten E. Christensen, and Edward A. Anderson**

anie_201908917_sm_miscellaneous_information.pdf

# Supporting Information

## Contents


**1. General experimental details** **S2**

**2. Experimental procedures and characterization data** **S3**

**3. Data comparison tables of natural products** **S42**

**4. X-ray crystallographic data for compounds 1, 7b, 16a and 8c** **S52**

**5. Copies of NMR spectra** **S59**

- **Rubriflordilactone B** **S106**
- ***Epi*-rubriflordilactone B** **S109**
- ***Pseudo*-rubriflordilactone B** **S112**
- ***Epi-pseudo*-rubriflordilactone B** **S119**

## 1. General Experimental Considerations

*NMR Spectroscopy:* NMR spectra were acquired on Bruker DRX500, AVII500 (cryoprobe) or AVIII400 spectrometers, and were referenced to residual non-deuterated solvent peaks in $CDCl_3$ (δ = 7.26) or $C_5D_5N$ (δ = 8.74, 7.58, 7.22). $^{13}C$ NMR spectra were obtained on Bruker AVII500 (126 MHz, with cryoprobe) or AVIII400 (100 MHz) spectrometers and were referenced to solvent peaks in $CDCl_3$ (d = 77.2) or $C_5D_5N$ (150.3, 135.9, 123.9). Chemical shifts ($\delta_H$ and $\delta_C$) are reported in parts per million (ppm) with signal splittings recorded as singlet (s), doublet (d), triplet (t), quartet (q), quintet (quin), and multiplet (m); app = apparent. Coupling constants (*J*) are measured to the nearest 0.1 Hz and are presented as observed.

*Mass Spectrometry:* Low-resolution mass spectra (*m/z*) were recorded on a Waters LCT Premier EX mass spectrometer, using electrospray ionization (ESI). High-resolution mass spectra (HRMS) were recorded by the Departmental Mass Spectrometry Service, University of Oxford on a Bruker MicroTOF (resolution = 5000 FWHM) using electrospray ionisation ($ES^+$). The parent ion $[M]^+$, $[M+H]^+$ or $[M+Na]^+$ is calculated to 4 decimal places from the molecular formula, and all values are within a tolerance of 5 ppm.

*Infrared Spectroscopy:* Absorption spectra were obtained on a Bruker Tensor 27 FT-IR spectrometer. The sample was prepared as a thin film on a diamond/ZnSe PIKE Miracle ATR module. Wavelengths of maximum absorbance ($\nu_{max}$) are quoted in wavenumbers ($cm^{-1}$). Only selected, characteristic IR absorption data are provided for each compound.

*Specific rotations:* Optical rotations were recorded on a Perkin Elmer 241 or 341 polarimeter with a path length of 1 dm (sodium D line, 589 nm). Specific rotations ($[\alpha]_D^{25}$) are reported in units of $10^{-1}$ deg $cm^2$ $g^{-1}$. Concentrations are reported in g/100 mL. Temperatures are reported in °C (typically 25 °C).

*Chromatography:* Flash chromatography refers to normal phase column chromatography on silica gel using a head pressure of $N_2$, using either Merck Geduran® Silicagel 60 (40-63 mm) or Macherey-Nagel Silica 60 M (40-63 mm). Thin-layer chromatography was performed on Merck Kieselgel 60 $F_{254}$ plates with visualization by ultraviolet light (254 nm) and/or heating the plate after staining with vanillin or $KMnO_4$. High performance liquid chromatography (HPLC) was performed on an Agilent 1200 Series running in normal phase under UV detection using a ZORBAX RX-SIL (150 mm x 4.6 mm ID) as the analytical column. Chiral analysis was carried out using DAICEL CHIRALPAK-IA, IB or IC (250 mm x 4.6 mm ID).

*Materials:* Unless otherwise stated, all reactions were carried out in oven-dried glassware under an atmosphere of argon, using anhydrous reaction solvents. $Et_2O$, $CH_2Cl_2$, THF and toluene were dried over activated alumina before use. All other commercially available reagents and solvents were either used as received, and/or dried and purified before use using standard procedures. petroleum ether refers to the fraction of light petroleum ether boiling at 40-60 °C unless stated otherwise.

## 2. Experimental procedures and characterization data

**(*R*)-4-(2-((4-methoxybenzyl)oxy)ethyl)oxetan-2-one, 10**

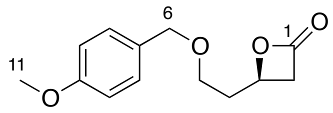

To a solution of TMSQ (1.02 g, 2.56 mmol, 0.1 equiv.)[1] and $LiClO_4$ (819 mg, 7.70 mmol, 0.3 equiv.) in $Et_2O$ (20 mL) was added $CH_2Cl_2$ (20 mL). The reaction mixture was cooled to –78 °C. To this was added DIPEA (12.0 mL, 71.8 mmol, 2.8 equiv.), followed by aldehyde **10** (4.98 g, 25.7 mmol, 1.0 equiv.). A solution of acetyl chloride (3.30 mL, 50.5 mmol, 1.8 equiv.) in $CH_2Cl_2$ (20 mL) was added over 3 h *via* syringe pump, and the resulting solution was stirred at –78 °C for 21 h. The reaction was quenched by addition of $Et_2O$ (50 mL) followed by saturated aq. $NH_4Cl$ (50 mL). It was then extracted using $CH_2Cl_2$ (3 x 50 mL). The combined organic layer was dried using $Na_2SO_4$, concentrated, and purified using column chromatography (7:3 petroleum ether / $Et_2O$) to yield **10** (*er 82:*18, 4.36 g, 18.5 mmol, 72 %).

**$R_f$** 0.12 (4:1 petroleum ether/EtOAc); **IR** (thin film) $\nu_{max}/cm^{-1}$: 2866, 1822, 1612, 1586, 1464, 1302, 1175, 938, 822, 756; **$^1$H NMR** (400 MHz, $CDCl_3$) $\delta_H$ 7.24 (2H, d, *J* = 8.4 Hz, H8), 6.88 (2H, d, *J* = 8.4, H9), 4.71-4.66 (1H, m, H3), 4.43 (2H, s, H6), 3.80 (3H, s, H11), 3.61-3.56 (2H, m, H5), 3.37 (1H, dd, *J* = 13.2 and 5.6 Hz, H2), 3.33 (1H, dd, *J* = 13.2 and 5.6 Hz, H2) 2.16-2.02 (2H, m, H4); **$^{13}$C NMR** (100 MHz, $CDCl_3$) $\delta_C$ 168.4 (C=O), 159.4 (C10), 130.0 (C7), 129.3 (C8), 128.5, 113.9 (C9), 73.0 (C6) , 69.2 (C5), 65.5 (C3), 55.3 (C11), 43.2 (C2), 34.8 (C4); **Anal.** (%) calcd for $C_{13}H_{16}O_4$: C 66.09; H 6.83. Found: C 66.18; H 6.72.

Enantiomeric excess was determined by chiral HPLC: *er* 82:18 (CHIRALPAK IB, 1.0 mL/min, 5% IPA/hexanes, Rt (*S*)-17.05 min, Rt (*R*)-17.97 min).

**(*R*)-(*S*,*E*)-pent-3-en-2-yl 3-hydroxy-5-((4-methoxybenzyl)oxy)pentanoate, 12**

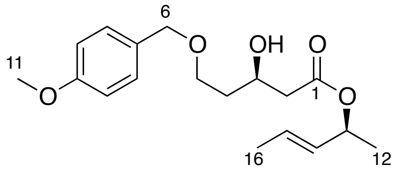

To a solution of alcohol **11** (1.55 g, 18.0 mmol, 1.8 equiv.) in THF (35 mL) at –20 °C was added MeMgBr (6 mL, 18.0 mmol, 3 M in THF, 1.8 equiv.). The solution was stirred for 20 min followed by the addition of a solution of **10** (2.49 g, 9.99 mmol, 1.0 equiv.) in THF (10 mL). The resulting solution was slowly warmed to rt over 3 h. The reaction was quenched by addition of saturated aq. $NH_4Cl$ (50 mL), the layers were separated, and the aqueous layer was extracted with $Et_2O$ (4 x 50 mL). The combined organic layers were dried ($Na_2SO_4$) and concentrated *in vacuo*. The residue was purified *via* column chromatography (3:2→1:1 petroleum ether / $Et_2O$) to afford **12** (2.07 g, 6.00 mmol, 60%, 82:18 mixture of diastereomers at C3) as a colourless oil.

**$R_f$** 0.22 (3:2 Petroleum Ether/$Et_2O$); **$[\alpha]_D^{25}$** –34 (*c* = 1.00, $CHCl_3$) (this value was obtained from **12** prepared through a different, but lengthier route involving asymmetric allylation to set the C3 stereocentre. Detqails of this will be disclosed in due course); **IR** (thin film) $\nu_{max}/cm^{-1}$ 2936, 2860, 1727, 1613, 1586, 1513, 1444, 1370,

1247, 1208, 1173, 1094; **$^1$H NMR** (400 MHz, $CDCl_3$) $\delta_H$ 7.19 (2H, d, $J$ = 8.4 Hz, H8), 6.75 (2H, d, $J$ = 8.4, H9), 5.70-5.63 (1H, dq, $J$ = 13.0 and 6.5 Hz, H15), 5.44-5.38 (1H, m, H14), 5.32-5.25 (1H, m, H13), 4.39 (2H, s, H6), 4.17-4.13 (1H, m, H3), 3.74 (3H, s, H11), 3.63-3.52 (2H, m, H5), 3.3 (1H, br, OH), 2.41 (2H, d, $J$ = 6.4 Hz, H2), 1.75-1.69 (2H, m, H4), 1.64-1.62 (3H, m, H16), 1.22 (3H, d, $J$ = 6.4 Hz, H12); **$^{13}$C NMR** (100 MHz, $CDCl_3$) $\delta_C$ 171.8 (C=O), 159.3 (C10), 130.6 (C14), 130.2 (C7), 129.3 (C8), 128.5 (C15), 113.9 (C9), 72.9 (C6), 71.5 (C13), 67.6 (C5), 66.9 (C3), 55.3 (C11), 41.9 (C2), 36.0 (C4), 20.3 (C12), 17.7 (C16). **HRMS** ($ESI^+$) calculated for $C_{18}H_{26}O_5$[M+Na]$^+$ 345.1673, found 345.1665.

**(2*R*,3*R*,*E*)-Methyl 2-((*R*)-1-hydroxy-3-((4-methoxybenzyl)oxy)propyl)-3-methylhex-4-enoate, 13**

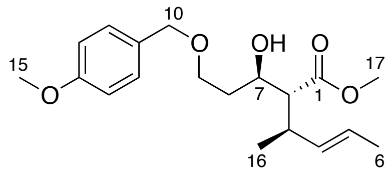


To a solution of *i*-$Pr_2NH$ (2.10 mL, 14.9 mmol, 2.5 equiv.) in THF (10 mL) at 0 °C under argon was added *n*-BuLi (2.5 M in hexanes, 5.95 mL, 14.9 mmol, 2.5 equiv.) dropwise. After 10 min, the resulting solution of LDA was added dropwise, over 1 h, to a stirred suspension of **12** (1.92 g, 5.95 mmol, 1.0 equiv.) in THF (90 mL) at –78 °C under argon. The reaction was further stirred for 90 min then allowed to warm to RT and stirred for another 24 h. It was then acidified to pH 2 with 1 N HCl. The layers were separated, and the aqueous layer extracted with EtOAc (3 × 15 mL). The combined organic layers were dried over $Na_2SO_4$ and concentrated. This residue was dissolved in toluene (120 mL) and MeOH (30 mL) and placed under Ar. To the stirred solution at 0 °C was added 3.9 mL $TMSCHN_2$ (2 M in hexane, 1.35 equiv.). The mixture was stirred at 0 °C for 30 min and then at RT for 90 min before being quenched with acetic acid (3.0 mL). The resulting solution was extracted with EtOAc (3 × 25 mL), dried over $Na_2SO_4$, concentrated and purified *via* flash chromatography (8:2 petroleum ether / $Et_2O$) to afford ester **13** (1.42 g, 4.28 mmol, 72%, 82:18 mixture of diastereomers at C7). A small amount of the ester was carefully purified for the purpose of characterisation.

**$R_f$** 0.33 (1:1 petroleum ether / $Et_2O$); **$[\alpha]_D^{25}$** +5.9 (c = 0.64, $CHCl_3$); **IR** (thin film) $\nu_{max}$/cm$^{-1}$ 3504, 2954, 2858, 1732, 1613, 1586, 1514, 1362, 1248, 1035, 970; **$^1$H NMR** (400 MHz, $CDCl_3$) $\delta_H$ 7.23 (2H, d, $J$ = 8.6 Hz, H12), 6.85 (2H, $J$ = 8.6 Hz, H13) 5.51-5.28 (2H, m, H4, H5), 4.42 (2H, s, H10), 3.96 (1H, m, H7), 3.78 (3H, s, H15) 3.68-3.55 (2H, m, H9), 3.65 (3H, s, H17), 2.63-2.53 (1H, m, H3), 2.46 (1H, d, $J$ = 6.3 Hz, OH), 2.28-2.24 (1H, dd, $J$ = 4.8 Hz, H2), 1.8-1.72 (1H, m, H8), 1.69-1.64 (1H, m, H8) 1.6 (3H, d, $J$ = 5.9 Hz, H6), 1.05 (3H, d, $J$ = 6.8 Hz, H16); **$^{13}$C NMR** (100 MHz, $CDCl_3$) $\delta_C$ 175.0 (C=O), 159.2 (C14), 133.6 (C5), 130.3 (C11), 129.3 (C12), 125.2 (C4), 113.8 (C13), 72.9 (C17) 68.2 (C10), 67.5 (C7), 56.6 (C9), 55.2 (C15), 51.1 (C2), 36.7 (C3), 35.6 (C8), 18.7 (C16), 17.8 (C6); **HRMS** ($ESI^+$) calculated for $C_{19}H_{29}O_5$ [M+H]$^+$ 337.2010, found 337.2016.

**(2*S*,3*R*)-5-((4-methoxybenzyl)oxy)-2-((*R*,*E*)-pent-3-en-2-yl)pentane-1,3-diol, S1**

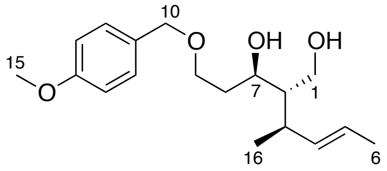

To a stirred solution of **13** (2.00 g, 5.96 mmol, 1.0 equiv.) in $Et_2O$ (40 mL) at –78 °C was added $LiAlH_4$ (3.0 mL, 4M in $Et_2O$, 12.0 mmol, 2.0 equiv.) dropwise. The reaction was allowed to warm to RT over 3 h, then it was quenched by addition of 1 N NaOH (50 mL) and extracted with $Et_2O$ (3 x 30 mL). The combined organic layers were dried over $Na_2SO_4$, concentrated, and purified by flash column chromatography (2:3 petroleum ether / $Et_2O$) to afford **S1** (1.45 g, 4.71 mmol, 79%).

**$R_f$** 0.23 (3:7 EtOAc/petroleum ether); **$[\alpha]_D^{25}$** +13.7 (c = 1.02, $CHCl_3$); **IR** (thin film) $\nu_{max}/cm^{-1}$: 3429, 2959, 2916, 2855, 1613, 1586, 1439, 1351, 1248, 1175, 1094, 1035, 971, 849; **$^1$H NMR** (400 MHz, $CDCl_3$) $\delta_H$ 7.23 (2H, d, *J* = 8.6 Hz, H12) , 6.87 (2H, *J* = 8.6 Hz, H13), 5.47-5.40 (1H, m, H5), 5.30-5.24 (1H, dd, *J* = 8.9 Hz and 15.6 Hz, H4), 4.45 (2H, m, H10), 4.03 (1H, m, H7), 3.88-3.82 (1H, m, OH), 3.8 (3H, s, H15), 3.79-3.60 (4H, m, H1, H9), 3.23 (1H, t, *J* = 5.4 Hz, OH), 2.47-2.38 (1H, m, H3), 2.03-1.91 (1H, m, H8), 1.79-1.73 (1H, m, H8), 1.64 (3H, dd, *J* = 1.4 Hz and 6.1 Hz, H6), 1.23-1.28 (1H, m, H2), 1.06 (3H, d, *J* = 6.8 Hz, H16); **$^{13}$C NMR** (100 MHz, $CDCl_3$) $\delta_C$ 159.4 (C14), 134.5 (C5), 129.8 (C11), 129.4 (C12), 125.0 (C4), 113.9 (C13), 74.2 (C7) 73.1 (C10), 69.4 (C9) ,62.06 (C1), 55.8 (C15), 50.1 (C2), 35.6 (C3), 35.1 (C8), 19.2 (C6), 17.9 (C16); **HRMS** ($ESI^+$) calculated for $C_{18}H_{28}O_4Na$ $[M+Na]^+$ 331.1880, found 331.1873.

**(3*R*,4*S*,5*R*,*E*)-4-(((*tert*-butyldimethylsilyl)oxy)methyl)-1-((4-methoxybenzyl)oxy)-5-methyloct-6-en-3-ol, S2**

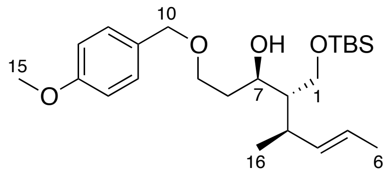

To a solution of **S1** (2.67 g, 8.67 mmol, 1.0 equiv.) in $CH_2Cl_2$ (21 mL) at 0 °C was added TBSCl (1.57 g, 10.4 mmol, 1.2 equiv.), DMAP (106 mg, 0.87 mmol, 0.1 equiv.) and imidazole (1.18 g, 17.3 mmol, 2.0 equiv.). The mixture was allowed to stir at 0 °C for 50 min before quenching with water (50 mL). It was then extracted with $Et_2O$ (3 x 25 mL), dried ($Na_2SO_4$), and concentrated. Purification by flash column chromatography (4:1 petroleum ether / $Et_2O$) afforded **S2** (3.37 g, 7.97 mmol, 92%) as a yellow oil.

**$R_f$** 0.36 (4:1 petroleum ether / $Et_2O$); **$[\alpha]_D^{25}$** +17 (c = 0.99, $CHCl_3$); **IR** (thin film) $\nu_{max}/cm^{-1}$: 3512, 2927, 2047, 1741, 1584, 1362, 1249, 1172, 1075, 969; **$^1$H NMR** (400 MHz, $CDCl_3$) $\delta_H$ 7.26 (2H, d, *J* = 8.6 Hz, H12), 6.87 (2H, *J* = 8.6 Hz, H13), 5.47-5.39 (1H, m, H5), 5.28-5.21 (1H, m, H4), 4.46 (2H, t, *J* = 12.3 Hz, H10), 3.98-3.87 (2H, m, H1, H7), 3.82-3.79 (1H, m, H1), 3.80 (3H, s, H15), 3.64 (2H, t, *J* = 6.6 Hz, H9), 3.53 (1H, d, *J* = 7.3 Hz, OH), 2.61-2.52 (1H, m, H3), 1.93-1.85 (1H, m , H8), 1.82-1.73 (1H, m, H8), 1.65 (3H, d, *J* = 6.0 Hz, H6), 1.15-1.10 (1H, m, H2), 1.06 (3H, d, *J* = 6.88 Hz, H16), 0.89 (9H, s, $SiC(CH_3)_3$), 0.05 (3H, s, $Si(CH_3)_2$), 0.04 (3H, s, $Si(CH_3)_2$); **$^{13}$C NMR** (100 MHz, $CDCl_3$) $\delta_C$ 159.3 (C14), 135.9 (C5), 130.8 (C11), 129.4 (C12), 124.8 (C4), 113.9 (C13), 73.0 (C7) 70.0 (C10), 68.1 (C9), 62.5 (C1), 55.4 (C15), 48.8 (C2), 36.7 (C3), 35.4

(C8), 25.9 (SiC(CH$_3$)$_3$), 19.2 (C6), 18.0 (SiC) 17.9 (C16), -5.5 and -5.6 (SiC(CH$_3$)$_2$); **HRMS** (ESI$^+$) calcd for $C_{18}H_{27}O_6$ [M+Na]$^+$ 445.2745, found 445.2749.

**(4*S*,5*S*,6*R*)-5-(((tert-butyldimethylsilyl)oxy)methyl)-8-((4-methoxybenzyl)oxy)-4-methyloctane-2,3,6-triol, S3**

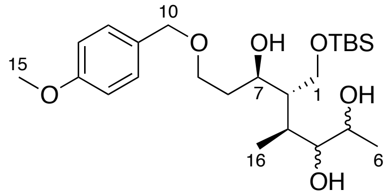


To a stirred solution of alkene **S2** (612 mg, 1.45 mmol, 1.0 equiv.) in acetone (14 mL) and water (4.6 mL) was sequentially added $OsO_4$ (4% wt in water, 0.37 mL, 0.058 mmol, 0.04 equiv.) and NMO (50% wt in water, 0.61 mL, 2.61 mmol, 1.8 equiv.). The reaction mixture was stirred at RT for 3 h before being quenched with sat. *aq*. $Na_2S_2O_3$ solution (20 mL). The layers were separated, and the aqueous layer extracted with EtOAc (3 x 20 mL). The combined organic layers were dried with $Na_2SO_4$, and concentrated. The crude product was purified by flash column chromatography through a short plug of silica (49:1 $CH_2Cl_2$ / MeOH) to afford the triols **S3** (686 mg, 1.45 mmol, 2.8:1 inconsequential mixture of diastereomers at C7 and C8, 99%) as a yellow oil.

**R$_f$** 0.3 (49:1 DCM/MeOH); **IR** (thin film) $\nu_{max}$/cm$^{-1}$ : 3373, 2959, 1514, 1464, 1362, 1259, 1088, 1038, 836, 802, 703; **$^1$H NMR** (400 MHz, CDCl$_3$) $\delta_H$ 7.23 (2H, d, *J* = 8.6 Hz, H13), 6.87 (2H, *J* = 8.6 Hz, H12) , 4.43 (2H, s, H10), 4.08-3.17 (12H, m, H1, H4, H5, H7, H9 and H15), 2.04 (1H, m, H2), 2.02 (1H, m, H3), 1.90 (2H, m, H8), 1.22 (3H, d, H6), 1.03 (3H, d, H16) 0.93 (9H, s, SiC(CH$_3$)$_3$), 0.02 (3H, s, Si(CH$_3$)$_3$); 0.01 (3H, s, Si(CH$_3$)$_3$); **$^{13}$C NMR** (100 MHz, CDCl$_3$) $\delta_C$ 159.3 (C14), 130.0 (C11), 129.9 (C12), 113.9 (C13), 76.9 (C10), 73.0 (C7), 69.6 (C4, C5), 69.0 (C4, C5), 67.8 (C9), 61.0 (C1), 55.3 (C15), 49.1 (C2), 37.7 (C3), 35.4 (C8), 25.9 (SiC(CH$_3$)$_3$), 20.8 (C6), 17.2 (C16), 11.3 (SiC), –5.6 (SiC(CH$_3$)$_2$); **HRMS** (ESI$^+$) calculated for $C_{24}H_{45}O_6Si$ [M+H]$^+$ 457.2980, found 457.2976.

**(3*S*,4*S*,5*R*)-4-(((*Tert*-butyldimethylsilyl)oxy)methyl)-5-(2-((4-methoxybenzyl)oxy)ethyl)-3-methyltetrahydrofuran-2-ol, S4**

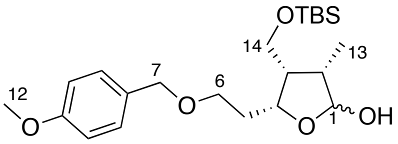


To a stirred solution of triol **S3** (660 mg, 1.45 mmol, 1.0 equiv.) in $CH_2Cl_2$ (16 mL) was added $NaIO_4$ on silica (10 wt%, 3.71 g, 1.73 mmol, 1.2 equiv.). The suspension was stirred for 30 min before passed through a short plug of silica, concentrated, and purified by flash column chromatography (99:1 $CH_2Cl_2$ / MeOH) to afford lactols **S4** (578 mg, 1.41 mmol, 97%, 2.5:1 inseparable inconsequential mixture of epimers) as a yellow oil.

**R$_f$** 0.56 (49:1 DCM / MeOH); **IR** (thin film) $\nu_{max}$/cm$^{-1}$ 3479, 2955, 2857, 1514, 1250, 1096, 1039, 837; **$^1$H NMR** (400 MHz, CDCl$_3$) $\delta_H$ 7.26 (2H, d, *J* = 8.6 Hz, H10), 6.87 (2H, *J* = 8.6 Hz, H9), 5.10 (1H, m, H1), 4.50

(2H, m, H7), 4.26 (1H, m, H4), 3.84 (1H, d, $J$ = 10.2 Hz, H14), 3.80 (3H, s, H12), 3.72-3.58 (3H, m, H6, H14), 2.55-2.45 (1H, m, H2) 2.14-1.82 (3H, m, H3, H5), 1.08 (3H, d, $J$ = 7.2 Hz, H13), 0.95 (9H, s, $SiC(CH_3)_3$), 0.15 (6H, s, $Si(CH_3)_3$); **$^{13}$C NMR** (100 MHz, $CDCl_3$) $\delta_C$ 159.1 (C11), 130.7 (C8), 129.3 (C9), 113.8 (C10), 99.0 (C1), 72.8 (C7), 67.9 (C4), 59.2 (C6), 55.2 (C12 and C14), 45.6 (C5), 41.4 (C2), 30.3 (C3), 25.8 ($SiC(CH_3)_3$), 18.2 (C13), 12.5 (SiC), -5.5 ($SiCH_3$) and -5.6 ($SiCH_3$); **HRMS** ($ESI^+$) calculated for $C_{22}H_{38}O_5SiNa$ $[M+Na]^+$ 433.2381, found 433.2379.

**((2*R*,3*S*,4*S*)-5-Methoxy-2-(2-((4-methoxybenzyl)oxy)ethyl)-4-methyltetrahydrofuran-3-yl)methanol, 14**

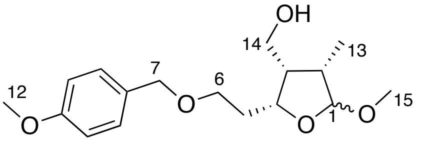


To a stirred solution of lactols **S4** (578 mg, 1.41 mmol, 1.0 equiv.) in MeOH (55 mL) at 0 °C was added (±)-CSA (26 mg, 0.11 mmol, 0.08 equiv.). The mixture was stirred at 0 °C for 3 h before addition of 0.1 mL $Et_3N$, then it was concentrated. The residue was taken up in THF (24 mL), cooled to 0 °C under argon, and TBAF (1 M in THF, 1.68 mL, 1.2 equiv.) was added. The reaction was warmed to rt overnight, then quenched by addition of sat. *aq*. $NH_4Cl$ (10 mL). The layers were separated, and the aqueous layer was extracted with $Et_2O$ (3 x 20 mL). The combined organic layers were dried over $Na_2SO_4$, concentrated, and the residue was purified *via* column chromatography (1:1 petroleum ether / $Et_2O$) to afford **14** (307 mg, 1.09 mmol, 88%) as a colourless oil.

**$R_f$** 0.17 (3:7 petroleum ether / $Et_2O$); **$[\alpha]_D^{25}$** +66 (c = 1.00, $CHCl_3$); **IR** (thin film) $\nu_{max}/cm^{-1}$ 3444, 2932, 1613, 1586, 1513, 1461, 1364, 1247, 1174, 1094, 1031, 976; **$^1$H NMR** (400 MHz, $CDCl_3$) $\delta_H$ 7.25 (2H, d, $J$ = 8.2 Hz, H9), 6.87 (2H, $J$ = 8.6 Hz, H10), 4.59 (1H, d, $J$ = 3.2 Hz, H1), 4.45 (2H, s, H7), 4.29 – 4.20 (1H, m, H4), 3.80 (3H, s, H12), 3.76 (1H, dd, $J$ = 11.2, 4.0 Hz, H14), 3.70 – 3.60 (2H, m, H14, H6), 3.60–3.52 (1H, m, H6), 3.36 (3H, s, C15), 2.38 – 2.22 (2H, m, H2 and H3), 2.08 – 1.99 (1H, m, H5), 1.95–1.87 (1H, m, H5), 1.10 (3H, d, $J$ = 7.2 Hz, H13); **$^{13}$C NMR** (100 MHz, $CDCl_3$) $\delta_C$ 159.3 (C11), 130.2 (C8), 129.3 (C9), 113.8 (C10), 110.5 (C1), 78.2 (C4), 72.9 (C7), 67.8 (C6), 58.8 (C14), 55.6 (C15), 55.3 (C12), 46.1 (C3), 43.1 (C2), 31.4 (C5), 12.3 (C13); **HRMS** ($ESI^+$) calc. for $C_{17}H_{26}O_5Na$ $[M+Na]^+$ 333.1673, found 333.1673.

**(((2*R*,3*S*,4*S*)-5-Methoxy-2-(2-((4-methoxybenzyl)oxy)ethyl)-4-methyltetrahydrofuran-3-yl)ethynyl)trimethylsilane, S5**

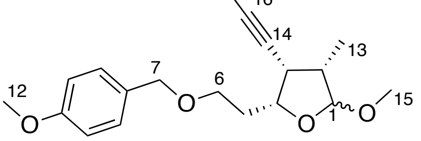


To a stirred solution of **14** (721 mg, 2.32 mmol, 1.0 equiv.) in $CH_2Cl_2$ (28 mL) and water (28 mL) was added TEMPO (73 mg, 0.47 mmol, 0.2 equiv.) and BAIB (973 mg, 3.01 mmol, 1.3 equiv.). The resulting biphasic solution was stirred for 3 h. The reaction was monitored using TLC and on completion, 3 mL of MeOH was added and the solution was stirred for another 2 h. The layers were then separated, and the aqueous layer was

extracted with $Et_2O$ (3 x 50 mL). The combined organic layers were dried over $Na_2SO_4$, concentrated and dried under vacuum to give a crude oil.

To a stirred suspension of (iodomethyl)triphenylphosphonium iodide (2.22 g, 4.18 mmol, 1.8 equiv.) in a flame dried flask in THF (35 mL) under argon at RT was added NaHMDS (1.98 mL, 3.95 mmol, 2 M in THF, 1.7 equiv.). After 20 min, the solution was cooled to –78 °C and a solution of the crude product from the previous step in THF (15 mL) was added. After 15 min, the reaction mixture was warmed to rt over 30 min until TLC showed complete formation of the *cis*-vinyl iodide derivative.

The reaction mixture was then cooled to –78 °C, and additional NaHMDS (3.50 mL, 7.00 mmol, 2 M in THF, 3.0 equiv.) was added. The reaction mixture was warmed to rt over 30 min and stirred for a further 10 min. The reaction was again cooled to –78 °C followed by addition of further NaHMDS (0.81 mL, 1.63 mmol, 2 M in THF, 0.7 equiv.) before adding TMSCl (0.89 mL, 7.00 mmol, 3.0 equiv.). The reaction was warmed to –20 °C before being quenched with saturated aq. $NH_4Cl$ solution. $Et_2O$ was added, the layers were separated, and the aqueous layer extracted three times with $Et_2O$ (3 x 100 mL). The combined organic layers were dried with $Na_2SO_4$, and concentrated. The crude product was purified by flash column chromatography (19:1 petroleum ether / $Et_2O$) to give alkyne **S5** (719 mg, 1.90 mmol, 82% over four steps) as a colourless oil.

**R$_f$** 0.14 (23:1 petroleum ether / $Et_2O$); **$[\alpha]_D^{25}$** +36 (c = 0.98, $CHCl_3$); **IR** (thin film) $\nu_{max}$/cm$^{-1}$: 2957, 2165, 1613, 1513, 1248, 1173, 1098, 1017, 944, 913; **$^1$H NMR** (400 MHz, $CDCl_3$) $\delta_H$ 7.27 (2H, d, *J* = 7.0 Hz, H9), 6.87 (2H, *J* = 7.7 Hz, H10), 4.63 (1H, d, *J* = 3.2 Hz, H1) 4.45 (2H, s, H7), 4.23 (1H, q, *J* = 6.1 and 13.2 Hz, H4) 3.80 (3H, s, H12), 3.60 (2H, t, *J* = 6.1 Hz, H6), 3.36 (3H, s, H15), 3.10 (1H, t, *J* = 6.0 Hz, H3), 2.34-2.24 (1H, m, H2), 2.04 (2H, q, *J* = 5.7 and 12.5 Hz, H5), 1.18 (3H, d, *J* = 7.2 Hz, H13), 0.15 (9H, s, Si($CH_3$)$_3$); **$^{13}$C NMR** (100 MHz, $CDCl_3$) $\delta_C$ 159.0, 130.6, 129.1, 113.7, 110.1, 102.5, 90.9, 76.5, 72.5, 67.0, 55.6, 55.2, 43.6, 40.3, 32.6, 13.8, 0.0; **HRMS** (ESI$^+$) calc. for $C_{21}H_{32}O_4NaSi$ [M+Na]$^+$ 399.1962, found 399.1962.

**(((2*R*,3*S*,4*S*)-2-(3,3-dibromoallyl)-5-methoxy-4-methyltetrahydrofuran-3-yl)ethynyl)trimethylsilane, S6**

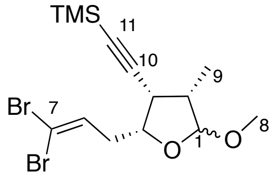


To a solution of PMB ether **S5** (193 mg, 0.51 mmol, 1.0 equiv.) in $CH_2Cl_2$ (8.5 mL) and water (3.0 mL), was added DDQ (175 mg, 0.768 mmol, 1.5 equiv.). The solution was allowed to stir for 45 min, followed by the addition of saturated $NaHCO_3$ solution. This was stirred for 15 min followed by extraction using $CH_2Cl_2$ (3 x 25 mL). The combined organic layers were dried over $Na_2SO_4$, and concentrated; the crude material was carried forward without purification to the next step.

To a solution of the crude alcohol in $CH_2Cl_2$ (10 mL) and water (10 mL) was added TEMPO (16 mg, 0.10 mmol, 0.2 equiv.) and BAIB (248 mg, 0.77 mmol, 1.5 equiv.). The biphasic mixture was stirred at rt for 3 h. The reaction was monitored by TLC and on completion, 0.5 mL MeOH was added. The reaction was stirred for 2 h followed by separation of the layers. The aqueous layer was extracted with $CH_2Cl_2$ (3 x 25 mL) and the

combined organic layers were dried ($Na_2SO_4$) and concentrated. The crude material was carried forward to the next step without further purification.

To a solution of $PPh_3$ (713 mg, 2.56 mmol, 5.0 equiv.) in $CH_2Cl_2$ (5 mL) at 0 °C was added $CBr_4$ (425 mg, 1.28 mmol, 2.5 equiv.). The mixture was stirred at 0 °C for 15 min before being cooled to −30 °C. To this was added dropwise a solution of the crude aldehyde and $Et_3N$ (0.71 mL, 5.13 mmol, 10 equiv.) in $CH_2Cl_2$ (3 mL), and the reaction mixture was warmed to 0 °C over 1h. The reaction was quenched by addition of with saturated aq. $NH_4Cl$, the layers were separated and the aqueous layer extracted with DCM (3 x 15 mL). The combined organic layers were dried ($Na_2SO_4$) and concentrated, and the residue was purified via flash column chromatography (99:1 petroleum ether / $Et_2O$) to yield dibromoalkene **S6** (166 mg, 0.405 mmol, 79% over three steps) as a colourless oil.

**R$_f$** 0.49 (24:1 petroleum ether / $Et_2O$); **$[\alpha]_D^{25}$** +28 (c = 0.91, $CHCl_3$); **IR** (thin film) $\nu_{max}$/cm$^{-1}$: 2961, 2170, 1625, 1455, 1249, 1197, 1100, 1081, 999, 839, 793; **$^1$H NMR** (400 MHz, $CDCl_3$) $\delta_H$ 6.54 (1H, t, J = 7.1, H6), 4.66 (1H, d, *J*= 3.6 Hz, H1), 4.15 – 4.09 (1H, m, H4), 3.36 (3H, s, H7), 3.18 (1H, dd, *J* = 6.4 and 7.1 Hz, H3), 2.56 – 2.46 (2H, m, H5), 1.17 (3H, d, *J*= 7.2 Hz, H8), 0.17 (9H, s, $Si(CH_3)_3$); **$^{13}$C NMR** (100 MHz, $CDCl_3$) $\delta_C$ 134.9 110.3, 101.8, 91.7, 90.2, 77.1, 55.7, 43.4, 39.8, 36.6, 13.9, 0.0; **HRMS** (ESI$^+$) calc. for $C_{14}H_{23}{}^{79}Br_2O_2Si$ $[M+H]^+$ 408.9829, found 408.9830.

**(((2*R*,3*S*,4*S*)-5-methoxy-4-methyl-2-(prop-2-yn-1-yl)tetrahydrofuran-3-yl)ethynyl)trimethylsilane, 15**

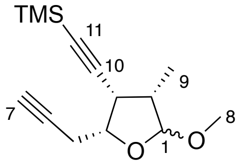


To a solution of dibromide **S6** (145 mg, 0.354 mmol, 1.0 equiv.) in THF (3.2 mL) under Ar at −78 °C was added LiHMDS (0.530 mL, 0.531 mmol, 1 M in THF, 1.5 equiv.). The solution was warmed to −15 °C over 2 h, then cooled again to −78 °C. To it was added *n*-BuLi (0.220 mL, 0.531 mmol, 2.5 M in hexanes, 1.5 equiv.) dropwise along the wall of the vial, and the mixture was stirred for 5 min. On completion as judged by TLC, a solution of saturated aq. $NH_4Cl$ was added at −78 °C. The mixture was warmed to rt and extracted with $Et_2O$ (3 x 15 mL), and the combined organic layers were dried ($Na_2SO_4$), and concentrated. The crude product was purified via flash column chromatography (19:1 petroleum ether / $Et_2O$) to yield diyne **15** (79 mg, 0.319 mmol, 90%) as a colourless oil.

**R$_f$** 0.34 (19:1 petroleum ether / $Et_2O$); **$[\alpha]_D^{25}$** +37 (c = 0.97, $CHCl_3$); **IR** (thin film) $\nu_{max}$/cm$^{-1}$: 2959, 2932, 2170, 1456, 1384, 1368, 1250, 1197, 1156, 1133, 1005, 968; **$^1$H NMR** (400 MHz, $CDCl_3$) $\delta_H$ 4.68 (1H, d, *J*= 3.6 Hz, H1), 4.27 – 4.21 (1H, m, H4) , 3.38 (3H, s, H7), 3.26 (1H, t, *J* = 6.4 Hz, H3), 2.64 – 2.59 (2H, m, H5), 2.35 – 2.25 (1H, m, H2), 2.00 (1H, t, *J* = 2.7 Hz, H7), 1.18 (3H, d, *J* = 7.27 Hz, H8), 0.16 (9H, s, $Si(CH_3)_3$); **$^{13}$C NMR** (100 MHz, $CDCl_3$) $\delta_C$ 110.4, 101.5, 91.6, 80.8, 77.7, 69.6, 55.8, 43.3, 39.8, 22.7, 13.8, 0.0; **HRMS** (ESI$^+$) calculated for $C_{14}H_{22}O_2SiNa$ $[M+Na]^+$ 273.1281, found 273.1282.

**(3*S*,4*S*,5*R*)-3-methyl-5-(prop-2-yn-1-yl)-4-((trimethylsilyl)ethynyl)tetrahydrofuran-2-yl acetate, 16**

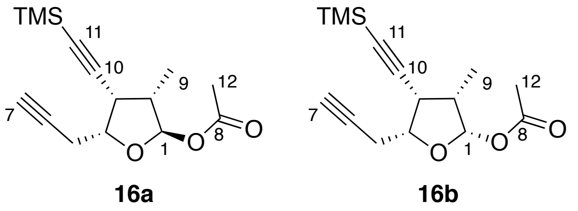

To a solution of methyl acetal **15** (140 mg, 0.561 mmol, 1.0 equiv.) in glacial AcOH (3.5 mL) at 0 °C was added acetic anhydride (0.300 mL, 3.14 mmol, 6.0 equiv.). Along the walls of the vial, conc. $H_2SO_4$ (15 µL, 0.28 mmol, 0.5 equiv.) was added, and the wall was rinsed with THF (3.5 mL). The solution was stirred at 0 °C for 2 h. On completion as judged by TLC, the reaction was quenched with saturated aq. $NaHCO_3$, and the extracted with $Et_2O$ (4 x 5 mL). The combined organic layers were dried ($Na_2SO_4$) and concentrated, and the residue was purified via flash column chromatography (19:1 petroleum ether / $Et_2O$) to give acetate **16** (131 mg, 0.471 mmol, 84%, 3:1 mixture) as a white solid. The mixture was used for the subsequent reactions unless stated otherwise. From a small amount of **16**, diastereomers **16a** and **16b** were separated for the purpose of characterisation. The structure of **16a** was confirmed using single crystal X-ray diffraction. See SI p48 for details.

**16a: R$_f$** 0.47 (4:1 petroleum ether / $Et_2O$); **$[\alpha]_D^{25}$** +23 (c = 1.1, $CHCl_3$); **IR** (thin film) $\nu_{max}$/cm$^{-1}$ 2980, 2961, 2924, 2169, 1743, 1376, 1248, 1236, 1060, 1009, 843; **$^1$H NMR** (400 MHz, $CDCl_3$) $\delta_H$ 5.94 (1H, d, *J* = 3.9 Hz, H1), 4.38 – 4.31 (1H, m, *J* = 6.1 Hz and 13.1 Hz, H4), 3.34 (1H, dd, *J* = 1.0 Hz and 6.3 Hz, H3), 2.72 – 2.61 (2H, m, H5) , 2.52 – 2.41 (1H, m, H2), 2.06 (3H, s, H12), 2.00 (1H, t, *J* = 2.7 Hz, H7), 1.21 (3H, d, *J* = 7.2 Hz, H9), 0.16 (s, $Si(CH_3)_3$); **$^{13}$C NMR** (101 MHz, $CDCl_3$) $\delta_C$ 170.4, 103.0, 100.4, 92.4, 80.3, 79.6, 69.9, 43.1, 39.7, 22.5, 21.3, 13.6, -0.0; **mp** 82-86 °C; **HRMS** (ESI$^+$) calc. for $C_{15}H_{22}O_3NaSi$ $[M+Na]^+$ 301.1230; found 301.1230.

**16b: R$_f$** 0.34 (4:1 petroleum ether / $Et_2O$); **$[\alpha]_D^{25}$** –49 (c = 0.55, $CHCl_3$); **IR** (thin film) $\nu_{max}$/cm$^{-1}$: 2960, 2926, 2855, 2171, 1744, 1375, 1250, 1230, 1064, 1011, 990.2, 843; **$^1$H NMR** (400 MHz, $CDCl_3$) $\delta_H$ 6.16 (1H, d, *J* = 4.8 Hz, H1), 4.25 (1H, *app* q, *J* = 6.7 Hz, H4), 3.22 (1H, t, *J* = 6.8 Hz, H3), 2.73 – 2.67 (2H, m, H5), 2.55 – 2.47 (1H, m, H2), 2.05 (3H, s, H12), 1.18 (3H, d, *J* = 7.0 Hz, H9), 0.18 (9H, s, $Si(CH_3)_3$); **$^{13}$C NMR** (101 MHz, $CDCl_3$) $\delta_C$ 170.2, 100.9, 98.7, 91.6, 80.7, 69.7, 41.4, 38.4, 23.5, 21.2, 10.2, -0.0; **mp** 58-64 °C; **HRMS** (ESI$^+$) calc. for $C_{15}H_{22}O_3NaSi$ $[M+Na]^+$ 301.1230; found 301.1230.

**(3*S*,4*S*,5*R*)-5-(3-Iodoprop-2-yn-1-yl)-3-methyl-4-((trimethylsilyl)ethynyl)tetrahydrofuran-2-yl acetate, 17**

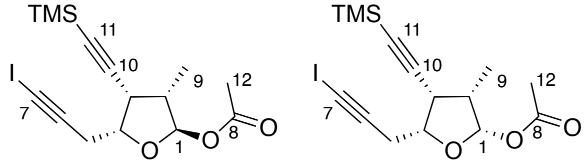

**17a** **17b**

To a solution of $I_2$ (962 mg, 3.78 mmol, 10 equiv.) in toluene (8 mL) was added morpholine (0.66 mL, 7.59 mmol, 20 equiv.) was added dropwise and the resulting mixture was sonicated for 10 min before being warmed to 45 °C. The stirring was continued for 30 min before adding a solution of acetate **16** (106 mg, 0.38 mmol, 1.0 equiv.) in toluene (2 mL). The resulting mixture was stirred at 45 °C overnight before being cooled to rt and quenched with saturated aq. $Na_2SO_3$ (50 mL). The resulting biphasic mixture was vigorously stirred for 30 min before being extracted with $Et_2O$ (4 x 20 mL). The combined organic layers were dried using $Na_2SO_4$, concentrated, and the residue purified by flash column chromatography (19:1 petroleum ether / $Et_2O$) to give iodoalkyne **17** (140 mg, 0.346 mmol, 91%, 3:1 mixture of diastereomers) as white solids. The mixture was used for the subsequent reactions unless stated otherwise. From a small amount of **17**, diastereomers **17a** and **17b** were separated for the purpose of characterisation.

**17a: R$_f$** 0.47 (4:1 petroleum ether / $Et_2O$); **$[\alpha]_D^{25}$** −16 (c = 1.00, $CHCl_3$); **IR** (thin film) $\nu_{max}/cm^{-1}$ 2970, 2171, 1738, 1457, 1376, 1249, 1130, 1009, 915, 843; **$^1$H NMR** (400 MHz, $CDCl_3$) $\delta_H$ 5.93 (1H, d, *J* = 3.7 Hz, H1), 4.37 – 4.28 (1H, m, H4), 3.34 (1H, d, *J* = 13.3 Hz, H3), 2.85 – 2.77 (2H, m, H5), 2.48 – 2.36 (1H, m, H2), 2.06 (3H, s, H12), 1.20 (3H, d, *J* = 7.2 Hz, H9), 0.16 (9H, s, $Si(CH_3)_3$); **$^{13}$C NMR** (101 MHz, $CDCl_3$) $\delta_C$ 170.4, 102.9, 100.4, 92.5, 90.2, 79.4, 43.0, 39.5, 24.8, 21.2, 13.6, -0.0, -4.5; **HRMS** (ESI$^+$) calculated for $C_{15}H_{21}IO_3NaSi$ $[M+Na]^+$ 427.0197 found 427.0198.

**17b: R$_f$** 0.34 (4:1 petroleum ether / $Et_2O$); **$[\alpha]_D^{25}$** −77 (c = 0.99, $CHCl_3$); **IR** (thin film) $\nu_{max}/cm^{-1}$ 2980, 2173, 1739, 1458, 1374, 1248, 1164, 1133, 1058, 907, 843; $^1$H NMR (400 MHz, $CDCl_3$) $\delta_H$ 6.11 (1H, d, *J* = 4.8 Hz, H1), 4.21 (1H, *app* q, *J* = 6.7 Hz, H4), 3.19 (1H, dd, *J* = 4.6 Hz and 5.6 Hz, H3), 2.84 (2H, q, *J* = 3.1 Hz and 6.6 Hz, H5), 2.51 – 2.40 (1H, m, H2), 2.01 (3H, s, H12), 1.15 (3H, d, *J* = 7.0 Hz, H9), 0.16 (s, 9H, $Si(CH_3)_3$); **$^{13}$C NMR** (101 MHz, $CDCl_3$) $\delta_C$ 170.1, 100.9, 98.7, 91.6, 90.5, 80.5, 41.3, 38.3, 25.8, 21.1, 10.2, -0.0, -4.8; **HRMS** (ESI$^+$) calculated for $C_{15}H_{21}IO_3NaSi$ $[M+Na]^+$ 427.0197 found 427.0197.

**(*S*)-5-((2*S*,3*S*,4*S*,5*R*)-5-(3-iodoprop-2-yn-1-yl)-3-methyl-4-((trimethylsilyl)ethynyl)tetrahydro-furan-2-yl)-3-methylfuran-2(5*H*)-one, 7a, and (*R*)-5-((2*S*,3*S*,4*S*,5*R*)-5-(3-iodoprop-2-yn-1-yl)-3-methyl-4-((trimethylsilyl)ethynyl)tetrahydrofuran-2-yl)-3-methylfuran-2(5*H*)-one, 7b**

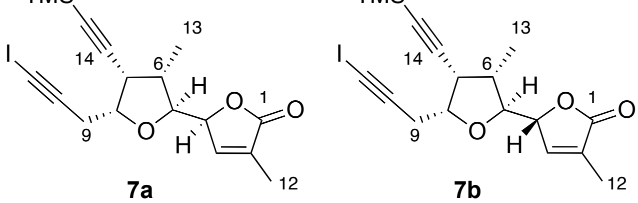

**7a** **7b**

To a solution of acetates **17a** and **17b** (140 mg, 0.346 mmol, 1.0 equiv.) and silyloxyfuran **18** (0.148 mL, 0.519 mmol, 1.5 equiv.) in anhydrous MeCN (4.5 mL) at –40 °C, was added dropwise a solution of $Bi(OTf)_3$ (454

mg, 0.693 mmol, 2.0 equiv.) in anhydrous MeCN (2.0 mL). The mixture was stirred at –40 °C for 1 h before being quenched with saturated aq. $NH_4Cl$ (20 mL). The solution was warmed to rt, then extracted with $Et_2O$ (4 x 20 mL). The combined organic layers were dried ($Na_2SO_4$**)** and concentrated, and the residue was purified via flash column chromatography (19:1→4:1 petroleum ether / $Et_2O$) to give **7a** (46 mg, 0.107 mmol, 31%, viscous oil) and **7b** (52 mg, 0.121 mmol, 35%, white solid).

**7a** $\mathbf{R_f}$ 0.46 (3:2 petroleum ether / $Et_2O$); $[\alpha]_D^{25}$ –89 (c = 1.01, $CHCl_3$); **IR** (thin film) $\nu_{max}$/cm$^{-1}$ 2959, 2926, 2167, 1760, 1456, 1250, 1085, 1050, 986, 944, 843, 760; **$^1$H NMR** (500 MHz, $CDCl_3$) $\delta_H$ 7.00 (1H, t, *J* = 1.7 Hz, H3), 4.90 (1H, q, *J* = 2.0 Hz, H4), 4.03 (1H, ddd, *J* = 7.5, 6.5, 4.4 Hz, H8), 3.89 (1H, dd, *J* = 9.5, 2.1 Hz, H5), 3.14 (1H, dd, *J* = 6.4, 4.4 Hz, H7), 2.73 (2H, dd, *J* = 7.1, 2.8 Hz, H9), 2.60 (1H, dt, *J* = 9.5, 6.6 Hz, H6), 1.94 (3H, t, *J* = 1.8 Hz, H12), 1.21 (3H, d, *J* = 6.7 Hz, H13), 0.16 (9H, s, $Si(CH_3)_3$); **$^{13}$C NMR** (126 MHz, $CDCl_3$) $\delta_C$ 174.1, 145.9, 131.1, 100.6, 92.7, 90.2, 82.3, 80.1, 79.8, 42.0, 38.9, 24.4, 13.4, 10.8, 0.1, -5.0; **HRMS** (ESI$^+$) calc. for $C_{18}H_{23}IO_3SiNa$ $[M+Na]^+$ 465.0353; found 465.0352.

**7b** $\mathbf{R_f}$ 0.51 (3:2 petroleum ether / $Et_2O$); $[\alpha]_D^{25}$ +18 (c = 0.91, $CHCl_3$); **IR** (thin film) $\nu_{max}$/cm$^{-1}$ 2960, 2926, 2167, 1758, 1457, 1250, 1104, 1043, 993, 843; **$^1$H NMR** (500 MHz, $CDCl_3$) $\delta_H$ 7.15 (1H, t, *J* = 1.7 Hz, H3), 4.84 (1H, dt, *J* = 6.4, 1.9 Hz, H4), 4.13 (1H, td, *J* = 6.9, 4.6 Hz, H8), 3.61 (1H, dd, *J* = 8.8, 6.4 Hz, H5), 3.16 (1H, dd, *J* = 6.5, 4.6 Hz, H7), 2.78 (2H, dd, *J* = 6.9, 3.4 Hz, H9), 2.33 (1H, dt, *J* = 8.9, 6.7 Hz, H6), 1.93 (3H, t, *J* = 1.8 Hz, H12), 1.20 (3H, d, *J* = 6.7 Hz, H13), 0.15 (9H, s, $Si(CH_3)_3$); **$^{13}$C NMR** (126 MHz, $CDCl_3$) $\delta_C$ 174.0, 146.9, 130.8, 100.5, 92.8, 90.5, 84.11, 82.5, 79.5, 42.4, 40.6, 24.6, 14.2, 10.7, 0.0, -4.9; **mp** 108-113°C; **HRMS** (ESI$^+$) calculated for $C_{18}H_{23}IO_3SiNa$ $[M+Na]^+$ 465.0353 found 465.0353.

**Ethyl 5-((4-methoxybenzyl)oxy)-3-oxopentanoate, S7**

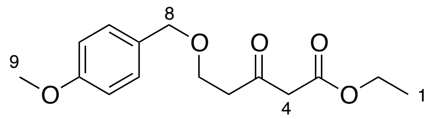


β-ketoester **S7** was prepared according to a literature procedure.[2] Ethyl diazoacetate (2.82 g, 24.7 mmol, 1.2 equiv.) was added with stirring at room temperature to a solution of anhydrous $SnCl_2$ (390 mg, 2.06 mmol, 0.1 equiv.) in $CH_2Cl_2$ (40 mL). A few drops of aldehyde **9** (4.00 g, 20.6 mmol, 1.0 equiv.) in $CH_2Cl_2$ (20 mL) were added to the suspension. After nitrogen evolution had begun, the remaining aldehyde solution was added dropwise over 10 min. The solution was stirred until the evolution of nitrogen had stopped (5 h), and then the mixture was transferred to a separating funnel containing saturated brine (80 mL) and diethyl ether (240 mL). After separation of the layers, the aqueous phase was extracted with diethyl ether (3 x 200 mL). The organic layers were combined, dried ($MgSO_4$), filtered, and concentrated. Flash chromatography of the residue provided the β-ketoester **S7** (4.30 g, 18.54 mmol, 75%) as a slightly yellow oil (keto-enol mixture).

$\mathbf{R_f}$ 0.42 (2:1 petroleum ether / EtOAc); **$^1$H NMR** (400 MHz, $CDCl_3$) $\delta_H$ 7.24 (2H, d, *J* = 8.0 Hz, ArH), 6.87 (2H, d, *J* = 8.8 Hz, ArH), 4.43 (2H, s, H8), 4.28-4.06 (2H, m, H2), 3.79 (3H, s, H9), 3.71 (2H, t, *J* = 6.2 Hz, H7), 3.47 (2H, s, H4), 2.81 (2H, t, *J* = 6.2 Hz, H6), 1.26 (3H, t, *J* = 7.2 Hz, H1); **$^{13}$C NMR** (101 MHz, $CDCl_3$) $\delta_C$ 201.6, 167.2, 159.3, 130.0, 129.4, 113.9, 73.0, 64.7, 61.4, 55.4, 49.8, 43.2, 14.2.

**Ethyl (*S*)-3-hydroxy-5-((4-methoxybenzyl)oxy)pentanoate: 19**

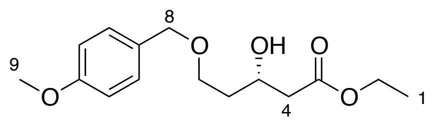

Dry, degassed DMF (3 mL) was added to a flask containing benzene ruthenium(II) chloride dimer (45 mg, 0.090 mmol, 0.03 equiv.) and (*S*)-(–)-BINAP (112 mg, 0.18 mmol, 0.006 equiv.). The slurry was heated to 90 °C with stirring for 20 min. The resulting reddish brown solution was then cooled to rt, and was added via syringe to a Parr flask containing a degassed solution of *β*-ketoester **S7** (8.00 g, 28.6 mmol, 1.0 equiv.) in dry ethanol (15 mL). The hydrogenation flask was flushed a few times with hydrogen, and then pressurized with 5.0 bar hydrogen, and heated to 100 °C with vigorous stirring for 20 h. The solution was then cooled to rt, concentrated, and purified by flash chromatography (4:1 petroleum ether / $Et_2O$) to provide the *β*-hydroxyester **19** (6.45 g, 22.9 mmol, 80%, 94% *ee*[*]) as a pale-yellow liquid.

**R$_f$** 0.36 (4:1 petroleum ether / $Et_2O$); **$[\alpha]_D^{25}$** +7.2 (c = 0.5, $CHCl_3$); **IR** (thin film) $\nu_{max}$/cm$^{-1}$ 3485, 2936, 1730, 1613, 1514, 1173.9, 1092, 1031, 820; **$^1$H NMR** (400 MHz, $CDCl_3$) $\delta_H$ 7.24 (2H, d, *J* = 8.5 Hz, ArH), 6.86 (2H, d, *J* = 8.5 Hz, ArH), 4.43 (2H, s, H8), 4.25-4.19 (1H, br s, H5), 4.14 (2H, q, *J* = 7.2 Hz, H2), 3.79 (3H, s, H9), 3.71-3.56 (2H, m, H7), 3.43 (1H, s, OH), 2.47 (2H, d, *J* = 6.3 Hz, H4), 1.81-1.70 (2H, m, H6), 1.25 (3H, t, *J* = 7.1 Hz, H1); **$^{13}$C NMR** (101 MHz, $CDCl_3$) $\delta_C$ 172.5, 159.3, 130.2, 129.4, 113.9, 73.0, 67.7, 67.1, 60.6, 55.3, 41.7, 36.1, 14.3; **HRMS** (ESI$^+$) calc. for $C_{15}H_{22}O_5Na$ $[M+Na]^+$ 305.1359; found 305.1361.

[*]%*ee* was calculated through Mosher ester analysis of **19** according to the following procedure:

**Ethyl (*S*)-5-((4-methoxybenzyl)oxy)-3-(((*R*)-3,3,3-trifluoro-2-methoxy-2-phenylpropanoyl)oxy)-pentanoate, S8**

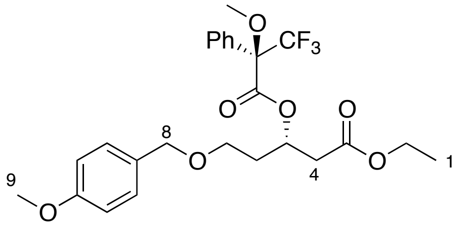

To solution of alcohol **19** (20.0 mg, 0.071 mmol, 1.0 equiv.) in anhydrous $CH_2Cl_2$ (1 mL) was added dry pyridine (0.035 mL, 0.44 mmol, 6.2 equiv.) and (*S*)-(–)-MTPA chloride (0.050 mL, 0.27 mmol, 3.8 equiv.) at rt. The mixture was stirred at ambient temperature for 6 h. The reaction was quenched with water, extracted with $Et_2O$ (3 x 5 mL). The combined organic layers were dried over $MgSO_4$ and purified by flash column chromatography (10:1 petroleum ether / $Et_2O$) to give **S8** (25.0 mg, 0.050 mmol, 71%).

**R$_f$** 0.5 (10:1 petroleum ether / $Et_2O$); **$[\alpha]_D^{25}$** +6.7 (c = 0.50, $CHCl_3$); **IR** (thin film) $\nu_{max}$/cm$^{-1}$ 2852, 1745, 1613, 1514, 1247, 1169, 1018, 820, 717; **$^1$H NMR** (400 MHz, $CDCl_3$) $\delta_H$ 7.55-7.48 (2H, m, ArH), 7.38 (3H, m, ArH), 7.24 (2H, d, *J* = 8.7 Hz, ArH), 6.87 (2H, d, *J* = 8.7 Hz, ArH), 5.63 (1H, m, H5), 4.43-4.37 (2H, m, H8), 4.04 (2H, qd, *J* = 7.1, 2.4 Hz, H2), 3.80 (3H, s, H9), 3.53-3.44 (6H, m, H10 and H7), 2.67 (2H, d, *J* = 6.4 Hz, H4), 2.03 (2H, p, *J* = 6.0 Hz, H6), 1.18 (3H, t, *J* = 7.1 Hz, H1); **$^{13}$C NMR** (101 MHz, $CDCl_3$) $\delta_C$ 169.9, 165.9, 159.4, 132.2, 130.2, 129.7, 129.4, 128.5, 127.6, 123.4 (q, *J* = 288.7 Hz, $CF_3$) 114.0, 72.9, 71.6, 65.7, 60.9, 55.5, 55.4, 39.0, 33.9, 14.2; **HRMS** (ESI$^+$) calc. for $C_{25}H_{29}O_7F_3Na$ $[M+Na]^+$ 521.1758; found 521.1754.

**Ethyl (*S*)-5-((4-methoxybenzyl)oxy)-3-(((*S*)-3,3,3-trifluoro-2-methoxy-2-phenylpropanoyl)oxy)-pentanoate, S9**

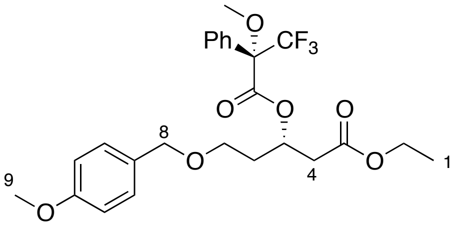


To solution of alcohol **19** (20.0 mg, 0.071 mmol, 1.0 equiv.) in anhydrous $CH_2Cl_2$ (1 mL) was added dry pyridine (0.035 mL, 0.44 mmol, 6.2 equiv.) and (*S*)-(–)-MTPA chloride (0.050 mL, 0.27 mmol, 3.8 equiv.) at rt. The mixture was stirred at ambient temperature for 6 h. The reaction was quenched with water, extracted with $Et_2O$ (3 x 5 mL). The combined organic layers were dried over $MgSO_4$ and purified by flash column chromatography (10:1 petroleum ether / $Et_2O$) to give **S9** (27 mg, 0.055 mmol, 77%).

**R**$_f$ 0.5 (10:1 petroleum ether / $Et_2O$); $[\alpha]_D^{25}$ –20 (c = 0.50, $CHCl_3$); **IR** (thin film) $\nu_{max}$/cm$^{-1}$ 2855, 1745, 1613, 1514, 1247, 1169, 1019, 820, 717; **$^1$H NMR** (400 MHz, $CDCl_3$) $\delta_H$ 7.55-7.50 (2H, m, ArH), 7.43-7.34 (3H, m, ArH), 7.22 (2H, d, *J* = 8.7 Hz, ArH), 6.87 (2H, d, *J* = 8.7 Hz, ArH), 5.63 (1H, m, H5), 4.38-4.27 (2H, m, H8), 4.11 (2H, qd, *J* = 7.1, 1.7 Hz, H2), 3.80 (3H, s, H9), 3.53 (3H, s, H10), 3.43-3.26 (2H, m, H7), 2.71 (2H, d, *J* = 6.3 Hz, H4), 1.96 (2H, ddt, *J* = 7.7, 5.7, 2.7 Hz, H6), 1.22 (3H, t, *J* = 7.1 Hz, H1); **$^{13}$C NMR** (101 MHz, $CDCl_3$) $\delta_C$ 170.1, 166.0, 159.4, 132.4, 130.3, 129.7, 129.4, 128.5, 127.5, 123.4 (q, *J* = 288.5 Hz, $CF_3$), 114.0, 72.8, 71.4, 65.5, 61.0, 55.6, 55.4, 39.2, 33.8, 14.2; **HRMS** (ESI$^+$) calculated for $C_{25}H_{29}O_7F_3Na$ $[M+Na]^+$ 521.1758, found 521.1753.

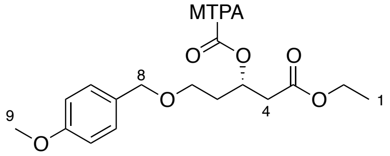


| Proton positions | S-Mosher's ester | R-Mosher's ester | $\Delta\delta_{SR}$ |
|---|---|---|---|
| 1 | 1.22 | 1.18 | 0.04 |
| 2 | 4.11 | 4.04 | 0.07 |
| 4 | 2.71 | 2.67 | 0.04 |
| 5 | 5.63 | 5.63 | 0.00 |
| 6 | 1.96 | 2.03 | – 0.07 |
| 7 | 3.34 | 3.48 | – 0.14 |
| 8 | 4.32 | 4.40 | – 0.08 |

The Mosher esters were analyzed by NMR and the C5 hydroxyl was thus found to be of ***S*** configuration.

**Ethyl (*S*)-2-((*S*)-1-hydroxy-3-((4-methoxybenzyl)oxy)propyl)pent-4-enoate, 20**

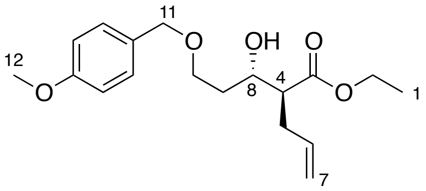

Compound **20** was prepared using a modified literature procedure.[3,4] To a solution of hydroxyester **19** (4.00 g, 14.2 mmol, 1.0 equiv.) in THF (50 mL) at –78 °C was added LDA (17.0 mL, 34.0 mmol, 2M in THF, 2.4 equiv.) dropwise via syringe over 10 min. The mixture was stirred at –78 °C for 1 h, and then warmed to –30 °C for 30 min, then re-cooled to –78 °C. A solution of allyl iodide (2.6 mL, 28.4 mmol, 2.0 equiv.) in HMPA (5.0 mL, 17.0 mmol, 1.2 equiv.) was added, and the reaction was allowed to stir at –78 °C for 4 h before being quenched with saturated aq. $NH_4Cl$ (20 mL) and EtOAc. The mixture was extracted with EtOAc (3 x 100 mL), and the combined organic extracts were washed with brine, dried ($MgSO_4$) and concentrated. The residue was purified by flash chromatography (4:1 petroleum ether / $Et_2O$) to afford the title compound **20** as a mixture of 14:1 mixture of diastereomers (2.65 g, 7.81 mmol, 55%) as yellow oil, along with recovered starting material (600 mg, 2.13 mmol, 15%). The stereochemistry of this compound was confirmed by X-ray crystallography on subsequent derivative **8c**.

**R$_f$** 0.33 (1:1 petroleum ether/$Et_2O$); **$[\alpha]_D^{25}$** +4.5 (c = 0.50, $CHCl_3$); **IR** (thin film) $\nu_{max}/cm^{-1}$ 3486, 2936, 2862, 1730, 1613, 1513, 1176, 1093, 1033, 821, 638; **$^1$H NMR** (500 MHz, $CDCl_3$) $\delta_H$ 7.24 (2H, d, *J* = 8.0 Hz, ArH), 6.88 (2H, d, *J* = 8.0 Hz, ArH), 5.82-5.68 (1H, m, H6), 5.11 (1H, d, *J* = 17.0 Hz, H7), 5.05 (1H, d, *J* = 10.1 Hz, H7). 4.45 (2H, s, H11), 4.17 (2H, q, *J* = 7.0 Hz, H2), 4.01-3.90 (1H, m, H8), 3.81 (3H, s, H12), 3.72-3.66 (1H, m, H10), 3.72-3.60 (2H, m, H10), 2.57-2.49 (1H, m, H4), 2.49-2.33 (2H, m, H5), 1.88-1.70 (2H, m, H9), 1.27 (3H, t, *J* = 7.0 Hz, H1); **$^{13}$C NMR** (126 MHz, $CDCl_3$) $\delta_C$ 174.6, 159.4, 135.1, 130.2, 129.5, 117.2, 114.0, 73.1, 71.0, 68.1, 60.6, 55.4, 51.1, 34.8, 33.5, 14.4; **HRMS** (ESI$^+$) calc. for $C_{18}H_{26}O_5Na$ $[M+Na]^+$ 345.1673l; found 345.1676.

**(2*R*,3*S*)-2-Allyl-5-((4-methoxybenzyl)oxy)pentane-1,3-diol, S10**

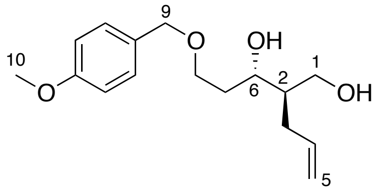

To a stirred solution of **20** (2.50 g, 7.76 mmol, 1.0 equiv.) in ether (75 mL) at –78 °C was added dropwise $LiAlH_4$ (3.90 mL, 15.5 mmol, 4 M in ether 2.0 equiv.). The reaction was stirred at –78 °C for 15 min, and then warmed to rt and stirred for 3 h. The reaction was then quenched by addition of 1 N NaOH (50 mL), and extracted with $Et_2O$ (3 x 100 mL). The combined organic layers were dried ($MgSO_4$), concentrated, and purified by flash chromatography (2:3 petroleum ether / $Et_2O$) to yield **S10** (1.75 g, 6.36 mmol, 82%).

**R$_f$** 0.23 (3:7 ethyl acetate / petroleum ether); **$[\alpha]_D^{25}$** +9.8 (c = 0.50, $CHCl_3$); **IR** (thin film) $\nu_{max}/cm^{-1}$ 3387, 2864, 1613, 1513, 1246.0, 1175, 1086, 1033, 820; **$^1$H NMR** (400 MHz, $CDCl_3$) $\delta_H$ 7.24 (2H, d, *J* = 8.6 Hz, ArH), 6.88 (2H, d, *J* = 8.6 Hz, ArH), 5.87-5.69 (1H, m, H4), 5.09 (1H, s, H5), 5.03 (1H, d, *J* = 11.1 Hz, H5), 4.46

(2H, s, H9), 3.92-3.85 (2H, m, H1), 3.80 (3H, s, H10), 3.76-3.61 (1H, m, H6), 3.69-3.61 (2H, m, H8), 2.25-2.07 (2H, m, H3), 2.00-1.87 (1H, m, H2), 1.81-1.72 (1H, m, H7), 1.61-1.54 (1H, m, H7); **$^{13}$C NMR** (101 MHz, $CDCl_3$) $\delta_C$ 159.5, 136.7, 129.8, 129.5, 116.7, 114.0, 75.9, 73.3, 69.6, 64.1, 55.4, 44.8, 34.7, 33.4; **HRMS** ($ESI^+$) calc. for $C_{16}H_{24}O_4Na$ $[M+Na]^+$ 303.1567; found 303.1567.

**(3*S*,4*R*)-4-(((*Tert*-butyldimethylsilyl)oxy)methyl)-1-((4-methoxybenzyl)oxy)hept-6-en-3-ol, S11**

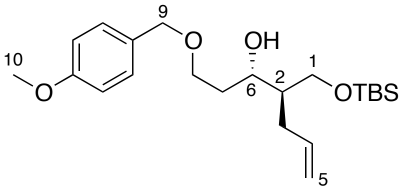


To a solution of diol **S10** (2.48 g, 8.86 mmol, 1.0 equiv.) in $CH_2Cl_2$ (20 mL) at 0 °C was added TBSCl (1.59 g, 10.6 mmol, 1.2 equiv.), DMAP (108 mg, 0.89 mmol, 0.1 equiv.) and imidazole (1.20 g, 17.7 mmol, 2.0 equiv.). The reaction was stirred at 0 °C for 60 min before quenching with water. It was extracted with $Et_2O$ (3 x 100 mL), and the combined organic extracts were dried ($MgSO_4$) and concentrated. Purification by flash chromatography (4:1 petroleum ether / $Et_2O$) gave **S11** (3.40 g, 8.59 mmol, 97%) as a colourless oil.

**$R_f$** 0.36 (4:1 petroleum ether / $Et_2O$); **$[\alpha]_D^{25}$** −1.9 (c = 0.50, $CHCl_3$); **IR** (thin film) $\nu_{max}/cm^{-1}$ 3505, 2929, 2857, 1513, 1464.4, 1248, 1088, 1038, 834, 776; **$^1$H NMR** (400 MHz, $CDCl_3$) $\delta_H$ 7.20 (2H, d, *J* = 8.5 Hz, ArH), 6.81 (2H, d, *J* = 8.5 Hz, ArH), 5.78-5.63 (1H, m, H4), 4.97 (1H, s, H5), 4.98 (1H, d, *J* = 26.4 Hz, H5), 4.39 (2H, s, H9), 3.79 (2H, ddd, *J* = 11.4, 8.8, 3.4 Hz, H1), 3.74 (3H, s, H10), 3.63–3.58 (3H, m, H6 and H8), 3.48 (1H, d, *J* = 5.2 Hz, OH), 2.21 (1H, dt, *J* = 14.7, 8.0 Hz, H3), 2.11 (1H, dt, *J* = 14.7, 8.0 Hz, H3), 1.80–1.72 (2H, m, H7), 1.50 (1H, m, H2), 0.83 (9H, s, $SiC(CH_3)_3$). 0.00 (6H, s, $Si(CH_3)_2$); **$^{13}$C NMR** (101 MHz, $CDCl_3$) δ 159.3, 137.0, 130.6, 129.4, 116.5, 113.8, 73.0, 72.4, 68.4, 63.7, 55.4, 44.5, 35.4, 33.0, 26.0, 18.2, -5.5, -5.5; **HRMS** ($ESI^+$) calc. for $C_{22}H_{38}O_4NaSi$ $[M+Na]^+$ 417.2432; found 417.2435.

**(4*R*,5*S*)-4-(((*Tert*-butyldimethylsilyl)oxy)methyl)-5-(2-((4-methoxybenzyl)oxy)ethyl) dihydrofuran-2(3*H*)-one, 21**

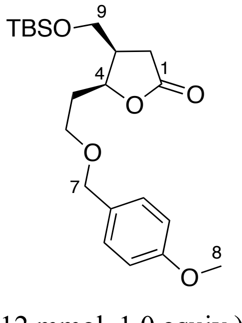


To a stirred solution of alkene **S11** (3.20 g, 8.12 mmol, 1.0 equiv.) in acetone (75 mL) and water (25 mL), was sequentially added $OsO_4$ (4% wt in water, 2.0 mL, 0.325 mmol, 0.04 equiv.) and NMO (50% wt in water, 3.2 mL, 14.6 mmol, 1.8 equiv.). The reaction mixture was stirred at rt for 3 h before being quenched with saturated *aq*. $Na_2S_2O_3$. The mixture was stirred for 1 h, then the layers were separated, and the aqueous layer extracted

with ethyl acetate (3 x 200 mL). The combined organic layers were dried ($MgSO_4$) and concentrated to give the crude triol as a 3:1 mixture of diastereomers, which was carried forward without purification.

To a stirred solution of crude triol in $CH_2Cl_2$ (180 mL) was added $NaIO_4$ on silica (10 wt%, 51.9 g, 24.4 mmol, 3.0 equiv.). The suspension was stirred for 12 h before being filtered through a short plug of silica. Following concentration, the crude lactol was carried forward without further purification.

To a stirred solution of the lactol in $CH_2Cl_2$ (85 mL) at rt were successively added 4Å MS (5.00 g), NMO (2.40 g, 16.2 mmol, 2.0 equiv.) and TPAP (284 mg, 0.812 mmol, 0.1 equiv.). Stirring was continued for 12 h, then the mixture was filtered through a Celite pad, and concentrated. The residue was purified by flash chromatography (9:1 petroleum ether / EtOAc) to give lactone **21** (2.60 g, 6.58 mmol, 81% over 3 steps) as a colourless oil.

**R**$_f$ 0.5 (4:1 petroleum ether / AcOEt); $[\alpha]_D^{25}$ –37 (c = 0.50, $CHCl_3$); **IR** (thin film) $\nu_{max}/cm^{-1}$ 2935, 2862, 1729, 1613, 1513, 1246, 1176, 1093, 1033, 820, 638; **$^1$H NMR** (400 MHz, $CDCl_3$) $\delta_H$ 7.21 (2H, d, *J* = 8.6 Hz, ArH), 6.83 (2H, d, *J* = 8.6 Hz, ArH), 4.69 (1H, q, *J* = 6.6 Hz, H4), 4.48–4.30 (2H, m, H7), 3.76 (3H, s, H8), 3.68–3.50 (4H, m, H6 and H9), 2.60 (1H, dd, *J* = 16.6, 8.4 Hz, H2), 2.56–2.49 (1H, m, H3), 2.39 (1H, dd, *J* = 16.6, 3.1 Hz, H2), 1.99-1.87 (2H, m, H5), 0.83 (9H, s, $SiC(CH_3)_3$), 0.00 (3H, s, $Si(CH_3)_2$), -0.01 (3H, s, $Si(CH_3)_2$); **$^{13}$C NMR** (101 MHz, $CDCl_3$) $\delta_C$ 176.6, 159.3, 130.3, 129.4, 113.9, 79.4, 73.0, 66.6, 61.4, 55.4, 40.0, 33.0, 30.6, 25.8, 18.2, -5.6; **HRMS** ($ESI^+$)calc. for $C_{21}H_{34}O_5NaSi$ $[M+Na]^+$ 417.2432; found 417.2433.

**(3*S*,4*R*,5*S*)-4-(((*Tert*-butyldimethylsilyl)oxy)methyl)-5-(2-((4-methoxybenzyl)oxy)ethyl)-3-methyldihydrofuran-2(3*H*)-one, 22**

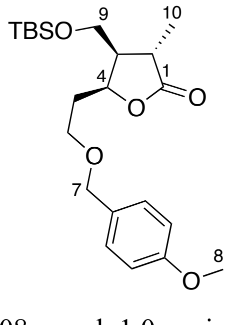


To a stirred solution of lactone **21** (2.00 g, 5.08 mmol, 1.0 equiv.) in THF (32 mL) at –78 °C was added LDA (3.06 mL, 6.12 mmol, 2 M in THF, 1.2 equiv.) dropwise over 10 min. The mixture was stirred at –78 °C for 2 h, then methyl iodide (1.26 mL, 20.3 mmol, 4.0 equiv.) was added dropwise. The reaction was stirred at –78 °C for a further 8 h before being quenched with saturated aqueous $NH_4Cl$ and EtOAc. The mixture was warmed to rt and extracted with EtOAc (3 x 200 mL), and the combined organic extracts were washed with brine, dried ($MgSO_4$) and concentrated. The residue was purified by flash chromatography (4:1 petroleum ether / EtOAc) to afford the title compound **22** (1.56 g, 3.81 mmol, 75%) as yellow liquid (>10:1 *dr*).

**R**$_f$ 0.50 (4:1 petroleum ether / $Et_2O$); $[\alpha]_D^{25}$ –25 (c = 0.50, $CHCl_3$); **IR** (thin film) $\nu_{max}/cm^{-1}$ 2930, 2850, 1725, 1618, 1513, 1246, 1168, 1020, 824, 632; **$^1$H NMR** (400 MHz, $CDCl_3$) $\delta_H$ 7.24 (2H, s, *J* = 8.2 Hz), 6.87 (2H, d, *J* = 8.2 Hz), 4.75 (1H, ddd, *J* = 10.6, 7.2, 2.7 Hz, H4), 4.53–4.31 (2H, m, H7), 3.80 (3H, s, H8), 3.74–3.57 (4H, m, H6 and H9), 2.51 (1H, m, H2), 2.38–2.27 (1H, m, H3), 2.03–1.88 (1H, m, H5), 1.85–1.77 (1H, m,

H5), 1.27 (3H, d, $J$ = 7.2 Hz, H10), 0.87 (9H, s, $SiC(CH_3)_3$ ), 0.04 (3H, s, $Si(CH_3)_2$), 0.04 (3H, s, $Si(CH_3)_2$); **$^{13}$C NMR** (101 MHz, $CDCl_3$) δ 179.6, 159.3, 130.4, 129.4, 113.9, 77.3, 73.0, 66.5, 61.0, 55.4, 47.8, 36.9, 30.8, 25.9, 18.2, 14.8, -5.4, -5.5; **HRMS** ($ESI^+$) calc. for $C_{22}H_{36}O_5SiNa$ $[M+Na]^+$ 431.2224; found 431.2223.

**((2*S*,3*R*,4*S*)-5-Methoxy-2-(2-((4-methoxybenzyl)oxy)ethyl)-4-methyltetrahydrofuran-3-yl)methanol, 23**

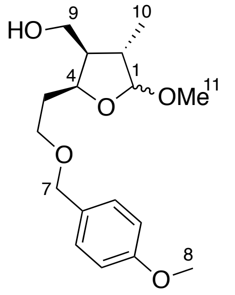


To a stirred solution of lactone **22** (905 mg, 2.21 mmol, 1.0 equiv.) in $CH_2Cl_2$ (18 mL) at –78 ºC was added DIBALH (4.0 mL, 3.76 mmol, 0.94 M in THF, 1.7 equiv.) dropwise. The reaction was stirred for 1 h, then it was quenched with 1 M NaOH (10 mL) at –78 °C, allowed to warm to rt, and diluted with dichloromethane (20 mL) and water (20 mL). The layers were separated, and the aqueous layer was extracted with dichloromethane (3 x 10 mL). The organic layers were combined, washed with brine (50 mL), dried over $Na_2SO_4$, and concentrated to give a mixture of lactols.

To a stirred solution of these lactols in MeOH (80 mL) at 0 °C was added (±)-CSA (41 mg, 0.18 mmol, 0.08 equiv.). The reaction was stirred at 0 °C for 3 h, then $Et_3N$ (2 mL) was added, and the reaction was concentrated. The residue was used directly in the next step without further purification.

To a stirring solution of methyl acetals in THF (24 mL) at 0 °C was added TBAF (1 M in THF, 2.43 mL, 2.43 mmol, 1.1 equiv.). The reaction was warmed to rt overnight, then quenched with saturated *aq*. $NH_4Cl$ (10 mL). The layers were separated, and the aqueous layer extracted with $Et_2O$ (3 × 20 mL). The combined organic layers were dried ($Na_2SO_4$) and concentrated. The residue was purified *via* column chromatography (1:1 petroleum ether / $Et_2O$) to give a mixture of 1:1 mixture of diastereomers (610 mg, 1.97 mmol, 89% over three steps) as a colourless oil.

**$R_f$** 0.17 (3:7 petroleum ether / $Et_2O$); **IR** (thin film) $\nu_{max}$/cm$^{-1}$ 3432, 2930, 2862, 1613, 1586, 1247, 1093, 1032, 1001, 820.6; **$^{1}$H NMR** (400 MHz, $CDCl_3$) $\delta_H$ 7.30 (2H, d, $J$ = 8.8 Hz, ArH), 6.91 (2H, d, $J$ = 8.8 Hz, ArH), 4.77 (0.5H, d, $J$ = 4.9 Hz, H1), 4.59 (0.5H, d, $J$ = 2.3 Hz, H1), 4.53–4.46 (2H, m, H7), 4.40 (0.5H, ddd, $J$ = 9.3, 6.3, 4.3 Hz, H4), 4.31 (0.5H, ddd, $J$ = 9.1, 7.9, 4.1 Hz, H4), 3.82 (3H, s, H8), 3.73–3.56 (4H, m, H6 and H9), 3.37 (1.5H, s, H11), 3.36 (1.5H, s, H11), 2.17 (0.5H, ddd, $J$ = 8.0, 6.6, 3.8 Hz, H2), 2.12–2.00 (1.5H, m, H2 and H5), 2.00-1.86 (1.5H, m, H3 and H5), 1.80–1.71 (0.5H, m, H3), 1.14 (1.5H, d, $J$ = 6.7 Hz, H10), 1.06 (1.5H, d, $J$ = 6.9 Hz, H10); **$^{13}$C NMR** (101 MHz, $CDCl_3$) $\delta_C$ 159.2, 159.2, 130.4, 130.3, 129.4, 129.4, 113.8, 113.8, 111.4, 105.1, 78.4, 76.5, 72.9, 72.8, 68.1, 67.9, 61.8, 61.6, 55.3, 55.3, 55.3, 54.7, 50.6, 48.4, 43.2, 40.6, 31.6, 31.1, 17.2, 12.6; **HRMS** ($ESI^+$) calc. for $C_{17}H_{26}O_5Na$ $[M+Na]^+$ 333.1673; found 333.1673.

**(2*S*,3*R*,4*S*)-3-Ethynyl-5-methoxy-2-(2-((4-methoxybenzyl)oxy)ethyl)-4-methyltetrahydrofuran, S12**

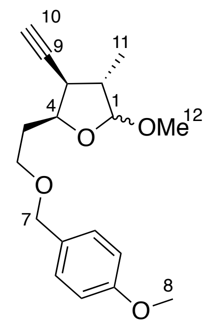


To a stirred solution of alcohol **23** (410 mg, 1.32 mmol, 1.0 equiv.) in $CH_2Cl_2$ (7 mL) and water (7 mL) was added TEMPO (41 mg, 0.26 mmol, 0.20 equiv.) and BAIB (638 mg, 1.98 mmol, 1.5 equiv.). The biphasic solution was stirred for 7 h at rt, and then quenched with saturated aq. $Na_2SO_3$ (20 mL). It was then extracted with $Et_2O$ (3 × 20 mL). The combined organic layers were dried over $MgSO_4$, and concentrated. The resuling crude aldehyde was carried forward without further purification.

To a solution of Ohira−Bestmann reagent **24** (760 mg, 3.96 mmol, 3.0 equiv.) in dry THF (12 mL) at –78 °C was added NaOMe (25% solution in MeOH, 5.1 mL, 3.96 mmol) dropwise. The mixture was stirred for 20 min, then a solution of the above crude aldehyde in dry THF (12 mL) was added dropwise. The reaction was allowed to slowly warm to rt over 30 min, and was then quenched with saturated aq. $NH_4Cl$ (25 mL). The mixture was diluted with $H_2O$ (20 mL) and extracted with $Et_2O$ (3 × 50 mL). The combined organic extracts were washed with brine (20 mL), dried over $MgSO_4$, and concentrated to afford the crude product as a yellow oil. Purification by flash chromatography (9:1 petroleum ether / EtOAc) afforded **S12** as a white solid (233 mg, 0.752 mmol, 57%).

$\mathbf{R_f}$ 0.31 (4:1 petroleum ether / $Et_2O$); **IR** (thin film) $\nu_{max}/cm^{-1}$ 3291, 2932, 1613, 1513, 1246, 1174, 1099, 1075, 988, 820, 757; **$^1$H NMR** (500 MHz, $CDCl_3$) $\delta_H$ 7.28 (2H, d, *J* = 8.6 Hz, ArH), 6.88 (2H, d, *J* = 8.6 Hz, ArH), 4.76 (0.5H, d, *J* = 4.5 Hz, H1), 4.57 (0.5H, d, *J* = 4.0 Hz, H1), 4.50-4.43 (2H, m, H7), 4.37–4.26 (1H, m, H4), 3.80 (3H, s, H8), 3.67–3.58 (2H, m, H6), 3.36 (1.5H, s, H12), 3.31 (1.5H, s, H12), 2.88 (0.5H, ddd, *J* = 10.8, 8.4, 2.6 Hz, H3), 2.73 (0.5H, ddd, *J* = 8.4, 7.2, 2.5 Hz, H3), 2.36–2.29 (0.5H, m, H2), 2.24-2.16 (0.5H, m, H2), 2.15 (0.5H, d, *J* = 2.5 Hz, H10), 2.13 (0.5H, d, *J* = 2.5 Hz, H10), 2.12–2.04 (1H, m, H5), 1.94 (0.5H, ddt, *J* = 14.1, 10.6, 5.4 Hz, H5), 1.82 (0.5H, dddd, *J* = 13.9, 9.5, 6.6, 5.3 Hz, H5), 1.17 (1.5H, d, *J* = 7.0 Hz, H11), 1.11 (1.5H, d, *J* = 6.9 Hz, H11); **$^{13}$C NMR** (126 MHz, $CDCl_3$) $\delta_C$ 159.2, 159.2, 130.8, 130.8, 129.4, 129.4, 113.9, 113.9, 111.9, 105.0, 82.9, 82.1, 77.3, 75.3, 72.8, 72.8, 72.1, 72.0, 67.3, 67.2, 56.0, 55.4, 55.4, 54.8, 46.6, 45.2, 41.2, 39.0, 33.8, 33.4, 16.3, 11.8; **HRMS** (ESI$^+$) calc. for $C_{18}H_{24}O_4Na$ $[M+Na]^+$ 327.1567; found 327.1566.

**(((2*S*,3*R*,4*S*)-5-Methoxy-2-(2-((4-methoxybenzyl)oxy)ethyl)-4-methyltetrahydrofuran-3-yl)ethynyl)trimethylsilane, S13**

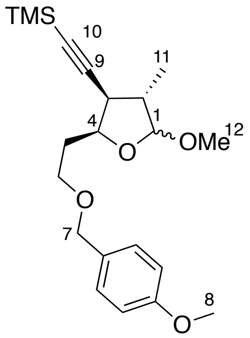

To a stirred solution of terminal alkyne **S12** (170 mg, 0.559 mmol, 1.0 equiv.) in dry THF (12 mL) under argon at –78 °C was slowly added *n*-BuLi (2.5 M in THF, 0.67 mL, 1.68 mmol, 3.0 equiv.). The mixture was stirred for 30 min at –78 °C, then chlorotrimethylsilane (0.14 ml, 1.12 mmol, 2.0 equiv.) was added. The mixture stirred for 60 min at –78 °C, and then the reaction was quenched by addition of saturated aq. $NH_4Cl$ (20 mL). $Et_2O$ was added, and the aqueous layer was extracted with diethyl ether (3 x 50 mL). The combined organic layers were dried with $MgSO_4$, and concentrated. The crude product was purified by flash chromatography (19:1 petroleum ether / $Et_2O$) to give **S13** (193 mg, 0.551 mmol, 92%) as a light yellow oil.

**R$_f$** 0.36 (4:1 petroleum ether / $Et_2O$); **IR** (thin film) $\nu_{max}$/cm$^{-1}$ 2957, 2862, 2175, 1613, 1513, 1247, 1173, 1099, 1073, 998, 841, 759; **$^1$H NMR** (500 MHz, $CDCl_3$) $\delta_H$ 7.28 (2H, d, *J* = 8.6 Hz, ArH), 6.88 (2H, d, *J* = 8.6 Hz, ArH), 4.74 (1H, d, *J* = 4.5 Hz, H1), 4.46 (2H, s, H7), 4.29 (1H, td, *J* = 8.8, 4.2 Hz, H4), 3.80 (3H, s, H8), 3.68–3.55 (2H, m, H6), 3.33 (3H, s, H12), 2.90 (1H, dd, *J* = 10.6, 8.5 Hz, H3), 2.23–2.11 (1H, m, H5), 2.07 (1H, m, H5,), 1.80 (1H, m, H2), 1.10 (3H, d, *J* = 6.9 Hz, H11), 0.14 (9H, s, TMS); **$^{13}$C NMR** (126 MHz, $CDCl_3$) $\delta_C$ 159.2, 130.8, 129.3, 113.9, 88.4, 88.3, 77.5, 77.4, 77.2, 76.9, 75.4, 72.8, 55.4, 54.7, 45.4, 40.0, 33.7, 16.1, 11.9, 0.0; **HRMS** (ESI$^+$) calc. for $C_{21}H_{32}O_4NaSi$ [M+Na]$^+$ 399.1962; found 399.1956.

**(((2*S*,3*R*,4*S*)-5-Methoxy-4-methyl-2-(prop-2-yn-1-yl)tetrahydrofuran-3-yl)ethynyl)trimethylsilane, 25**

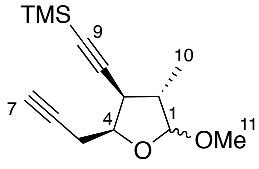

To a solution of **S13** (190 mg, 0.505 mmol, 1.0 equiv.) in $CH_2Cl_2$ (8.5 mL) at rt was added $H_2O$ (3 mL), followed by DDQ (172 mg, 0.758 mmol, 1.5 equiv.). The reaction mixture was stirred for 35 min (monitored by TLC). The reaction was then quenched with saturated aq. $NaHCO_3$ (50 mL) and was stirred for 30 min to dissolve all solids. The layers were separated, and the aqueous layer was extracted with dichloromethane (3 × 20 mL), and the combined organic layers were dried over $Na_2SO_4$ and concentrated. The residue was redissolved in $CH_2Cl_2$ (3 mL) and water (3 mL) and stirred at rt. TEMPO (16 mg, 0.10 mmol, 0.2 equiv.) and BAIB (244 mg, 0.758 mmol, 1.5 equiv.) were added sequentially, and the reaction was stirred at rt for 3.5 h, after which time TLC indicated complete consumption of the alcohol. The reaction was quenched with MeOH (1 mL) and stirred for 30 min. Water (5 mL) and $CH_2Cl_2$ (5 mL) were added, the layers were separated, and the aqueous layer was extracted with dichloromethane (3 × 20 mL). The combined organic layers were dried

over $Na_2SO_4$ and concentrated. The residue was azeotroped twice with 5 mL dry toluene, and then stored under Ar.

To a solution of $PPh_3$ (662 mg, 2.53 mmol, 5.0 equiv.) in $CH_2Cl_2$ (7 mL) at 0 °C was added $CBr_4$ (419 mg, 1.26 mmol, 2.5 equiv.). The mixture was stirred at 0 °C for 15 min, then cooled to −30 °C. To this, a solution of the crude aldehyde and $Et_3N$ (0.70 mL, 5.05 mmol, 10 equiv.) in $CH_2Cl_2$ (3 mL) was added dropwise, and the reaction mixture was allowed to warm to 0 °C over 1h. The reaction was quenched with saturated aq. $NH_4Cl$ (20 mL), the layers were separated, and the aqueous layer extracted with $CH_2Cl_2$ (3 x 15 mL). The combined organic layers were dried over $Na_2SO_4$, concentrated, and the residue was purified via flash chromatography (49:1 petroleum ether / $Et_2O$) to give the crude dibromoalkene (which co-eluted with 1-(2,2-dibromovinyl)-4-methoxybenzene from the PMB deprotection).

This mixture was transferred to a flame-dried flask as a solution in THF (10 mL). The solution was cooled to −78 °C, then LiHMDS (2.05 mL, 2.05 mmol, 1 M in THF, 4.0 equiv.) was added. The resulting solution was warmed slowly to −15 °C over 2 h, then cooled again to −78 °C. *n*-BuLi (0.61 mL, 1.51 mmol, 2.5 M in hexanes, 3.0 equiv.) was added dropwise along the walls of the vial, and stirred for 5 min. The reaction was monitored by the TLC and on completion, a solution of saturated aq. $NH_4Cl$ (10 mL) was added at −78 °C. The mixture was warmed to rt, and extracted with $Et_2O$ (3 x 25 mL). The combined organic layers were dried over $Na_2SO_4$, and concentrated. The residue was purified via flash chromatography (49:1 petroleum ether / $Et_2O$) to yield diyne **25** (82 mg, 0.33 mmol, 65%) as a colourless oil.

**$R_f$** 0.34 (19:1 petroleum ether / $Et_2O$); **IR** (thin film) $\nu_{max}/cm^{-1}$ 3313, 2956, 2175, 1613, 1513, 1249, 1104, 1099, 1016, 842, 760; **$^1$H NMR** (400 MHz, $CDCl_3$) $\delta_H$ 4.65–4.56 (1H, m, H1), 4.31 (1H, app q, *J* = 7.4 Hz, H4), 3.42 (3H, s, H11), 2.77 (1H, t, *J* = 8.1 Hz, H3), 2.69–2.53 (2H, m, H5), 2.29 (1H, m, H2), 2.02 (1H, br s, H7), 1.19 (3H, d, *J* = 7.2 Hz, H10), 0.0 (9H, s, TMS); **$^{13}$C NMR** (101 MHz, $CDCl_3$) δ 111.6, 103.4, 89.0, 82.2, 79.2, 69.5, 56.0, 46.7, 42.3, 23.8, 16.4, 0.1; **HRMS** ($ESI^+$) calc. for $C_{14}H_{22}O_2SiNa$ $[M+Na]^{+}$: 273.1281; found 273.1281.

**(3*S*,4*R*,5*S*)-3-Methyl-5-(prop-2-yn-1-yl)-4-((trimethylsilyl)ethynyl)tetrahydrofuran-2-yl acetate: S14**

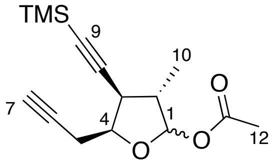


To a solution of methyl acetal **25** (147 mg, 0.588 mmol, 1.0 equiv.) in THF (4 mL) was added acetic acid (4 mL) and the mixture was cooled to 0 °C. Conc. sulfuric acid (20 µL, 0.33 mmol, 0.5 equiv.) was added slowly to the reaction, followed by $Ac_2O$ (0.32 mL, 3.53 mmol, 6.0 equiv.), and the mixture was stirred at 0 °C for 2 h until TLC indicated the consumption of starting material. The reaction was quenched with saturated aq. $NaHCO_3$ (30 mL), poured into $Et_2O$ (20 mL), and the layers were separated. The aqueous layer was extracted with $Et_2O$ (3 × 20 mL), then the organic layers were combined and dried over $Na_2SO_4$. The mixture was concentrated, and the residue was purified by flash chromatography (4:1 pentane / $Et_2O$) to give a 3.3:1 mixture of acetates **S14** (148 mg, 0.535 mmol, 91%) as an oil.

**R$_f$** 0.30 (4:1 petroleum ether / $Et_2O$); **IR** (thin film) $\nu_{max}$/cm$^{-1}$ 2961, 2173, 1753, 1458, 1330, 1230, 1111, 1088, 1007, 958, 899, 842; **$^1$H NMR** (400 MHz, $CDCl_3$) $\delta_H$ 6.29 (1H, d, $J$ = 4.4 Hz, H1), 5.85 (1H, d, $J$ = 2.5 Hz, H1*), 4.45 (1H, ddd, $J$ = 8.4, 7.0, 4.7 Hz, H4), 4.39 (1H, m, H4*), 3.00 (1H, dd, $J$ = 11.5, 8.6 Hz, H3), 2.87 (2H, dd, $J$ = 7.0, 5.8 Hz, H5*), 2.68–2.59 (1H, m, H5), 2.68–2.59 (1H, m, H5*) 2.56–2.40 (2H, m, H2 and H5), 2.05 (3H, s, H12*), 2.05 (3H, s, H12), 2.03–1.94 (1H, m, H7), 1.18 (3H, d, $J$ = 7.2 Hz, H10*), 1.09 (3H, d, $J$ = 6.8 Hz, H10), 0.16 (9H, s, $SiMe_3$); **$^{13}$C NMR** (101 MHz, $CDCl_3$) $\delta_C$ 170.4*, 170.1, 103.8, 102.9*, 102.6, 98.6, 98.6*, 89.8, 89.5*, 81.0, 80.9*, 80.2*, 78.7, 70.2, 69.9*, 46.8*, 44.2, 41.2*, 39.3, 23.5, 23.5*, 21.4*, 21.2, 16.2*, 11.5, 0.0*, -0.0; **HRMS** (ESI$^+$) calc. for $C_{15}H_{22}O_3SiNa$ $[M+Na]^+$ 301.1230; found 301.1230. *= peaks corresponding to minor diastereomer.

**(3*S*,4*R*,5*S*)-5-(3-Iodoprop-2-yn-1-yl)-3-methyl-4-((trimethylsilyl)ethynyl)tetrahydrofuran-2-yl acetate, 26**

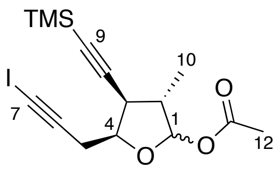


To a solution of $I_2$ (2.73 g, 10.7 mmol, 11 equiv.) in toluene (30 mL) under an Ar atmosphere at 0 °C was added morpholine (1.9 mL, 21.5 mmol, 22 equiv.) dropwise. The mixture was sonicated to remove solids from the sides of the flask, then heated to 45 °C for 30 min. A solution of diyne **S14** (270 mg, 0.970 mmol, 1.0 equiv.) in toluene (5 mL) was then added dropwise at 45 °C, and the reaction mixture was stirred at 45 °C for 16 h. The mixture was then allowed to cool to room temperature and was filtered, washing the filtered solids with toluene. The filtrate was washed with saturated aq. $Na_2SO_3$, brine, and then dried over $Na_2SO_4$ and concentrated. The crude product was purified by flash chromatography (4:1 pentane / $Et_2O$) to give **26** (378 mg, 0.936 mmol, 97%) as an oil.

**R$_f$** 0.30 (4:1 petroleum ether / $Et_2O$); **IR** (thin film) $\nu_{max}$/cm$^{-1}$: 2961, 2171, 1737, 1457, 1420, 1372, 1248, 1111, 1054, 977, 841; **$^1$H NMR** (500 MHz, $CDCl_3$) $\delta_H$ 6.26 (1H, d, $J$ = 4.5 Hz, H1), 5.84 (1H, d, $J$ = 2.7 Hz, H1*), 4.43 (1H, ddd, $J$ = 8.5, 6.2, 5.0 Hz, H4), 4.38 (1H, q, $J$ = 6.9 Hz, H4*), 3.00 (1H, dd, $J$ = 11.5, 8.6 Hz, H3), 2.88–2.81 (3H, m, H3* and H5*), 2.80 (1H, dd, $J$ = 17.0, 5.1 Hz, H5), 2.63 (1H, dd, $J$ = 17.0, 6.3 Hz, H5), 2.57–2.52 (1H, m, H2*), 2.48–2.44 (1H, m, H2), 2.06 (3H, s, H12*), 2.05 (3H, s, H12), 1.17 (3H, d, $J$ = 7.2 Hz, H10*), 1.10 (3H, d, $J$ = 6.8 Hz, H10) 0.17 (9H, s, ($SiMe_3$)), 0.16 (9H, s, ($SiMe_3$)*); **$^{13}$C NMR** (126 MHz, $CDCl_3$) $\delta_C$ 170.4*, 170.1, 103.7, 102.7*, 102.5, 98.6, 98.6*, 90.8*, 90.8, 89.9, 89.5*, 80.0*, 78.6, 46.6*, 44.1, 41.1*, 39.2, 25.8*, 25.7, 21.4*, 21.1, 16.1*, 11.5, 0.0, 0.0*, -4.5, -4.7*; **HRMS** (ESI$^+$) calc. for $C_{15}H_{21}O_3{}^{127}ISiNa$ $[M+Na]^+$427.0197; found 427.0202. *= peaks corresponding to minor diastereomer.

**5-((3*S*,4*R*,5*S*)-5-(3-Iodoprop-2-yn-1-yl)-3-methyl-4-((trimethylsilyl)ethynyl)tetrahydrofuran-2-yl)-3-methylfuran-2(5*H*)-one, 8a-d**

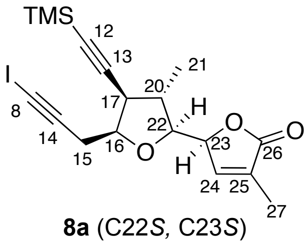


**8a** (C22*S*, C23*S*)

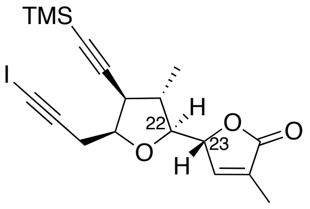


**8b** (C22*S*, C23*R*)

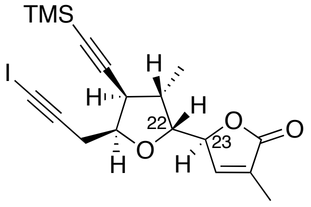


**8c** (C22*R*, C23*S*)

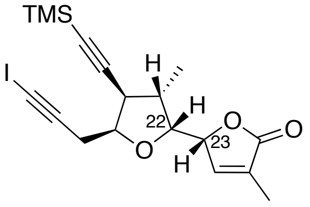


**8d** (C22*R*, C23*R*)

To a solution of Bi(OTf)$_3$ (1.23 g, 1.87 mmol, 2.0 equiv.) in acetonitrile (16 mL) under an Ar atmosphere at –40 °C was added dropwise a solution of acetate **26** (378 mg, 0.936 mmol, 1.0 equiv.) and triisopropyl((3-methylfuran-2-yl)oxy)silane **18** (0.401 mL, 1.40 mmol, 1.5 equiv.) in acetonitrile (4 mL). The reaction mixture was stirred at –40 °C for 1 h until TLC showed the reaction to be complete. The reaction was quenched with saturated aq. $NH_4Cl$ (10 mL) and allowed to warm to rt. The mixture was poured into $CH_2Cl_2$ and the layers were separated. The aqueous layer was extracted with $CH_2Cl_2$ (3 x 100 mL), the organic layers were combined, washed with water and then brine, and dried over $Na_2SO_4$. After concentration, purification of the residue by flash chromatography (19:1 pentane / $Et_2O$) gave **8c** (81 mg), **8b** (65 mg), and **8a/8d** (121 mg, inseparable mixture of diastereomers, also contaminated with the furanone from hydrolysis of **18**).

**8c: R$_f$** 0.43 (3:1 pentane / $Et_2O$); **mp** = 113-115 °C; $[\alpha]_D^{25}$ –121 (c = 1.01, $CHCl_3$); **IR** (thin film) $\nu_{max}$/cm$^{-1}$ 2960, 2171, 1761, 1249, 1094, 1059, 1007, 843, 760; **$^1$H NMR** (500 MHz, $CDCl_3$) δ 7.30-7.24 (m, 1H, H24), 4.70 (1H, dt, *J* = 9.3, 1.9 Hz, H23), 4.28 (1H, q, *J* = 6.5 Hz, H16), 3.81 (1H, dd, *J* = 9.3, 5.8 Hz, H22), 2.88 (1H, dd, *J* = 6.2, 4.4 Hz, H17), 2.76–2.68 (2H, m, H15), 2.68–2.61 (1H, m, H20), 1.92 (3H, t, *J* = 1.7 Hz, H27), 1.19 (3H, d, *J* = 7.2 Hz, H21), 0.13 (9H, s, TMS); **$^{13}$C NMR** (126 MHz, $CDCl_3$) δ 174.0, 149.0, 130.2, 125.5, 102.8, 90.9, 90.5, 82.0, 78.6, 77.9, 43.1, 42.6, 25.2, 13.7, 10.7, 0.0; **HRMS** (ESI$^+$) calc. for $C_{18}H_{23}O_3{}^{127}ISiNa$ $[M+Na]^+$465.0353; found 465.0357.

**8b: R$_f$** 0.32 (3:1 pentane / $Et_2O$); $[\alpha]_D^{25}$ –14 (c = 0.63, $CHCl_3$); **IR** (thin film) $\nu_{max}$/cm$^{-1}$ 2960, 2926, 2357, 2173, 1761, 1657, 1454, 1317, 1249, 1207, 1091, 1071, 1039, 951, 843, 759, 700, 668; **$^1$H NMR** (400 MHz, $CDCl_3$) δ 7.24 (1H, m, H24), 4.86 (1H, m, H23), 4.18 (1H, td, *J* = 6.8, 5.4 Hz, H16), 3.31 (1H, t, *J* = 7.2 Hz, H22), 2.84–2.63 (3H, m, H15/H17), 2.45 (1H, m, H20), 1.94 (3H, t, *J* = 1.8 Hz, H27), 1.19 (3H, d, *J* = 6.8 Hz, H21), 0.16 (9H, s, TMS); **$^{13}$C NMR** (101 MHz, $CDCl_3$) $\delta_C$ 174.0, 147.6, 130.5, 103.1, 91.0, 90.1, 86.2, 82.5, 78.1, 77.2, 44.3, 43.1, 25.6, 17.2, 10.7, 0.0; **HRMS** (ESI$^+$) calc. for $C_{18}H_{23}O_3{}^{127}ISiNa$ $[M+Na]^+$465.0353; found 465.0356.

**8a/d: R$_f$** 0.19 (3:1 pentane / $Et_2O$); **IR** (thin film) $\nu_{max}$/cm$^{-1}$ 2959, 2926, 2167, 1759, 1456, 1250, 1051, 989, 944, 843, 760, 699; **$^1$H NMR** (500 MHz, $CDCl_3$) $\delta_H$ 7.06–7.02 (1H, m, H24(**8a**)), 6.97–6.94 (1H, m, H24(**8d**)), 4.94 (1H, dt, *J* = 3.5, 1.9 Hz, H23(**8a**)), 4.86 (1H, q, *J* = 1.9 Hz, H23(**8d**)), 4.73 (1H, m, furanone impurity),

4.25 (1H, dd, $J$ = 7.8, 1.7 Hz, H22(**8d**)), 4.19 (1H, dt, $J$ = 7.5, 5.6 Hz, H16(**8d**)), 4.14 (1H, td, $J$ = 7.6, 5.0 Hz, H16(**8a**)), 3.71 (1H, dd, $J$ = 8.8, 3.1 Hz, H22(**8a**)), 3.15 (1H, dd, $J$ = 10.6, 8.1 Hz, H17(**8d**)), 2.77 (1H, dd, $J$ = 9.6, 7.4 Hz, H17(**8a**)), 2.74–2.51 (5H, m, H15/H20(**8d**); H15(**8a**)), 2.27 (1H, dt, $J$ = 13.7, 6.9 Hz, H20(**8a**)), 1.96 (3H, t, $J$ = 1.7 Hz, H27(**8a**)), 1.93−1.90 (5H, m, H27(**8d**); furanone), 1.26 (3H, d, $J$ = 7.0 Hz, 3H, H21(**8d**)), 1.13 (3H, d, $J$ = 6.7 Hz, 3H, H21(**8a**)), 0.13 (18H, s, TMS(**8a**/**8d**)); **$^{13}$C NMR** (126 MHz, $CDCl_3$) $\delta_C$ 174.1, 173.7, 146.3, 145.6, 131.3, 130.9, 103.4, 102.5, 91.3, 91.1, 89.8, 89.3, 84.0, 80.7, 79.6, 79.4, 78.9, 78.0, 77.4, 77.1, 76.8, 43.0, 42.2, 41.2, 40.7, 26.0, 25.6, 15.9, 13.1, 11.0, 10.8, 0.1, 0.0 ppm; **HRMS** (ESI$^+$) calc. for $C_{18}H_{23}O_3{}^{127}ISiNa$ $[M+Na]^+$465.0353; found 465.0357.

**Stereochemical assignment of Diynes (8a-8d)**

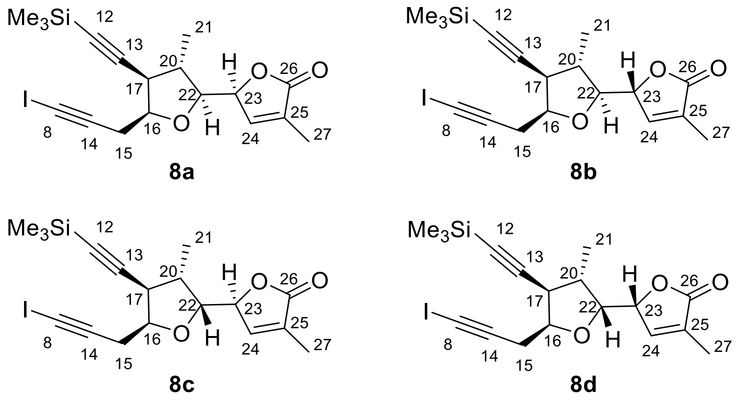


To assign the stereochemistry of the four diastereomers produced during the connection of the F and G rings, a combination of nOe enhancements and coupling constant analysis was used. In adduct **8b**, the C22 stereocentre was assigned as *S*, with this proton on the bottom face of the ring (as drawn) due to strong nOe enhancements with the protons on the C21 methyl and at C17. The C23 stereocentre was assigned as *R* (proton on the top face as drawn) due to a combination of a strong enhancement of the C23 proton with the proton at C20, an enhancement between H24 and H22, and a coupling constant $J$(H22–H23) of 7.5 Hz. These observations would be explained by a dominant conformation similar to that in **Figure S1b**, in which the H23 and H22 protons are antiperiplanar.

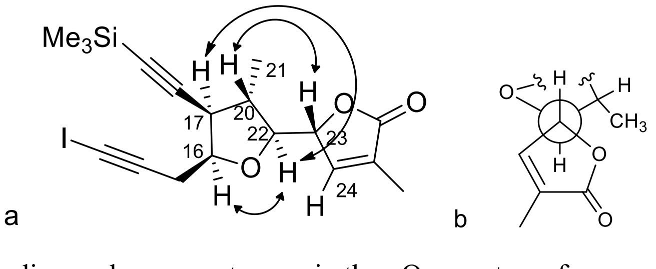


**Figure S1.** a. Strong and medium enhancements seen in the nOe spectrum for compound **8b**; b. Conformation of butenolide ring in compound **8b**.

The C22 stereocentre of adduct **8c** was assigned as *R* due to the lack of corresponding strong nOe enhancements of H22 with the protons at C16 and C17 on the bottom face (see above, and discussion on **8a** below). Assignment of the C23 stereocentre as *S* (bottom face as drawn) was based on strong enhancements of H23

with the C21 methyl protons in combination with the coupling constant for *J*(H22–H23) of 9.3 Hz, again indicating an antiperiplanar orientation. These observations would be explained by the conformation shown in **Figure S2**. The configuration of **8c** was also confirmed by x-ray crystallographic analysis. (See P S48 for details)

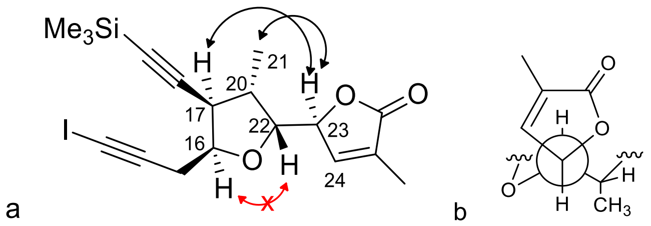


**Figure S2.** a. Strong enhancements seen in the nOe spectrum for compound **8c**; b. Conformation of butenolide ring in compound **8c**.

Compounds **8a** and **8d** were obtained as an inseparable mixture. After assignments of all peaks by $^{1}$H-$^{1}$H COSY, cross peaks in the $^{1}$H-$^{1}$H NOESY spectrum were used to provide stereochemical information on these final two diastereomers. The proton at C22 in one diastereomer displayed strong enhancements with H17 and the methyl group C21, indicating that it was oriented on the bottom face, which matches the proposed C22*S* stereochemistry of the natural product. This diastereomer was named **8a**. The proton at C23 displayed a medium strength enhancement with the proton at C20 and the methyl group at C21. A coupling constant for H22-H23 of ~3 Hz, and the through-space enhancement seen between H22 and H24 in the butenolide ring, would all be explained by an *S* configuration at C23, and the conformation shown in **Figure S3**.

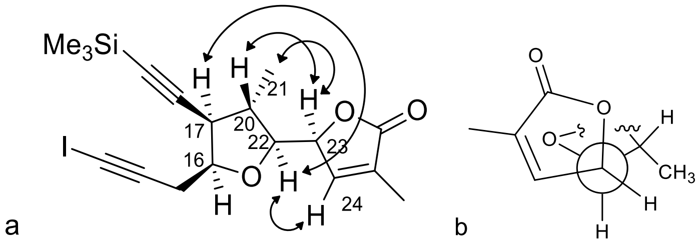


**Figure S3.** a. Enhancements seen in the nOe spectrum for compound **8a**; b. Conformation of butenolide ring in compound **8a**.

The final diastereomer (**8d**) displayed a lack of strong enhancements between the H22 and either the H16 or H17 signals, which indicated an *R* configuration (when compared with other diastereomers), with the proton at C22 being on the top face of the F ring. The proton at C23 showed a strong enhancement with the methyl group at C21, which combined with a coupling value of 1.9 Hz, suggested the 23*R* stereochemistry in which the butenolide adopts a conformational rotation placing the H22 and H23 close to a 90° angle, as shown in **Figure S4**.

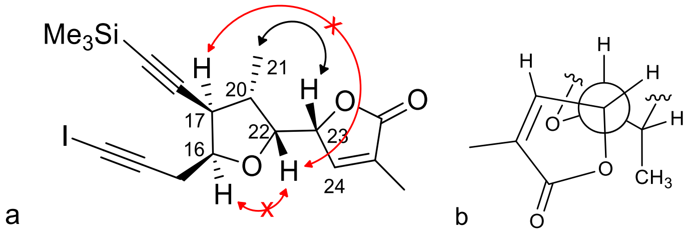


**Figure S4.** a. Enhancements seen in the nOe spectrum for compound **8d**; b. Conformation of butenolide ring in compound **8d**.

**(2*R*,3*S*,4*S*,5*R*)-5-(4-Hydroxydeca-2,9-diyn-1-yl)-3-methyl-4-((trimethylsilyl)ethynyl)tetrahydrofuran-2-yl acetate, 27**

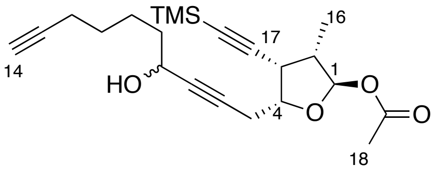


To a solution of iodoalkyne **16a** (20 mg, 0.070 mmol, 1.5 equiv.) in THF (0.3 mL) at –78 °C was added *n*-BuLi (24 μL, 2.5 M in hexanes, 0.061 mmol, 1.3 equiv.) dropwise. The solution was stirred for 45 min, then a solution of hept-6-ynal (5.0 mg, 0.047 mmol, 1.0 equiv.) in THF (0.2 mL) was added. The solution was slowly warmed to –40 °C and was stirred for 4 h. The reaction was quenched with saturated aq. $NH_4Cl$ (5 mL). The aqueous layer was extracted using EtOAc (4 x 5 mL). The combined organic layers were dried using $Na_2SO_4$ and concentrated, and the residue was purified via flash chromatography (5:1 petroleum ether / EtOAc to yield **27** (6.0 mg, 0.025 mmol, 35%) as a viscous oil.

Alternatively, to a solution of hept-6-ynal (10.0 mg, 0.053 mmol, 1.3 equiv.) and iodoalkyne **17a** (17 mg, 0.041 mmol, 1.0 equiv.) in THF (2 mL) was added $CrCl_2$ (97%, 30.0 mg, 0.245 mmol, 6.0 equiv., addition in the glovebox), and the solution was stirred at rt for 15 h. The reaction was quenched with 1 M serine solution (15 mL). The aqueous layer was extracted using EtOAc (4 x 10 mL), the combined organic layers were dried using $Na_2SO_4$, and concentrated. The residue was purified via flash column chromatography (10:3 petroleum ether / EtOAc) to yield **27** (12 mg, 0.039 mmol, 73%) as a viscous oil.

**R$_f$** 0.51 (1:1 petroleum ether / EtOAc); **IR** (thin film) $\nu_{max}$/cm$^{-1}$ 3459, 3311, 2930, 2171, 1740, 1461, 1374 1187, 1162, 1087, 966; **$^1$H NMR** (400 MHz, $CDCl_3$) $\delta_H$ 5.92 (1H, d, *J* = 4.1 Hz, H1), 4.35 (1H, m, H8), 4.31 (1H, tdd, *J* = 7.1, 5.7, 1.1 Hz, H4), 3.31 (1H, dd, *J* = 7.3, 5.7 Hz, H3), 2.72–2.62 (2H, m, H5), 2.52–2.42 (1H, m, H2), 2.23–2.17 (2H, m, H12), 2.06 (3H, s, H19), 1.94 (1H, t, *J* = 2.7 Hz, H14), 1.81 (1H, br s, OH), 1.72–1.63 (2H, m, H9), 1.61–1.51 (4H, m, H10, H11), 1.21 (3H, d, *J* = 7.1 Hz, H16), 0.16 (9H, s, ($SiMe_3$)); **$^{13}$C NMR** (101 MHz, $CDCl_3$) $\delta_C$ 170.2, 125.5, 101.0, 98.7, 91.4, 8.3, 82.5, 81.6, 80.8, 68.3, 62.4, 41.3, 4.4, 37.3, 30.3, 28.1, 25.9, 25.8, 24.3, 24.3, 21.1, 18.3, 15.2, 14.0, 10.2, -0.00; **HRMS** (ESI$^+$) calc. for $C_{22}H_{32}O_4SiNa$ $[M+Na]^+$ 411.1962; found 411.1961.

**(7b*S*,8*S*,9*R*,10a*R*)-1-Hydroxy-8-methyl-1,2,3,4,5,7b,8,9,10a,11-decahydrocyclohepta[4,5]indeno-[2,1-b]furan-9-yl acetate, 28**

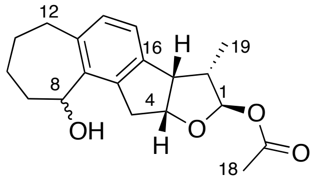

To a solution of **27** (15.0 mg, 0.039 mmol, 1.0 equiv.) in THF (1.0 mL) at rt was added AcOH (2.3 μL, 0.039 mmol, 1.0 equiv.), and then TBAF (78 μL, 0.078 mmol, 1 M in THF, 2.0 equiv.). The reaction was stirred for 15 min, after which time starting material had been consumed. The reaction was then quenched by addition of saturated aq. $NH_4Cl$ (10 mL). The aqueous layer was extracted using EtOAc (4 x 10 mL). The combined organic layers were dried using $Na_2SO_4$, and concentrated. The crude product was used directly in the next step without further purification.

To a stirred solution of $RhCl(PPh_3)_3$ (7.2 mg, 7.8 μmol, 0.2 equiv.) in DCE (5 mL) was added a solution of the above crude triyne in DCE (6 mL). The reaction was stirred at 50 °C for 12 h, then cooled to rt and concentrated. The residue was purified via flash chromatography (2:1 petroleum ether / $Et_2O$) to yield **28** (9.0 mg, 0.028 mml, 73% over two steps) as a white solid (inconsequential mixture of C8 diastereomers).

**R$_f$** 0.29 and 0.36 (2:3 petroleum ether / $Et_2O$); **IR** (thin film) $\nu_{max}/cm^{-1}$ 3471, 2926, 1740, 1456, 1378, 1121, 1084, 1009, 972, 808; **$^1$H NMR** (400 MHz, $CDCl_3$) $\delta_H$ 6.96 (2H, dd, *J* = 5.9, 2.3 Hz, H14 and H15), 5.91 (1H, dd, *J* = 10.8, 1.9 Hz, H1), 5.18–5.06 (2H, m, H4 and H8), 3.98 (1H, ddd, *J* = 10.0, 7.7, 2.7 Hz, H3), 3.56–3.21 (1H, m, H5), 3.22–3.09 (2H, m, H12), 2.78–2.59 (2H, m, H2 and H5), 2.33–2.10 (2H, m, H9, H10 or H11), 2.09 (3H, s, H18), 2.01–1.87 (1H, m, H9, H10 or H11), 1.83–1.54 (4H, m, OH and H9, H10 or H11), 0.90 (3H, 2 x s, H18); **$^{13}$C NMR** (101 MHz, $CDCl_3$) $\delta_C$ 170.7, 142.5, 142.0, 140.9, 139.2, 139.0, 138.2, 138.1, 129.4, 129.4, 125.5, 125.4, 105.7, 105.6, 848, 84.7, 71.4, 71.0, 51.5, 51.4, 42.6, 42.6, 38.6, 38.4, 35.5, 35.5, 33.2, 33.0, 30.3, 29.7, 28.7, 28.3, 24.9, 24.8, 21.4, 14.5, 14.5; **HRMS** (ESI$^+$) calc. for $C_{19}H_{24}O_4Na$ $[M+Na]^+$ 339.1567; found 339.1567.

**(7b*S*,8*S*,9*R*,10a*R*)-8-Methyl-3,4,5,7b,8,9,10a,11-octahydrocyclohepta[4,5]indeno[2,1-b]furan-9-yl acetate, 29**

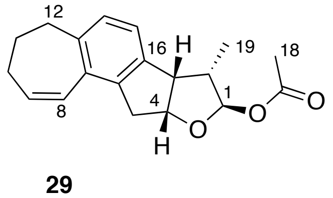

**29**

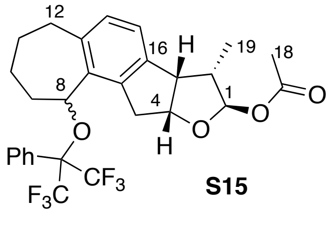

**S15**

To a flame dried vial in the glovebox was added Martin sulfurane (33 mg, 0.049 mmol, 1.5 equiv.). To it was added a solution of alcohol **28** (10 mg, 0.033 mmol, 1.0 equiv.) in $CH_2Cl_2$ (2 mL). The reaction was stirred at rt for 10 min, then quenched by addition of saturated aq. $NaHCO_3$ (5 mL). After stirring for 10 min, the layers were separated, and the aqueous layer was extracted with $CH_2Cl_2$ (4 x 10 mL). The combined organic layers

were dried using $Na_2SO_4$, and concentrated. The residue was purified via flash chromatography (9:1 petroleum ether / $Et_2O$ to afford an inseparable 7:1 mixture of **29** and **S15** (8.3 mg, 0.041 mmol, 83%) as a viscous oil.
**R**$_f$ 0.20 (4:1 petroleum ether / $Et_2O$); **IR** (thin film) $\nu_{max}$/cm$^{-1}$: 2981, 2927, 1745, 1461, 1382, 1260, 1151, 1185, 1033, 955; **$^1$H NMR** (400 MHz, $CDCl_3$) $\delta_H$ 6.95 (1H, d, *J*= 8.0 Hz, H14), 6.94 (1H, d, *J*= 8.0 Hz, H15), 6.36 (1H, td, H8), 5.99 (1H, dt, *J* = 12.2, 4.7 Hz, H9), 5.94 (1H, d, *J* = 1.6 Hz, H1), 5.17 (1H, td, *J* = 7.4, 2.8 Hz, H4), 4.02 (1H, t, *J* = 8.1 Hz, H3), 3.20 (1H, dd, *J* = 17.6, 7.4 Hz, H5), 3.06 (1H, dd, *J* = 17.6, 2.8 Hz, H5), 2.77 (2H, t, *J* = 5.5 Hz, H12), 2.70–2.62 (1H, m, H2), 2.48–2.29 (2H, m, H10), 2.09 (3H, s, H18), 2.02–1.90 (2H, m, H11), 0.86 (3H, d, *J* = 7.5 Hz, H19); **$^{13}$C NMR** (101 MHz, $CDCl_3$) $\delta_C$ 170.6, 141.8, 141.1, 137.7, 133.2, 132.9, 127.7, 126.3, 124.3, 105.9, 84.9, 51.5, 42.4, 38.7, 35.3, 31.9, 28.5, 21.4, 14.5; **HRMS** (ESI$^+$) calc. for $C_{19}H_{22}O_3Na$ [M+Na]$^+$ 321.1461; found 321.1461.

**(*S*)-3-Methyl-5-((7b*S*,8*S*,9*S*,10a*R*)-8-methyl-3,4,5,7b,8,9,10a,11-octahydrocyclohepta[4,5]indeno[2,1-b]furan-9-yl)furan-2(5*H*)-one, 30**

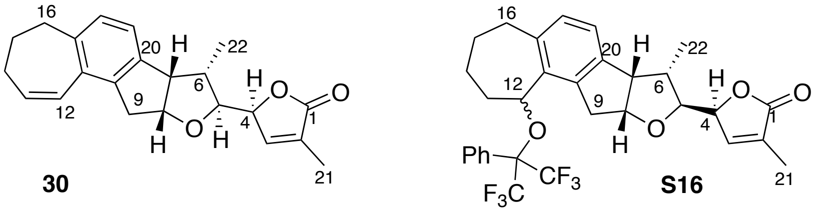


To a solution of $Bi(OTf)_3$ (62 mg, 0.094 mmol, 2.0 equiv.) in acetonitrile (0.2 mL) under an Ar atmosphere at –40 °C was added dropwise a solution of acetate **29** (14 mg, 0.047 mmol, 1.0 equiv.) and triisopropyl((3-methylfuran-2-yl)oxy)silane (18 mg, 0.071 mmol, 1.5 equiv.) in acetonitrile (1 mL). The reaction was stirred at –40 °C for 1 h until complete as judged by TLC. The reaction was quenched by addition of saturated aq. $NH_4Cl$ (5 mL), and allowed to warm to rt. The mixture was poured into $CH_2Cl_2$ (10 mL) and the layers were separated. The aqueous layer was extracted with $CH_2Cl_2$ (3 x 5 mL) and the organic layers were combined, washed with water, brine, and then dried over $Na_2SO_4$ and concentrated. Purification by flash chromatography (1:6 $Et_2O$ / pentanes) gave an inseparable 7:1 mixture of **30** and **S16** (4.5 mg, 0.013 mmol, 27%) as a colourless oil.
**R**$_f$ 0.30 (3:7 petroleum ether / $Et_2O$); **$[\alpha]_D^{25}$** –59 (c = 0.49, $CHCl_3$); **IR** (thin film) $\nu_{max}$/cm$^{-1}$ 2925, 1759, 1660, 1451, 1283, 1261, 1213, 1191, 1143, 1107, 1082, 942, 881.4; **$^1$H NMR** (400 MHz, $CDCl_3$, only peaks corresponding to **30** are assigned) $\delta_H$ 7.04 (1H, d, *J* = 7.7 Hz, H18), 6.99-6.96 (1H, m, H3), 6.95 (1H, d, *J* = 7.8 Hz, H19), 6.39 (1H, dt, *J* = 12.1, 2.1 Hz, H12), 5.98 (1H, dt, *J* = 12.2, 4.7 Hz, H13), 4.89 (2H, m, H4 and H8), 3.80 (1H, dd, *J* = 8.0, 5.9 Hz, H5), 3.51 (1H, dd, *J* = 9.6, 2.4 Hz, H7), 3.05 (2H, d, *J* = 3.8 Hz, H9), 2.82–2.68 (3H, m, H6 and H16), 2.41–2.32 (2H, m, H14), 2.07-1.93 (5H, m, H15 and H21), 1.23 (3H, d, *J* = 7.1 Hz, H22); **$^{13}$C NMR** (101 MHz, $CDCl_3$, only peaks corresponding to **30** are assigned) $\delta_C$ 174.4, 146.3, 142.7, 141.1, 137.7, 133.0, 132.9, 130.6, 127.4, 126.4, 124.5, 83.3, 82.3, 79.8, 53.7, 39.8, 39.3, 35.2, 31.9, 28.5, 13.3, 10.9; **HRMS** (ESI$^+$) calc. for $C_{22}H_{24}O_3Na$ [M+Na]$^+$ 359.1618; found 359.1619.

**(2*R*,3*S*,4*S*,5*R*)-5-(5-((3S,3a*R*,6a*R*)-2,2-Dimethyl-5-oxo-3a-(prop-2-yn-1-yl)hexahydrofuro[3,2-b]furan-3-yl)-4-hydroxypent-2-yn-1-yl)-3-methyl-4-((trimethylsilyl)ethynyl)tetrahydrofuran-2-yl acetate, S17**

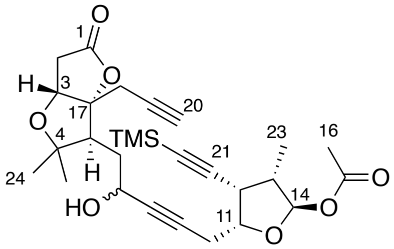

A mixture of AB ring aldehyde **4** (16.5 mg, 0.070 mmol, 1.0 equiv.) and iodoalkyne **17** (34.0 mg, 0.084 mmol, 1.2 equiv.) in a vial was azeotroped thrice with toluene then placed under an $N_2$ atmosphere. To this vial, anhydrous THF (3 mL) was added and the vial was taken into glovebox. To the solution, $CrCl_2$ (97%, 51 mg, 0.42 mmol, 6.0 equiv.) was added, the vial was sealed and removed from the golovebox, and the solution was stirred at rt for 15 h. The reaction was quenched with 1 M serine (15 mL). The aqueous layer was extracted using EtOAc (4 x 10 mL). The combined organic layers were dried ($Na_2SO_4$), concentrated, and the residue purified via flash chromatography (7:3 petroleum ether / EtOAc) to yield **S17** (31 mg, 0.060 mmol, 86%) as a white solid.

**$R_f$** 0.39 (1:1 petroleum ether / EtOAc); **IR** (thin film) $\nu_{max}/cm^{-1}$ 3448, 3286, 2971, 2171, 1785, 1375, 1250, 1197, 1018, 930, 844, 761; **$^1$H NMR** (500 MHz, $CDCl_3$) $\delta_H$ 5.91 (1H, d, $J$ = 4.1 Hz, H14), 4.53–4.46 (1H, m, H3), 4.47–4.36 (1H, m, H7), 4.35–4.26 (1H, m, H11), 3.44–3.21 (1H, m, H12), 3.07–2.94 (2H, m, H2 and H18), 2.75–2.60 (3H, m, H2 and H10), 2.56–2.41 (2H, m, H13 and H18), 2.30 (1H, dd, $J$ = 10.4, 3.2 Hz, H5), 2.20 (0.6H, d, $J$ = 4.2 Hz, OH), 2.13 (0.35H, d, $J$ = 6.0 Hz, OH*), 2.06 (4H, s, H16 and H20), 1.88–1.71 (1H, m, H6), 1.63–1.48 (1H, m, H6), 1.34 (1.71H, s, H24), 1.33 (1.36H, s, H*24), 1.20 (3H, d, $J$ = 7.2 Hz, H23), 1.20 (3H, d, $J$ = 7.2 Hz, H*23), 1.06 (3H, s, H24), 0.15 (9H, s, ($Si(CH_3)_3$)); **$^{13}$C NMR** (126 MHz, $CDCl_3$, peaks for both diastereomers are listed and not assigned) $\delta_C$ 175.5, 175.4, 170.6, 102.9, 102.9, 100.6, 100.5, 94.7, 94.5, 92.4, 92.3, 83.4, 83.4, 83.1, 82.6, 81.6, 81.4, 79.5, 79.5, 78.7, 78.7, 78.7, 77.9, 77.9, 72.3, 72.3, 61.6, 61.1, 55.7, 54.7, 43.1, 43.0, 39.9, 39.9, 37.5, 37.5, 33.8, 33.4, 27.7, 27.6, 25.9, 25.7, 22.7, 22.6, 21.3, 20.5, 13.5, 0.1, 0.0.**;** **HRMS** (ESI$^+$) calc. for $C_{28}H_{37}O_7Si$ $[M–H]^-$ 513.2314; found 513.2308.

**(2*R*,3*S*,4*S*,5*R*)-5-(5-((3*S*,3a*R*,6a*R*)-2,2-Dimethyl-5-oxo-3a-(prop-2-yn-1-yl)hexahydrofuro[3,2-b]-furan-3-yl)-4-hydroxypent-2-yn-1-yl)-4-ethynyl-3-methyltetrahydrofuran-2-yl acetate, 31**

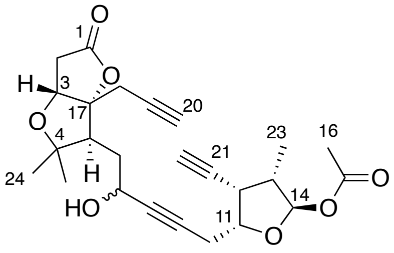

To a solution of **S17** (31 mg, 0.060 mmol, 1.0 equiv.) in THF (2.0 mL) at rt was added AcOH (3.4 µL, 0.060 mmol, 1.0 equiv.). To it was added TBAF (120 µL, 1M in THF, 0.120 mmol, 2.0 equiv.). The reaction was stirred for 15 min, monitored by TLC. After disappearance of starting material, the reaction was quenched by

addition of saturated aq. $NH_4Cl$ (10 mL). The aqueous layer was extracted with EtOAc (4 x 10 mL), and the combined organic extracts were dried ($Na_2SO_4$) and concentrated. The crude product was purified via flash chromatography (1:1 petroleum ether / EtOAc) to give **31** (21 mg, 0.048 mmol, 80%) as a white solid.

**$R_f$** 0.26 (1:1 petroleum ether / EtOAc); **IR** (thin film) $\nu_{max}/cm^{-1}$ 3451, 3287, 2981, 2886, 1775, 1473, 1378, 1237, 1197, 1131, 1061, 934; **$^1$H NMR** (500 MHz, $CDCl_3$) $\delta_H$ 5.91 (1H, d, $J$ = 4.5 Hz, H14), 5.91 (1H, d, $J$ = 4.5 Hz, H*14), 4.53–4.42 (2H, m, H3 and H7), 4.33 (1H, m, H11), 3.33 (1H, ddt, $J$ = 8.9, 6.2, 3.1 Hz, H12), 3.14–2.91 (2H, m, H2 and H18), 2.77–2.62 (3H, m, H2 and H10), 2.56–2.41 (3H, m, H13, H18 and H22), 2.38–2.32 (1H, m, H5), 2.30 (0.34H, d, $J$ = 2.5 Hz, OH*), 2.28 (0.53H, d, $J$ = 2.5 Hz, OH) 2.06 (4H, s, H16 and H20), 1.85-1.72 (1H, m, H6), 1.59–1.48 (1H, m, H6) 1.34 (3H, s, H24), 1.32 (3H, s, H*24), 1.23 (3H, d, $J$ = 7.2 Hz, H23), 1.23 (3H, d, $J$ = 7.2 Hz, H*23), 1.06 (3H, s, H24); **$^{13}$C NMR** (126 MHz, $CDCl_3$, peaks for both diastereomers are listed and not assigned) $\delta_C$ 175.5, 175.4, 170.6, 170.6, 102.9, 102.9, 94.7, 94.5, 83.4, 83.4, 82.9, 82.4, 81.8, 81.5, 79.3, 79.3, 78.7, 78.6, 78.5, 78.0, 77.8, 75.5, 75.4, 72.4, 72.2, 61.5, 61.3, 55.4, 54.7, 43.0, 43.0, 39.1, 39.0, 37.6, 37.5, 33.7, 33.2, 27.6, 27.4, 25.9, 25.7, 22.4, 22.3, 21.3, 20.5, 20.5, 13.3, 13.2; **HRMS** (ESI$^+$) calculated for $C_{25}H_{30}O_7Na$ $[M+H]^+$ 465.18837 found 465.18806.

*= peaks corresponding to second diastereomer

**Acetate 32**

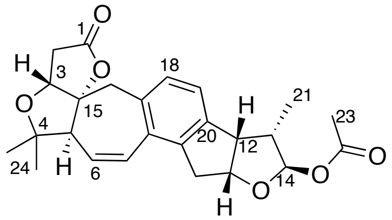


To a stirred solution of $RhCl(PPh_3)_3$ (2.0 mg, 3.00 μmol, 0.1 equiv.) in DCE (2 mL) was added a solution of **31** (10 mg, 0.021 mmol, 1.0 equiv.) in DCE (3 mL). The reaction was stirred at 50 °C for 12 h, then it was cooled to rt and concentrated. The residue was dried under vacuum.

To a solution of 2-nitrophenyl selenocyanate (15 mg, 0.063 mmol, 3.0 equiv.) in THF (0.2 mL) under inert atmosphere at rt was added *n*-$Bu_3P$ (13 mg, 0.063, 3.0 equiv.). The solution was stirred for 15 min following which a solution of the crude in THF (0.5 mL) from the previous step was added. The reaction was stirred for 2 h at rt before quenching with the $NH_4Cl$ (sat. sol.). The mixture was poured into EtOAc and the layers were separated. The aqueous layer was extracted with EtOAc and the organic layers were combined, washed with water, brine, and then dried over $Na_2SO_4$. The solvent was removed to give a residue which was passed through a short plug of silica to remove excess of 2-nitrophenyl selenocyanate and *n*-$Bu_3P$. The resulting mixture was used for the next step without further purification.

To the above crude, 1 mL THF was added followed by $H_2O_2$ (50 wt% solution in water, 15μL, 0.21 mmol, 10.0 equiv.) at rt. The resulting solution was stirred for 15 min before being quenched with $Na_2SO_3$ (sat. sol.). After stirring for 15 min, the mixture was poured into EtOAc (10 mL) and the layers were separated. The aqueous layer was extracted with EtOAc (4 × 10 mL) and the organic layers were combined, washed with

water, and then dried over $Na_2SO_4$. The solvent was removed to give a residue which was purified using column chromatography to afford alkene **32** (3.9 mg, 0.008 mmol, 40% over all yield).

**R$_f$** 0.56 (3:2 petroleum ether / EtOAc); **[α]$_D^{25}$:** +34.9 (c= 0.68, $CHCl_3$) **IR** (thin film) $\nu_{max}$/cm$^{-1}$: 2923, 2851, 1776, 1743, 1557, 1513, 1458, 1382, 1260, 1151, 1039, 954; **$^1$H NMR** (500 MHz, CDCl3) δ 7.04 (1H, d, *J* = 7.7 Hz, H18), 6.97 (1H, d, *J* = 7.6 Hz, H19), 6.59 (1H, d, *J* = 12.0 Hz, H7), 5.93 (1H, d, *J* = 1.6 Hz, H14), 5.80 (1H, br s, H6), 5.23–5.12 (1H, m, H11), 4.29 (1H, m, H3), 4.04 (1H, t, *J* = 8.1 Hz, H12), 3.20–2.80 (5H, m, H5, H10 and H16), 2.77–2.61 (2H, m, H2 and H13), 2.39–2.21 (1H, m, H2), 2.09 (3H, s, H23), 1.38 (3H, s, H24), 1.25 (3H, s, H25), 0.87 (3H, d, *J* = 7.4 Hz, H21); **$^{13}$C NMR** (126 MHz, $CDCl_3$) δ 174.6, 170.6, 141.3, 135.2, 129.4, 127.7, 125.4, 125.0, 105.7, 84.8, 68.2, 59.9, 51.6, 42.3, 38.2, 35.7, 31.9, 29.7, 26.4, 23.9, 22.7, 22.3, 21.4, 14.6, 14.1. **HRMS** (ESI$^+$) calculated for $C_{25}H_{30}O_7Na$ $[M+H]^+$ 447.17781 found 447.17791.

**Acetate 33**

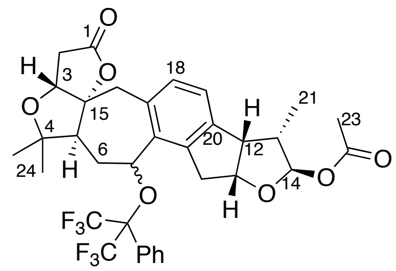


To a stirred solution of $RhCl(PPh_3)_3$ (2.0 mg, 0.003 mmol, 0.1 equiv.) in *anhyd.* DCE (2 mL), a solution of **31** (10 mg, 0.021 mmol, 1.0 equiv.) in DCE (3 mL) was added. The reaction was stirred at 50 °C. After 12 h, on completion of the reaction, the reaction vessel was cooled to rt and DCE was evaporated. The residue was dried under vacuum.

To a flame dried vial, in the glovebox, Martin's sulfurane was added (25 mg, 0.031 mmol, 1.5 equiv.). To it, 1 mL dry DCM was added followed by a solution of the crude residue (in 1 mL DCM) from the previous step. The reaction was stirred for 10 min before quenching with 5 mL $NaHCO_3$ solution. The aqueous layer was extracted with EtOAc (4 × 10 mL) and the organic layers were combined, washed with water, and then dried over $Na_2SO_4$. The solvent was removed to give a residue which was purified using column chromatography to afford acetate **33** (10 mg, 0.015 mmol, 70% over two steps).

**R$_f$** 0.56 (1:1 petroleum ether / EtOAc); **IR** (thin film) $\nu_{max}$/cm$^{-1}$ 2951, 2862, 1770 1445, 1378, 1058; **$^1$H NMR** (400 MHz, $CDCl_3$) $\delta_H$ 7.35 (1H, tt, *J* = 6.8, 1.7 Hz, Ph), 7.21–7.11 (4H, m, Ph), 7.09 (1H, d, *J* = 7.6 Hz, H18), 7.04 (1H, d, *J* = 7.6 Hz, H19), 5.78 (1H, d, *J* = 2.1 Hz, H14), 5.10 (1H, dd, *J* = 8.8, 3.8 Hz, H11), 4.64 (1H, td, *J* = 7.1, 2.3 Hz, H7), 4.59 (1H, d, *J* = 4.9 Hz, H3), 3.83 (1H, d, *J* = 14.2 Hz, H10), 3.76 (1H, t, *J* = 7.8 Hz, H12), 2.85 (1H, dd, *J* = 18.3, 5.0 Hz, H2), 2.72 (1H, d, *J* = 18.3 Hz, H2), 2.67–2.59 (2H, m, H10 and H13), 2.58–2.44 (2H, m, H5 and H16), 2.15–2.02 (1H, m, H16), 2.06 (3H, s, H23), 1.75–1.57 (2H, m, H6), 1.23 (3H, s, H24), 1.22 (3H, s, H24), 0.88 (3H, d, *J* = 7.5 Hz, H21); **$^{13}$C NMR** (101 MHz, $CDCl_3$) δ 175.2, 170.6, 142.1, 139.5, 134.7, 133.2, 130.1, 129.8, 129.1, 128.1, 127.9, 126.6, 105.4 98.4, 85.1, 84.4, 79.4, 74.3, 51.4, 50.5, 42.5, 38.5, 37.6, 36.8, 32.4, 31.9, 30.3, 24.9, 22.7, 21.3, 14.3, 14.1; **HRMS** (ESI$^+$) calc. for $C_{25}H_{30}O_7Na$ $[M+H-C_9H_6F_6O]^+$ 447.1778; found 447.1779.

**(3a*R*,6*S*,6a*R*)-6-(2-Hydroxy-5-((2*R*,3*S*,4*S*,5*S*)-4-methyl-5-((*S*)-4-methyl-5-oxo-2,5-dihydrofuran-2-yl)-3-((trimethylsilyl)ethynyl)tetrahydrofuran-2-yl)pent-3-yn-1-yl)-5,5-dimethyl-6a-(prop-2-yn-1-yl)tetrahydrofuro[3,2-b]furan-2(3*H*)-one, S18**

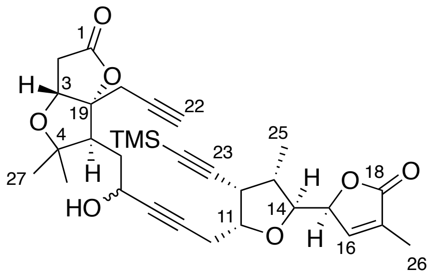


A mixture of AB ring aldehyde **4** (11.2 mg, 0.048 mmol, 1.0 equiv.) and FG ring diyne **7a** (24.2 mg, 0.055 mmol, 1.15 equiv.) was azeotroped with thrice with toluene then placed under an $N_2$ atmosphere. To this vial, anhydrous THF (2 mL) was added and the vial was taken into glovebox. $CrCl_2$ (97%, 35.0 mg, 0.29 mmol, 6.0 equiv.) was added, the vial was sealed and removed from the glovebox, and the solution was stirred at rt for 15 h. The reaction was quenched by addition of 1 M serine solution (15 mL). The aqueous layer was extracted with EtOAc (4 x 10 mL), and the combined organic layers were dried ($Na_2SO_4$) and concentrated. The residue was purified via flash chromatography (10:3 petroleum ether / EtOAc) to give **S18** (22 mg, 0.040 mmol, 83%) as a white solid (inconsequential 1:1 mix of diastereomers).

**R$_f$** 0.20 (3:2 petroleum ether / EtOAc); **IR** (thin film) $\nu_{max}$/cm$^{-1}$ 3453, 3280, 2960, 2852, 2169, 1761, 1457, 1375, 1196, 1045, 988; **$^1$H NMR** (400 MHz, $CDCl_3$) $\delta_H$ 7.07–6.95 (1H, m, H16), 4.91 (1H, dd, *J* = 2.0, 2.0 Hz, H15), 4.49 (1H, m, H3), 4.47–4.37 (1H, m, H7), 4.07–3.98 (1H, m, H11), 3.86 (1H, dd, *J* = 9.5, 2.2 Hz, H14), 3.15 (1H, dd, *J* = 6.3, 4.2 Hz, H12), 3.09–2.89 (2H, m, H2 and H20), 2.75–2.54 (4H, m, H2, H10, H13), 2.54–2.39 (1H, m, H20), 2.35–2.23 (1H, m, H5), 2.09 (1H, q, *J* = 2.7 Hz, H22), 1.93 (3H, d, *J* = 1.6 Hz, H26), 1.82–1.73 (1H, m, H6), 1.61–1.47 (1H, m, H6), 1.34 (3H, s, H27), 1.32 (3H, s, H$^*$27), 1.21 (3H, d, *J* = 7.1 Hz, H25), 1.06 (3H, s, H27), 0.15 (9H, s, $(SiCH_3)_3$); **$^{13}$C NMR** (126 MHz, $CDCl_3$, peaks for both diastereomers are listed and not assigned) $\delta_C$ 175.5, 175.5, 175.4, 174.3, 146.0, 146.0, 131.2, 131.1, 130.9, 128.8, 100.8, 100.8, 100.8, 94.7, 94.7, 94.6, 94.6, 92.5, 92.4, 92.4, 83.4, 83.4, 82.3, 80.3, 80.3, 80.1, 80.0, 78.7, 78.7, 77.9, 72.4, 68.2, 61.5, 61.4, 61.0, 60.9, 55.7, 55.7, 54.7, 42.0, 39.1, 38.7, 37.5, 37.5, 33.7, 33.6, 30.4, 28.9, 27.7, 27.7, 27.7, 25.8, 25.7, 25.7, 23.7, 23.0, 22.2, 20.5, 14.1, 13.4, 11.0, 10.8, 0.1, 0.1; **HRMS** (ESI$^+$) calc. for $C_{31}H_{40}O_3SiNa$ [M+Na]$^+$ 575.2436; found 575.24340.

**(3a*R*,6*S*,6a*R*)-6-(5-((2*R*,3*S*,4*S*,5*S*)-3-ethynyl-4-methyl-5-((*S*)-4-methyl-5-oxo-2,5-dihydrofuran-2-yl)tetrahydrofuran-2-yl)-2-hydroxypent-3-yn-1-yl)-5,5-dimethyl-6a-(prop-2-yn-1-yl)tetrahydrofuro[3,2-b]furan-2(3*H*)-one, 34**

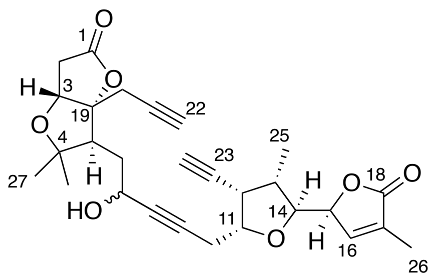


To a solution of **S18** (21 mg, 0.038 mmol, 1.0 equiv.) in THF (1.5 mL) at rt was added AcOH (2.2 µL, 0.038 mmol, 1.0 equiv.) and then TBAF (77 µL, 1M in THF, 0.077 mmol, 2.0 equiv.). The reaction was stirred for 15 min, after which time starting material had been consumed (TLC). The reaction was quenched by addition of saturated aq. $NH_4Cl$ (10 mL). The aqueous layer was extracted using EtOAc (4 x 10 mL), and the combined organic layers were dried ($Na_2SO_4$) and concentrated. The residue was purified via flash chromatography (1:1 petroleum ether / EtOAc) to yield **34** (12 mg, 0.025 mmol, 66%) as a white solid (inconsequential 1:1 mix of diastereomers).

**$R_f$** 0.28 (2:3 petroleum ether / EtOAc); **IR** (thin film) $\nu_{max}$/cm$^{-1}$ 3464, 3281, 2924, 1759, 1453, 1280, 1198, 1084, 1022, 929, 654; **$^1$H NMR** (500 MHz, $CDCl_3$) $\delta_H$ 6.99 (1H, t, *J* = 1.8 Hz, H16), 4.91 (1H, dd, *J* = 2.2, 2.1 Hz, H15), 4.51 (1H, dd, *J* = 7.1, 4.4 Hz, H3), 4.47–4.42 (1H, m, H7), 4.06 (1H, td, *J* = 7.2, 4.2 Hz, H11), 3.88 (1H, dd, *J* = 9.8, 2.3 Hz, H14), 3.21 (1H, ddt, *J* = 8.5, 6.5, 3.3 Hz, H12), 3.12–2.94 (2H, m, H2 and H20), 2.78–2.58 (4H, m, H2, H10 and H13), 2.55–2.42 (1H, m, H20), 2.32 (2H, ddd, *J* = 28.7, 10.3, 2.9 Hz, H5 and H24), 2.20–2.05 (2H, m, H22 and OH), 1.94 (3H, s, 3H), 1.84–1.72 (1H, m, H6), 1.62–1.50 (1H, m, H6), 1.34 (1.81H, s, H27), 1.33 (1.36H, s, H*27), 1.24 (3H, m, H25), 1.07 (3H, s, H27); **$^{13}$C NMR** (126 MHz, $CDCl_3$, peaks for both diastereomers are listed and not assigned) $\delta_C$ 175.5, 175.4, 174.2, 145.9, 131.2, 128.8, 94.7, 94.5, 83.4, 83.4, 83.0, 82.5, 82.4, 82.3, 81.6, 81.4, 80.1, 80.1, 79.9, 79.9, 78.8, 78.8, 78.7, 77.9, 77.8, 75.6, 75.5, 72.4, 72.3, 61.4, 61.2, 55.4, 54.7, 40.9, 39.0, 39.0, 37.6, 37.5, 33.6, 33.4, 29.7, 27.6, 27.5, 25.9, 25.7, 22.1, 20.5, 20.5, 13.3, 10.8. **HRMS** (ESI$^+$) calc. for $C_{28}H_{31}O_7$ $[M-H]^+$ 479.2075; found 479.2081.

**Rubriflordilactone B, 1**

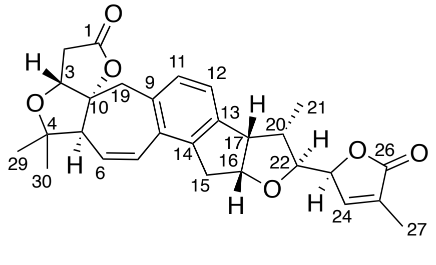


To a solution of $Bi(OTf)_3$ (25 mg, 0.038 mmol, 2.0 equiv.) in acetonitrile (0.2 mL) under Ar at –40 °C was added dropwise a solution of acetate **32** (8.0 mg, 0.019 mmol, 1.0 equiv.) and triisopropyl((3-methylfuran-2-yl)oxy) silane (9.0 mg, 0.025 mmol, 1.5 equiv.) in acetonitrile (0.5 mL). The reaction mixture was stirred at –40 °C for 1 h until TLC showed the reaction to be complete. The reaction was quenched with saturated aq. $NH_4Cl$ (1 mL) and allowed to warm to rt. The mixture was poured into $CH_2Cl_2$ (10 mL) and the layers were separated. The aqueous layer was extracted with $CH_2Cl_2$ (3 x 5 mL), and the combined organic layers were

washed with water, brine, dried over $Na_2SO_4$, and then concentrated. Purification of the residue by flash chromatography (1:6 $Et_2O$ / pentanes) afforded rubriflordilactone B (**1**) and C23-*epi*-rubriflordilactone B (***epi*-1)** as an inseparable mixture (6.0 mg, 0.021 mmol, 56%).

Alternatively, to a stirred solution of $RhCl(PPh_3)_3$ (2.0 mg, 3.00 μmol, 0.1 equiv.) in anhydrous DCE (2 mL) was added a solution of **34** (10 mg, 0.021 mmol, 1.0 equiv.) in DCE (3 mL). The reaction was stirred at 50 °C for 12 h, then it was cooled to rt and concentrated. The residue was dried under vacuum before addition of benzene (4 mL), followed by PTSA.$H_2O$ (~1.0 mg, ~4.00 μmol, 0.3 equiv.). The reaction was heated to 80 °C for 2 h, and then cooled to rt and quenched by addition of $H_2O$ (5 mL). The aqueous layer was extracted with EtOAc (4 x 10 mL). The combined organic layers were dried ($Na_2SO_4$) and concentrated. The residue was purified via flash chromatography (10:3 petroleum ether / EtOAc) to yield **1** (5.5 mg, 2.00 μmol, 57% over two steps) as a white crystalline solid.

**R$_f$** 0.40 (2:3 petroleum ether / EtOAc); $[\alpha]_D^{25}$ −12.1 (c = 0.47, $CHCl_3$); **IR** (thin film) $\nu_{max}/cm^{-1}$ 2972, 2926, 1758, 1638, 1460, 1440, 1383, 1373, 1215, 1169, 1007, 934; **$^1$H NMR** (500 MHz, $CDCl_3$) $\delta_H$ 7.14 (1H, d, *J* = 7.8 Hz, H11), 6.98 (1H, t, *J* = 1.7 Hz, H24), 6.96 (1H, d, *J* = 7.7 Hz, H12), 6.59 (1H, d, *J* = 11.8 Hz, H7), 5.80 (1H, br s, H6), 5.01–4.74 (2H, m, H16 and H23), 4.32 (1H, br s, H1), 3.81 (1H, t, *J* = 6.9 Hz, H17), 3.50 (1H, dd, *J* = 9.6, 2.1 Hz, H22), 3.18–2.85 (5H, m, H2, H15 and H19), 2.83–2.61 (3H, m, H5, H19 and H20), 1.95 (3H, t, *J* = 1.8 Hz, H27), 1.56 (3H, s, H29), 1.39 (3H, br s, H30), 1.25 (3H, d, *J* = 6.9 Hz, H21); **$^{13}$C NMR** (126 MHz, $CDCl_3$) $\delta_C$ 174.9, 174.6, 146.5, 142.5, 139.6, 133.0, 130.6, 129.2, 128.2, 127.7, 125.6, 103.7, 85.3, 83.4, 82.4, 79.7, 59.5, 53.9, 41.6, 39.5, 39.4, 35.8, 28.6, 22.3, 13.2, 11.00; **mp** 310-314 °C; **HRMS** ($ESI^+$) calc. for $C_{28}H_{30}O_6Na$ $[M+Na]^+$ 485.1935; found 485.1932. For X-ray crystal structure, see SI p48.

**(3a*R*,6*S*,6a*R*)-6-(2-Hydroxy-5-((2*R*,3*S*,4*S*,5*S*)-4-methyl-5-((*R*)-4-methyl-5-oxo-2,5-dihydrofuran-2-yl)-3-((trimethylsilyl)ethynyl)tetrahydrofuran-2-yl)pent-3-yn-1-yl)-5,5-dimethyl-6a-(prop-2-yn-1-yl)tetrahydrofuro[3,2-b]furan-2(3*H*)-one, S19**

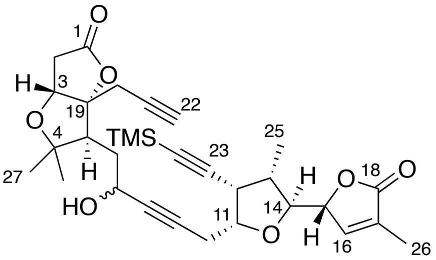


A mixture of AB ring aldehyde **4** (13.8 mg, 0.058 mmol, 1.0 equiv.) and FG ring diyne **7b** (31 mg, 0.07 mmol, 1.2 equiv.) was azeotroped with thrice with toluene then placed under an $N_2$ atmosphere. Anhydrous THF (2.5 mL) was added, and the vial was taken into glovebox. To the solution, $CrCl_2$ (97%, 43 mg, 0.35 mmol, 6.0 equiv.) was added, the vial was sealed and removed from the glovebox, and the solution was stirred at rt for 15 h. The reaction was quenched with 1 M serine solution (20 mL), and the aqueous layer was extracted with EtOAc (4 x 10 mL × 4). The combined organic layers were dried ($Na_2SO_4$) and concentrated. The residue was purified via flash chromatography (10:3 petroleum ether / EtOAc) to yield **S19** (28 mg, 0.049 mmol, 85%) as a white solid (inconsequential 1:1 mix of diastereomers).

**R$_f$** 0.43 (2:3 petroleum ether / EtOAc); **IR** (thin film) $\nu_{max}$/cm$^{-1}$ 3449, 3279, 2980, 2169, 1760, 1459, 1385, 1195, 1155, 1083, 941; **$^1$H NMR** (500 MHz, $CDCl_3$) $\delta_H$ 7.14 (1H, t, $J$ = 1.6 Hz, H16), 5.06–4.67 (1H, m, H15), 4.70–4.36 (2H, m, H3 and H7), 4.12 (1H, td, $J$ = 7.0, 3.5 Hz, H11), 3.65 (1H, dd, $J$ = 9.0, 6.1 Hz, H14), 3.16 (1H, dt, $J$ = 6.7, 4.3 Hz, H12), 3.09–2.96 (2H, m, H2 and H20), 2.75–2.59 (3H, m, H2 and H10), 2.56–2.42 (1H, m, H20), 2.41–2.26 (2H, m, H5 and H13), 2.09 (1H, t, $J$ = 2.6 Hz, H22), 1.92 (3H, s, H26), 1.86–1.73 (1H, m, H6), 1.62–1.51 (1H, m, H6), 1.34 (3H, s, H27), 1.33 (3H, s, H*27), 1.19 (3H, d, $J$ = 6.7 Hz, H25), 1.07 (3H, s, H27), 0.14 (9H, s, $(SiCH_3)_3$); **$^{13}$C NMR** (126 MHz, $CDCl_3$, peaks for both diastereomers are listed and not assigned) $\delta_C$ 175.5, 175.4, 174.1, 146.7, 130.9, 100.7, 100.7, 94.7, 94.5, 92.6, 84.0, 83.4, 83.4, 83.4, 82.9, 82.4, 81.5, 81.2, 79.5, 79.5, 78.7, 78.7, 77.9, 77.8, 72.4, 72.3, 61.5, 61.2, 55.6, 54.8, 42.4, 40.3, 40.3, 37.5, 37.5, 33.7, 33.4, 27.7, 27.6, 25.9, 25.7, 22.4, 20.5, 14.2, 10.7, 0.1; **HRMS** (ESI$^+$) calc. for $C_{31}H_{40}O_3SiNa$ $[M+Na]^+$ 575.2436; found 575.2433.

**(3a*R*,6*S*,6a*R*)-6-(5-((2*R*,3*S*,4*S*,5*S*)-3-Ethynyl-4-methyl-5-((*R*)-4-methyl-5-oxo-2,5-dihydrofuran-2-yl)tetrahydrofuran-2-yl)-2-hydroxypent-3-yn-1-yl)-5,5-dimethyl-6a-(prop-2-yn-1-yl)tetrahydrofuro[3,2-b]furan-2(3*H*)-one, 35**

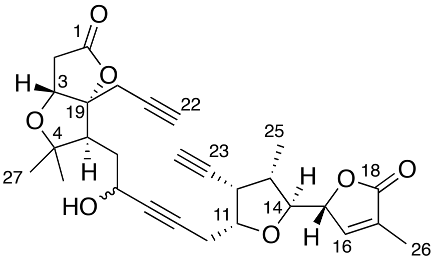


To a solution of **S19** (28 mg, 0.058 mmol, 1.0 equiv.) in THF (2.0 mL) at rt was added AcOH (6.3 µL, 0.116 mmol, 1.0 equiv.) and then TBAF (116 µL, 1M in THF, 0.116 mmol, 2.0 equiv.). The reaction was stirred for 15 min, after which time starting material had been consumed (TLC). The reaction was quenched by addition of saturated aq. $NH_4Cl$ (10 mL). The aqueous layer was extracted with EtOAc (4 x 10 mL). The combined organic layers were dried ($Na_2SO_4$) and concentrated, and the residue was purified via flash chromatography (1:3 petroleum ether / $Et_2O$) to yield **35** (19.7 mg, 0.047 mmol, 81%) as a white solid (inconsequential 1:1 mix of diastereomers).

**R$_f$** 0.31 (2:3 petroleum ether / EtOAc); **IR** (thin film) $\nu_{max}$/cm$^{-1}$ 3464, 3281, 2924, 1759, 1453, 1280, 1198, 1084, 1022, 929; **$^1$H NMR** (500 MHz, $CDCl_3$) $\delta_H$ 7.13 (1H, t, $J$ = 1.7 Hz, H16), 4.97–4.81 (1H, m, H15), 4.62–4.37 (2H, m, H3 and H7), 4.19–4.05 (1H, m, H11), 3.68 (1H, ddd, $J$ = 8.8, 6.0, 1.8 Hz, H14), 3.32–3.15 (1H, m, H12), 3.15–2.89 (2H, m, H2 and H20), 2.83–2.59 (3H, m, H2 and H10), 2.57–2.43 (1H, m, H20), 2.41–2.33 (2H, m, H5 and H13), 2.30 (1H, d, $J$ = 7.9 Hz, H*24) 2.29 (1H, d, $J$ = 7.9, 2.5 Hz, 1H), 2.09 (1H, dd, $J$ = 4.71, 2.4 Hz, 1H), 1.93 (3H, s, H26), 1.87-1.69 (1H, m, H6), 1.62–1.49 (1H, m, H6), 1.34 (3H, s, H27), 1.33 (3H, s, H*27), 1.21 (3H, d, $J$ = 6.8 Hz, H25), 1.07 (3H, s, H27). *=observable diastereomer peak; **$^{13}$C NMR** (126 MHz, $CDCl_3$, peaks for both diastereomers are listed and not assigned) $\delta_C$ 175.5, 175.4, 174.0, 146.5, 130.9, 94.7, 94.5, 84.0, 83.5, 83.4, 82.7, 82.4, 81.6, 79.4, 79.4, 78.8, 78.74, 78.69, 78.65, 78.0, 77.8,

75.7, 75.6, 72.4, 72.3, 68.2, 61.4, 61.3, 55.4, 54.7, 41.3, 40.0, 39.9, 38.7, 37.6, 37.5, 33.6, 33.2, 30.4, 28.9, 27.6, 27.4, 25.9, 25.7, 23.7, 23.0, 22.2, 22.2, 20.5, 20.5, 14.2, 14.1, 14.1, 11.0, 10.7; **HRMS** ($ESI^+$) calc. for $C_{28}H_{36}O_7N$ $[M+NH_4]^+$ 498.2486; found 498.2483.

***Epi*-rubriflordilactone B, *epi*-1**

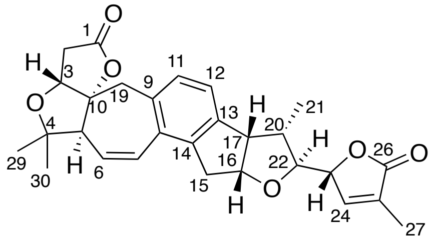


To a stirred solution of $RhCl(PPh_3)_3$ (1.9 mg, 2.05 µmol, 0.1 equiv.) in anhydrous DCE (1.5 mL) was added a solution of **35** (9.0 mg, 18.73 µmol, 1.0 equiv.) in DCE (2 mL). The reaction was stirred at 50 °C for 15 h, then it was cooled to rt and concentrated. The residue was dried under vacuum, then benzene (4 mL) was added followed by PTSA.$H_2O$ (1.2 mg, 6.30 µmol, 0.3 equiv.). The reaction was heated to 80 °C for 2 h, then cooled to rt and quenched by addition of $H_2O$ (5 mL). The aqueous layer was extracted with EtOAc (4 x 10 mL). The combined organic layers were dried ($Na_2SO_4$) and concentrated. The residue was purified via flash chromatography (10:3 petroleum ether / EtOAc) to give ***epi*-1** (5.4 mg, 11.68 µmol, 61% over two steps) as a thick colourless oil.

$\mathbf{R_f}$ 0.46 (petroleum ether / EtOAc (2:3)); $[\alpha]_D^{25}$ +92 (c = 0.50, $CHCl_3$); **IR** (thin film) $\nu_{max}/cm^{-1}$ 2980, 1761, 1655, 1460, 1383, 1322, 1215, 1168, 1067, 954; **$^1$H NMR** (500 MHz, $CDCl_3$) $\delta_H$ 7.19–7.04 (2H, m, H16 and H22), 6.96 (1H, d, $J$ = 7.7 Hz, H23), 6.60 (1H, d, $J$ = 12.0 Hz, H7), 5.85 (1H, br s, H6), 5.10-4.94 (1H, m, H15), 4.91-4.81 (1H, m, H11), 4.31 (1H, br s, H3), 3.97-3.74 (1H, m, H14), 3.30 (1H, br s, H12), 3.14-2.65 (6H, m, H2, H5, H10 and H20), 2.71 (1H, d, $J$ = 18.5 Hz, H20), 2.53 (1H, m, $J$ = 7.5 Hz, H13), 1.92 (3H, app t, $J$ = 1.8 Hz, H27), 1.46-0.98 (6H, m, H25), 1.20 (3H, d, $J$ = 7.0 Hz, H26); **$^{13}$C NMR** (126 MHz, $CDCl_3$) $\delta_C$ 174.7, 174.0, 147.0, 142.0, 139.6, 133.1, 130.5, 129.1, 127.7, 125.6, 103.7, 85.3, 84.2, 83.1, 82.5, 59.4, 54.3, 41.0, 39.2, 35.7, 28.9, 22.2, 14.0, 10.7. Note: some resonances are too broad to be resolved, as seen by the Li group; **HRMS** ($ESI^+$) calc. for $C_{28}H_{30}O_6Na$ $[M+Na]^+$ 485.1935 found 485.1932.

**(3a*R*,6*S*,6a*R*)-6-(2-Hydroxy-5-((2*S*,3*R*,4*S*,5*S*)-4-methyl-5-((*S*)-4-methyl-5-oxo-2,5-dihydrofuran-2-yl)-3-((trimethylsilyl)ethynyl)tetrahydrofuran-2-yl)pent-3-yn-1-yl)-5,5-dimethyl-6a-(prop-2-yn-1-yl)tetrahydrofuro[3,2-b]furan-2(3*H*)-one, S20**

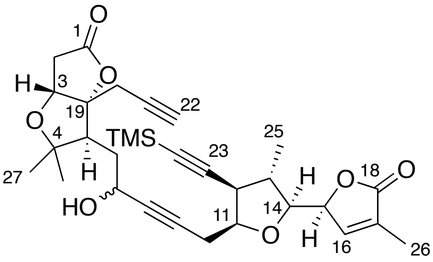


A mixture of AB ring aldehyde **4** (36 mg, 0.15 mmol, 1.0 equiv.) and FG ring diynes **8a/8d** (78 mg, 0.175 mmol, 1.15 equiv.) were azeotroped thrice with toluene then placed under an $N_2$ atmosphere. To this vial, anhydrous THF (6 mL) was added, and the vial was taken into the glovebox. To the solution, $CrCl_2$ (97%, 111 mg, 0.912 mmol, 6.0 equiv.) was added, then the vial was sealed and removed from the glovebox. The solution was stirred at rt for 15 h, then the reaction was quenched by addition of 1 M serine solution (30 mL). The aqueous layer was extracted with EtOAc (4 x 10 mL). The combined organic layers were dried ($Na_2SO_4$) and concentrated. The residue was purified via flash chromatography (10:3 petroleum ether / EtOAc) to give the triyne products (73 mg, 0.130 mmol, 87% yield). Further careful chromatography enabled separation of the diastereomers at the C14 and C15 positions (25 % EtOAc /pentane), giving the desired (C14*S*, C15*S*) alcohols **S20** (33 mg) (inconsequential 1:1 mix of diastereomers at the C7 alcohol).

**R$_f$** 0.43 (7:3 pentane / EtOAc); **IR** (thin film) $\nu_{max}$/cm$^{-1}$ 3427, 3280, 2960, 2925, 2177, 1762, 1661, 1457, 1387, 1301, 1277, 1178, 1021, 931; **$^1$H NMR** (400 MHz, $CDCl_3$) $\delta_H$ 7.03 (1H, q, *J* = 1.7 Hz, H16), 4.96 (1H, m, H15), 4.49 (1H, d, *J* = 7.0 Hz, H3), 4.45–4.35 (1H, m, H7), 4.15 (1H, dtd, *J* = 7.5, 4.6, 2.4 Hz, H11), 3.69 (1H, ddd, *J* = 9.4, 7.4, 2.2 Hz, H14), 3.17 (1H, d, *J* = 4.0 Hz, OH), 3.12–2.90 (2H, m, H2 and H20), 2.81 (1H, ddd, *J* = 10.6, 7.6, 3.5 Hz, H12), 2.66 (1H, ddd, *J* = 18.7, 3.3, 1.1 Hz, H2), 2.58–2.43 (4H, m, H10, H13 and H20), 2.33 (1H, dd, *J* = 9.6, 3.9 Hz, H5), 2.09–2.02 (1H, m, H22), 1.96 (3H, dt, *J* = 3.6, 1.8 Hz, H26), 1.89–1.74 (1H, m, H6), 1.61-1.52 (1H, m, H6), 1.35 (3H, s, H27), 1.18 (3H, d, *J* = 6.6 Hz, H$^*$25), 1.18 (3H, d, *J* = 6.6 Hz, H25), 1.08 (3H, s, 1H), 1.08 (3H, s, H$^*$27), 0.16 (9H, s, $(SiCH_3)_3$); **$^{13}$C NMR** (101 MHz, $CDCl_3$, peaks for both diastereomers are listed and not assigned) $\delta_C$ 175.5, 175.5, 174.9, 174.8, 146.2, 146.2, 131.2, 131.1, 102.5, 102.4, 94.9, 94.8, 89.7, 89.7, 83.7, 83.7, 83.4, 83.4, 82.4, 82.2, 79.1, 79.0, 78.7, 78.6, 78.1, 78.0, 77.5, 77.5, 72.2, 72.1, 61.3, 60.6, 55.6, 54.5, 43.1, 43.1, 40.7, 40.7, 37.5, 34.1, 34.0, 29.7, 29.4, 27.8, 27.7, 25.7, 25.7, 23.6, 20.5, 20.5, 15.3, 15.3, 10.8, 10.8, 0.03, 0.02; **HRMS** (ESI$^+$) calc. for $C_{31}H_{40}O_7SiNa$ $[M+Na]^+$ 575.2436; found 575.2434. (* = peaks corresponding to second diastereomer)

**(3a*R*,6*S*,6a*R*)-6-(5-((2*S*,3*R*,4*S*,5*S*)-3-Ethynyl-4-methyl-5-((*S*)-4-methyl-5-oxo-2,5-dihydrofuran-2-yl)tetrahydrofuran-2-yl)-2-hydroxypent-3-yn-1-yl)-5,5-dimethyl-6a-(prop-2-yn-1-yl)tetrahydrofuro[3,2-b]furan-2(3*H*)-one, 36**

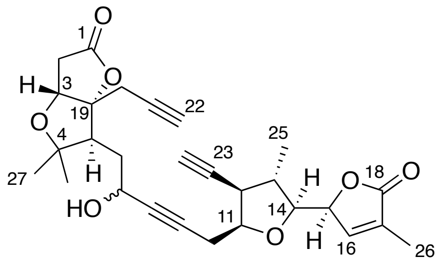


To a solution of **S20** (10.8 mg, 0.019 mmol, 1.0 equiv.) in THF (1 mL) was added AcOH (3 μL, 0.028 mmol, 2.0 equiv.) followed by TBAF (58 μL, 1 M in THF, 0.057 mmol, 3.0 equiv.). The solution was stirred for 5 min before being quenched by addition of saturated aq. $NH_4Cl$ (5 mL). The mixture was extracted with EtOAc (4 x 10 mL). The combined organic layers were dried ($Na_2SO_4$) and concentrated. The residue was purified via flash chromatography (10:3 petroleum ether / EtOAc) to provide triynes **36** (7.9 mg, 0.016 mmol, 85% yield, inconsequential 1:1 mix of diastereomers at C7 alcohol).

**$R_f$** 0.33 (3:7 EtOAc / pentane); **IR** (thin film) $\nu_{max}$/cm$^{-1}$ 3425, 3329, 2960, 2925, 2171, 1761, 1661, 1457, 1375, 1276, 1250, 1197, 1021, 932; **$^1$H NMR** (400 MHz, $CDCl_3$) $\delta_H$ 7.04 (1H, t, *J* = 1.7 Hz, H16), 5.00 (1H, q, *J* = 2.0 Hz, H15), 4.64–4.41 (2H, m, H3 and H7), 4.26–4.11 (1H, m, H11), 3.78 (1H, d, *J* = 3.5 Hz, OH), 3.68 (1H, dd, *J* = 9.4, 2.1 Hz, H$^*$14), 3.67 (1H, dd, *J* = 9.4, 2.1 Hz, H14), 3.09–2.93 (2H, m, H2 and H20), 2.83 (1H, ddd, *J* = 11.9, 8.1, 2.6 Hz, H12), 2.76–2.52 (4H, m, H2, H10 and H13), 2.51–2.47 (1H, m, H20), 2.47–2.37 (1H, m, H5), 2.28 (1H, d, *J* = 2.5 Hz, H$^*$24), 2.27 (1H, d, *J* = 2.5 Hz, H24), 2.11–2.03 (1H, m, H22), 1.96 (3H, q, *J* = 1.9 Hz, H26), 1.78 (1H, ddd, *J* = 13.9, 10.6, 5.6 Hz, H6), 1.58–1.51 (1H, m, H6), 1.35 (3H, s, H$^*$27), 1.34 (3H, s, H27), 1.23 (3H, d, *J* = 6.5 Hz, H25), 1.10 (3H, s, H$^*$27), 1.07 (3H, s, H27); **$^{13}$C NMR** (101 MHz, $CDCl_3$, peaks for both diastereomers are listed and not assigned) $\delta_C$ 175.5, 175.1, 175.1, 146.3, 146.3, 131.1, 131.0, 95.1, 94.7, 83.7, 83.7, 83.5, 83.4, 82.9, 82.6, 82.2, 81.8, 80.6, 80.5, 79.0, 78.7, 78.5, 78.2, 78.0, 77.2, 73.3, 73.1, 72.2, 72.1, 61.2, 60.7, 55.1, 54.4, 42.0, 41.9, 40.9, 40.8, 37.6, 37.6, 34.1, 33.5, 28.0, 27.4, 25.7, 23.7, 23.7, 22.7, 20.6, 20.5, 15.1, 15.0, 14.1, 10.8; **HRMS** (ESI$^+$) calc. for $C_{28}H_{32}O_7Na$ [M+Na]$^+$ 503.2040 found 503.2037. (* = peaks corresponding to second diastereomer).

***Pseudo*-rubriflordilactone B, 2**

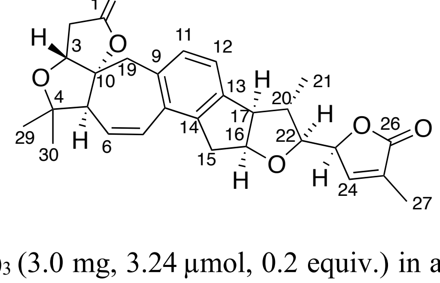


To a stirred solution of $RhCl(PPh_3)_3$ (3.0 mg, 3.24 μmol, 0.2 equiv.) in anhydrous DCE (2 mL) was added a solution of **36** (7.9 mg, 16.28 μmol, 1.0 equiv.) in DCE (0.5 mL). The mixture was stirred at 50 °C for 48 h

(until completion), then it was cooled to rt and concentrated. The residue was dried under vacuum, then benzene (2.5 mL) was added followed by PTSA.$H_2O$ (3.6 mg, 18.93 µmol, 1.1 equiv.). The reaction was heated to 80 °C for 12 h, then cooled to rt and quenched by addition of $H_2O$ (5 mL). The aqueous layer was extracted using EtOAc (4 x 10 mL), and the combined organic phases were dried using ($Na_2SO_4$) and concentrated. The residue was purified via flash chromatography (2:1 petroleum ether / EtOAc) to give **2** (3.2 mg, 6.91 µmol, 43% over two steps) as a thick colourless oil.

**$R_f$** 0.27 (1:1 EtOAc / pentane); **$[\alpha]_D^{25}$** −14.2 (c = 0.10, $CHCl_3$); **IR** (thin film) $\nu_{max}/cm^{-1}$ 2980, 2889, 1758, 1656, 1460, 1383, 1260, 1217, 1166, 1071, 1008, 952; **$^1H$ NMR** (500 MHz, $CDCl_3$) $\delta_H$ δ 7.0 (2H, s, H11 and H12), 6.61 (1H, dd, *J* = 12.1, 2.3 Hz, H7), 6.56 (1H, m, H24), 5.79 (1H, br s, H6), 4.91 (1H, dt, *J* = 6.3, 3.9 Hz, H16), 4.74 (1H, m, H23), 4.35 (1H, br s, H1), 3.80 (1H, dd, *J* = 6.8, 4.8 Hz, H22), 3.37 (1H, dd, *J* = 6.3, 4.5 Hz, H17), 3.05 (2H, s, H15αβ), 2.91–2.85 (4H, m, H2αβ, H5, and H19β), 2.73 (1H, d, *J* = 18.5 Hz, H19α), 2.18 (1H, m, H20), 1.82 (3H, t, *J* = 1.8 Hz, H27), 1.55 (3H, s, H29), 1.40 (3H, br s, H30), 1.29 (3H, d, *J* = 6.9 Hz, H21); **$^1H$ NMR** (500 MHz, Pyridine-*d5*) δ 7.05-7.13 (2H, m, H11 and H12), 6.89 (1H, br s, H24), 6.53 (1H, dd, *J* = 12.2, 2.2 Hz, H7), 5.74 (1H, br s, H6), 4.86-4.91 (2H, m, H16 and H23), 4.45 (1H, br s, H1), 3.76 (1H, dd, *J* = 7.0, 5.0 Hz, H22), 3.32 (1H, dd, *J* = 5.6, 5.4 Hz, H17), 3.21 (7H, m, H2α, H2β, H5, H15α, H15β, H19α and H19β; signal includes 3.02, 1H, dd, *J* = 18.0, 6.7 Hz; and 2.88 1H, br d, *J* = 18.0 Hz), 2.38-2.31 (1H, m, H20), 1.78 (3H, app t, *J* = 1.7 Hz, H27), 1.43-1.21 (6H, m, H29 and H30), 1.20 (3H, d, *J* = 6.9 Hz, H21).**$^{13}C$ NMR** (126 MHz, $CDCl_3$) δ 174.6, 173.7, 145.6, 143.5, 140.0, 131.2, 129.8, 128.0, 123.3, 104.0, 86.4, 85.3, 83.4, 80.4, 59.2, 58.1, 41.7, 37.9, 35.7, 29.7, 28.3, 22.1, 19.1, 10.8. **$^{13}C$ NMR** (126 MHz, Pyridine-*d5*) δ 175.5, 174.4, 147.2, 144.4, 140.7, 131.2, 130.6+, 129.1 (br)*, 128.4 (br)+, 124.0+, 103.7 (br)*, 87.3, 85.6 (br)*, 84.0, 81.5, 79.9 (br)*, 60.4 (br), 58.8, 42.9, 41.7 (br)*, 38.6, 36.2, 28.1*, 22.3, 19.0, 11.1. (+ = $sp^2$ C–H carbons identified from HSQC; * = visible at 60 °C acquisition temperature). Note: 2 peaks are missing due to broadening in $^{13}C$ NMR; **HRMS** ($ESI^+$) calc. for $C_{28}H_{30}O_6Na$ $[M+Na]^+$ 485.1935; found 485.1932.

**(3a*R*,6*S*,6a*R*)-6-(2-Hydroxy-5-((2*S*,3*R*,4*S*,5*S*)-4-methyl-5-((*R*)-4-methyl-5-oxo-2,5-dihydrofuran-2-yl)-3-((trimethylsilyl)ethynyl)tetrahydrofuran-2-yl)pent-3-yn-1-yl)-5,5-dimethyl-6a-(prop-2-yn-1-yl)tetrahydrofuro[3,2-b]furan-2(3*H*)-one, S21**

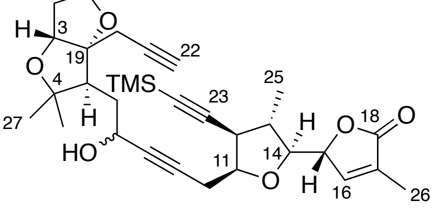


A mixture of AB ring aldehyde **4** (12 mg, 0.049 mmol, 1.0 equiv.) and FG ring diyne **8b** (25 mg, 0.057 mmol, 1.15 equiv.) were azeotroped thrice with toluene before placing under an $N_2$ atmosphere. To this vial, anhydrous THF (2 mL) was added, and the vial was taken into glovebox. To the solution, $CrCl_2$ (97%, 36 mg, 0.30 mmol, 6.0 equiv.) was added, the vial was sealed and removed from the glovebox, and then the solution was stirred at rt for 15 h. The reaction was quenched with 1 M serine solution (15 mL). The aqueous layer was

extracted using EtOAc (4 x 10 mL). The combined organic layers were dried ($Na_2SO_4$) and concentrated, and the residue was purified via flash chromatography (10:3 petroleum ether / EtOAc) to give triynes **S21** (25 mg, 0.046 mmol, 93%) (inconsequential 1:1 mix of diastereomers).

**R$_f$** 0.37 (2:3 pentane / EtOAc); **IR** (thin film) $\nu_{max}$/cm$^{-1}$ 3457, 3272, 2970, 2927, 2173, 1764, 1665, 1460, 1387, 1376, 1277, 1197, 1084, 931; **$^1$H NMR** (400 MHz, $CDCl_3$) $\delta_H$ 7.21 (1H, t, *J* = 1.7 Hz, H16), 4.99–4.80 (1H, m, H15), 4.50 (1H, dd, *J* = 6.0, 4.3 Hz, H3), 4.46–4.40 (1H, m, H7), 4.25–4.09 (1H, m, H11), 3.36 (1H, td, *J* = 6.9, 3.6 Hz, H14), 3.15–2.93 (2H, m, H2 and H20), 2.78 (1H, td, *J* = 6.9, 1.5 Hz, H12), 2.71–2.65 (1H, m, H2), 2.65–2.59 (1H, m, H10), 2.57–2.47 (2H, m, H10 and H20), 2.47–2.35 (2H, m, H13 and OH), 2.30 (1H, dd, *J* = 10.2, 3.2 Hz, H5), 2.07 (1H, t, *J* = 2.6 Hz, H22), 1.99–1.80 (3H, m, H26), 1.87–1.72 (1H, m, H6), 1.65–1.50 (1H, m, H6), 1.34 (3H, s, H27), 1.33 (3H, s, H*27), 1.15 (3H, d, *J* = 6.8 Hz, H25), 1.07 (3H, s, H27), 0.15 (9H,s, $(SiMe_3)_3$); **$^{13}$C NMR** (101 MHz, $CDCl_3$, peaks for both diastereomers are listed and not assigned) $\delta_C$ 175.5, 175.4, 174.1, 174.1, 147.3, 147.3, 130.6, 130.6, 103.4, 94.8, 94.6, 90.1, 90.05, 86.1, 86.08, 83.7, 83.4, 83.4, 83.1, 82.3, 82.2, 81.7, 81.4, 78.7, 78.6, 78.2, 78.19, 77.94, 77.9, 77.3, 72.27, 72.25, 61.7, 61.1, 55.8, 54.7, 43.7, 43.6, 43.0, 43.02, 37.6, 37.5, 33.9, 33.5, 27.6, 27.57, 25.8, 25.7, 23.4, 23.3, 20.5, 17.5, 17.47, 10.7, –0.0; **HRMS** (ESI$^+$) calc. for $C_{31}H_{40}O_7SiNa$ $[M+Na]^+$575.24355; found 575.24323. (* = peaks corresponding to second diastereomer)

**(3a*R*,6*S*,6a*R*)-6-(5-((2*S*,3*R*,4*S*,5*S*)-3-ethynyl-4-methyl-5-((*S*)-4-methyl-5-oxo-2,5-dihydrofuran-2-yl)tetrahydrofuran-2-yl)-2-hydroxypent-3-yn-1-yl)-5,5-dimethyl-6a-(prop-2-yn-1-yl)tetrahydro-furo[3,2-b]furan-2(3*H*)-one, 37**

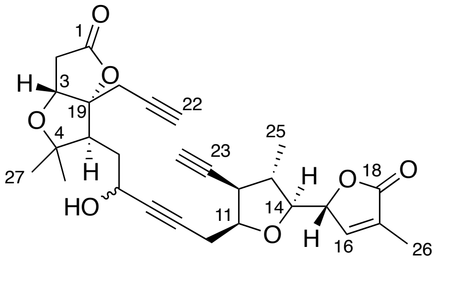


To a solution of **S21** (25 mg, 0.045 mmol, 1.0 equiv.) in THF (2 mL) was added AcOH (6.0 µL, 0.090 mmol, 2.0 equiv.) followed by TBAF (135 µL, 1 M in THF, 0.135 mmol, 3.0 equiv.). The solution was stirred for 5 min before being quenched by addition of saturated aq. $NH_4Cl$ (5 mL). The mixture was extracted with EtOAc (4 x 10 mL). The combined organic layers were dried ($Na_2SO_4$) and concentrated, and the crude purified via flash chromatography (10:3 petroleum ether / EtOAc) to provide triynes **37** (17 mg, 0.036 mmol, 80%) (inconsequential 1:1 mix of diastereomers).

**R$_f$** 0.29 (3:2 EtOAc / pentane); **IR** (thin film) $\nu_{max}$/cm$^{-1}$ 3446, 3284, 2972, 1759, 1458, 1388, 1320, 1278, 1197, 1019, 930; **$^1$H NMR** (400 MHz, $CDCl_3$) $\delta_H$ 7.21 (1H, q, *J* = 1.7 Hz, H16), 5.03–4.91 (1H, m, H15), 4.58–4.32 (2H, m, H3 and H7), 4.28–4.10 (1H, m, H11), 3.37 (1H, dd, *J* = 14.3, 7.2 Hz, H14), 3.12–2.91 (2H, m, H2 and H20), 2.78 (1H, td, *J* = 7.1, 2.4 Hz, H12), 2.73–2.56 (3H, m, H2 and H10), 2.56–2.43 (3H, m, H13, H20 and OH), 2.36–2.22 (2H, m, H5 and H24), 2.14–2.05 (1H, m, H22), 1.93 (3H, s, H26), 1.93 (3H, s, H*26), 1.87–1.74 (1H, m, H6), 1.63–1.49 (1H, m, H6), 1.34 (3H, s, H27), 1.33 (3H, s, H27), 1.17 (3H, d, *J* = 6.9 Hz,

H*25) 1.16 (3H, d, $J$ = 6.9 Hz, H25), 1.08 (3H, s, H*27) 1.07 (3H, s, H27); **$^{13}$C NMR** (101 MHz, $CDCl_3$, peaks for both diastereomers are listed and not assigned) δ 175.5, 175.4, 174.1, 174.0, 147.3, 147.2, 130.7, 130.7, 94.8, 94.5, 86.0, 86.0, 83.5, 83.4, 83.4, 82.9, 82.1, 82.1, 81.9, 81.5, 81.2, 81.1, 78.7, 78.6, 78.0, 77.9, 77.8, 73.6, 72.4, 72.2, 61.5, 60.2, 55.6, 54.6, 43.6, 43.3, 41.9, 41.9, 37.5, 37.5, 33.8, 33.4, 27.7, 27.5, 25.8, 25.7, 23.3, 23.3, 20.5, 17.4, 17.4, 10.7; **HRMS** (ESI$^+$) calc. for $C_{28}H_{32}O_7Na$ $[M+Na]^+$ 503.2040; found 503.2037. (* = peaks corresponding to second diastereomer)

**23-*Epi-pseudo*-rubriflordilactone B; *epi*-2**

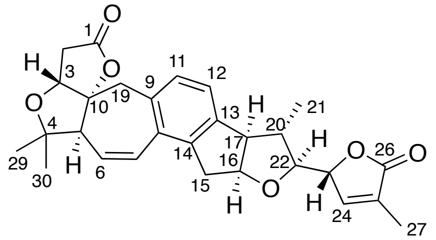


To a stirred solution of $RhCl(PPh_3)_3$ (8.4 mg, 9.08 µmol, 0.2 equiv.) in anhydrous DCE (4 mL) was added a solution of **37** (22 mg, 45.78 µmol, 1.0 equiv.) in DCE (3 mL). The reaction was stirred at 50 °C for 48 h and then cooled to rt and concentrated. The residue was dried under vacuum before adding benzene (2.5 mL), followed by PTSA.$H_2O$ (8.6 mg, 0.046 mmol, 1.0 equiv.). The reaction was heated to 80 °C for 12 h, and was then cooled to rt and quenched by addition of $H_2O$ (5 mL). The aqueous layer was extracted using EtOAc (4 x 10 mL). The combined organic layers were dried ($Na_2SO_4$) and concentrated, and the residue was purified via flash chromatography (2:1 petroleum ether / EtOAc) to yield **2** (7.5 mg, 16.48 µmol, 36% over two steps) as a thick colourless oil.

**$R_f$** 0.42 (3:2 petroleum ether / EtOAc); **$[\alpha]_D^{25}$** +18.0 (c = 0.25, $CHCl_3$); **IR** (thin film) $\nu_{max}/cm^{-1}$ 2979, 2926, 1760, 1659, 1459, 1383, 1260, 1216, 1168, 1065, 953, 799, 756; **$^1$H NMR** (500 MHz, $CDCl_3$) $\delta_H$ 7.12 (1H, d, $J$ = 7.5 Hz, H12), 7.01 (1H, d, $J$ = 7.5 Hz, H11), 6.94 (1H, br s, H24), 6.62 (1H, dd, $J$ = 12.0, 1.9 Hz, H7), 5.84 (1H, br s, H6), 4.96–4.87 (1H, m, H16), 4.32 (1H, br s, H1), 4.12 (1H, br s, H23), 3.43 (1H, dd, $J$ = 5.8, 2.6 Hz, H17), 3.35 (1H, dd, $J$ = 8.2, 4.3 Hz, H22), 3.23–2.77 (6H, m, H2α, H2β, H5, H15α, 15β, 19β), 2.71 (1H, d, $J$ = 18.4 Hz, H19α), 2.67–2.56 (1H, m, H20), 1.83 (3H, t, $J$ = 1.8 Hz, H27), 1.38 (3H, br s, H29), 1.32 (3H, d, $J$ = 7.1 Hz, H21), 1.25 (3H, br s, H29); **$^1$H NMR** (500 MHz, Pyridine-*d*5) δ 7.13 (2H, s, H11 and H12), 7.03 (1H, s, H24), 6.63–6.54 (1H, m, H7), 5.76 (1H, br s, H6), 4.86 (1H, t, $J$ = 6.1 Hz, H16), 4.50 (1H, br s, H23), 4.39 (1H, br s, H1), 3.51 (1H, dd, $J$ = 8.1, 4.3 Hz, H22), 3.33 (1H, dd, $J$ = 6.0, 3.5 Hz, H17), 3.28–3.13 (2H, m, H2β and H19β), 3.13–2.89 (4H, m, H5, H15α,15β and H19α), 2.89–2.80 (1H, m, H2α), 2.50 (1H, br s, H20), 1.73 (3H, s, H27), 1.34 (3H, br s, H29), 1.26 (3H, br s, H30), 1.18 (3H, d, $J$ = 7.0 Hz, H21); **$^{13}$C NMR** (126 MHz, $CDCl_3$) δ 174.7, 174.1, 148.0, 144.0, 140.1, 130.3, 130.2, 127.8, 123.7, 103.1, 88.2, 85.2, 83.9, 82.3, 79.9, 59.5, 58.2, 44.3, 41.9, 38.6, 35.8, 29.8, 22.1, 20.2, 10.7 ppm. **$^{13}$C NMR** (126 MHz, Pyridine-*d*5) δ 175.1, 173.9, 148.0, 144.3, 140.5, 133.9 (HMBC), 130.5, 130.1, 128.2, 123.1, 103.0, 87.7, 84.9, 83.7, 82.4, 80.4, 60.0, 58.4, 44.3, 41.5, 38.5, 35.9, 28.2, 21.9, 19.4, 10.6. Note: 2 peaks are missing due to broadening in $^{13}$C NMR; **HRMS** (ESI$^+$) calc. for $C_{28}H_{30}O_6Na$ $[M+Na]^+$ 485.1935; found 485.1932.

## 3. Data comparison tables of natural products

### Comparison of spectroscopic data for rubriflordilactone B:

**Table S1** Comparison of $^{1}H$ NMR chemical shifts of synthetic rubriflordilactone B in $CDCl_3$

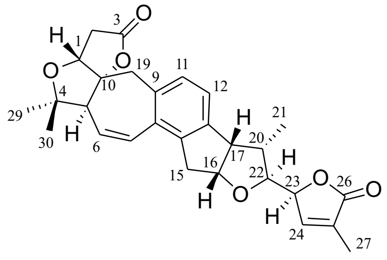

**1:** rubriflordilactone B

| Proton number | **Li *et al.***[a] δ $^{1}$H (ppm, mult, J (Hz)) 600 MHz | **Anderson *et al***[b] δ $^{1}$H (ppm, mult, J (Hz)) 500 MHz | **Δδ** (ppm) |
|---|---|---|---|
| 1 | 4.32 (br s) | 4.32 (br s) | 0.00 |
| 2 | 3.13–2.63 (m, overlapped) | 3.18-2.85 (m, overlapped) | - |
| 5 | 3.13–2.63 (m, overlapped) | 2.83-2.61 (m, overlapped) | 0.00 |
| 6 | 5.80 (br s) | 5.80 (br s) | 0.00 |
| 7 | 6.59 (d, 11.8) | 6.59 (d, 11.8) | 0.00 |
| 11 | 7.14 (d, 7.6) | 7.11 (d, 7.8) | 0.03 |
| 12 | 6.96 (d, 7.6) | 6.96 (d, 7.7 Hz) | 0.00 |
| 15 | 3.13–2.63 (m, overlapped) | 3.18-2.85 (m, overlapped) | - |
| 16 | 4.91–4.86 (m) | 5.01-4.74 (m) | - |
| 17 | 3.82 (dd, 6.7, 6.7) | 3.81 (t, 6.9 Hz) | 0.01 |
| 19α | 3.13–2.63 (m, overlapped) | 3.18-2.85 (m, overlapped) | - |
| 19β | 2.73 (d, 18.4) | 2.83-2.61 (m, overlapped) | - |
| 20 | 3.13–2.63 (m, overlapped) | 2.83-2.61 (m, overlapped) | 0.00 |
| 21 | 1.25 (d, 6.8) | 1.25 (d, 6.9) | 0.00 |
| 22 | 3.50 (dd, 9.6, 1.5) | 3.50 (dd, $J$ = 9.6, 2.1) | 0.00 |
| 23 | 4.91–4.86 (m) | 5.01-4.74 (m) | - |
| 24 | 7.00–6.97 (m) | 6.98 (t, 1.7 Hz) | 0.00 |
| 27 | 1.95 (dd, 1.6, 1.6) | 1.95 (t, 1.8) | 0.00 |
| 29 | 1.31–1.08 (m) | 1.56 (s) | 0.25 |
| 30 | 1.39 (s) | 1.39 (br s) | 0.00 |

[a] P. Yang, M. Yao, J. Li, Y. Li, A. Li, *Angew. Chem. Int. Ed.* **2016**, *55*, 6964

[b] Data were recorded in $CDCl_3$ on a Bruker AVX 500 MHz spectrometer with a cryoprobe ($^{1}H$, $^{13}C$, COSY, HSQC, HMBC).

**Table S2** Comparison of $^{13}C$ NMR chemical shifts of synthetic rubriflordilactone B in $CDCl_3$

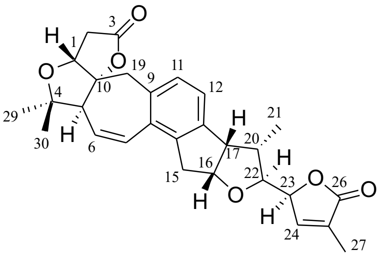

**1:** rubriflordilactone B

| Carbon number | **Li *et al.***[a] δ $^{13}C$ (ppm) 150 MHz | **Anderson *et al.***[b] δ $^{13}C$ (ppm) 125 MHz | **Δδ** (ppm) |
|---|---|---|---|
| 1 | Not visible | Not visible | - |
| 2 | 41.6 | 41.6 | 0.0 |
| 3 | 174.9 | 174.9 | 0.0 |
| 4 | 85.3 | 85.3 | 0.0 |
| 5 | 59.4 | 59.5 | -0.1 |
| 6 | 128.2 | 128.2 | 0.0 |
| 7 | 127.5 | 127.7 | -0.2 |
| 8 | Not visible | 133.0 | - |
| 9 | 142.5 | 142.5 | 0.0 |
| 10 | 103.4 | 103.7 | -0.3 |
| 11 | 125.6 | 125.6 | 1.0 |
| 12 | 129.2 | 129.2 | 0.0 |
| 13 | 139.6 | 139.6 | 0.0 |
| 14 | Not visible | 133.0[b] | - |
| 15 | Not visible | 39.5 | - |
| 16 | 79.7 | 79.7 | 0.0 |
| 17 | 53.9 | 53.9 | 0.0 |
| 19 | 35.8 | 35.8 | 0.0 |
| 20 | 39.4 | 39.4 | 0.0 |
| 21 | 13.2 | 13.2 | 0.0 |
| 22 | 82.4 | 82.4 | 0.0 |
| 23 | 83.4 | 83.4 | 0.0 |
| 24 | 146.5 | 146.5 | 0.0 |
| 25 | 130.6 | 130.6 | 0.1 |
| 26 | 174.6 | 174.6 | 0.0 |
| 27 | 11.0 | 11.0 | 0.0 |
| 29 | 28.7 | 28.6 | 0.1 |
| 30 | 22.3 | 22.3 | 0.0 |

[a] P. Yang, M. Yao, J. Li, Y. Li, A. Li, *Angew. Chem. Int. Ed.* **2016**, *55*, 6964

[b] Data were recorded in $CDCl_3$ on a Bruker AVX 500 MHz spectrometer with a cryoprobe ($^{1}H$, $^{13}C$, COSY, HSQC, HMBC). [b] Identified using HSQC.

**Comparison of spectroscopic data for *pseudo*-rubriflordilactone B:**

$^1$H NMR spectra of natural (top, 600 MHz, data from *Org. Lett.* **2006**, *8*, 991) and synthetic (bottom, 500 MHz) *pseudo*-rubriflordilactone B (**4**) (in pyridine-*d5*). It is our belief that the isolation NMR spectrum contains phthalate impurities which show resonances at 7.5 and 7.9 ppm. The inconsistency of these aromatic protons was noted by Sarotti and Kaufman (OL 2016, 6420); all other (aliphatic) protons show a very close match of chemical shift with the reported isolation data (see tables below). For further discussion see page S50.

*Natural:*

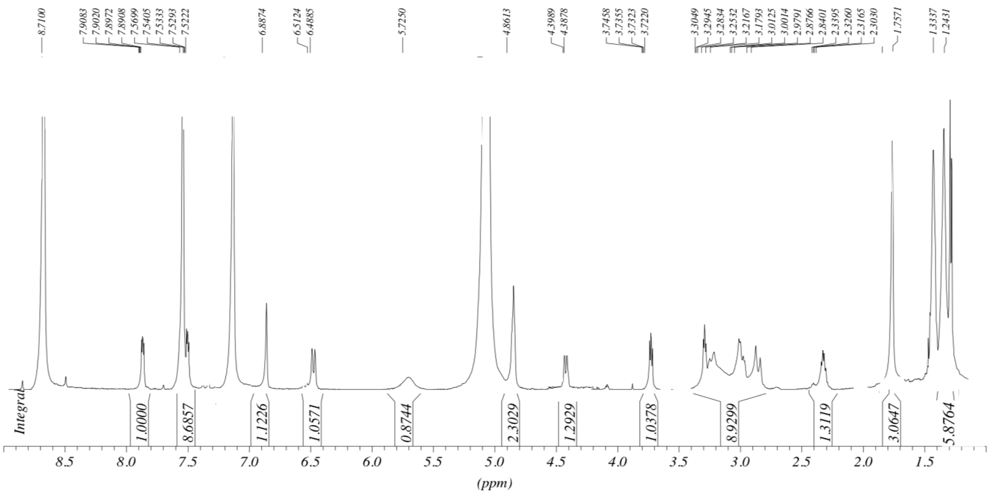


*Synthetic:*

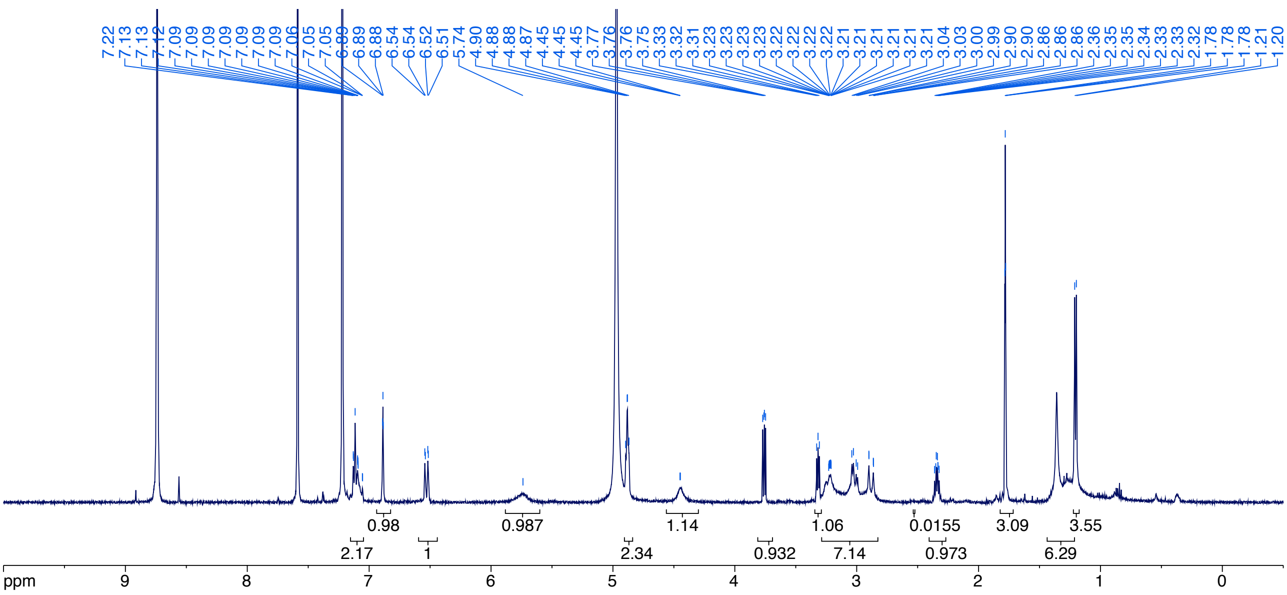

$^{13}C$ NMR spectra of natural[6] (top, 150 MHz) and synthetic (middle, 298 K, 125 MHz; bottom, 333 K / 60 °C, 125 MHz) *pseudo*-rubriflordilactone B (**4**) (in pyridine-*d5*). Note: residual $CDCl_3$ present in rt spectrum.

*Natural:*

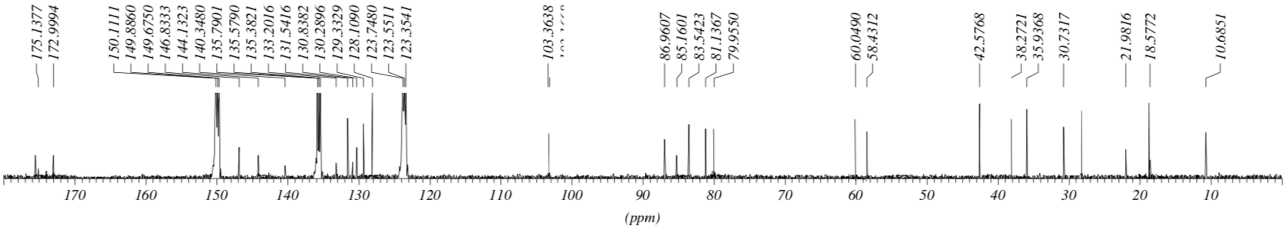


*Synthetic, rt:*

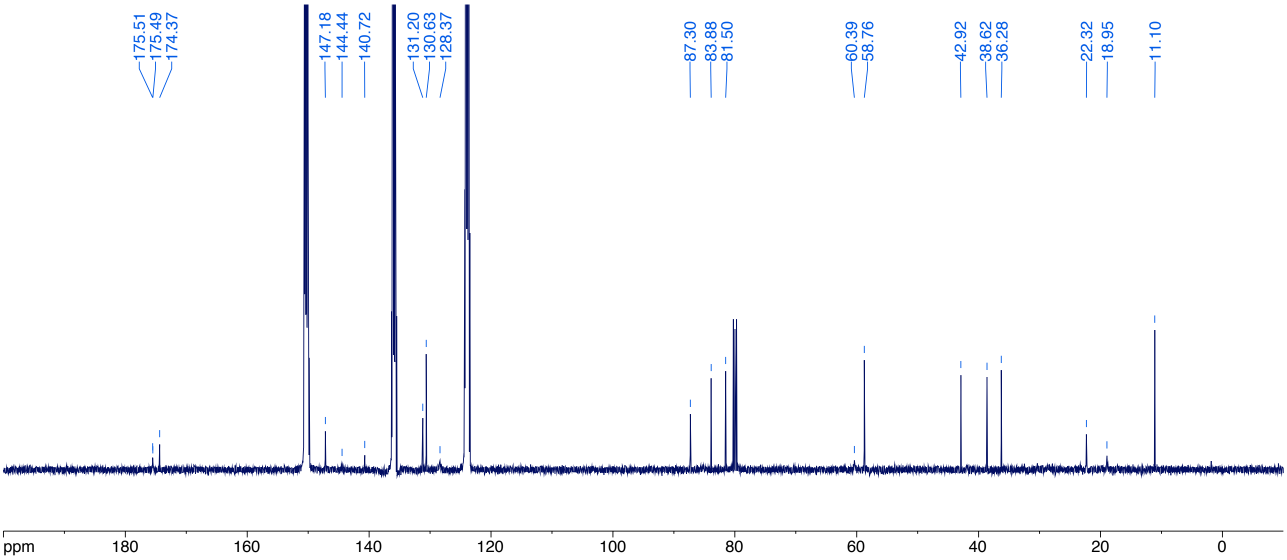


*Synthetic, 60 °C:*

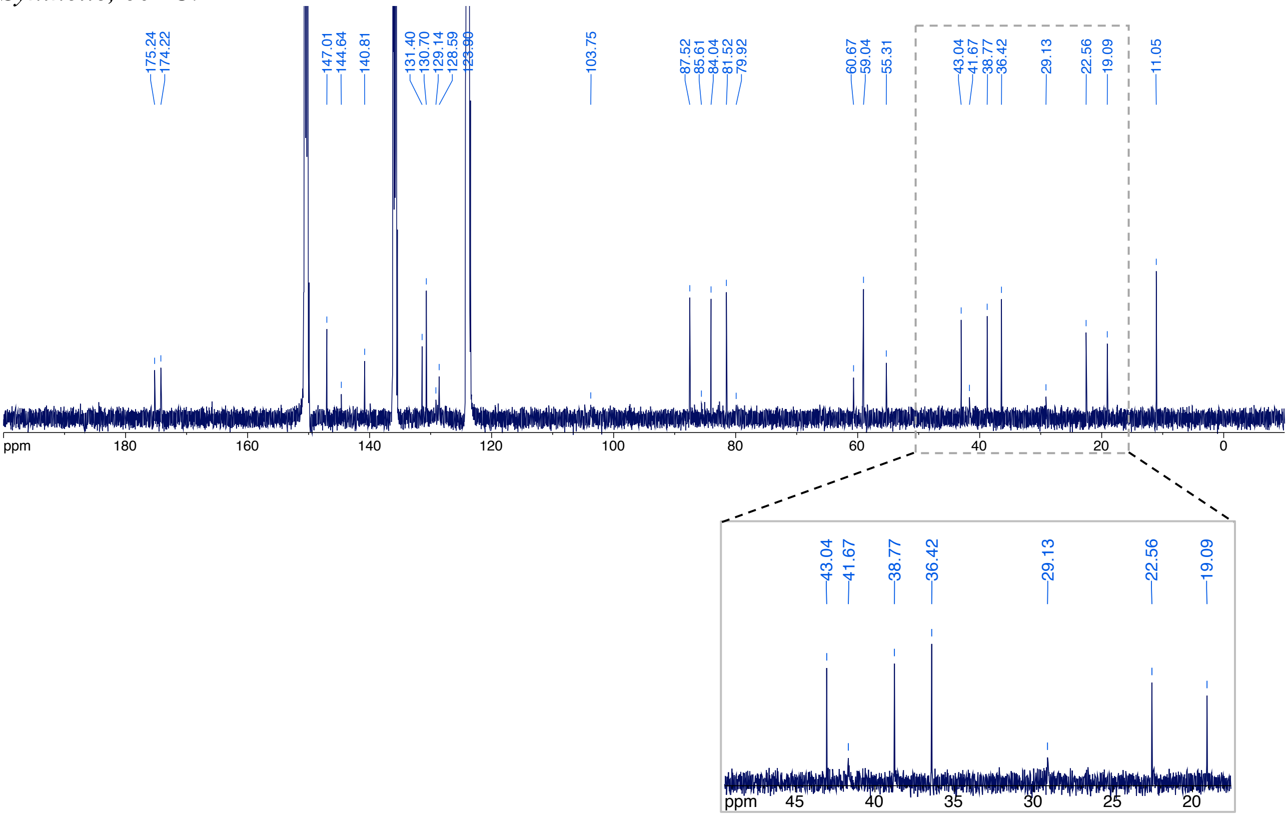

**Table S3** $^1$H NMR chemical shifts of natural and synthetic *pseudo*-rubriflordilactone B in pyridine-*d5*. Data for natural *pseudo*-rubriflordilactone B are taken W. L. Xiao *et al.*, *Org. Lett.* **2006**, *8*, 991.

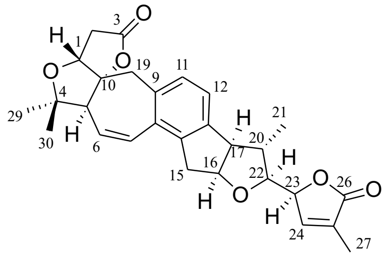

**2:** *pseudo*-rubriflordilactone B

| Proton number | **Natural**[a] δ $^1$H (ppm, mult, J (Hz)) 400 MHz | **Synthetic**[b] δ $^1$H (ppm, mult, J (Hz)) 500 MHz (ref. 7.22 ppm)[c] | **Δδ** (ppm)[c] |
|---|---|---|---|
| 1 | 4.42 (d, 5.3) | 4.45 (br s) | 0.03 |
| 2α | 2.86 (d, 18.0) | 2.88 (d, 18.2) | 0.02 |
| 2β | 3.23 (overlapped) | 3.22 (overlapped) | -0.01 |
| 5 | 2.87 (overlapped) | 2.88 (overlapped)[c] | 0.01 |
| 6 | 5.72 (br d, 12.6) | 5.74 (br s) | 0.02 |
| 7 | 6.50 (d, 12.6) | 6.53 (d, 12.1) | 0.03 |
| 11 | 7.53 (d, 5.5) | 7.08 (m) | -0.45 |
| 12 | 7.89 (d, 5.5) | 7.09 (m) | -0.80 |
| 15α | 3.23 (overlapped) | 3.22 (overlapped)[d] | -0.01 |
| 15β | 2.88 (overlapped) | 3.02 (overlapped)[e] | 0.14 |
| 16 | 4.86 (overlapped) | 4.88 (m) | 0.02 |
| 17 | 3.29 (dd, 5.3, 5.5) | 3.32 (t, 5.6) | -0.07 |
| 19α | 3.00 (d, 15.2) | 3.00 (overlapped) | 0.00 |
| 19β | 3.22 (d, 15.2) | 3.25 (overlapped) | 0.03 |
| 20 | 2.31 (m) | 2.34 (m) | -0.03 |
| 21 | 1.17 (3H, d, 8.2) | 1.20 (3H, d, 6.9) | 0.03 |
| 22 | 3.73 (dd, 5.2, 6.7) | 3.76 (t, 6.0) | 0.03 |
| 23 | 4.86 (overlapped) | 4.88 (m) | 0.02 |
| 24 | 6.88 (br s) | 6.89 (br s) | 0.01 |
| 27 | 1.76 (s) | 1.78 (s) | 0.02 |
| 29 | 1.24 (s) | 1.24 (br obscure) | 0.00 |
| 30 | 1.32 (s) | 1.33 (br s) | 0.01 |

[a] W. L. Xiao, L. M. Yang, N. B. Gong, L. Wu, R. R. Wang, J. X. Pu, X. L. Li, S. X. Huang, Y. T. Zheng, R. T. Li, Y. Lu, Q. T. Zheng, H. D. Sun, *Org. Lett.* **2006**, *8*, 991.

[b] Data were recorded in $C_5D_5N$ on a Bruker AVX 500 MHz spectrometer with a cryoprobe ($^1$H, $^{13}$C, COSY, HSQC, HMBC). Multiplets are listed here at the approximate centre of the peak.

[c] Note we are not able to judge the reference signal used in the isolation paper.

[c] Assigned from HSQC

[d] Assigned from COSY crosspeak with H16

[e] Assigned from COSY crosspeak with H15α

**Table S4** $^{13}$C NMR chemical shifts of natural and synthetic *pseudo*-rubriflordilactone B in pyridine-*d5*. Data for natural *pseudo*-rubriflordilactone B are taken W. L. Xiao *et al.*, *Org. Lett.* **2006**, *8*, 991. Data for synthetic *pseudo*-rubriflordilactone B are reported at 298 K (rt) and 333 K (60 °C), and are referenced to the solvent residual at 123.9 ppm.

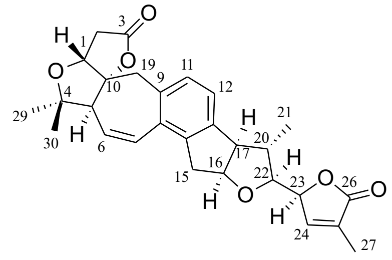

**2:** *pseudo*-rubriflordilactone B

| Carbon number | **Natural**[a] δ $^{13}$C (ppm) 100 MHz | **Synthetic**[b] δ $^{13}$C (ppm) 125 MHz, 298 K | **Synthetic**[b] δ $^{13}$C (ppm) 125 MHz, 333 K |
|---|---|---|---|
| 1 | 79.9 | – | 79.9 (br) |
| 2 | 35.9 | 36.2 | 36.4 |
| 3 | 175.1 | 175.5 | 175.2 |
| 4 | 85.1 | – | 85.6 (br) |
| 5 | 60.0 | 60.4 (br) | 60.7 |
| 6 | 128.1 | 128.4 (br)[c] | 128.6 |
| 7 | 127.9 | – | 129.1 (br)[d] |
| 8 | 133.2 | – | –[e] |
| 9 | 130.2 | – | –[e] |
| 10 | 103.4 | – | 103.7 (br) |
| 11 | 131.5 | 130.6[c] | 130.7 |
| 12 | 129.3 | 124.0[c] | 124.0 |
| 13 | 140.3 | 140.7 | 140.8 |
| 14 | 144.5 | 144.4 (br) | 144.6 (br) |
| 15 | 30.7 | – | 41.7 (br) |
| 16 | 83.5 | 84.0 | 83.9 |
| 17 | 58.4 | 58.8 | 59.0 |
| 19 | 38.2 | 38.6 | 38.8 |
| 20 | 42.5 | 42.9 | 43.0 |
| 21 | 18.6 | 19.0 | 19.1 |
| 22 | 86.9 | 87.3 | 87.5 |
| 23 | 81.1 | 81.5 | 81.5 |
| 24 | 146.8 | 147.2 | 147.0 |
| 25 | 130.8 | 131.2 | 131.4 |
| 26 | 173.0 | 174.4 | 174.2 |
| 27 | 10.7 | 11.1 | 11.0 |
| 29 | 28.3 | – | 28.1 |
| 30 | 21.9 | 22.3 | 22.6 |

[a] W. L. Xiao, L. M. Yang, N. B. Gong, L. Wu, R. R. Wang, J. X. Pu, X. L. Li, S. X. Huang, Y. T. Zheng, R. T. Li, Y. Lu, Q. T. Zheng, H. D. Sun, *Org. Lett.* **2006**, *8*, 991.

[b] Data were recorded in $C_5D_5N$ on a Bruker AVX 500 MHz spectrometer with a cryoprobe ($^1$H, $^{13}$C, COSY, HSQC, HMBC).

[c] Identified using HSQC

[d] Tentative assignment.

[e] Resonance not observed even at 60 °C.

**Table S5** $^{1}$H NMR chemical shifts of isolated natural product *pseudo*-rubriflordilactone B (**2**), and synthetic C23-*epi-pseudo*-rubriflordilactone B (***epi*-2**) in pyridine-*d5*. Data for natural *pseudo*-rubriflordilactone B are taken W. L. Xiao *et al.*, *Org. Lett.* **2006**, *8*, 991.

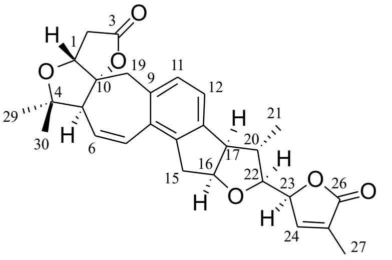

**2:** *pseudo*-rubriflordilactone B

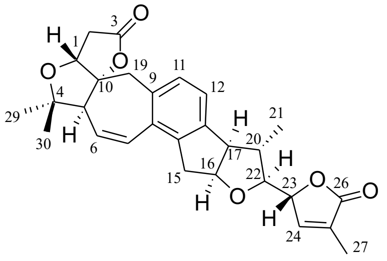

***epi*-2**: *epi-pseudo*-rubriflordilactone B

| Proton number | **Natural**[a] δ $^{1}$H (ppm, mult, J (Hz)) 400 MHz | **Synthetic**[b] δ $^{1}$H (ppm, mult, J (Hz)) 500 MHz | **Δδ** (ppm) |
|---|---|---|---|
| 1 | 4.42 (d, 5.3) | 4.39 (br s) | 0.03 |
| 2α | 2.86 (d, 18.0) | 2.84 (m) | 0.02 |
| 2β | 3.23 (overlapped) | 3.19 (overlapped) | 0.04 |
| 5 | 2.87 (overlapped) | 2.99 (overlapped) | -0.12 |
| 6 | 5.72 (br d, 12.6) | 5.76 (br s) | -0.04 |
| 7 | 6.50 (d, 12.6) | 6.59 (m) | -0.09 |
| 11 | 7.53 (d, 5.5) | 7.13 (s) | 0.40 |
| 12 | 7.89 (d, 5.5) | 7.13 (s) | 0.76 |
| 15α | 3.23 (overlapped) | 3.02 (overlapped) | 0.21 |
| 15β | 2.88 (overlapped) | 3.02 (overlapped) | -0.14 |
| 16 | 4.86 (overlapped) | 4.86 (t, 6.1) | 0 |
| 17 | 3.29 (dd, 5.3, 5.5) | 3.33 (dd, 6.0, 3.5) | -0.04 |
| 19α | 3.00 (d, 15.2) | 3.02 (overlapped) | -0.02 |
| 19β | 3.22 (d, 15.2) | 3.16 (overlapped) | 0.06 |
| 20 | 2.31 (m) | 2.50 (s) | -0.19 |
| 21 | 1.17 (d, 8.2) | 1.18 (d, 7) | -0.01 |
| 22 | 3.73 (dd, 5.2, 6.7) | 3.51 (dd, 8.1, 4.3) | 0.22 |
| 23 | 4.86 (overlapped) | 4.50 (overlapped) | 0.36 |
| 24 | 6.88 (br s) | 7.03 (s) | -0.15 |
| 27 | 1.76 (s) | 1.73 (s) | 0.03 |
| 29 | 1.24 (s) | 1.24 (overlapped) | 0 |
| 30 | 1.32 (s) | 1.24 (overlapped) | 0.08 |

[a] W. L. Xiao, L. M. Yang, N. B. Gong, L. Wu, R. R. Wang, J. X. Pu, X. L. Li, S. X. Huang, Y. T. Zheng, R. T. Li, Y. Lu, Q. T. Zheng, H. D. Sun, *Org. Lett.* **2006**, *8*, 991.

[b] Data were recorded in $C_5D_5N$ on a Bruker AVX 500 MHz spectrometer with a cryoprobe ($^{1}$H, $^{13}$C, COSY, HSQC, HMBC).

**Table S6** $^{13}$C NMR chemical shifts of isolated natural product and synthetic C23-*epi*-*pseudo*-rubriflordilactone B (***epi*-2**) in pyridine-*d5*. Data for natural *pseudo*-rubriflordilactone B are taken W. L. Xiao *et al.*, *Org. Lett.* **2006**, *8*, 991.

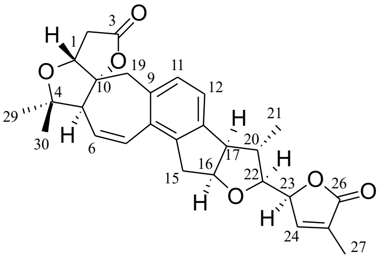

**2:** *pseudo*-rubriflordilactone B

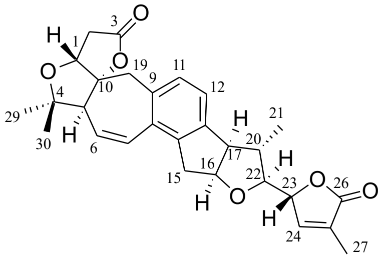

***epi*-2**: *epi*-*pseudo*-rubriflordilactone B

| Carbon number | **Natural**[a] δ $^{13}$C (ppm) 100 MHz | **Synthetic**[b] δ $^{13}$C (ppm) 125 MHz | **Δδ** (ppm) |
|---|---|---|---|
| 1 | 79.9 | 80.4 | -0.5 |
| 2 | 35.9 | 35.9 | 0 |
| 3 | 175.1 | 175.1 | 0 |
| 4 | 85.1 | 84.9 | 0.2 |
| 5 | 60.0 | 60.0 | 0 |
| 6 | 128.1 | overlapped/not visible | - |
| 7 | 127.9 | 128.2 | -0.3 |
| 8 | 133.2 | 133.9[b] | -0.7 |
| 9 | 130.2 | 130.1 | 0.1 |
| 10 | 103.4 | 103 | 0.4 |
| 11 | 131.5 | 130.1 | 1.4 |
| 12 | 129.3 | overlapped/not visible | - |
| 13 | 140.3 | 140.5 | -0.2 |
| 14 | 144.5 | 144.3 | 0.2 |
| 15 | 30.7 | 41.5 | -10.8 |
| 16 | 83.5 | 83.6 | -0.1 |
| 17 | 58.4 | 58.4 | 0 |
| 19 | 38.2 | 38.5 | -0.3 |
| 20 | 42.5 | 44.3 | -1.8 |
| 21 | 18.6 | 19.4 | -0.8 |
| 22 | 86.9 | 87.7 | -0.8 |
| 23 | 81.1 | 82.3 | -1.2 |
| 24 | 146.8 | 148.0 | -1.2 |
| 25 | 130.8 | 130.5 | 0.3 |
| 26 | 173.0 | 173.9 | -0.9 |
| 27 | 10.7 | 10.6 | 0.1 |
| 29 | 28.3 | 28.2 | 0.1 |
| 30 | 21.9 | 21.9 | 0 |

[a] W. L. Xiao, L. M. Yang, N. B. Gong, L. Wu, R. R. Wang, J. X. Pu, X. L. Li, S. X. Huang, Y. T. Zheng, R. T. Li, Y. Lu, Q. T. Zheng, H. D. Sun, *Org. Lett.* **2006**, *8*, 991.

[b] Data were recorded in $C_5D_5N$ on a Bruker AVX 500 MHz spectrometer with a cryoprobe ($^1$H, $^{13}$C, COSY, HSQC, HMBC). [b] Identified using HMBC

**Discussion of proposed misassignment at H11/C11, H12/C12, and C15:**

There are significant discrepancies between the spectroscopic data for synthetic *pseudo*-rubriflordilactone B, and the isolation paper.

**C15** (original assignment 30.7 ppm): The inconsistency of this carbon resonance was noted by Sarotti and Kaufman (OL 2016, 6420), who identifed HMQC correlations of this carbon with protons around 1.2 ppm in the isolation spectrum. This revealed a misassignment of this peak.

We found that the C15 peak was very difficult to identify, as its resonance is extremely broad at ambient temperature, and can barely be resolved from the baseline. By acquiring the $^{13}C$ NMR spectrum at 60 °C, a broad resonance was revealed at 41.7 ppm, which we assign as C15. Notably, this revised assignment is much more consistent with C15 in rubriflordilactone B (39.5 ppm).

**H11,C11/H12,C12:** We believe that the peaks originally assigned as H11 and H12 (7.53 and 7.89 ppm) in fact correspond to an isolation artifact, most likely phthalate impurities which commonly arise from plastic labware. Comparison of the $^{1}H$ NMR spectrum for dibutyl phthalate in d5-pyridine (Figure S5a) with the isolation paper reveals a compelling match between the aromatic signals at 7.5 and 7.9 ppm (Figure S5b).

Fig. S5a

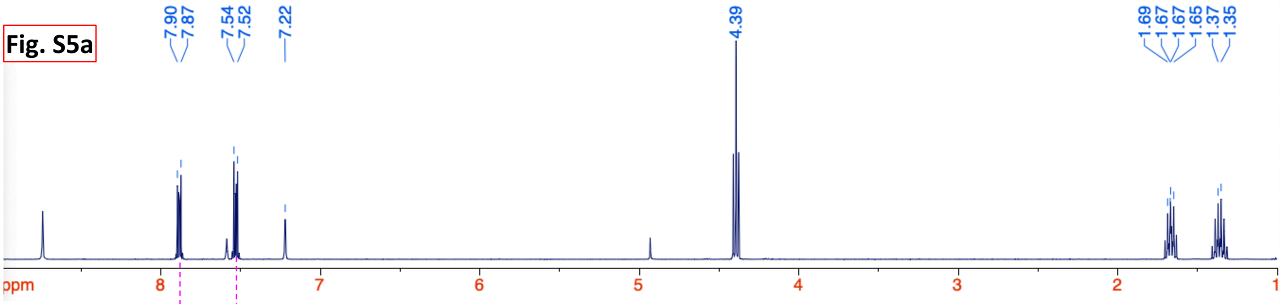


Fig. S5b

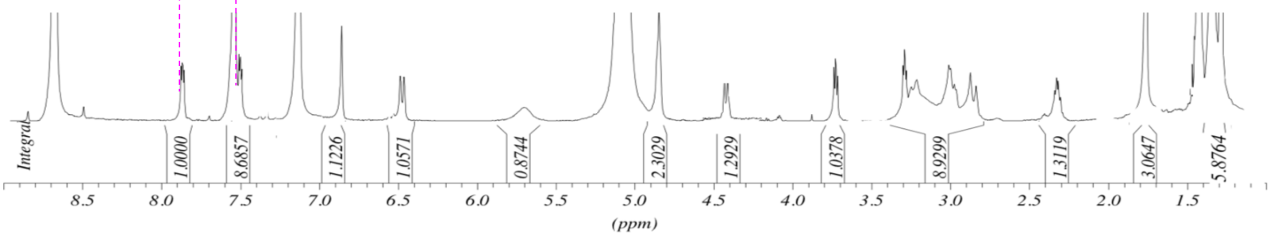


Similarly, The dibutyl phthalate $^{13}C$ NMR spectrum shows C–H carbons at 129.7 and 131.8 ppm (Figure S6a). These match with carbons at 129.3 and 131.5 ppm in the isolation paper (expansion in Figure S6b), which show HMQC cross-peaks (in the isolation paper) with the protons (Expansion Figure S6c). A further phthalate resonance at 133.5 ppm could correspond to a resonance in the isolation paper at 133.2 ppm.

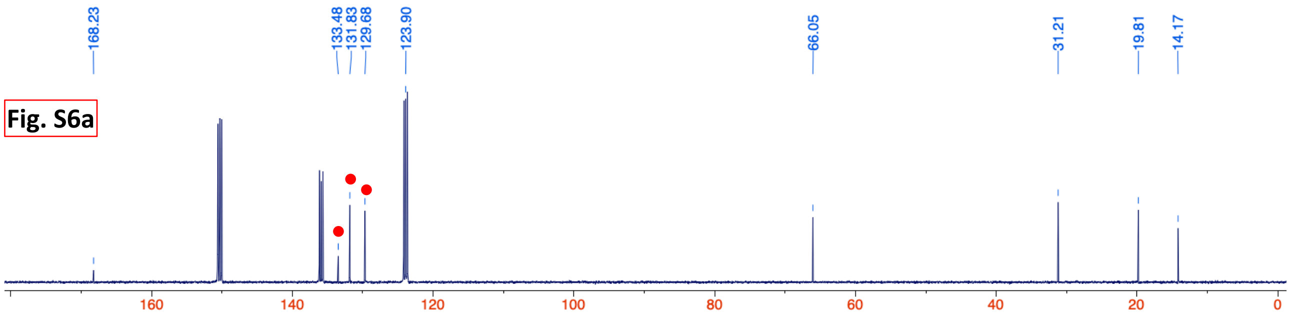


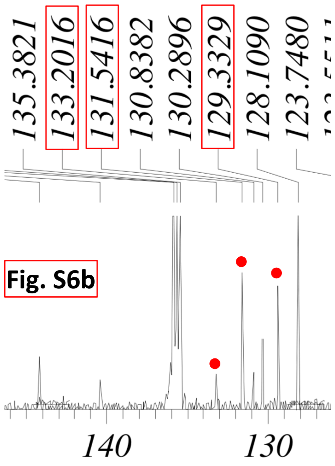


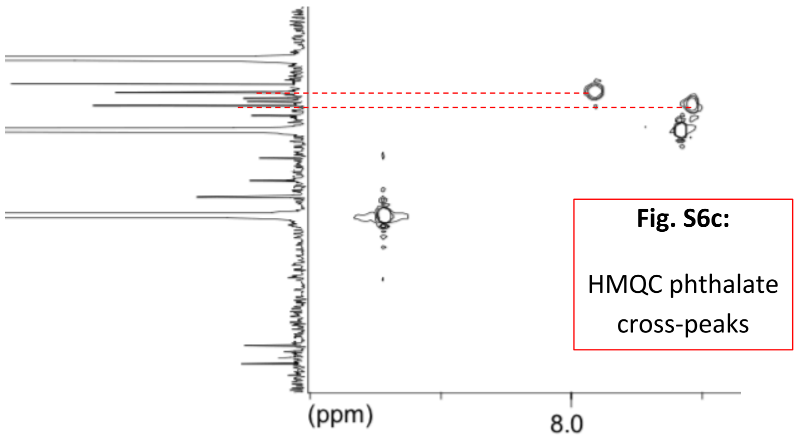


**Discussion of broad peaks in $^{13}$C NMR**: The interconversion of different rubriflordilactone conformations takes place on approximately the NMR timescale. The result is that a number of carbon signals (in particular aromatics) are significantly broadened. This no doubt led to the problems of carbon misassignment in the isolation paper, and indeed we must question the accuracy of the isolation data reported for most of these carbons. This does not invalidate the isolation of this natural product, but simply that it is very challenging to identify all carbon resonances.

The following peaks are affected; resonances that are broad or emerge only at 60 °C are listed in blue, and signals that (in our work) are not resolved at 60 °C despite long acquisition times are shown in red. Notably, these signals are clustered around the 7-membered C-ring, where it is reasonable to expect conformational interconversion on the NMR timescale. An consequence is that the corresponding cross-peaks in 2D NMR spectra are also not observed, which hinders complete identification of all carbon signals in the molecule.

C4 (60 °C)
C5
C6 (60 °C)
C7
C8 (not seen)
C9 (not seen)
C10 (60 °C)
C14
C15 (60 °C)
C29 (60 °C)

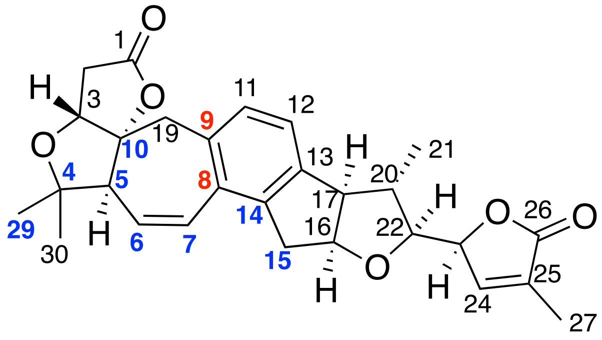

## 4. X-ray Crystallography

Low temperature single crystal X-ray diffraction data were collected for **1**, **7b** and **16a** with a (Rigaku) Oxford Diffraction SuperNova A diffractometer at 150 K, and data for **8c** were collected using I19-1 at the Diamond Light Source[1] at 100 K. All data were reduced using CrysAlisPro, solved using SuperFlip[2] and the structures were refined using CRYSTALS.[3]

Structure **7b** contained solvent accessible voids comprising of weak, diffuse electron density. The discrete Fourier transforms of the void regions were treated as contributions to the A and B parts of the calculated structure factors using PLATON/SQUEEZE[4] integrated within the CRYSTALS software. This enabled a comparison of models, one of which contained the disordered solvent, the other without. The change in R index (all data) was small, 3.10 versus 3.06 without the disordered solvent, and the model without the disordered solvent was thus chosen.

The Flack x parameter[5] was refined in all cases. Bayesian analysis of the Bijvoet pairs was also carried out using all the data used in the refinement.[6] This gave the Hooft y parameter, the P2 probability (the likelihood that the hand is correct given the crystal was enantiopure), and the P3 probability (the likelihood that the hand is correct given the crystal was enantiopure or racemic).

Further details about the refinements, including disorder modelling and restraints, are documented in the CIF. The crystallographic data have been deposited with the CCDC as entries CCDC 1904829–1904832.

[1] D. R. Allan, H. Nowell, S. A. Barnett, M. R. Warren, A. Wilcox, J. Christensen, L. K. Saunders, A. Peach, M. T. Hooper, L. Zaja, S. Patel, L. Cahill, R. Marshall, S. Trimnell, A. J. Foster, T. Bates, S. Lay, M. A. Williams, P. V. Hathaway, G. Winter, M. Gerstel & R. W. Wooley, *Crystals*, **2017**, *7*(11), 336.
[2] L. Palatinus & G. Chapuis, *J. Appl. Cryst.*, **2007**, *40*, 786–790.
[3] a) P. W. Betteridge, J. R. Carruthers, R. I. Cooper, K. Prout, & D. J. Watkin, *J. Appl. Cryst.*, **2003**, *36*, 1487; b) R. I. Cooper, A. L. Thompson & D. J. Watkin, *J. Appl. Cryst.* **2010**, *43*, 1100–1107.
[4] a) Spek, A., *J. Appl. Cryst.*, **2003**, *36*, 7–13; b) van der Sluis, P.; A. L. Spek, *Acta Cryst.*, **1990**, *A46*, 194–201.
[5]. H. D. Flack, *Acta Crystallogr. A* **1983**, *39*, 876–881; H. D. Flack, G. Bernardinelli, *J. Appl. Crystallogr.* **2000**, *33*, 1143–1148.
[6] R. W. W. Hooft, L. H. Straver, A. L. Spek, *J. Appl. Crystallogr.* **2008**, *41*, 96–103.

**Table S7.** Crystal data and structure refinement for **1** (***Rubriflordilactone-B***).

| | | |
|---|---|---|
| CCDC Identification code | 1904829 | |
| Empirical formula | C28 H30 O6 | |
| Formula weight | 462.54 | |
| Temperature | 150 K | |
| Wavelength | 1.54184 Å | |
| Crystal system | Orthorhombic | |
| Space group | P 21 21 21 | |
| Unit cell dimensions | a = 10.16740(10) Å | $\alpha$= 90°. |
| | b = 10.77070(10) Å | $\beta$= 90°. |
| | c = 21.5191(3) Å | $\gamma$ = 90°. |
| Volume | 2356.56(5) $Å^3$ | |
| Z | 4 | |
| Density (calculated) | 1.304 $Mg/m^3$ | |
| Absorption coefficient | 0.740 $mm^{-1}$ | |
| F(000) | 984 | |
| Crystal size | 0.16 x 0.16 x 0.02 $mm^3$ | |
| Theta range for data collection | 4.109 to 75.911°. | |
| Index ranges | -12<=h<=10, -13<=k<=12, -27<=l<=26 | |
| Reflections collected | 41106 | |
| Independent reflections | 4900 [R(int) = 0.0452] | |
| Completeness to theta = 75.911° | 99.9 % | |
| Absorption correction | Semi-empirical from equivalents | |
| Max. and min. transmission | 0.99 and 0.89 | |
| Refinement method | Full-matrix least-squares on $F^2$ | |
| Data / restraints / parameters | 4898 / 0 / 308 | |
| Goodness-of-fit on $F^2$ | 1.0084 | |
| Final R indices [I>2sigma(I)] | R1 = 0.0337, wR2 = 0.0874 | |
| R indices (all data) | R1 = 0.0359, wR2 = 0.0899 | |
| Largest diff. peak and hole | 0.29 and -0.16 e.$Å^{-3}$ | |
| Absolute structure parameter (Flack) | -0.087(15) for 2113 Friedel pairs | |
| Absolute structure parameter (Hooft) | -0.034(6) for 2042 Friedel pairs | |
| Hooft probability analysis | P2(correct) > 99.9999%, P3(correct) >99.9999% | |

**Table S8.** Crystal data and structure refinement for **7b**.

| | | |
|---|---|---|
| CCDC Identification code | 1904830 | |
| Empirical formula | C18 H23 I1 O3 Si1 | |
| Formula weight | 442.37 | |
| Temperature | 150 K | |
| Wavelength | 1.54184 Å | |
| Crystal system | Monoclinic | |
| Space group | P 21 | |
| Unit cell dimensions | a = 5.74910(10) Å | $\alpha$= 90°. |
| | b = 10.7705(2) Å | $\beta$= 90.519(2)°. |
| | c = 16.6286(3) Å | $\gamma$ = 90°. |
| Volume | 1029.61(3) $Å^3$ | |
| Z | 2 | |
| Density (calculated) | 1.427 $Mg/m^3$ | |
| Absorption coefficient | 12.862 $mm^{-1}$ | |
| F(000) | 444.000 | |
| Crystal size | 0.09 x 0.07 x 0.01 $mm^3$ | |
| Theta range for data collection | 4.892 to 76.342°. | |
| Index ranges | -7<=h<=7, -13<=k<=13, -20<=l<=20 | |
| Reflections collected | 21593 | |
| Independent reflections | 4271 [R(int) = 0.0565] | |
| Completeness to theta = 76.342° | 99.6 % | |
| Absorption correction | Semi-empirical from equivalents | |
| Max. and min. transmission | 0.88 and 0.49 | |
| Refinement method | Full-matrix least-squares on $F^2$ | |
| Data / restraints / parameters | 4270 / 391 / 264 | |
| Goodness-of-fit on $F^2$ | 0.9937 | |
| Final R indices [I>2sigma(I)] | R1 = 0.0280, wR2 = 0.0684 | |
| R indices (all data) | R1 = 0.0306, wR2 = 0.0705 | |
| Largest diff. peak and hole | 0.71 and -0.49 e.$Å^{-3}$ | |
| Absolute structure parameter (Flack) | -0.022(6) for 2006 Friedel pairs | |
| Absolute structure parameter (Hooft) | -0.032(6) for 1951 Friedel pairs | |
| Hooft probability analysis | P2(correct) > 99.9999%, P3(correct) >99.9999% | |

**Table S9.** Crystal data and structure refinement for **8c**.

| | | |
|---|---|---|
| CCDC Identification code | 1904831 | |
| Empirical formula | C18 H23 I O3 Si | |
| Formula weight | 442.37 | |
| Temperature | 100 K | |
| Wavelength | 0.68890 Å | |
| Crystal system | Orthorhombic | |
| Space group | P 21 21 21 | |
| Unit cell dimensions | a = 5.59160(10) Å | α= 90°. |
| | b = 11.18080(10) Å | β= 90°. |
| | c = 31.4928(4) Å | γ = 90°. |
| Volume | 1968.88(5) Å$^3$ | |
| Z | 4 | |
| Density (calculated) | 1.492 Mg/m$^3$ | |
| Absorption coefficient | 1.541 mm$^{-1}$ | |
| F(000) | 888 | |
| Crystal size | 0.14 x 0.01 x 0.01 mm$^3$ | |
| Theta range for data collection | 1.873 to 36.035°. | |
| Index ranges | -9<=h<=9, -18<=k<=18, -52<=l<=51 | |
| Reflections collected | 40497 | |
| Independent reflections | 9616 [R(int) = 0.064] | |
| Completeness to theta = 34.323° | 99.9 % | |
| Absorption correction | Semi-empirical from equivalents | |
| Max. and min. transmission | 0.98 and 0.69 | |
| Refinement method | Full-matrix least-squares on F$^2$ | |
| Data / restraints / parameters | 9606 / 0 / 209 | |
| Goodness-of-fit on F$^2$ | 1.0130 | |
| Final R indices [I>2sigma(I)] | R1 = 0.0340, wR2 = 0.0829 | |
| R indices (all data) | R1 = 0.0378, wR2 = 0.0843 | |
| Largest diff. peak and hole | 1.17 and -1.08 e.Å$^{-3}$ | |
| Absolute structure parameter (Flack) | -0.018(10) for 4051 Friedel pairs | |
| Absolute structure parameter (Hooft) | -0.020(8) for 3881 Friedel pairs | |
| Hooft probability analysis | P2(correct) > 99.9999%, P3(correct) >99.9999% | |

**Table S10.** Crystal data and structure refinement for **16a**.

| | | |
|---|---|---|
| CCDC Identification code | 1904832 | |
| Empirical formula | C15 H22 O3 Si1 | |
| Formula weight | 278.42 | |
| Temperature | 150 K | |
| Wavelength | 1.54180 Å | |
| Crystal system | Orthorhombic | |
| Space group | P 21 21 21 | |
| Unit cell dimensions | a = 8.99150(10) Å | $\alpha$= 90°. |
| | b = 10.9371(2) Å | $\beta$= 90°. |
| | c = 16.3586(3) Å | $\gamma$ = 90°. |
| Volume | 1608.72(5) $Å^3$ | |
| Z | 4 | |
| Density (calculated) | 1.150 $Mg/m^3$ | |
| Absorption coefficient | 1.304 $mm^{-1}$ | |
| F(000) | 600 | |
| Crystal size | 0.17 x 0.10 x 0.04 $mm^3$ | |
| Theta range for data collection | 4.864 to 76.263°. | |
| Index ranges | -11<=h<=11, -13<=k<=13, -20<=l<=20 | |
| Reflections collected | 34467 | |
| Independent reflections | 3372 [R(int) = 0.0432] | |
| Completeness to theta = 76.263° | 99.9 % | |
| Absorption correction | Semi-empirical from equivalents | |
| Max. and min. transmission | 0.95 and 0.88 | |
| Refinement method | Full-matrix least-squares on $F^2$ | |
| Data / restraints / parameters | 3371 / 0 / 173 | |
| Goodness-of-fit on $F^2$ | 1.0140 | |
| Final R indices [I>2sigma(I)] | R1 = 0.0219, wR2 = 0.0564 | |
| R indices (all data) | R1 = 0.0234, wR2 = 0.0574 | |
| Largest diff. peak and hole | 0.17 and -0.14 e.$Å^{-3}$ | |
| Absolute structure parameter (Flack) | 0.003(16) for 1430 Friedel pairs | |
| Absolute structure parameter (Hooft) | -0.003(8) for 1411 Friedel pairs | |
| Hooft probability analysis | P2(correct) > 99.9999%, P3(correct) >99.9999% | |

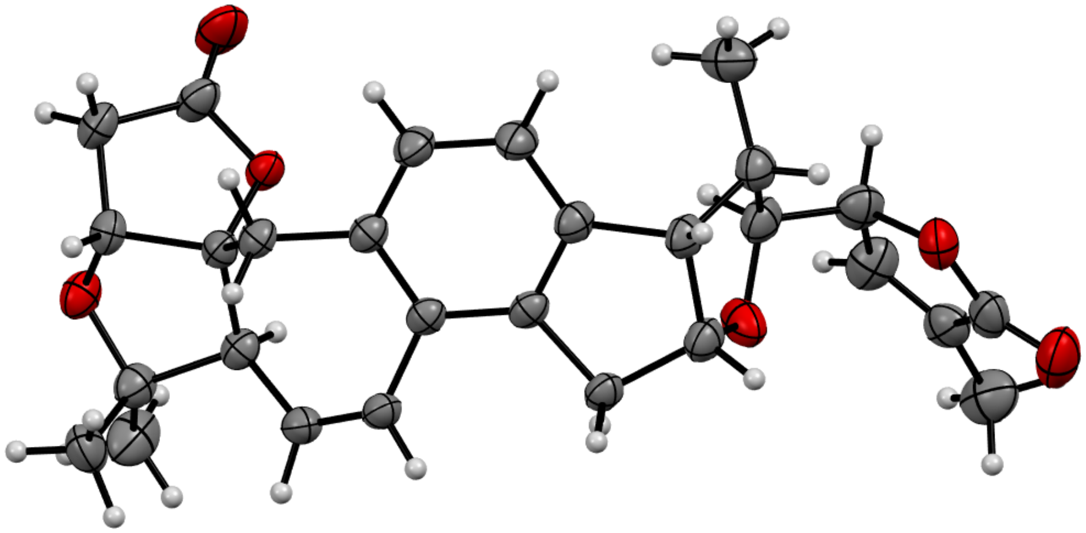

**Figure S1:** Solid state structure of **1** (***Rubriflordilactone B***). Displacement ellipsoid plots are drawn at 50% probability.

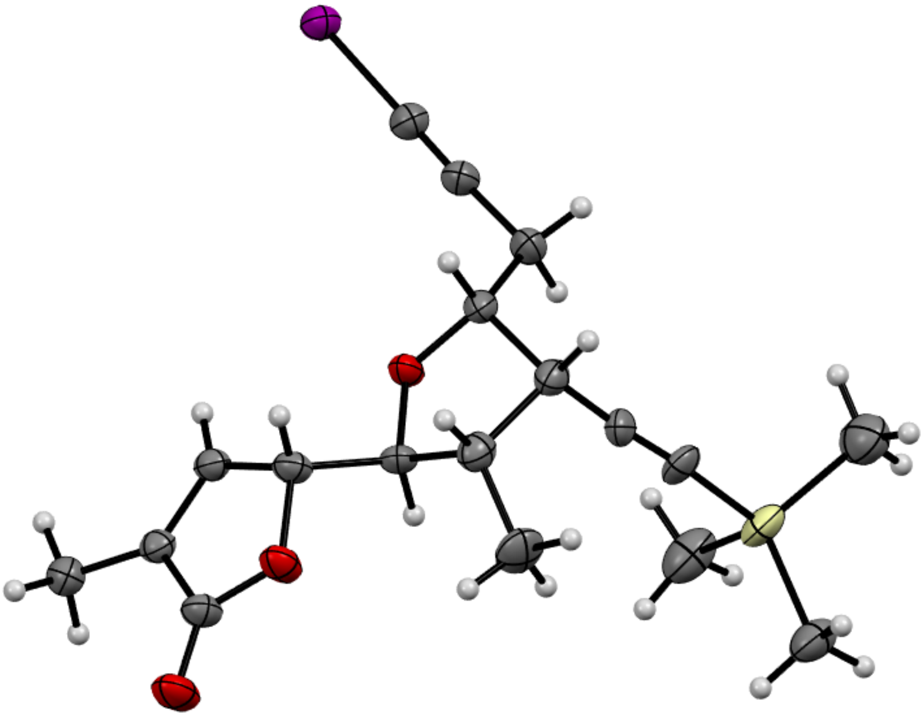

**Figure S2:** Solid state structure of **7b**. Displacement ellipsoid plots are drawn at 50% probability. Disordered components are omitted for clarity

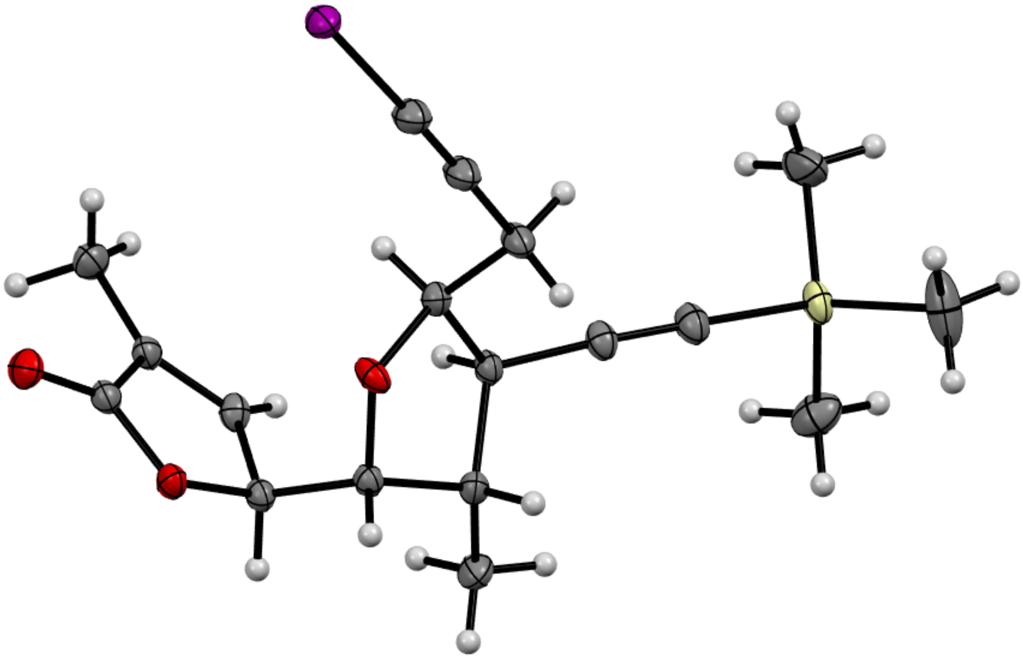

**Figure S3:** Solid state structure of **8c**. Displacement ellipsoid plots are drawn at 50% probability.

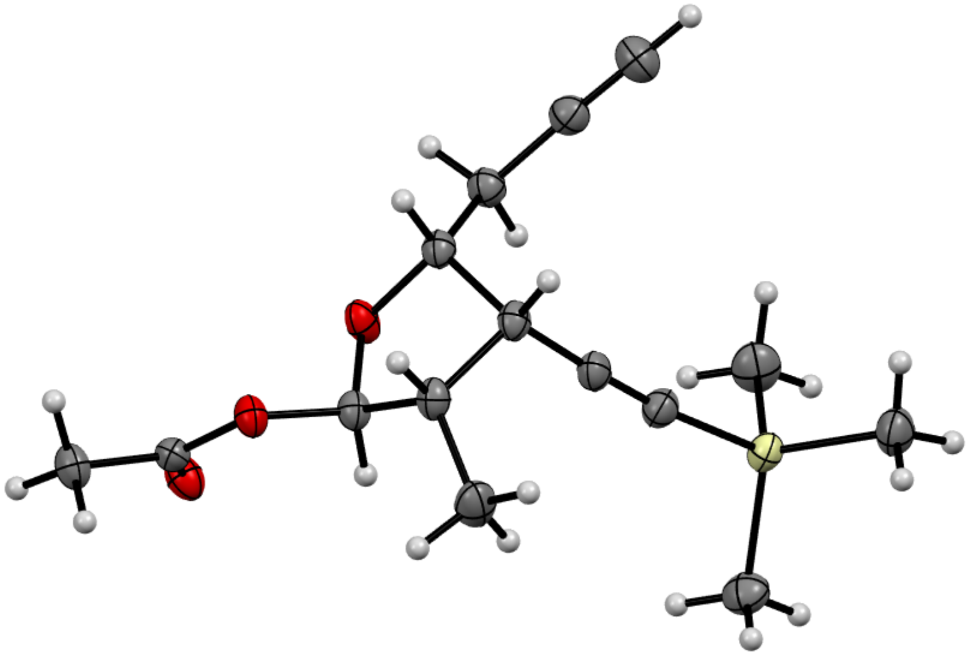

**Figure S4:** Solid state structure of **16a**. Displacement ellipsoid plots are drawn at 50% probability.

## 5. Copies of NMR spectra

**(*R*)-4-(2-((4-methoxybenzyl)oxy)ethyl)oxetan-2-one, 10**

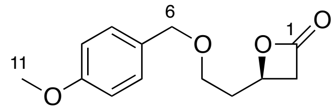

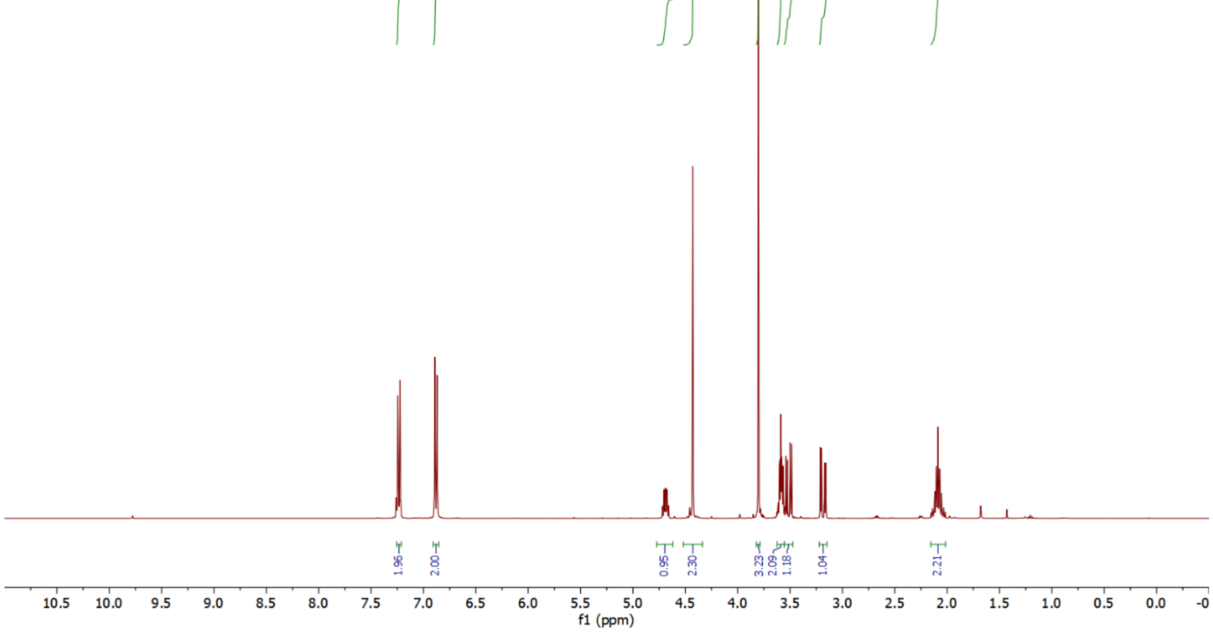


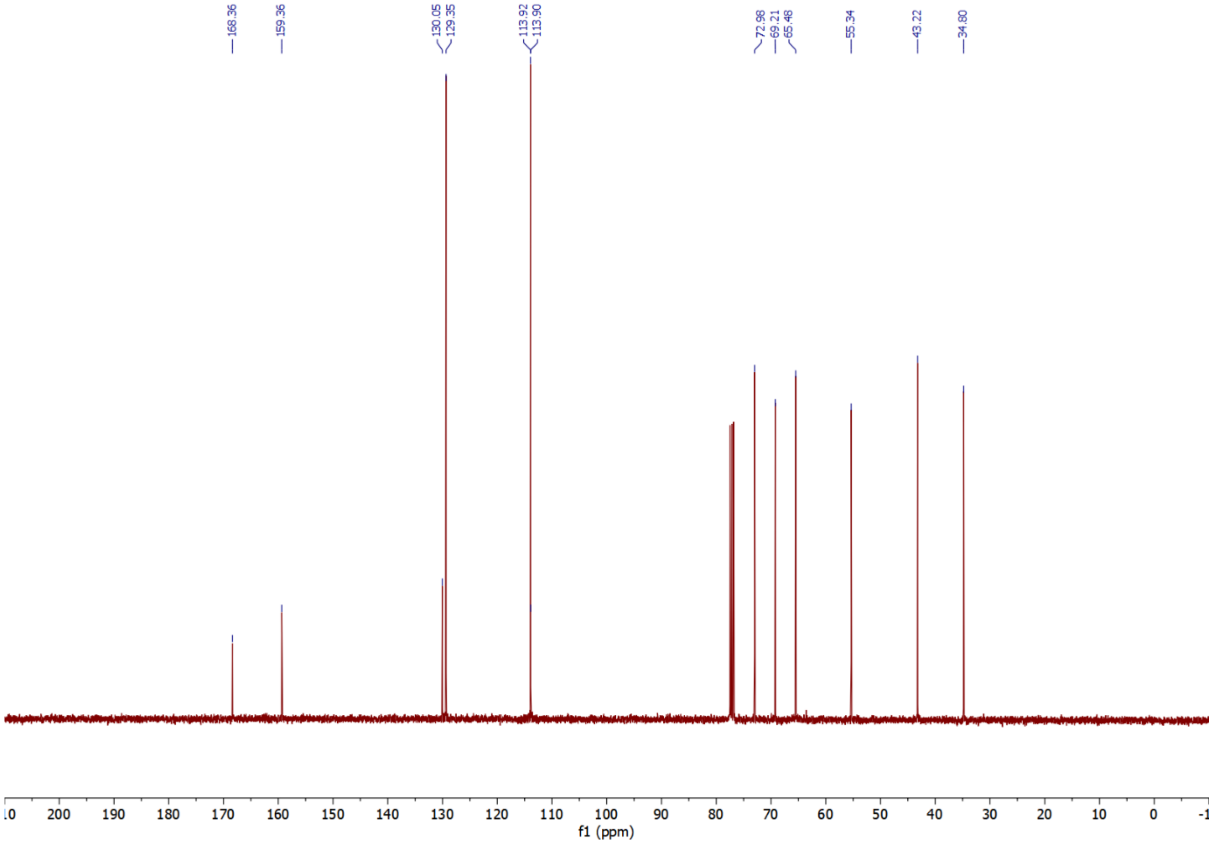

**(*R*)-(*S*,*E*)-pent-3-en-2-yl 3-hydroxy-5-((4-methoxybenzyl)oxy)pentanoate, 12**

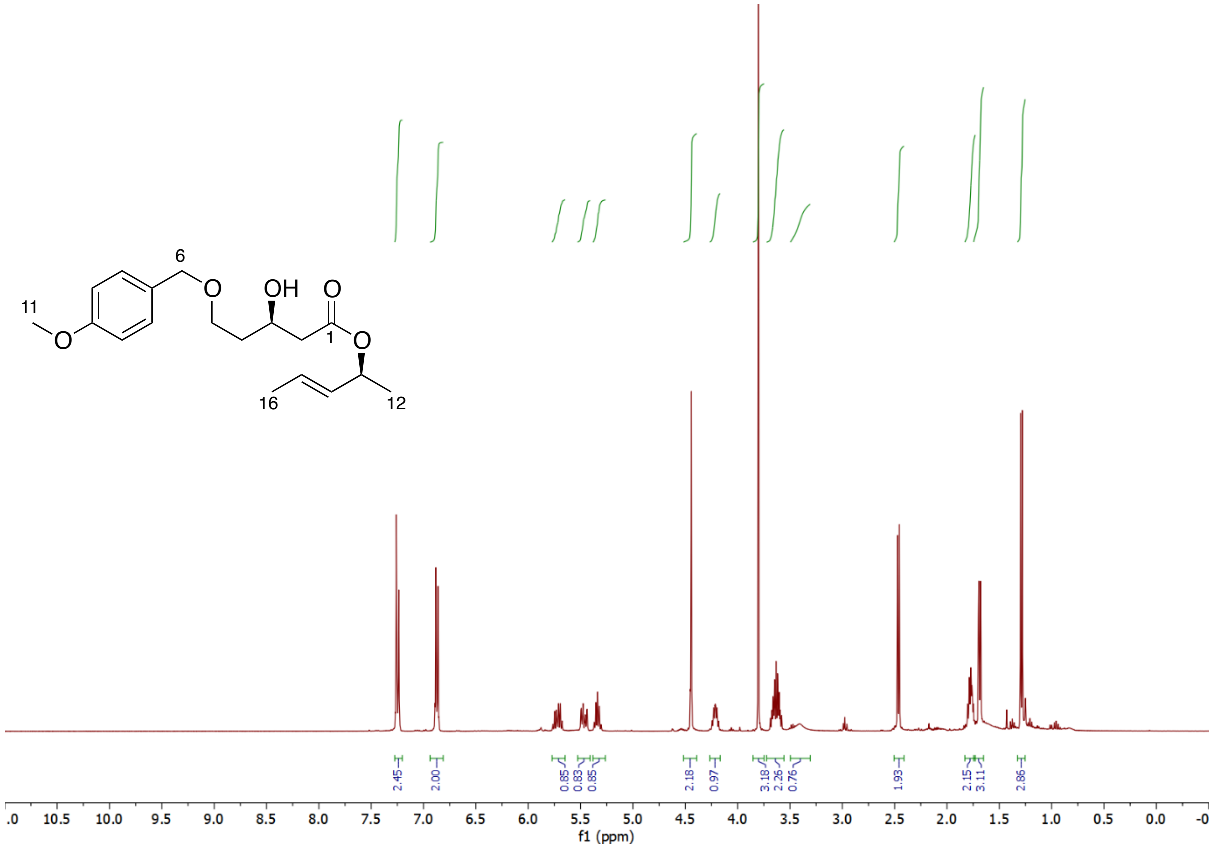


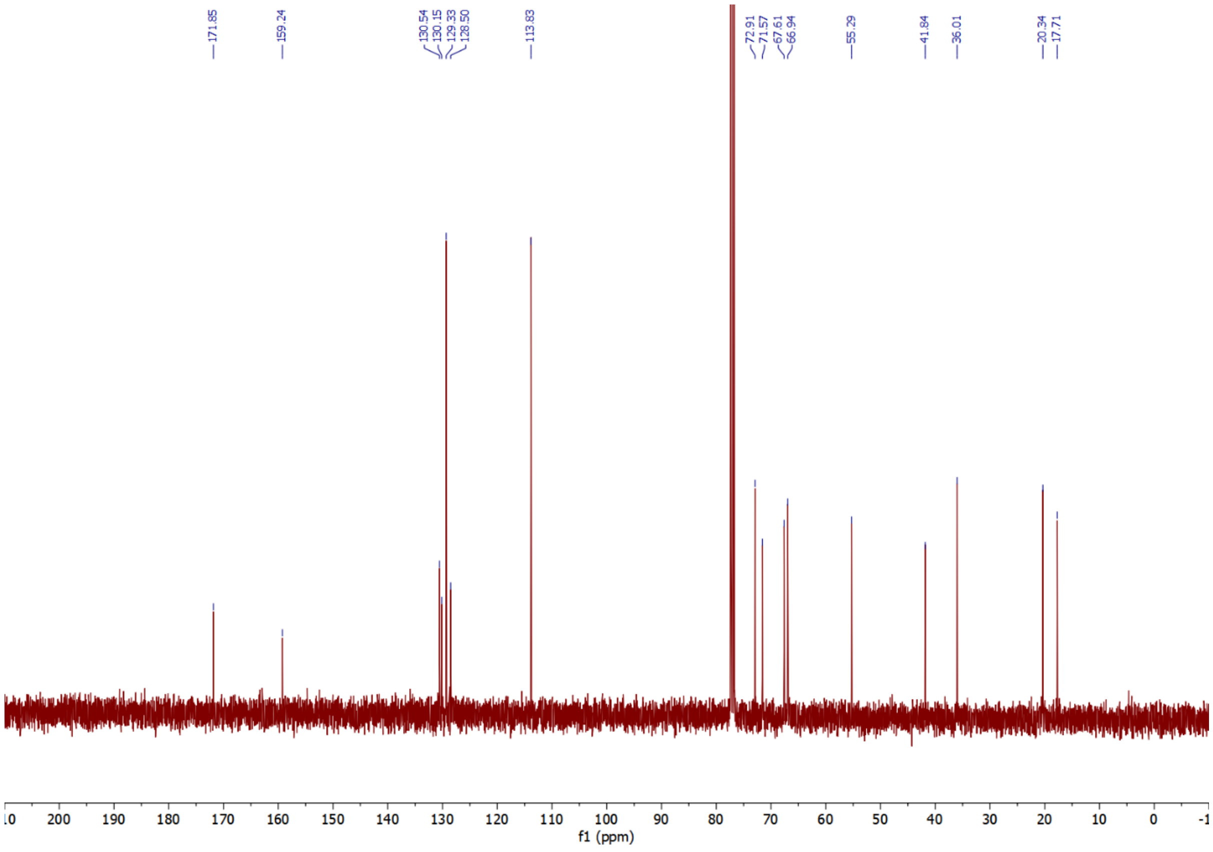

**(2*R*,3*R*,*E*)-Methyl 2-((*R*)-1-hydroxy-3-((4-methoxybenzyl)oxy)propyl)-3-methylhex-4-enoate, 13**

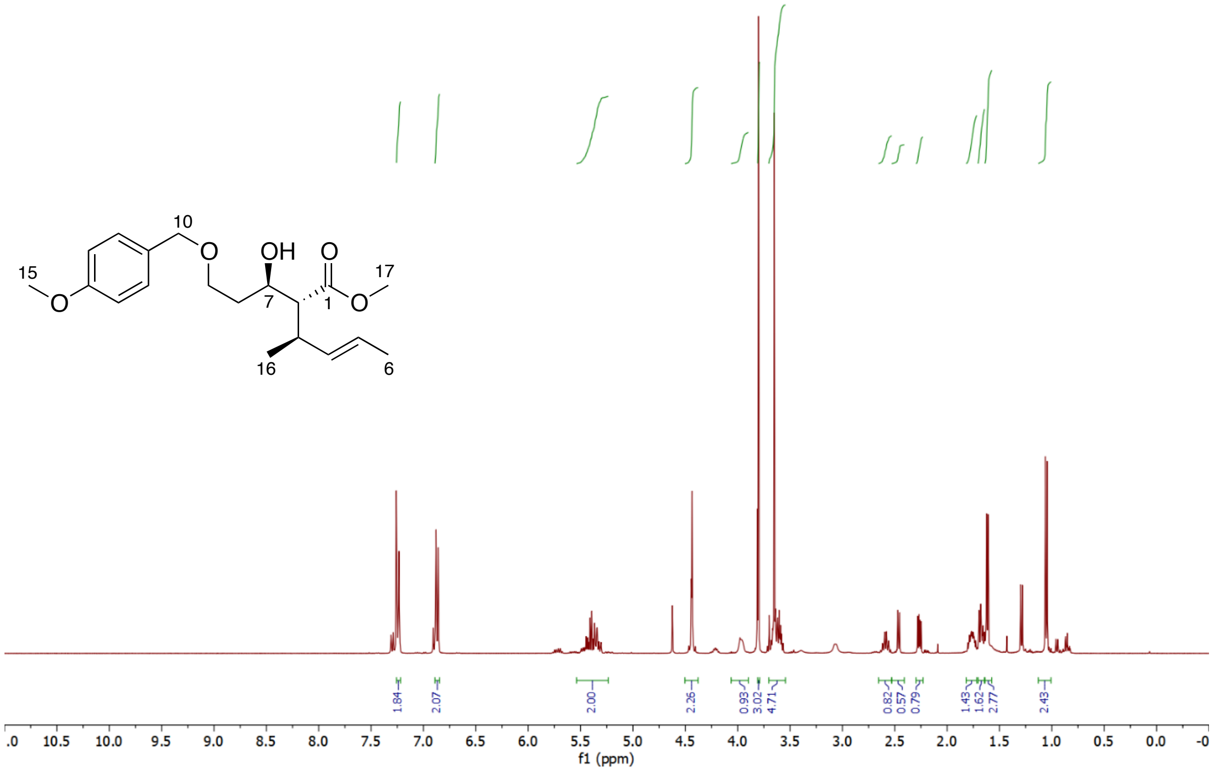


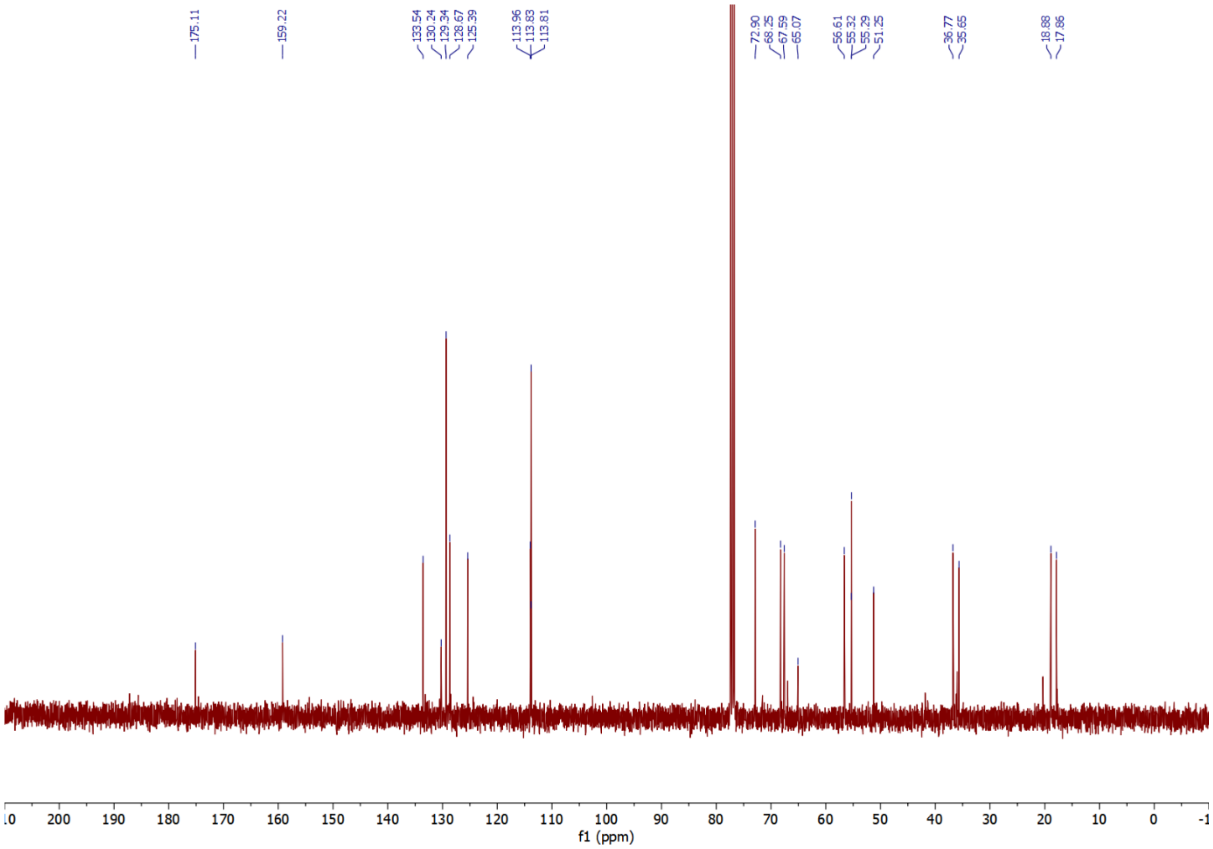

**(2*S*,3*R*)-5-((4-methoxybenzyl)oxy)-2-((*R*,*E*)-pent-3-en-2-yl)pentane-1,3-diol, S1**

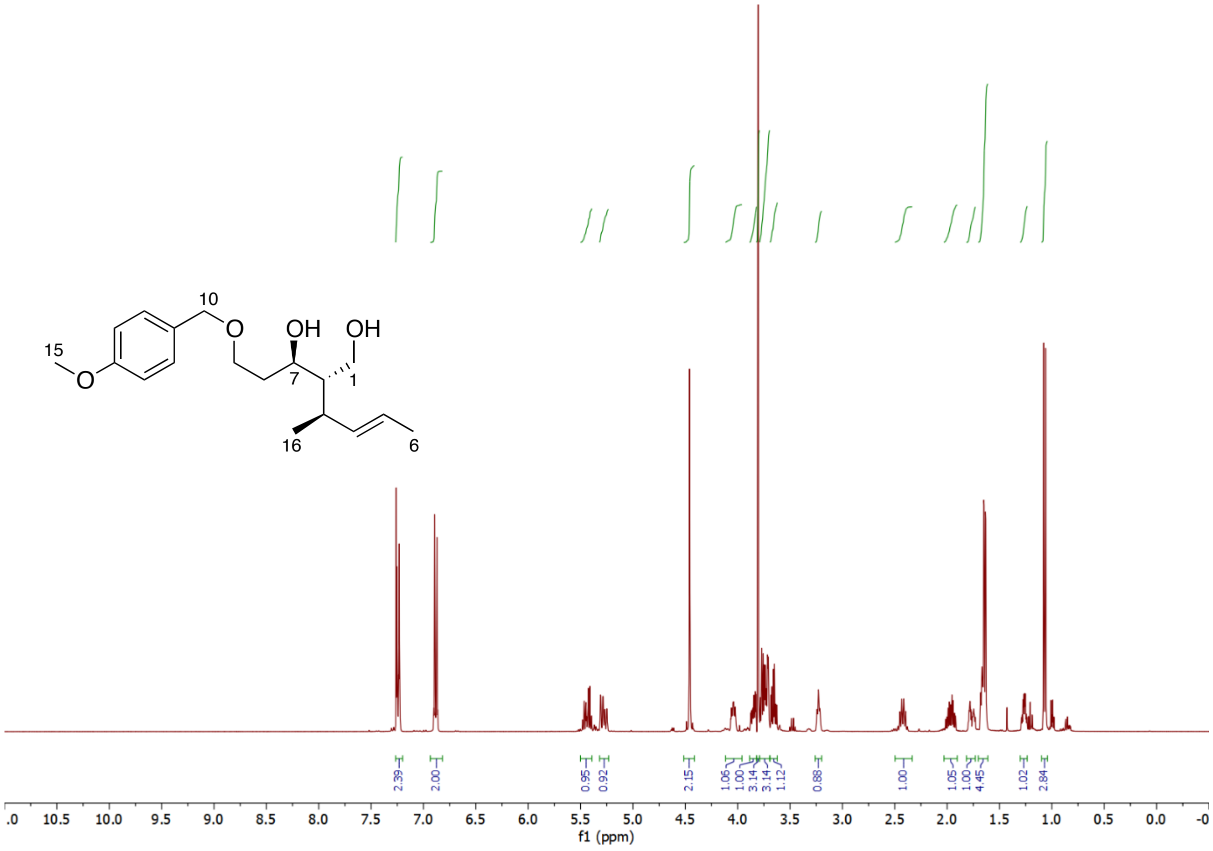


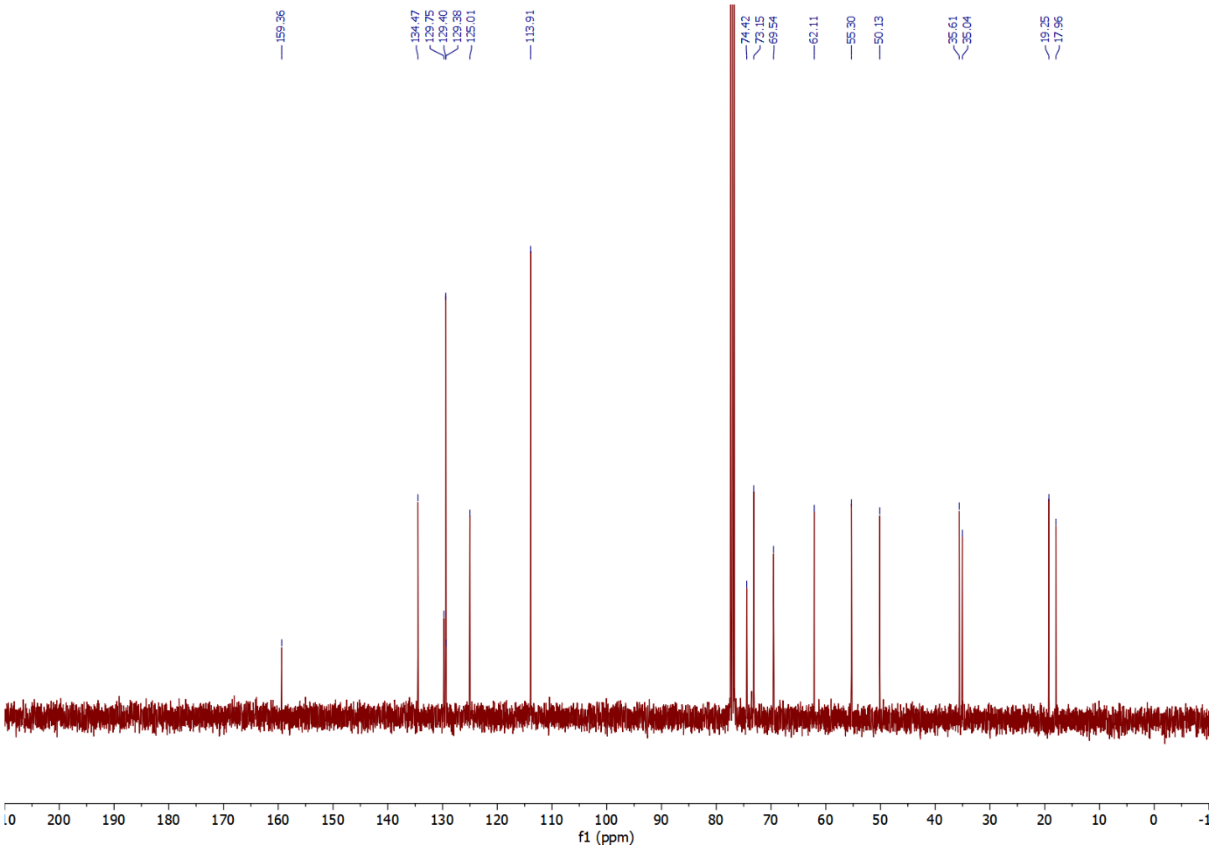

**(3*R*,4*S*,5*R*,*E*)-4-(((*tert*-butyldimethylsilyl)oxy)methyl)-1-((4-methoxybenzyl)oxy)-5-methyloct-6-en-3-ol, S2**

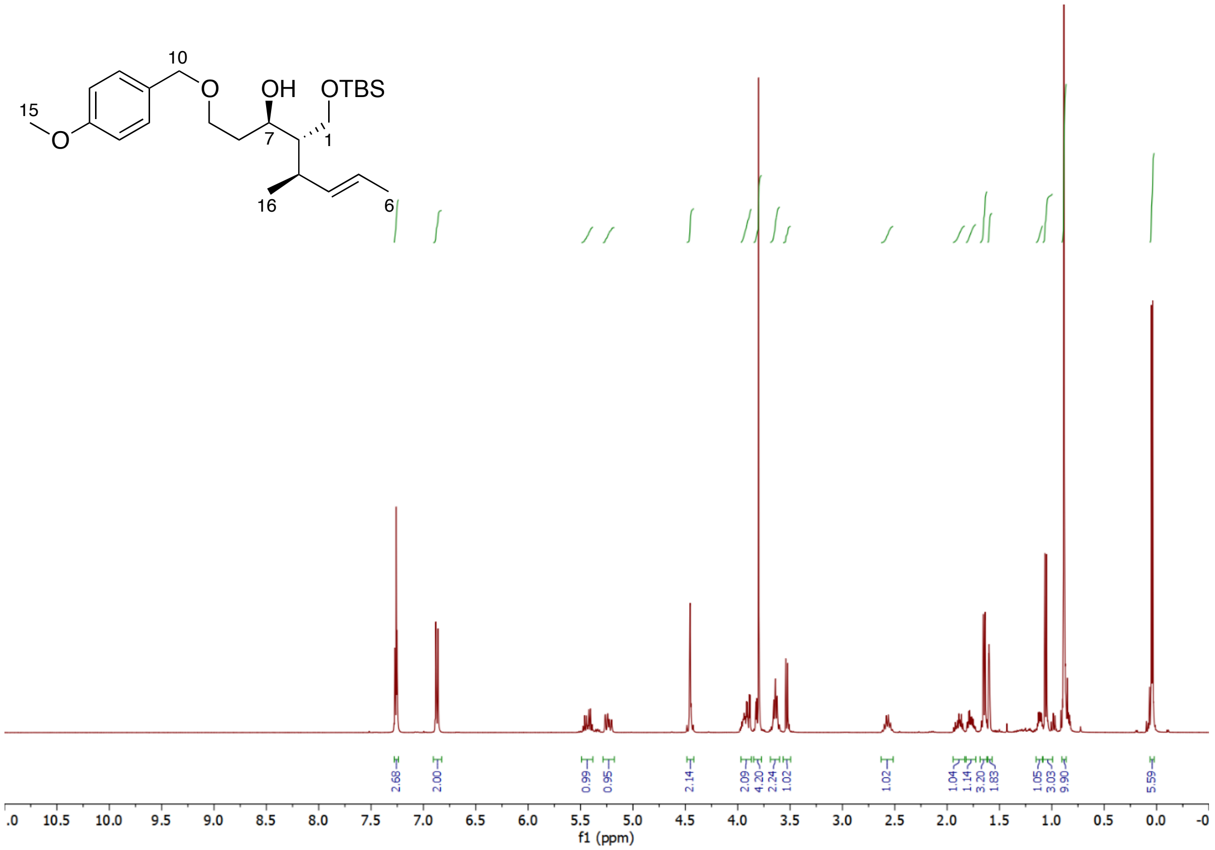


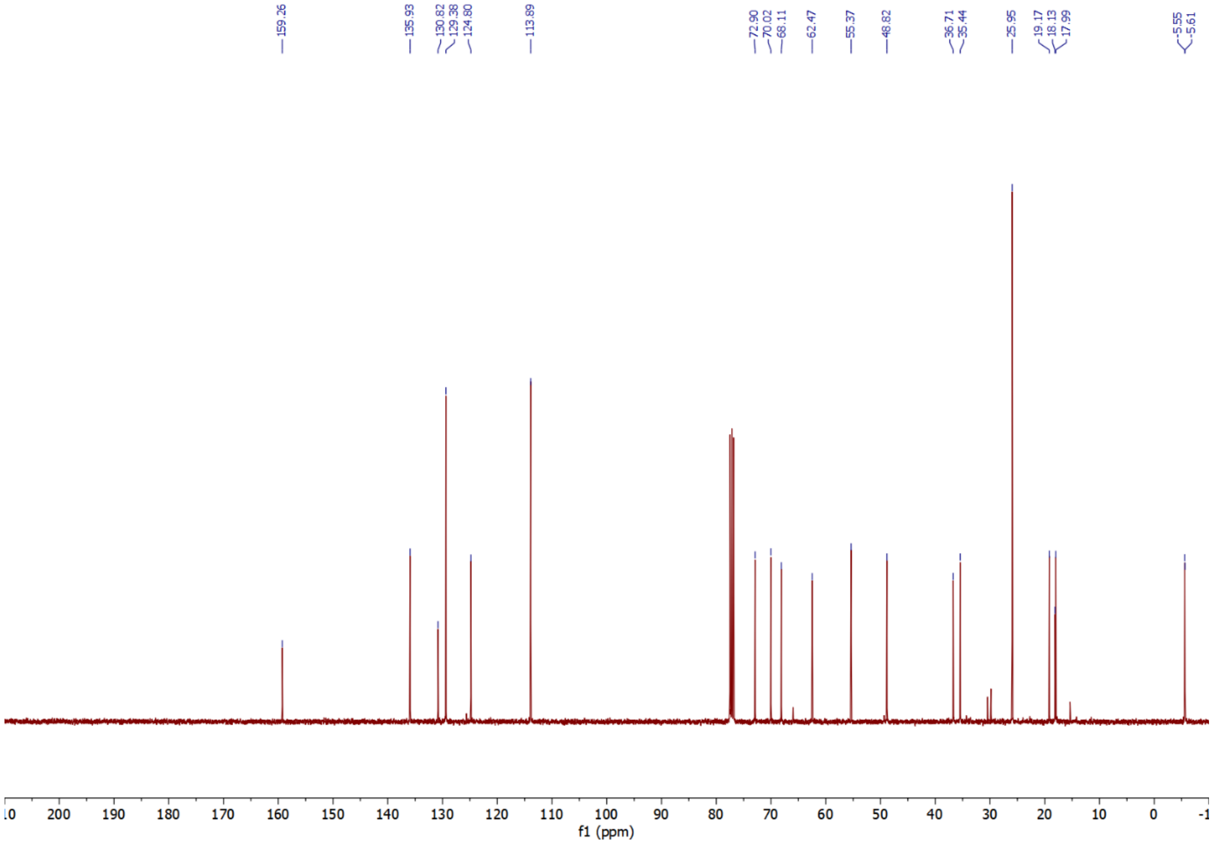

**(4*S*,5*S*,6*R*)-5-(((tert-butyldimethylsilyl)oxy)methyl)-8-((4-methoxybenzyl)oxy)-4-methyloctane-2,3,6-triol, S3**

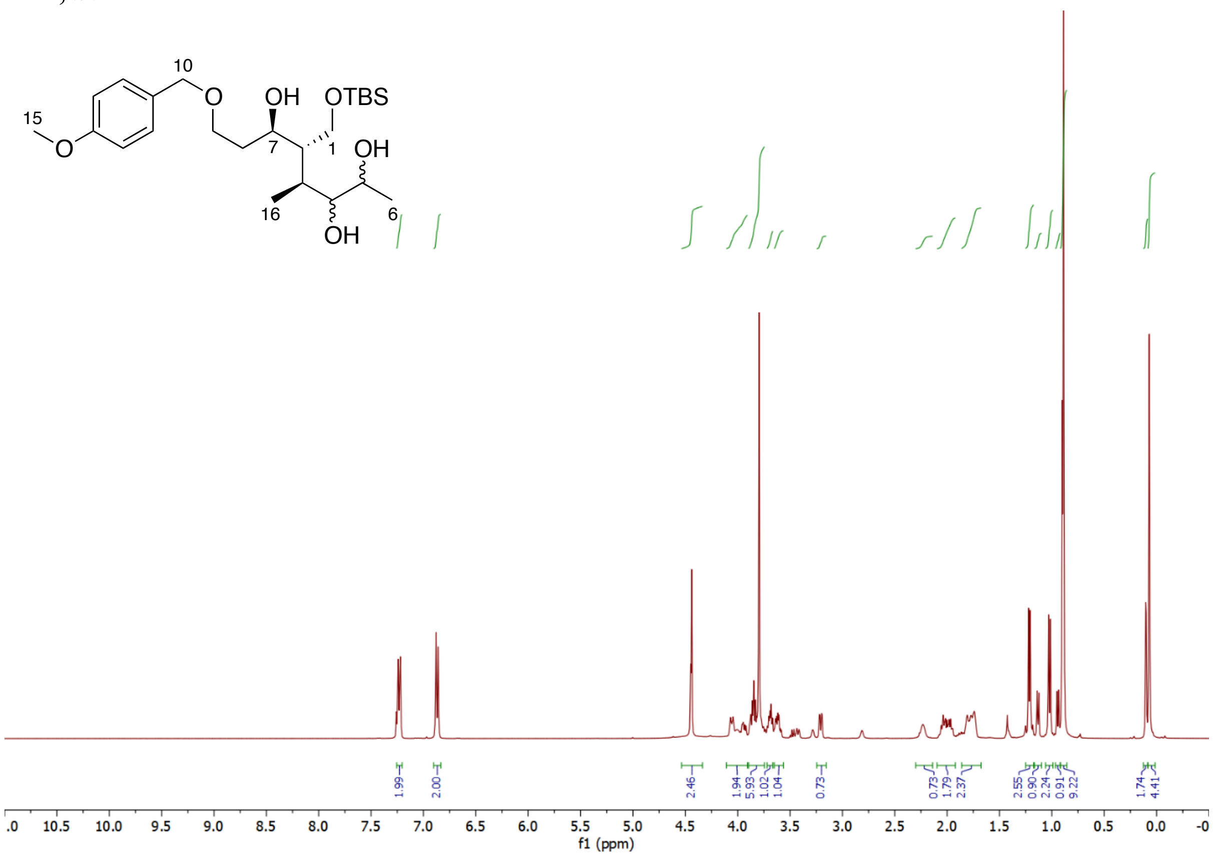


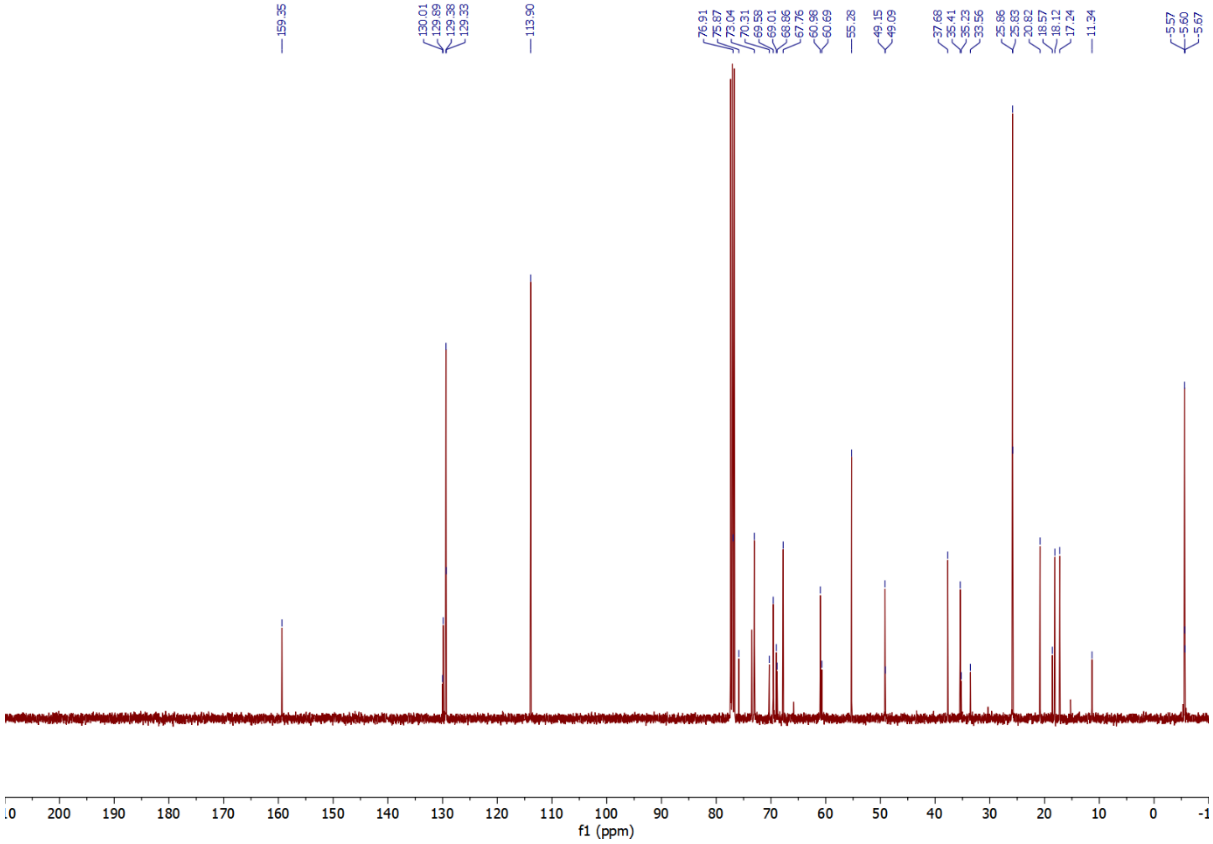

**(3*S*,4*S*,5*R*)-4-(((*Tert*-butyldimethylsilyl)oxy)methyl)-5-(2-((4-methoxybenzyl)oxy)ethyl)-3-methyltetrahydrofuran-2-ol, S4**

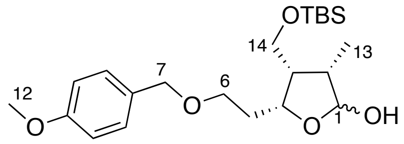


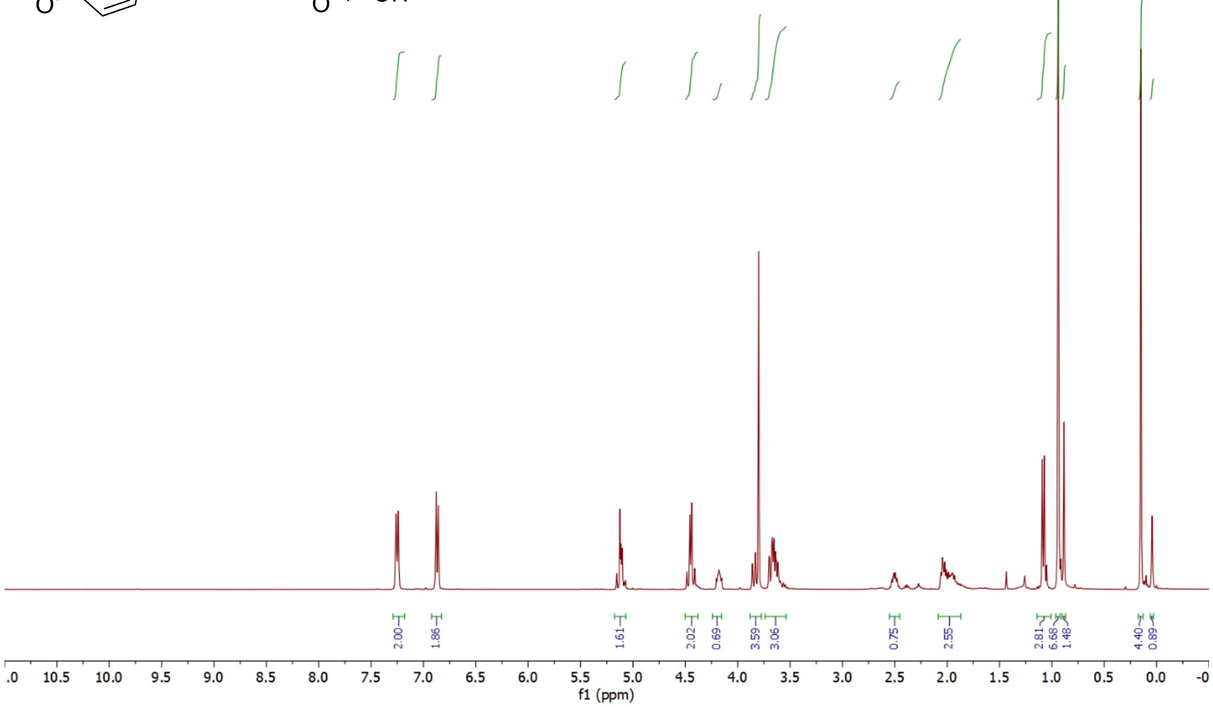


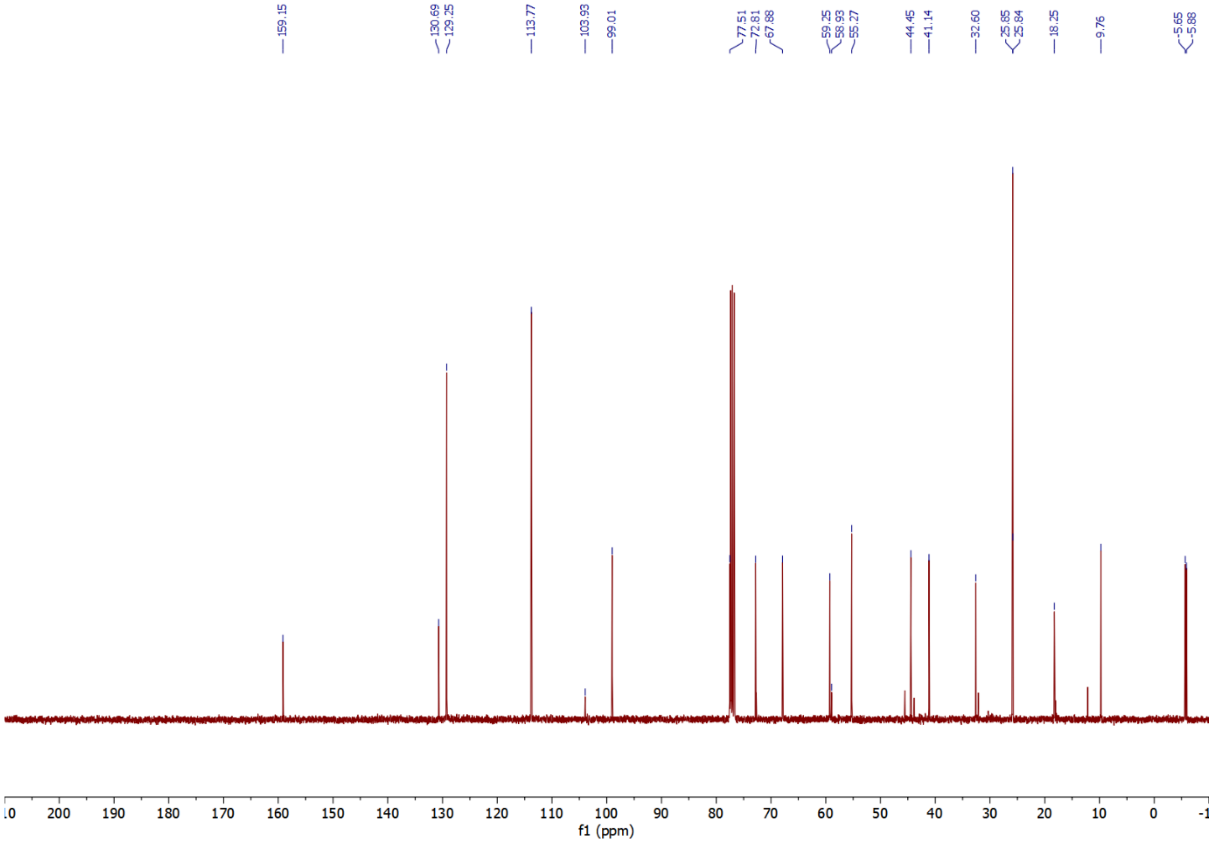

**((2*R*,3*S*,4*S*)-5-Methoxy-2-(2-((4-methoxybenzyl)oxy)ethyl)-4-methyltetrahydrofuran-3-yl) methanol, 14**

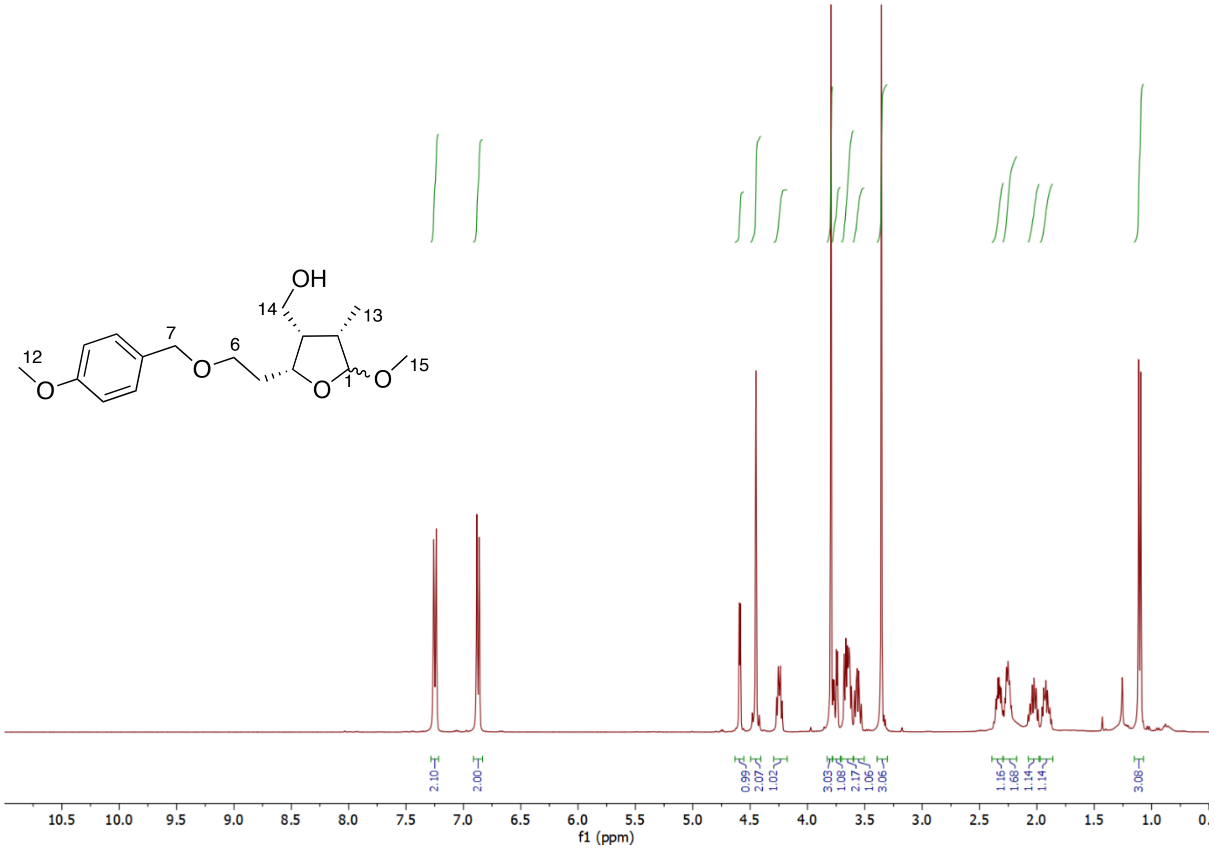


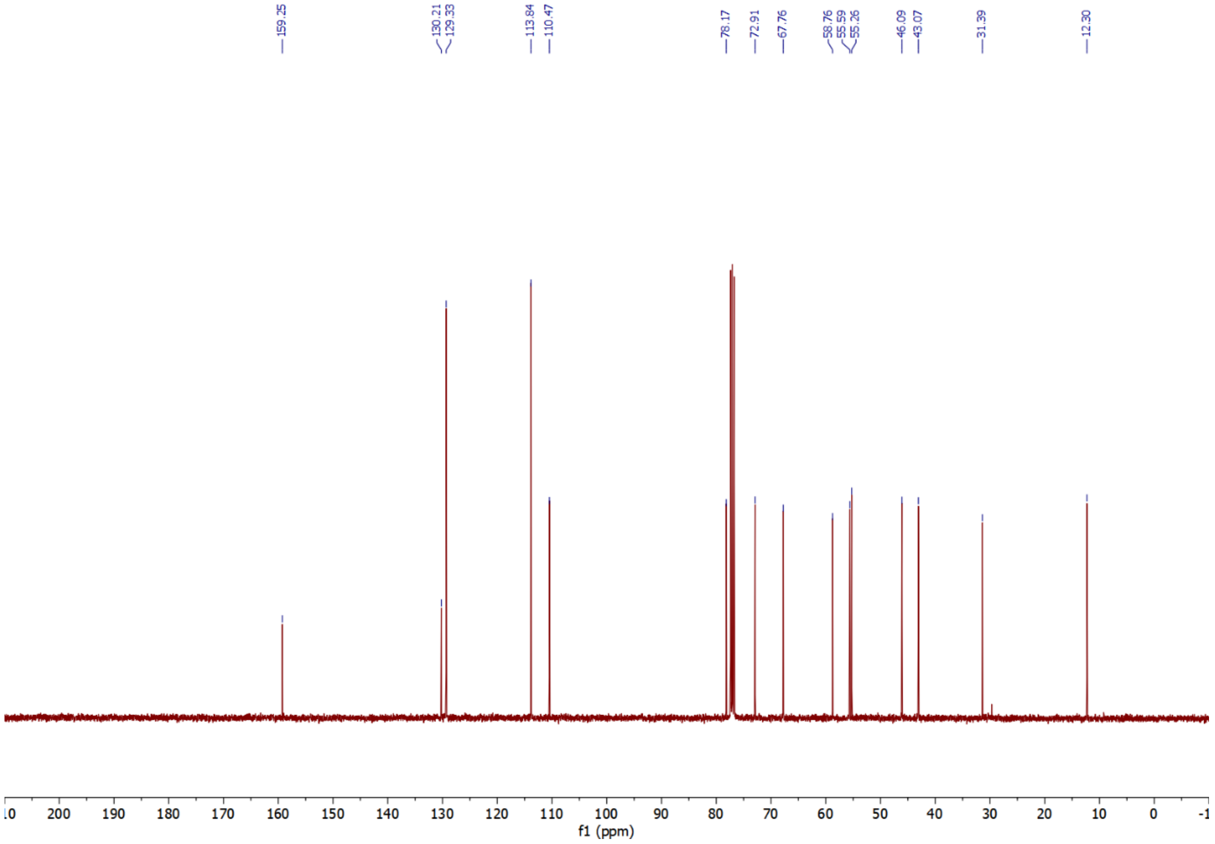

**(((2*R*,3*S*,4*S*)-5-Methoxy-2-(2-((4-methoxybenzyl)oxy)ethyl)-4-methyltetrahydrofuran-3-yl)ethynyl)trimethylsilane, S5**

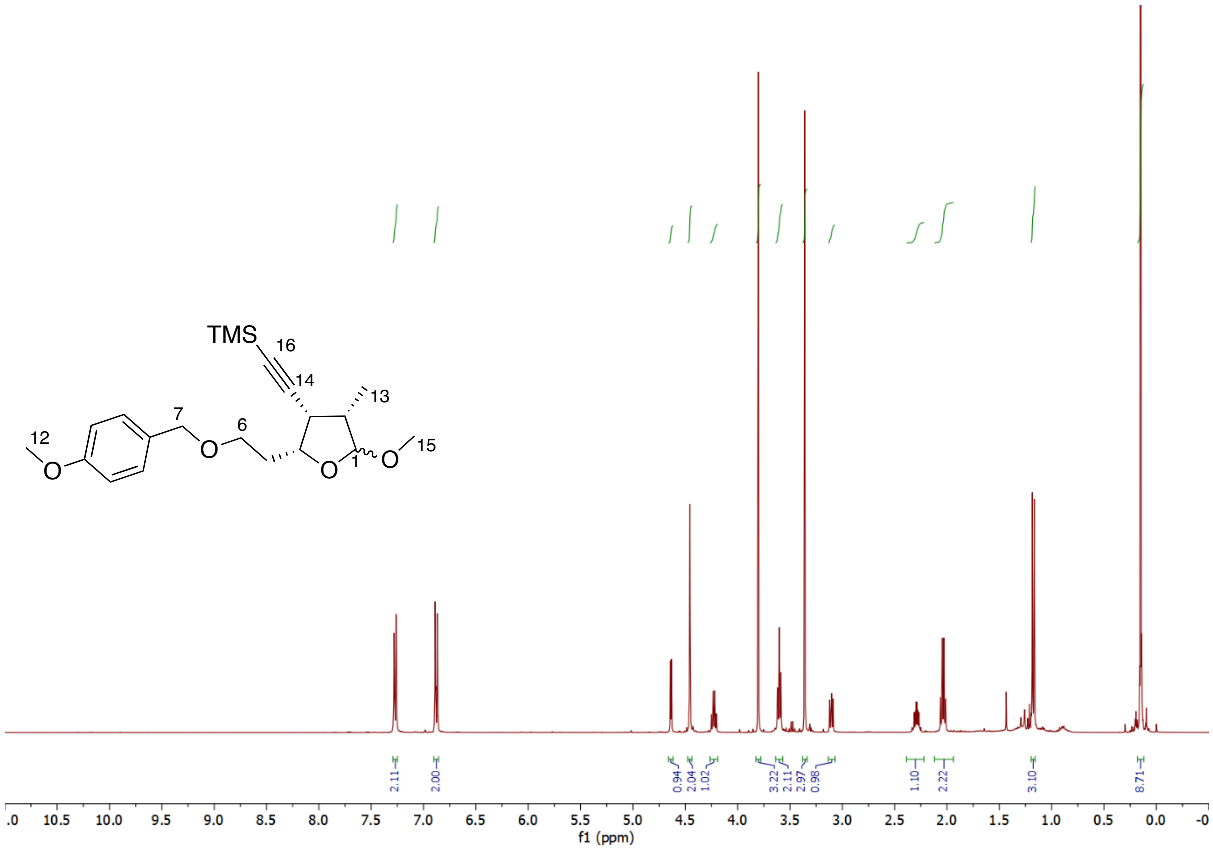


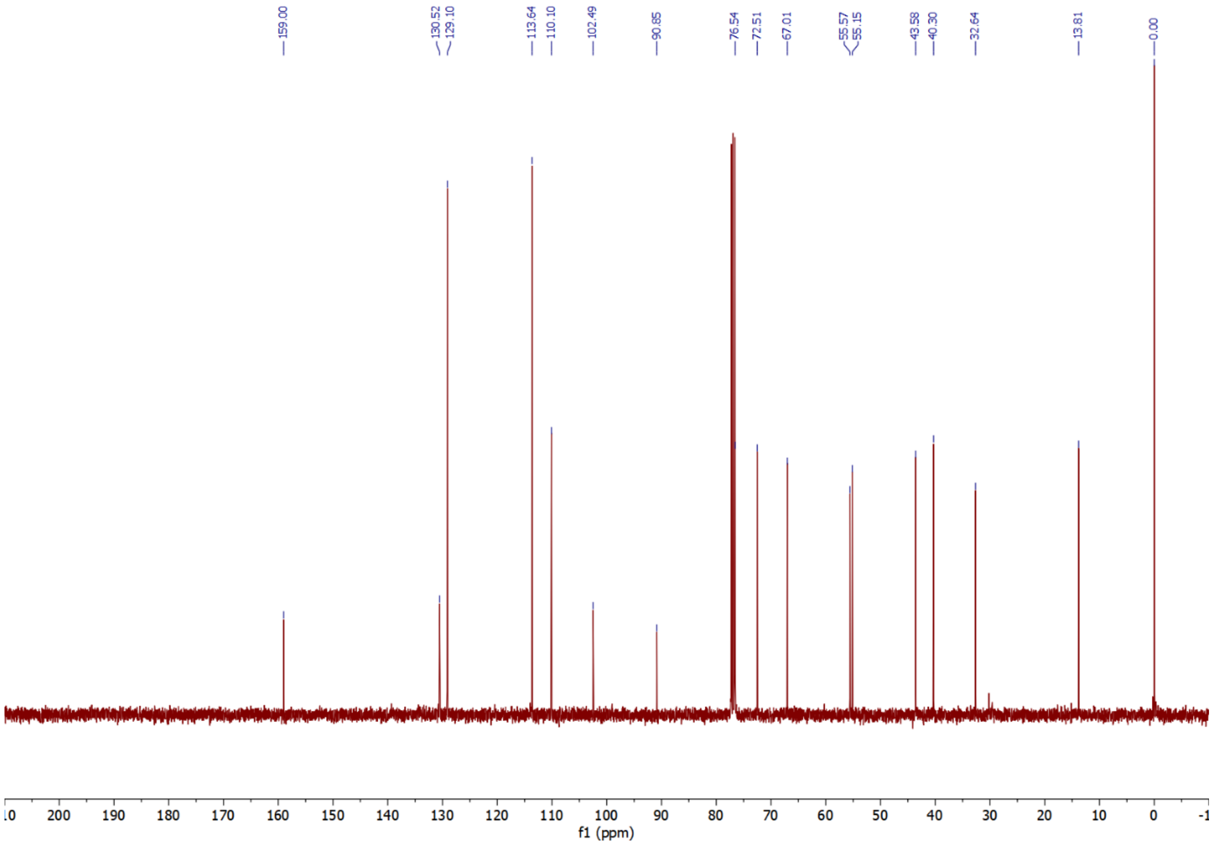

**(((2*R*,3*S*,4*S*)-2-(3,3-dibromoallyl)-5-methoxy-4-methyltetrahydrofuran-3-yl)ethynyl) trimethylsilane, S6**

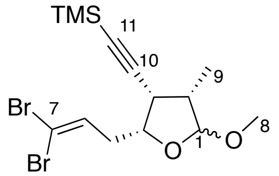


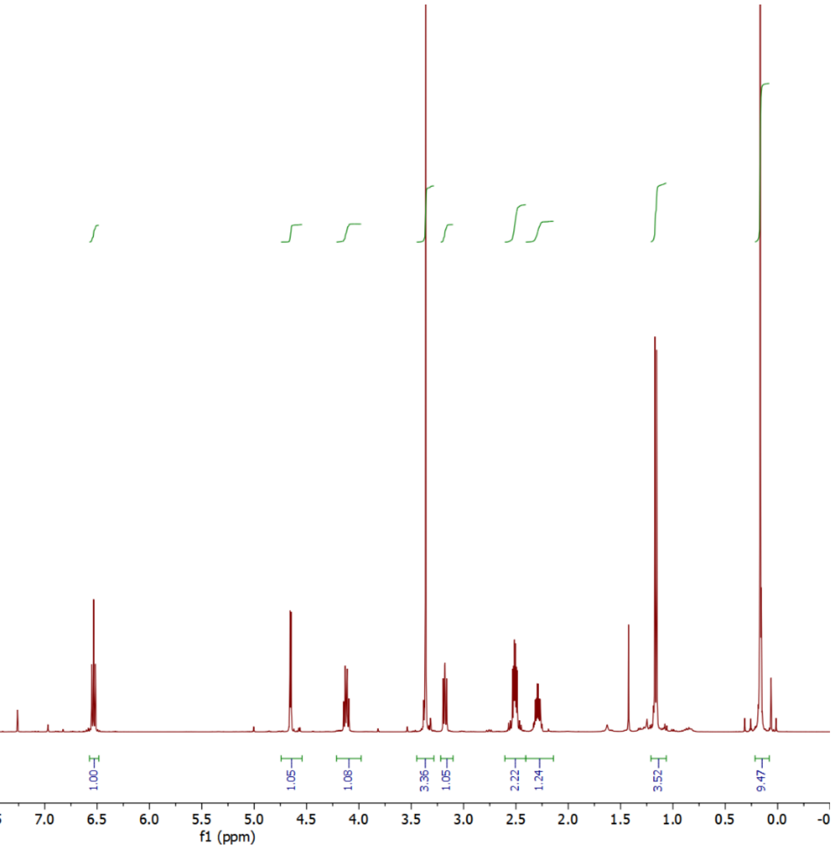


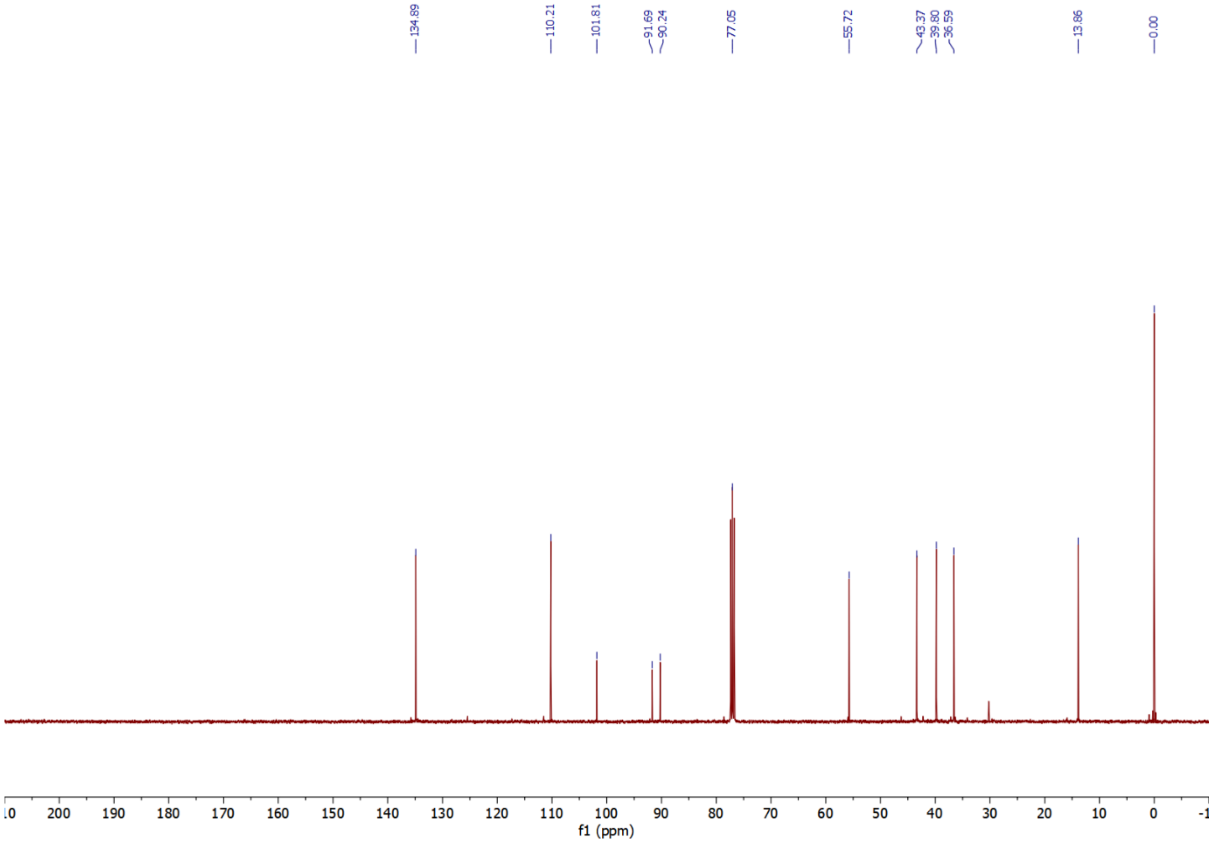

**(((2*R*,3*S*,4*S*)-5-methoxy-4-methyl-2-(prop-2-yn-1-yl)tetrahydrofuran-3-yl)ethynyl)trimethylsilane, 15**

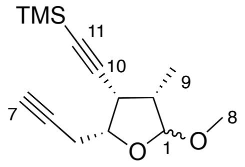


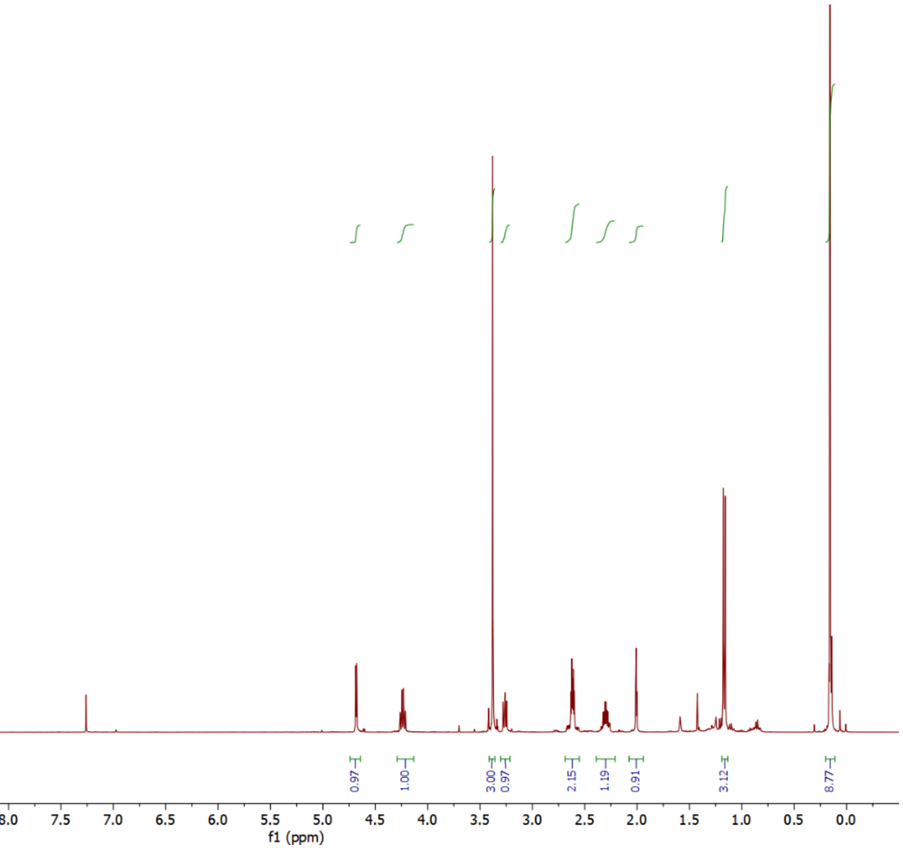


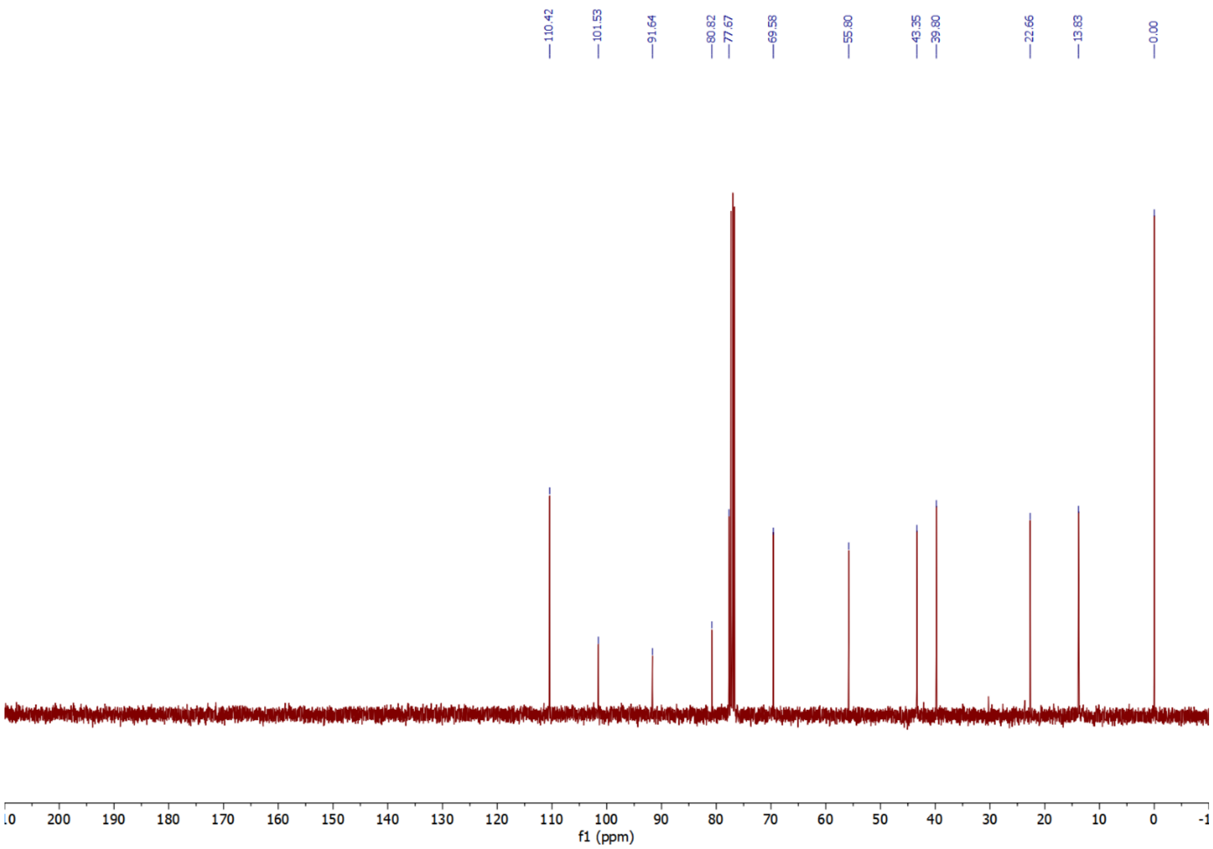

**(3*S*,4*S*,5*R*)-3-methyl-5-(prop-2-yn-1-yl)-4-((trimethylsilyl)ethynyl)tetrahydrofuran-2-yl acetate, 16**

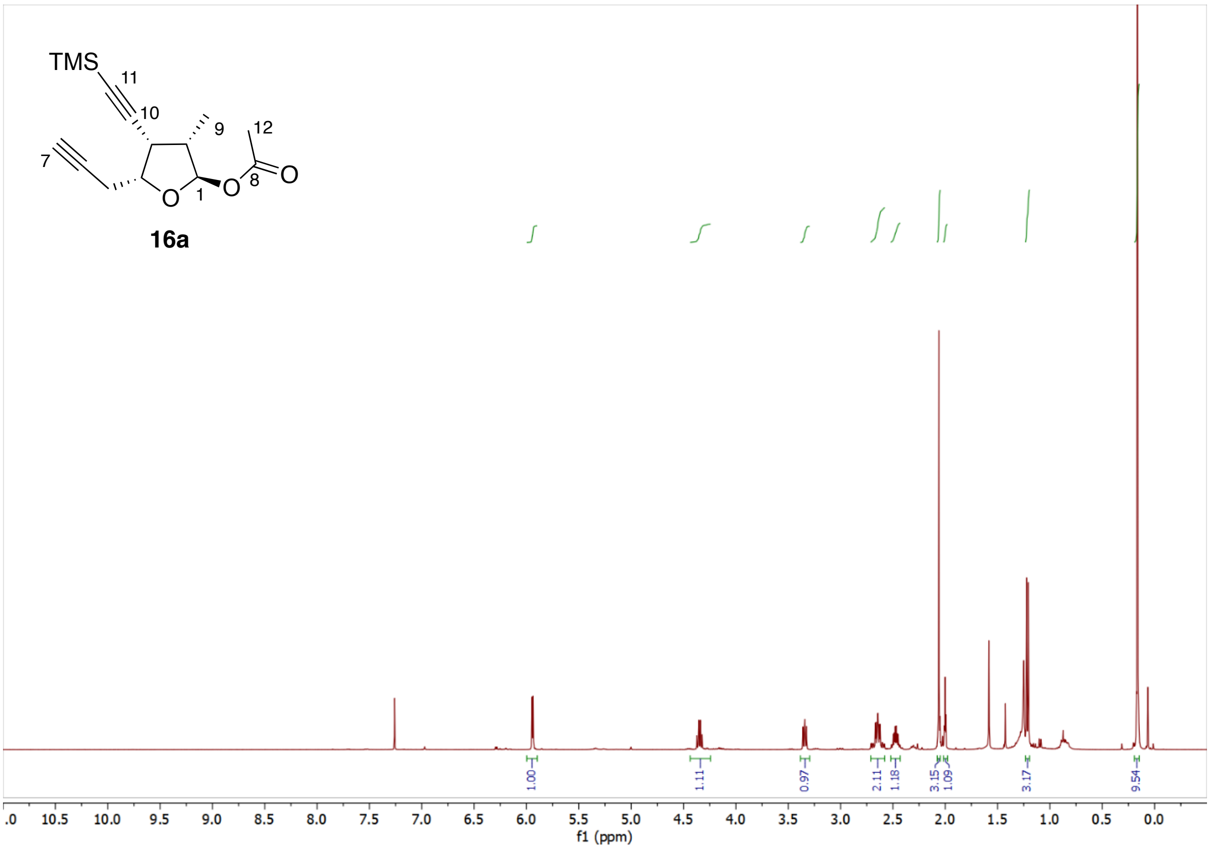


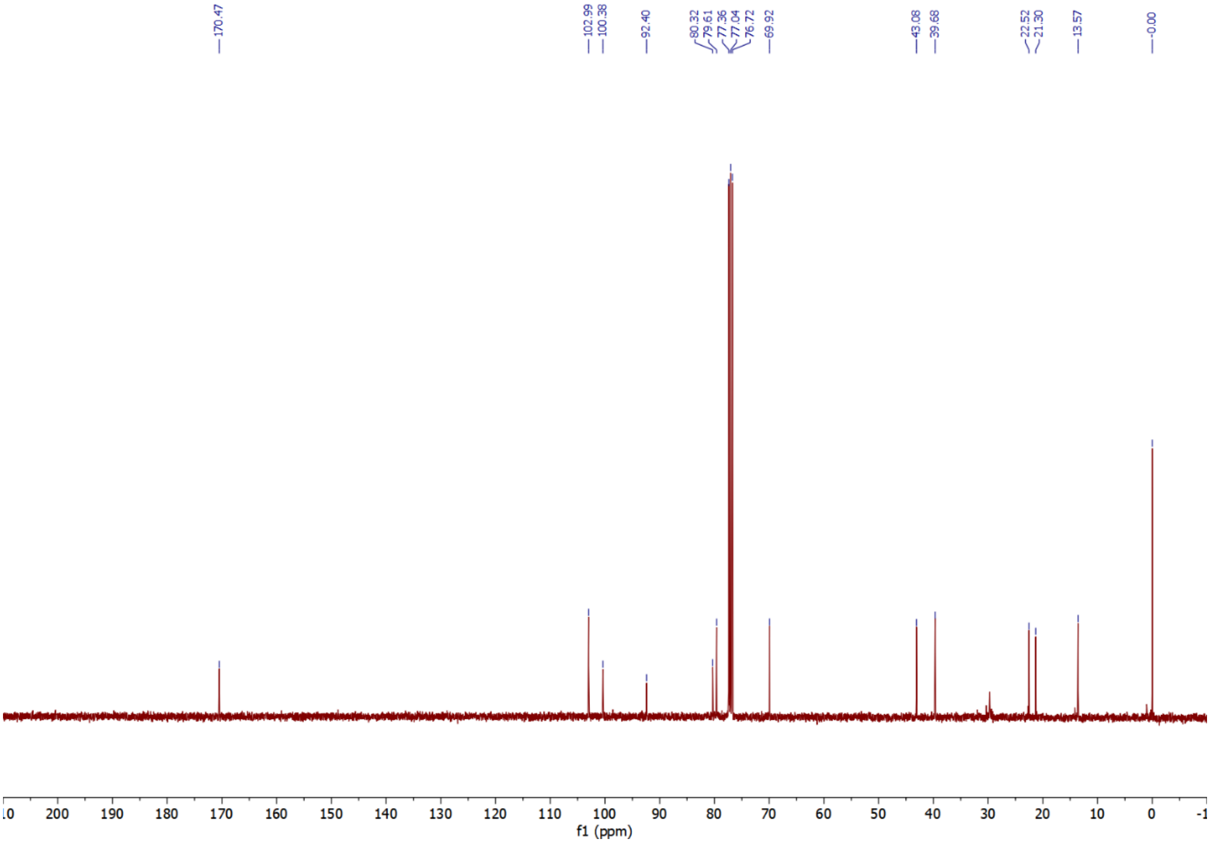

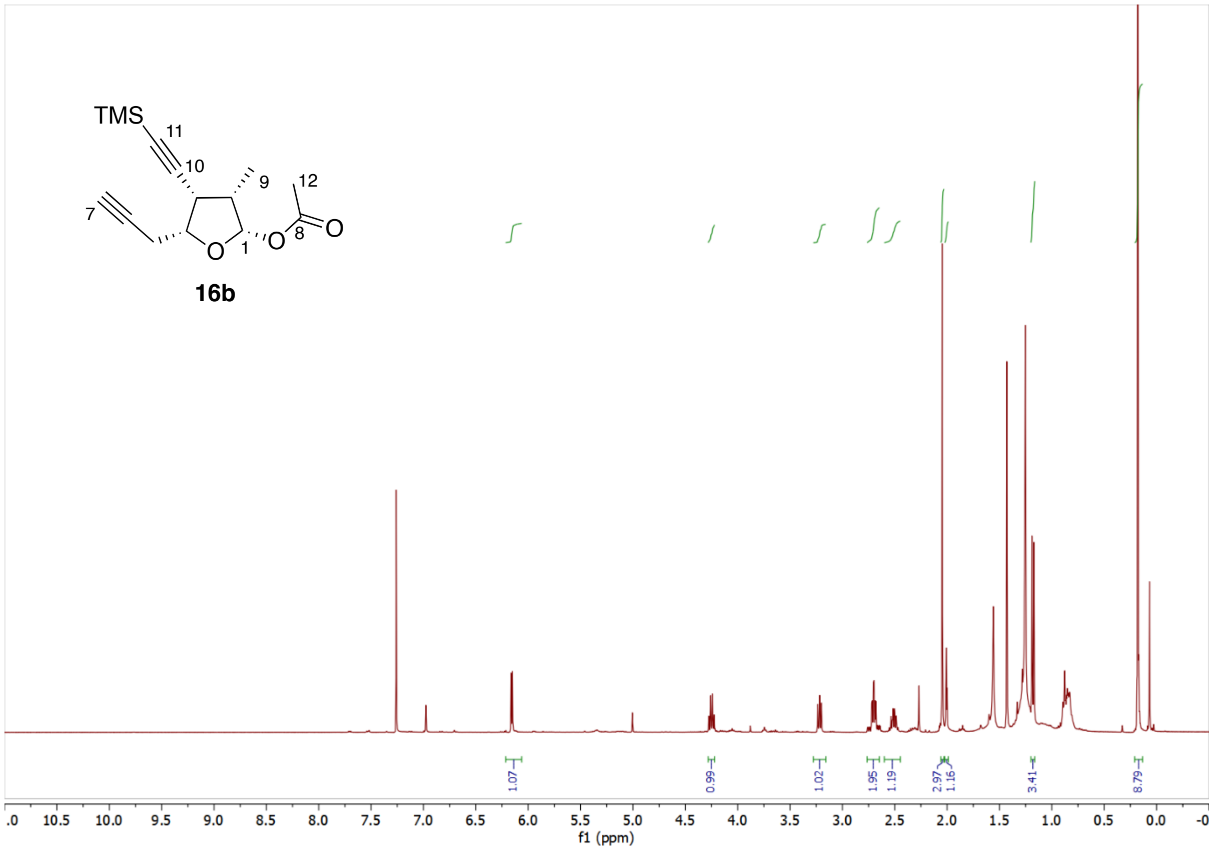
TMS
11
10
9
12
7
8
O
1
O
O
16b
1.07
0.99
1.02
1.95
1.19
2.97
1.16
3.41
8.79
f1 (ppm)

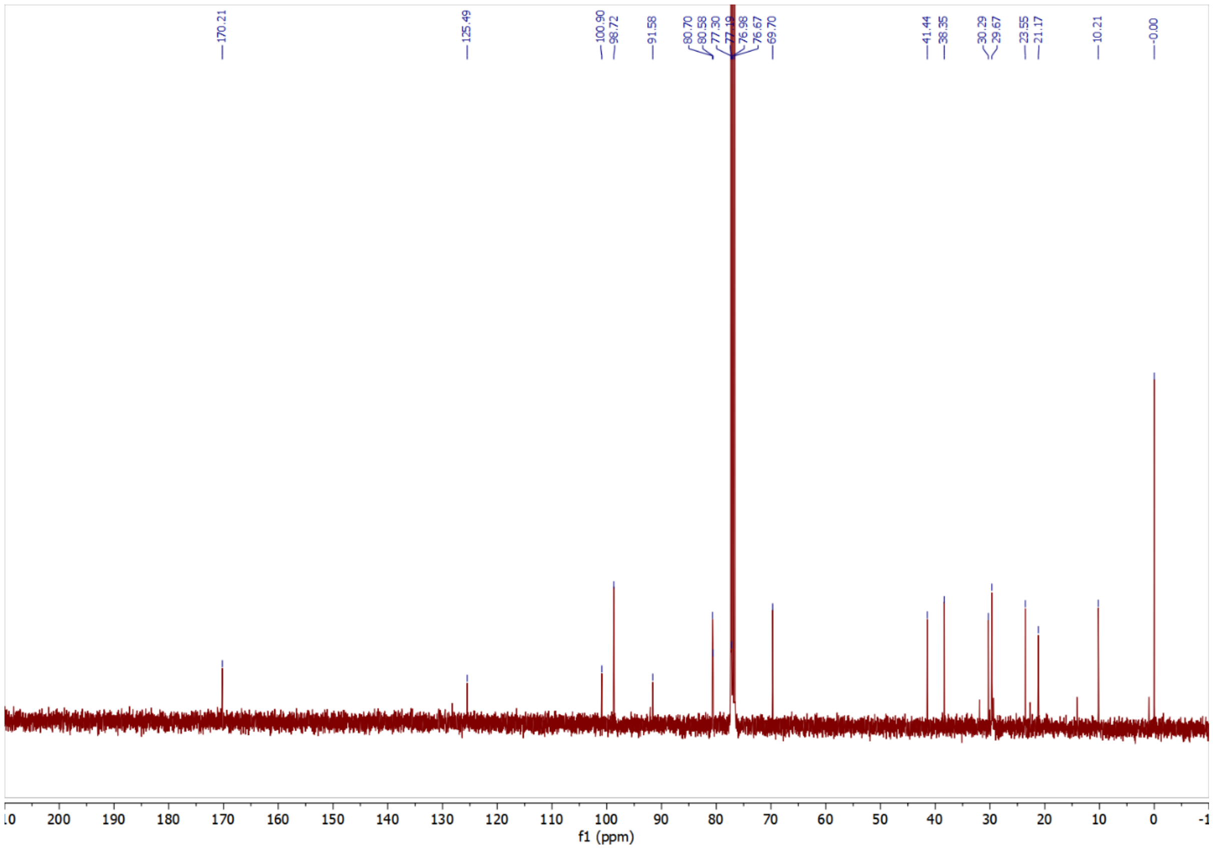
170.21
125.49
100.90
98.72
91.58
80.70
80.58
77.30
76.98
76.67
69.70
41.44
38.35
30.29
29.67
23.55
21.17
10.21
0.00
f1 (ppm)

**(3*S*,4*S*,5*R*)-5-(3-Iodoprop-2-yn-1-yl)-3-methyl-4-((trimethylsilyl)ethynyl)tetrahydrofuran-2-yl acetate, 17**

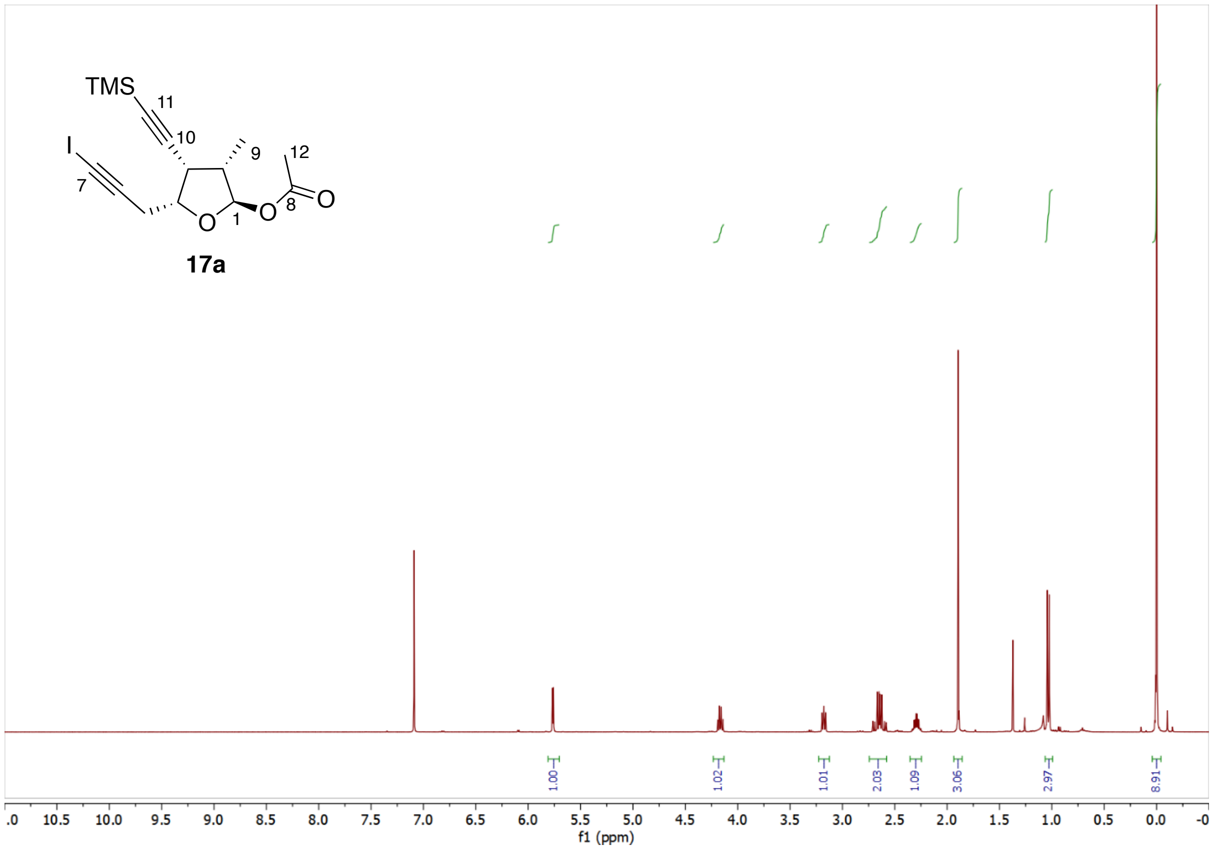


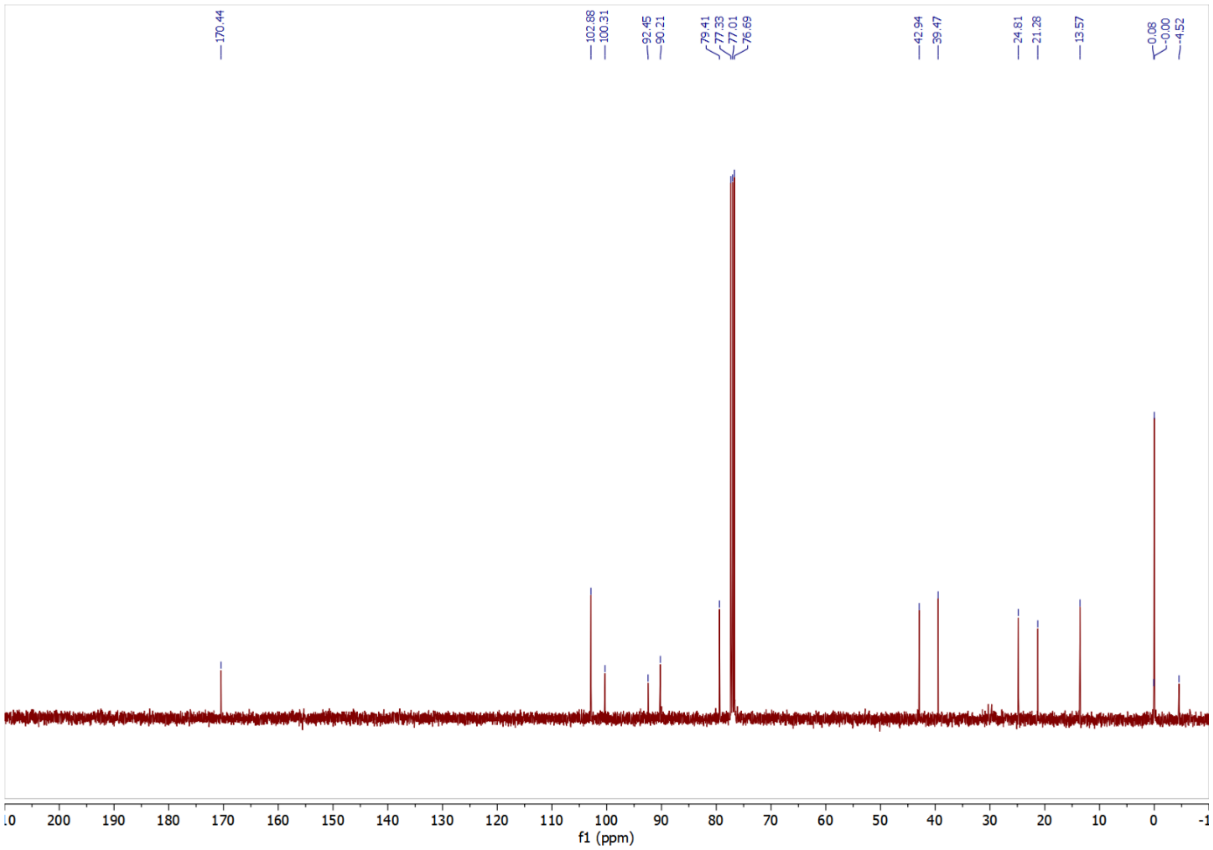

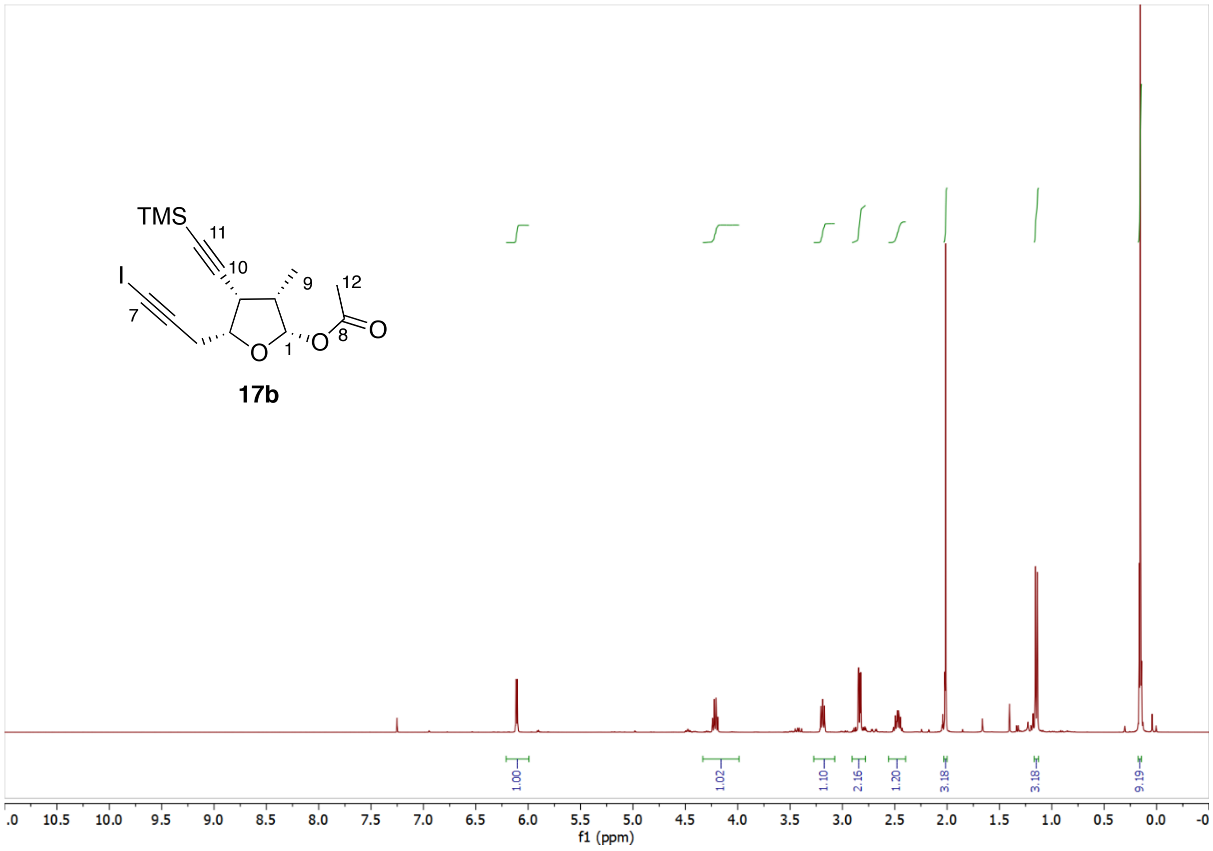
TMS
11
10
I
9
12
7
8
O
1
O
O
17b
1.00
1.02
1.10
2.16
1.20
3.18
3.18
9.19
f1 (ppm)

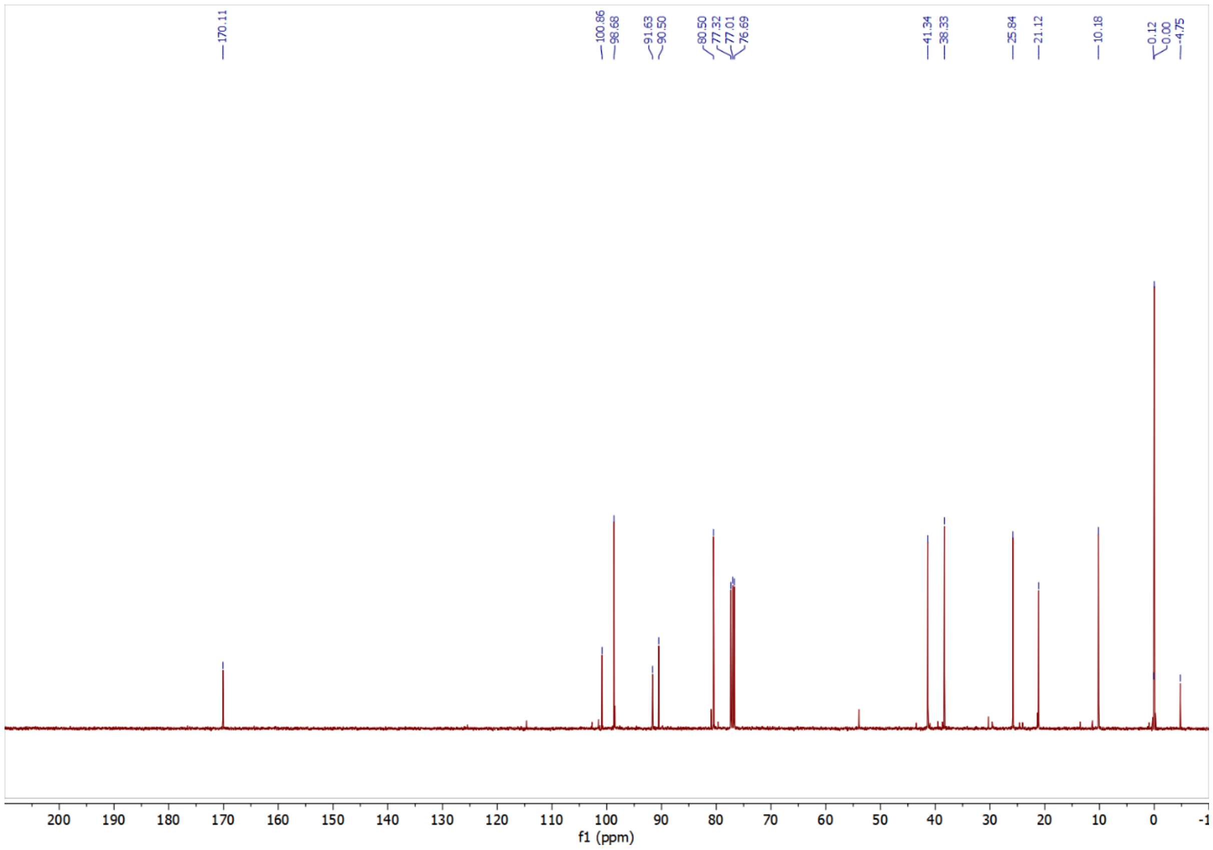
170.11
100.86
98.68
91.63
90.50
80.50
77.32
77.01
76.69
41.34
38.33
25.84
21.12
10.18
0.12
0.00
-4.75
f1 (ppm)

**(*S*)-5-((2*S*,3*S*,4*S*,5*R*)-5-(3-iodoprop-2-yn-1-yl)-3-methyl-4-((trimethylsilyl)ethynyl)tetrahydro-furan-2-yl)-3-methylfuran-2(5*H*)-one, 7a**

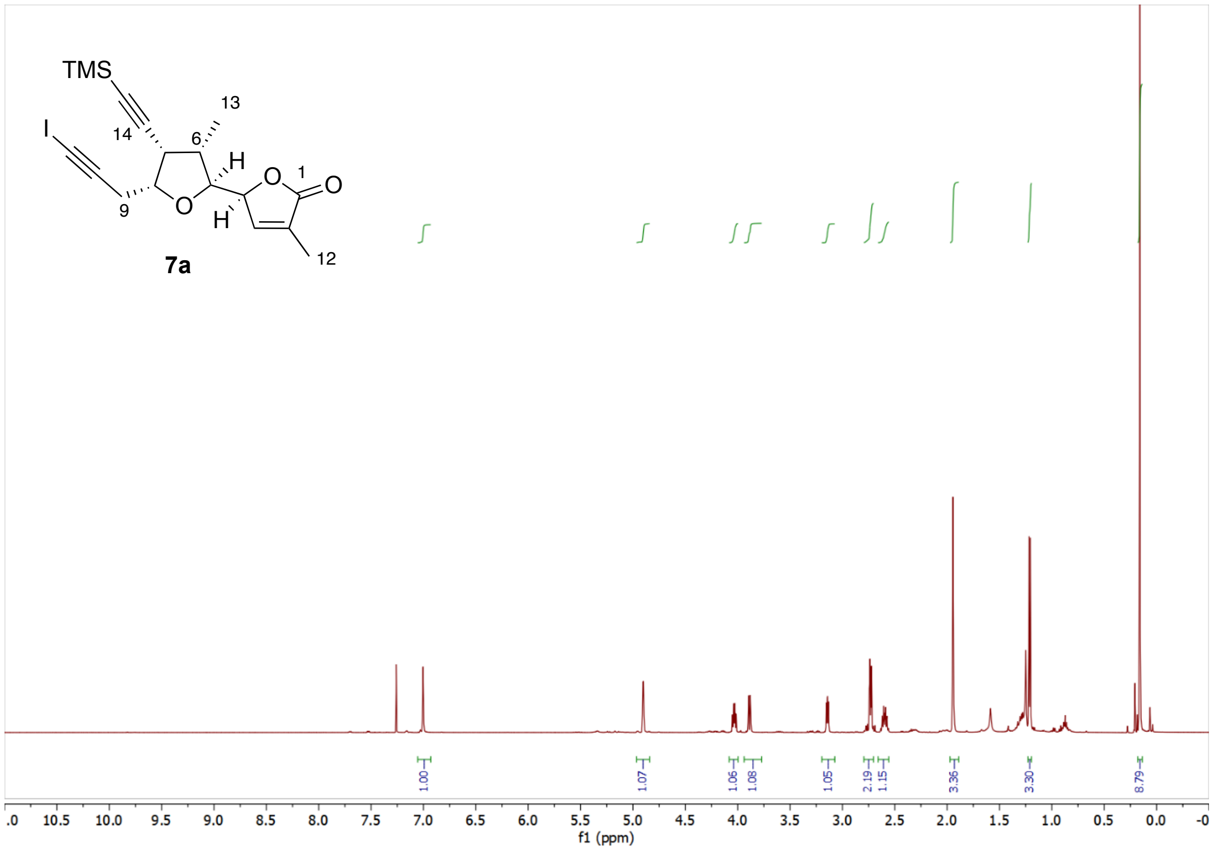


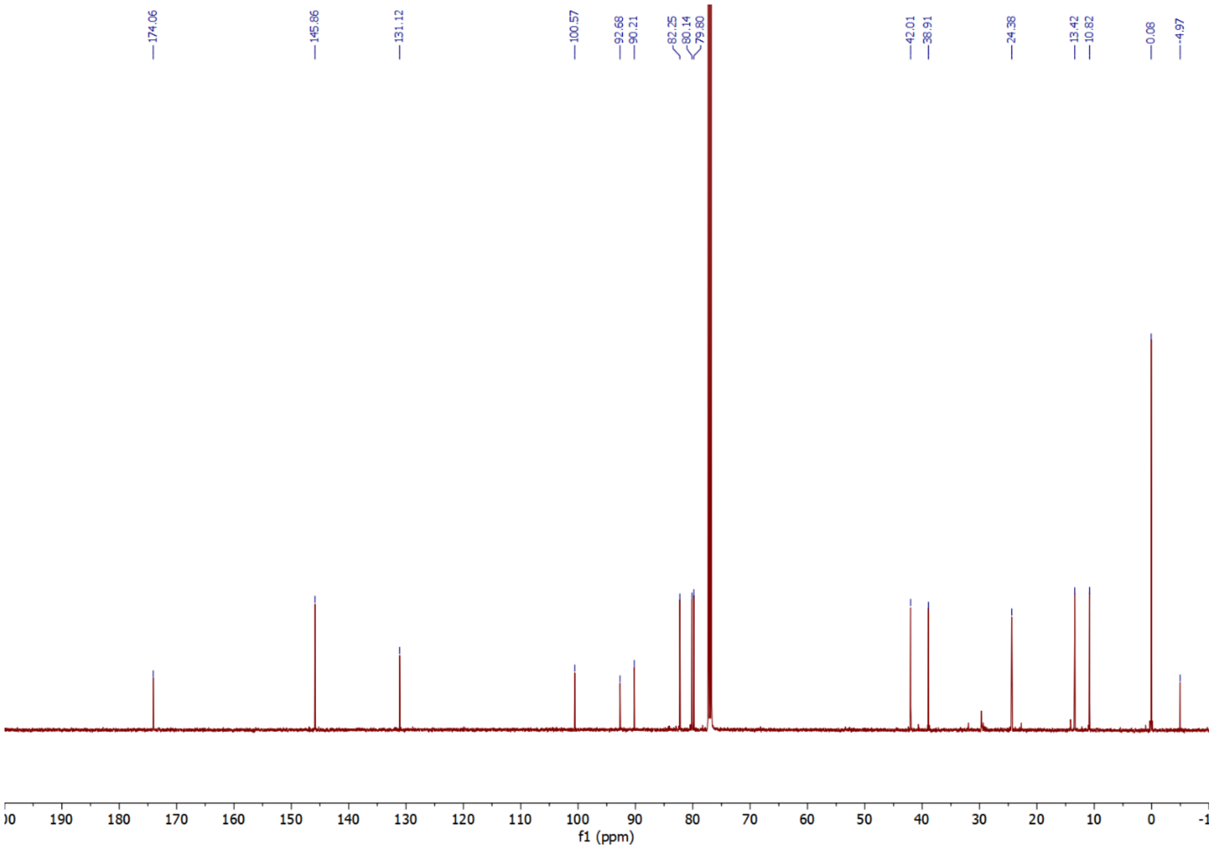

**(*R*)-5-((2*S*,3*S*,4*S*,5*R*)-5-(3-iodoprop-2-yn-1-yl)-3-methyl-4-((trimethylsilyl)ethynyl)tetrahydro-furan-2-yl)-3-methylfuran-2(5*H*)-one, 7b**

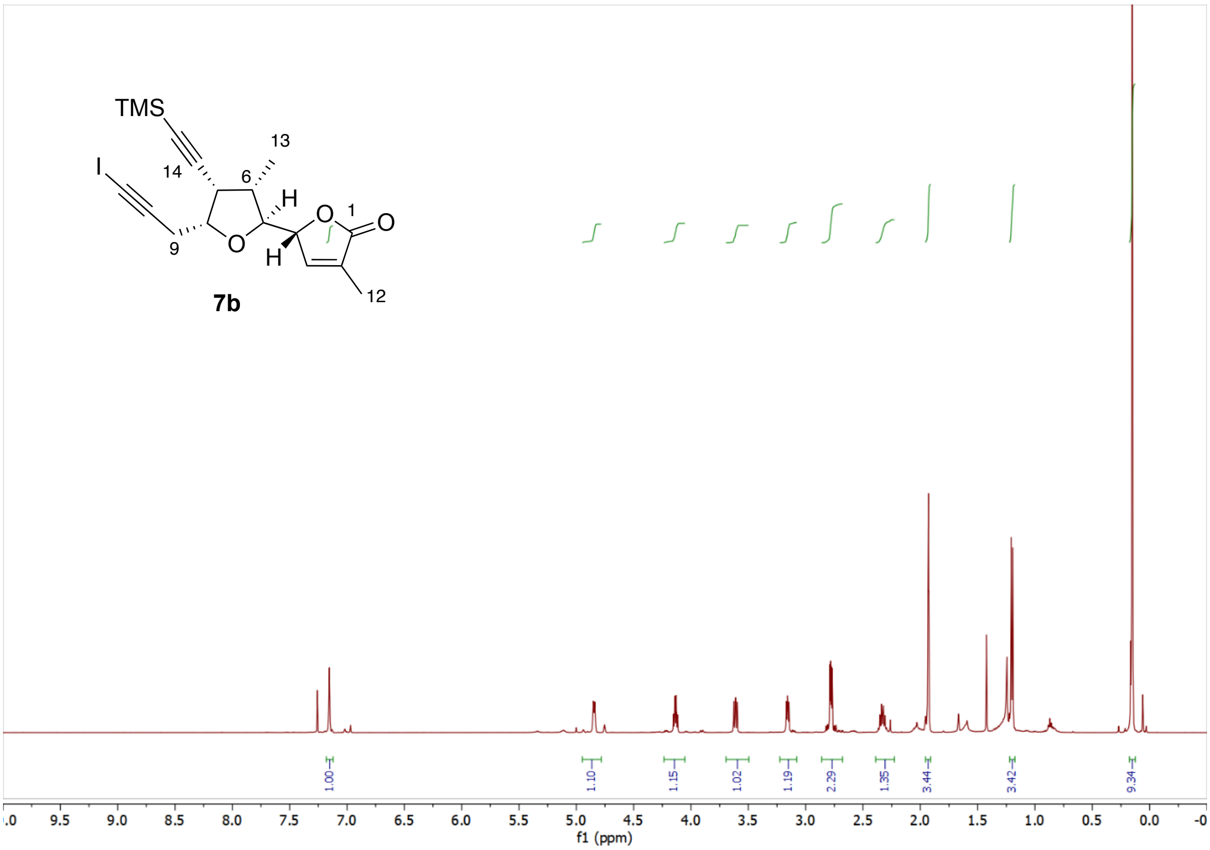


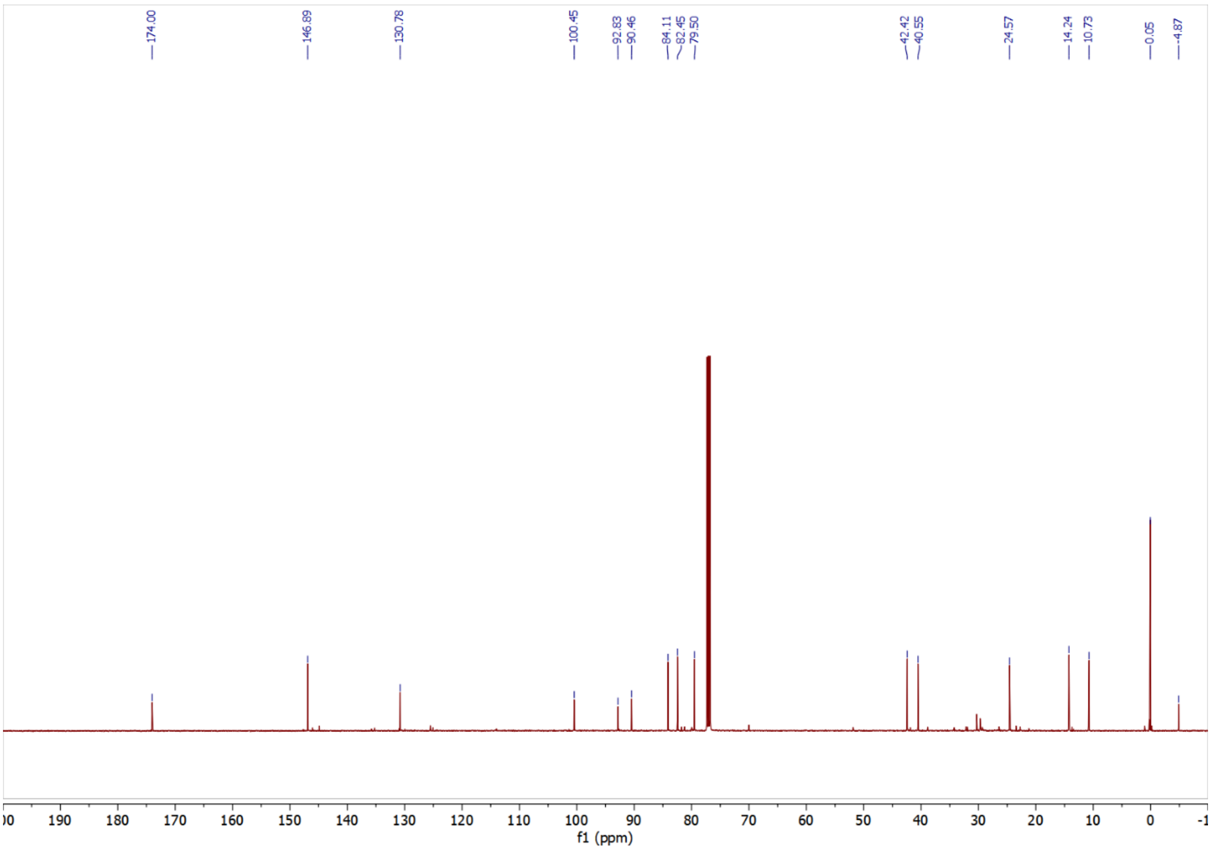

**Ethyl (*S*)-3-hydroxy-5-((4-methoxybenzyl)oxy)pentanoate: 19**

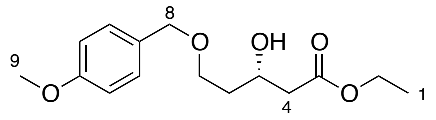


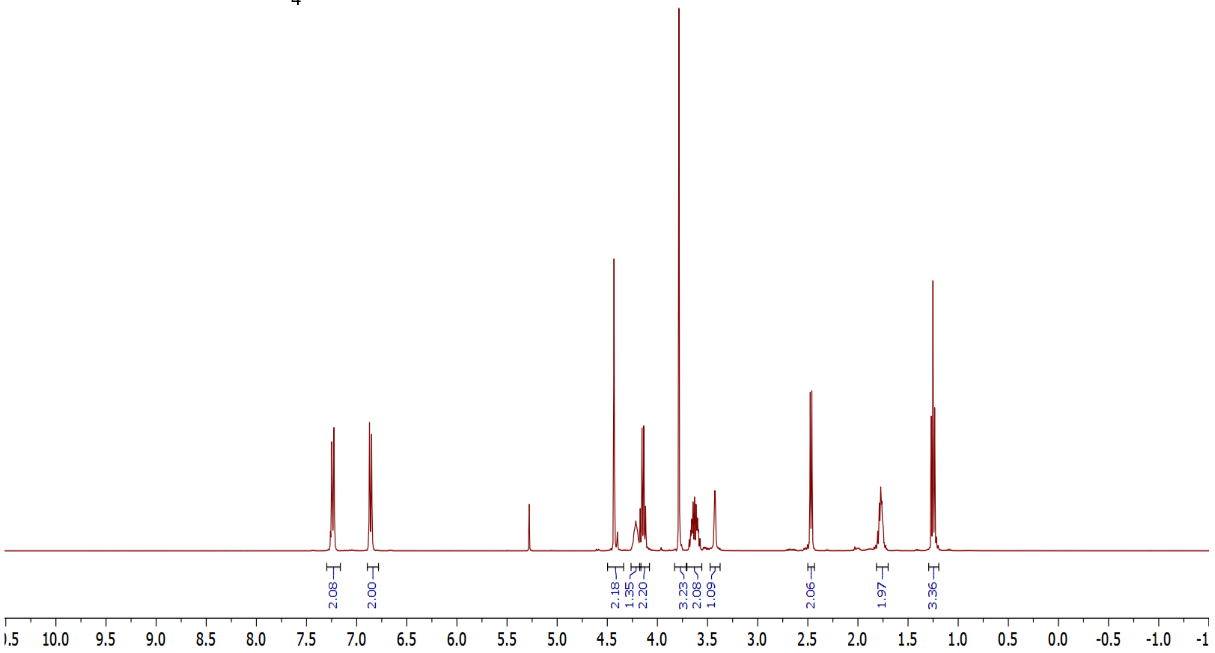


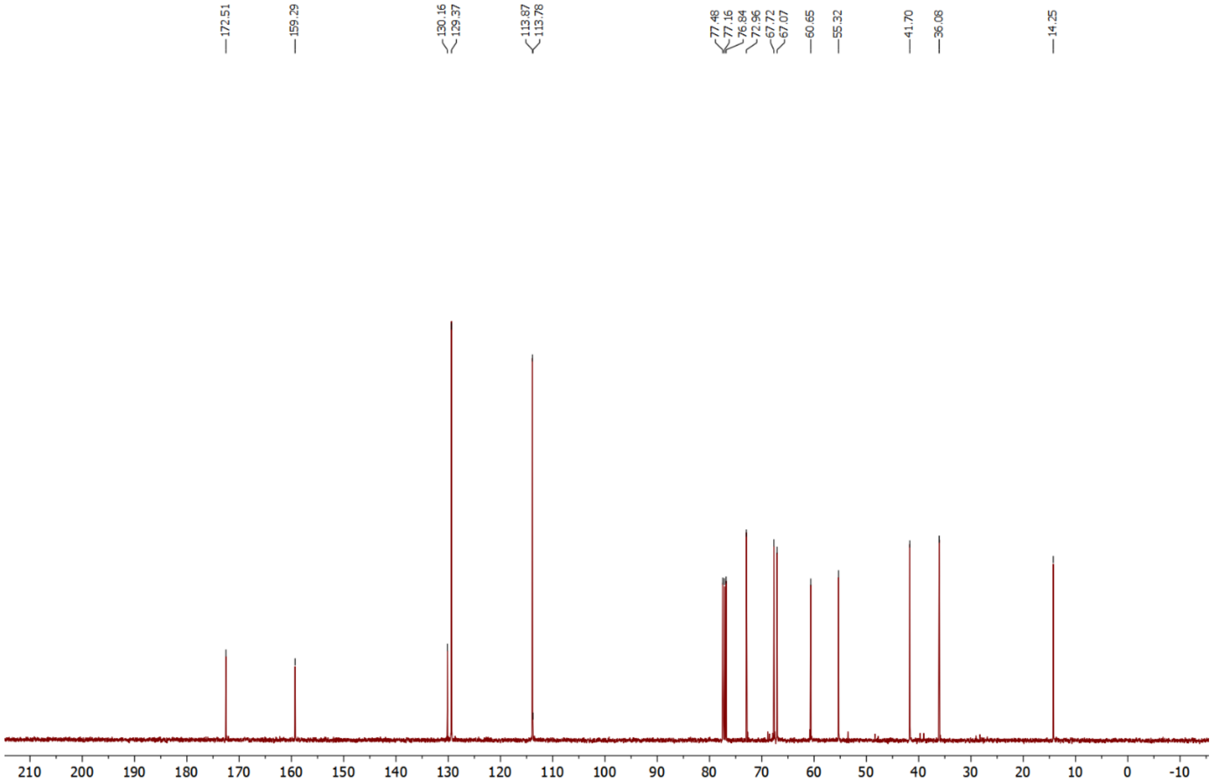

**Ethyl (*S*)-5-((4-methoxybenzyl)oxy)-3-(((*R*)-3,3,3-trifluoro-2-methoxy-2-phenylpropanoyl)oxy)-pentanoate, S8**

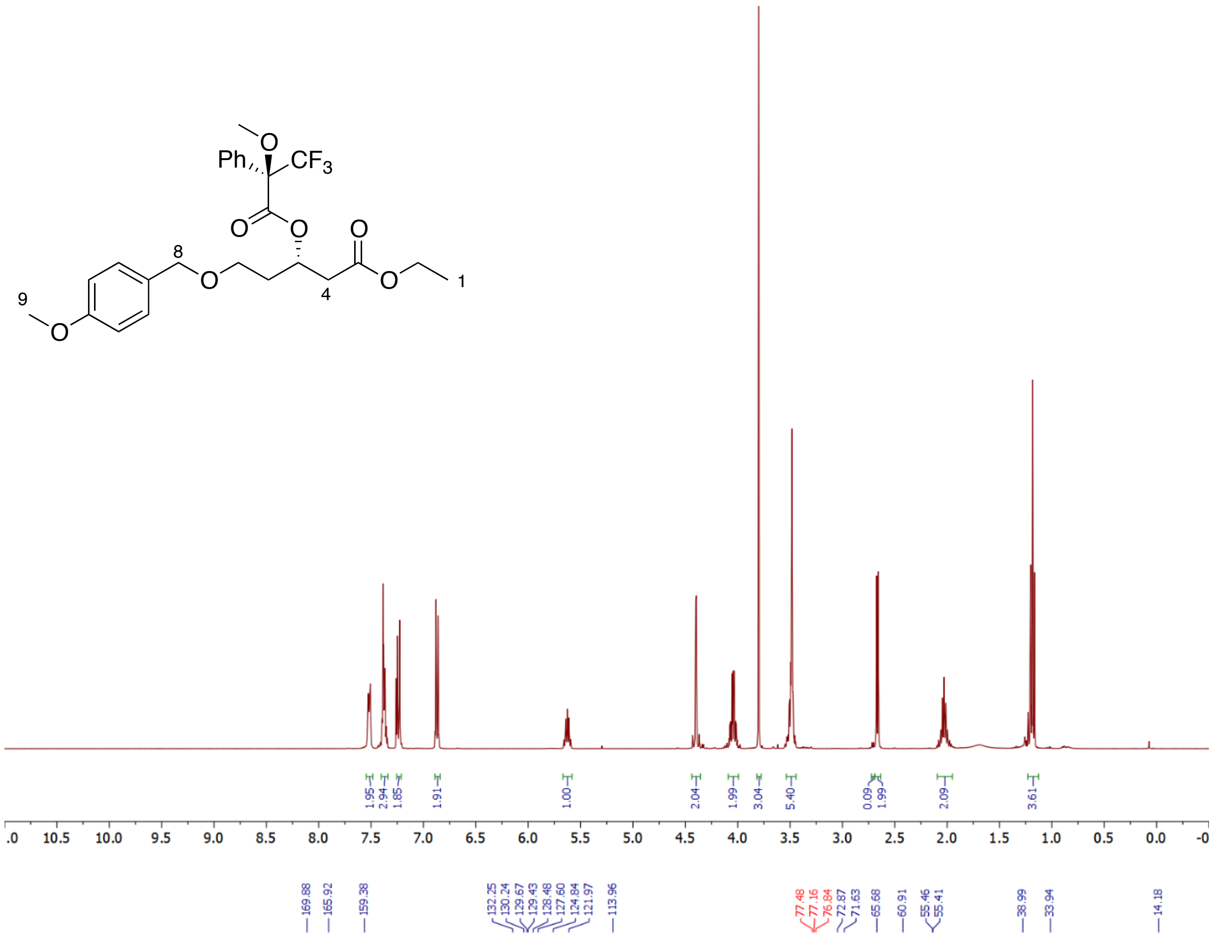

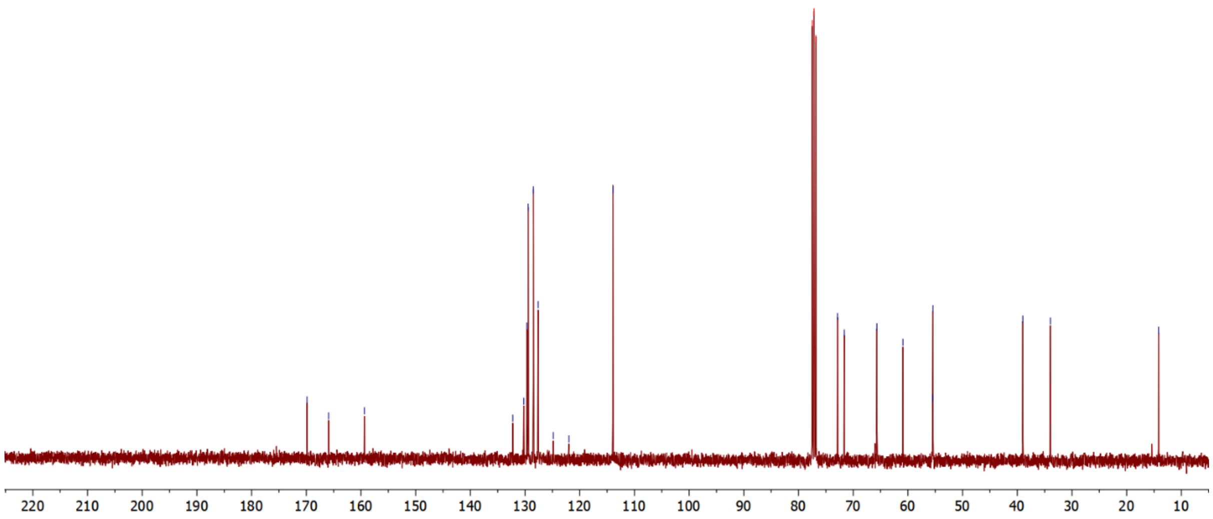

**Ethyl (*S*)-5-((4-methoxybenzyl)oxy)-3-(((*S*)-3,3,3-trifluoro-2-methoxy-2-phenylpropanoyl)oxy)-pentanoate, S9**

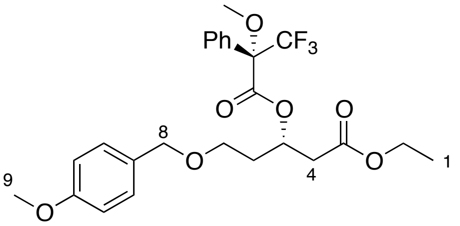


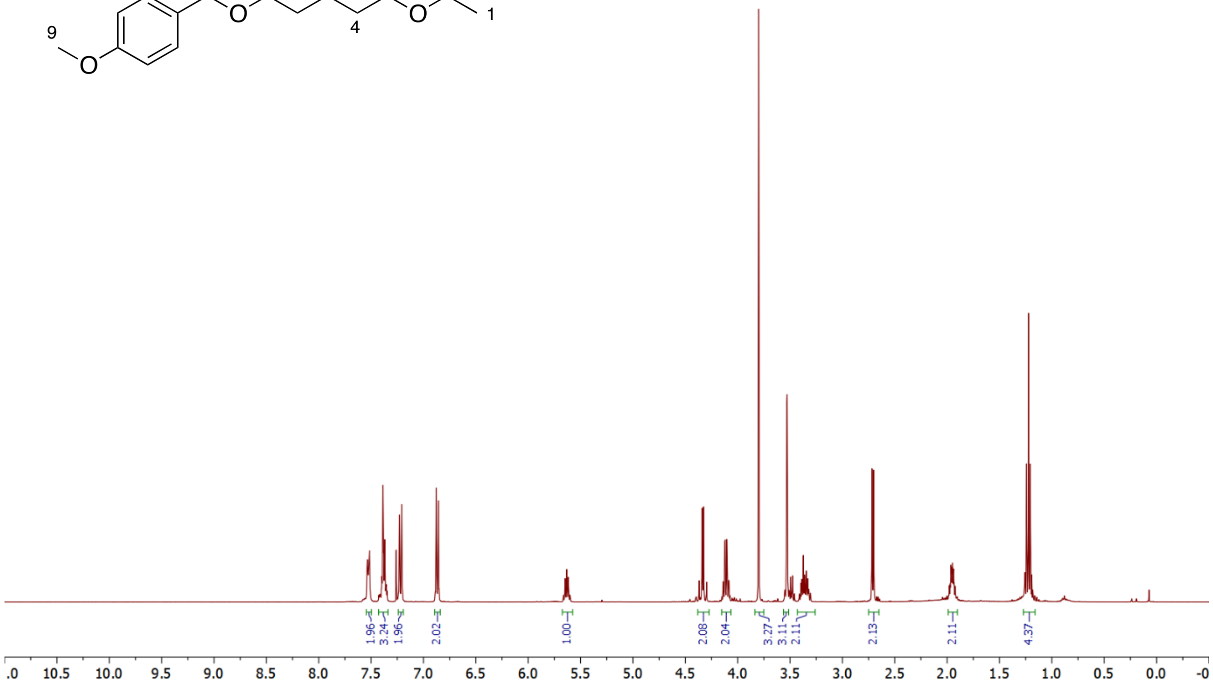


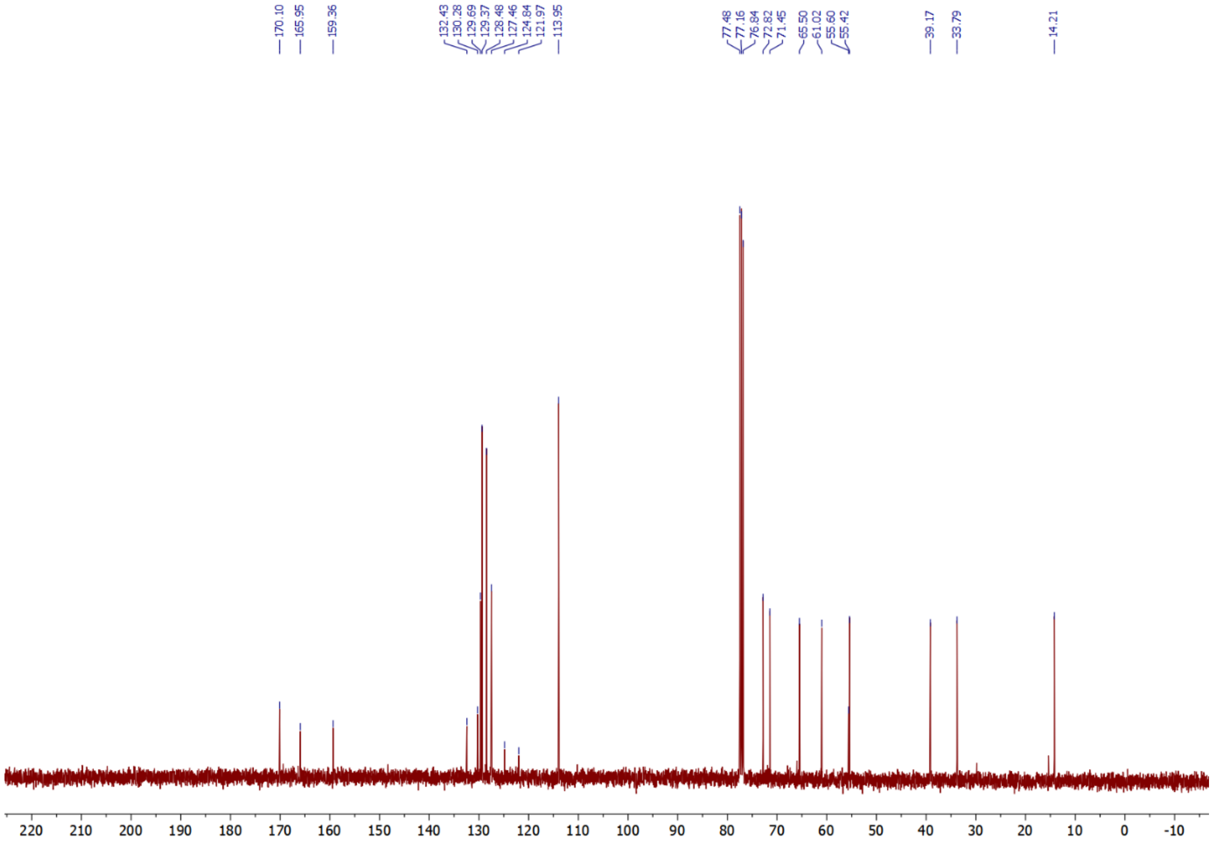

**Ethyl (*S*)-2-((*S*)-1-hydroxy-3-((4-methoxybenzyl)oxy)propyl)pent-4-enoate, 20**

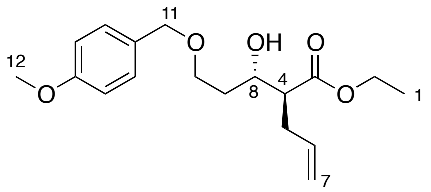


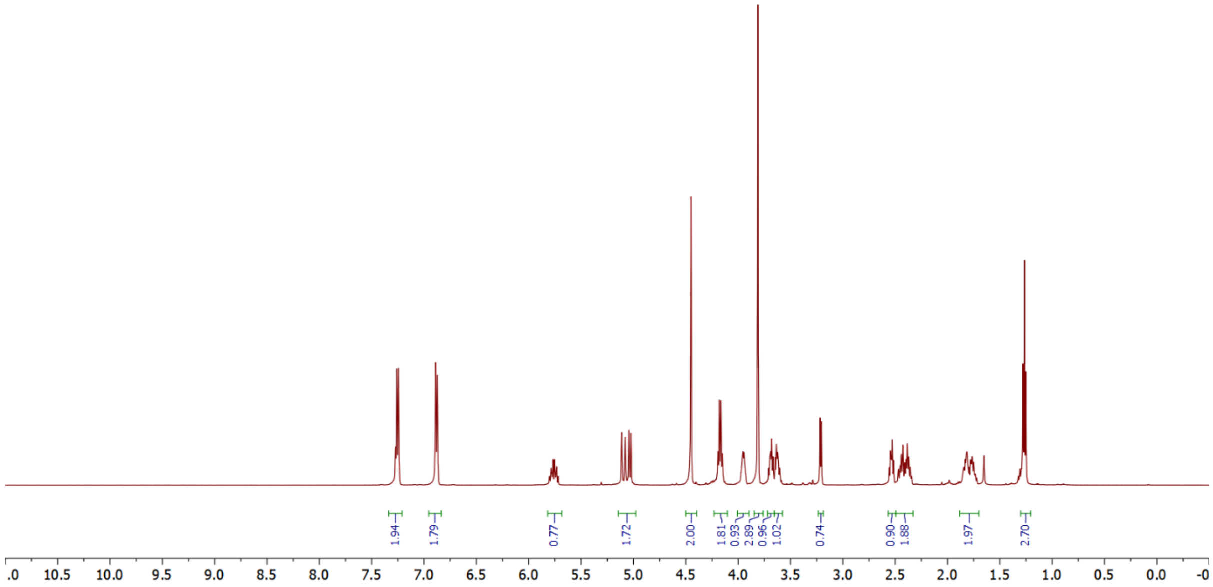

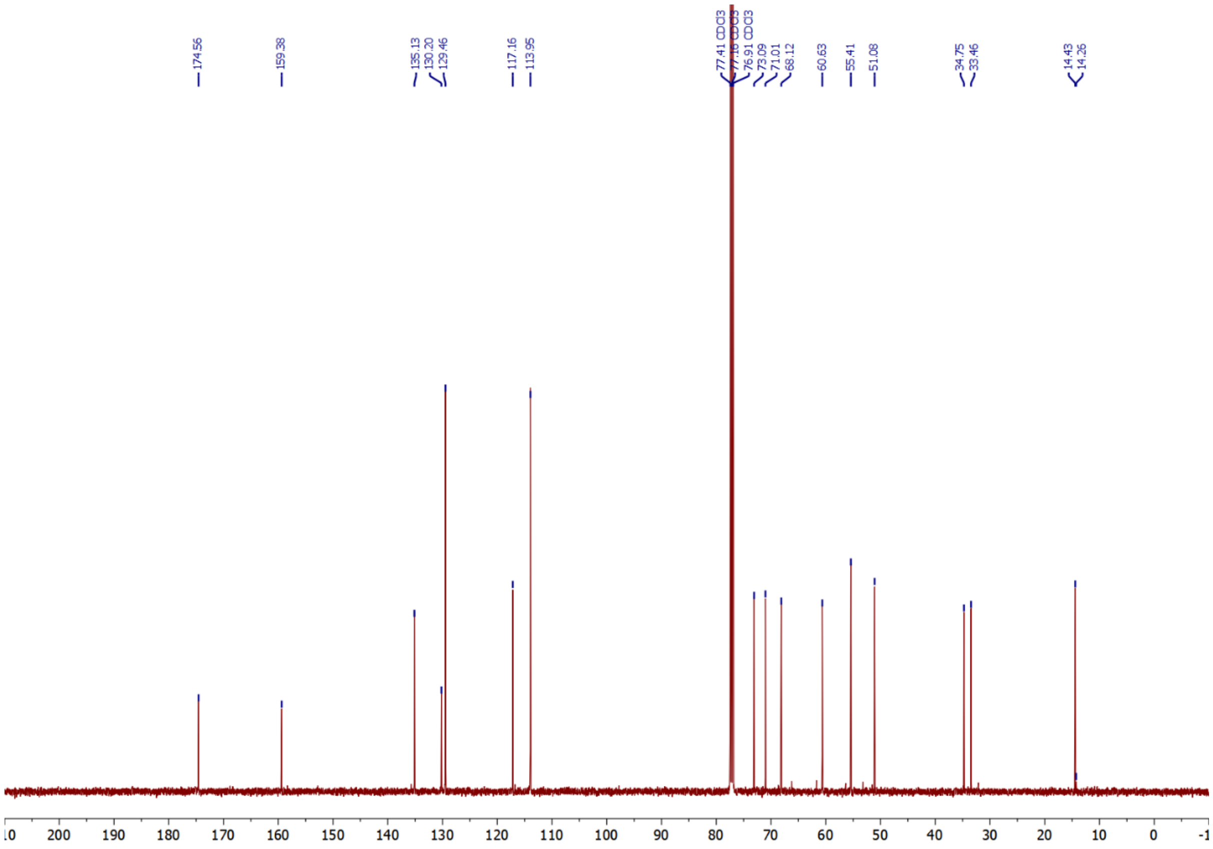

**(2*R*,3*S*)-2-Allyl-5-((4-methoxybenzyl)oxy)pentane-1,3-diol, S10**

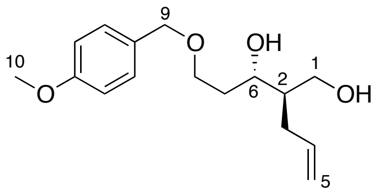


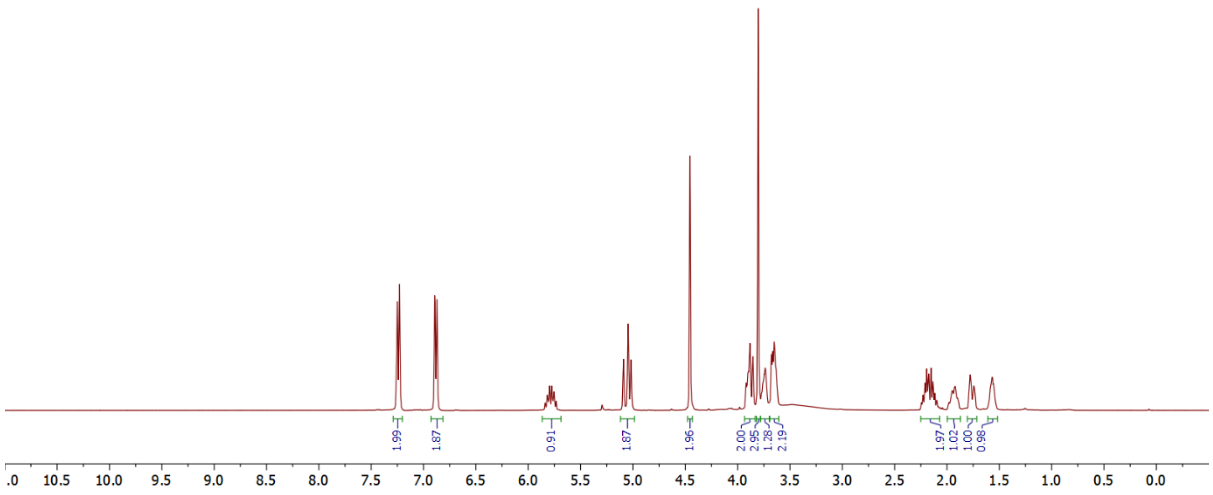

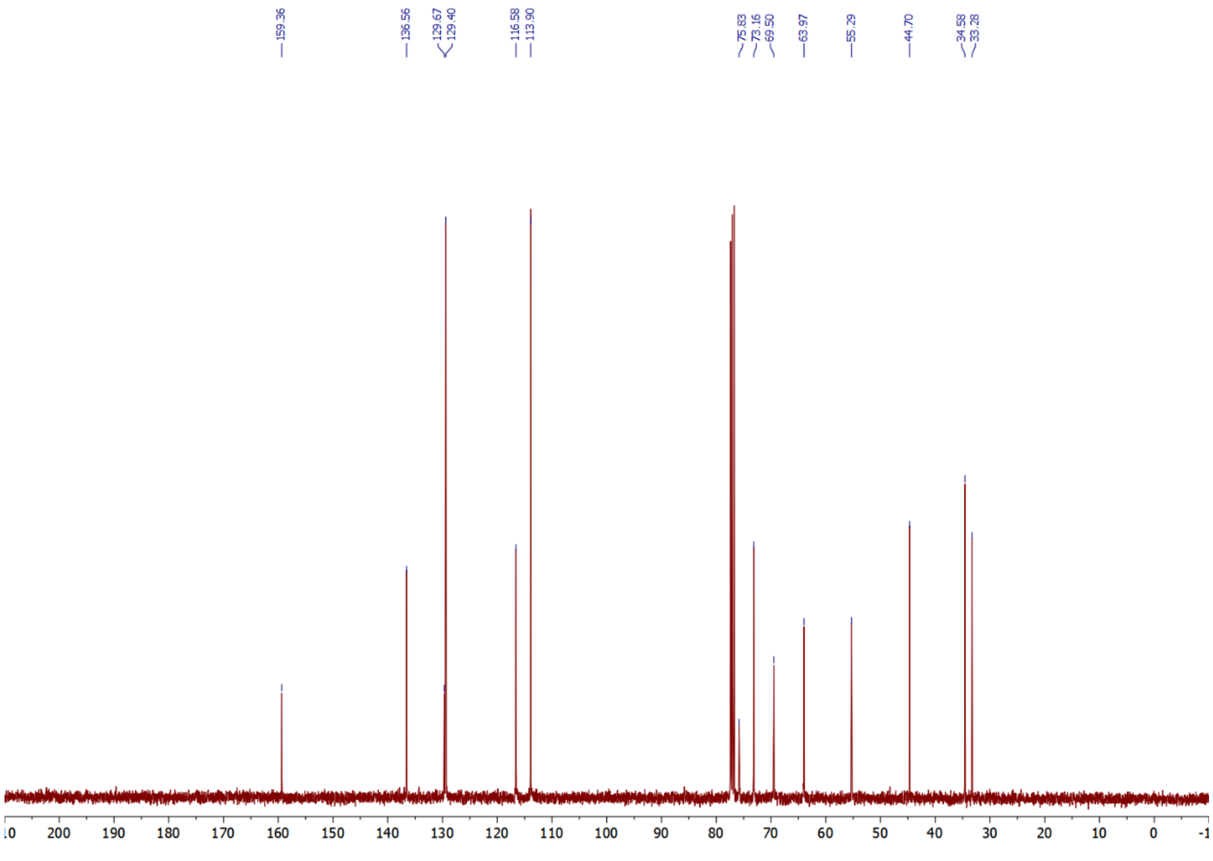

**(3*S*,4*R*)-4-(((*Tert*-butyldimethylsilyl)oxy)methyl)-1-((4-methoxybenzyl)oxy)hept-6-en-3-ol, S11**

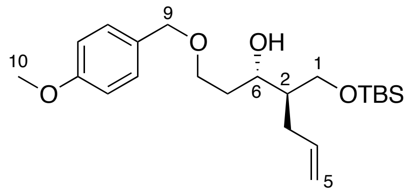


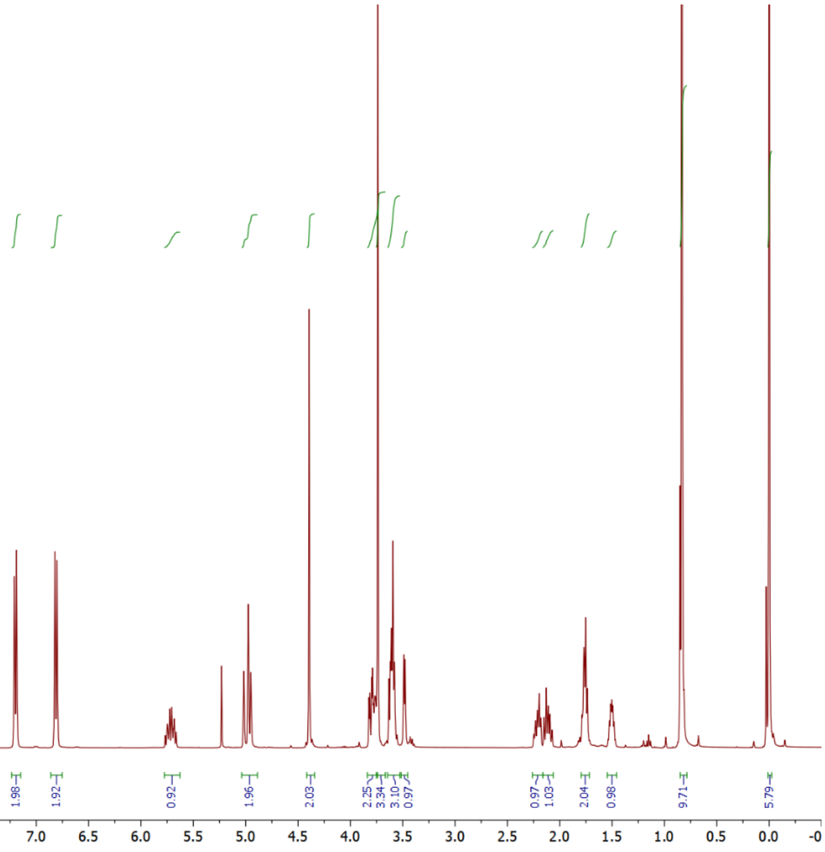

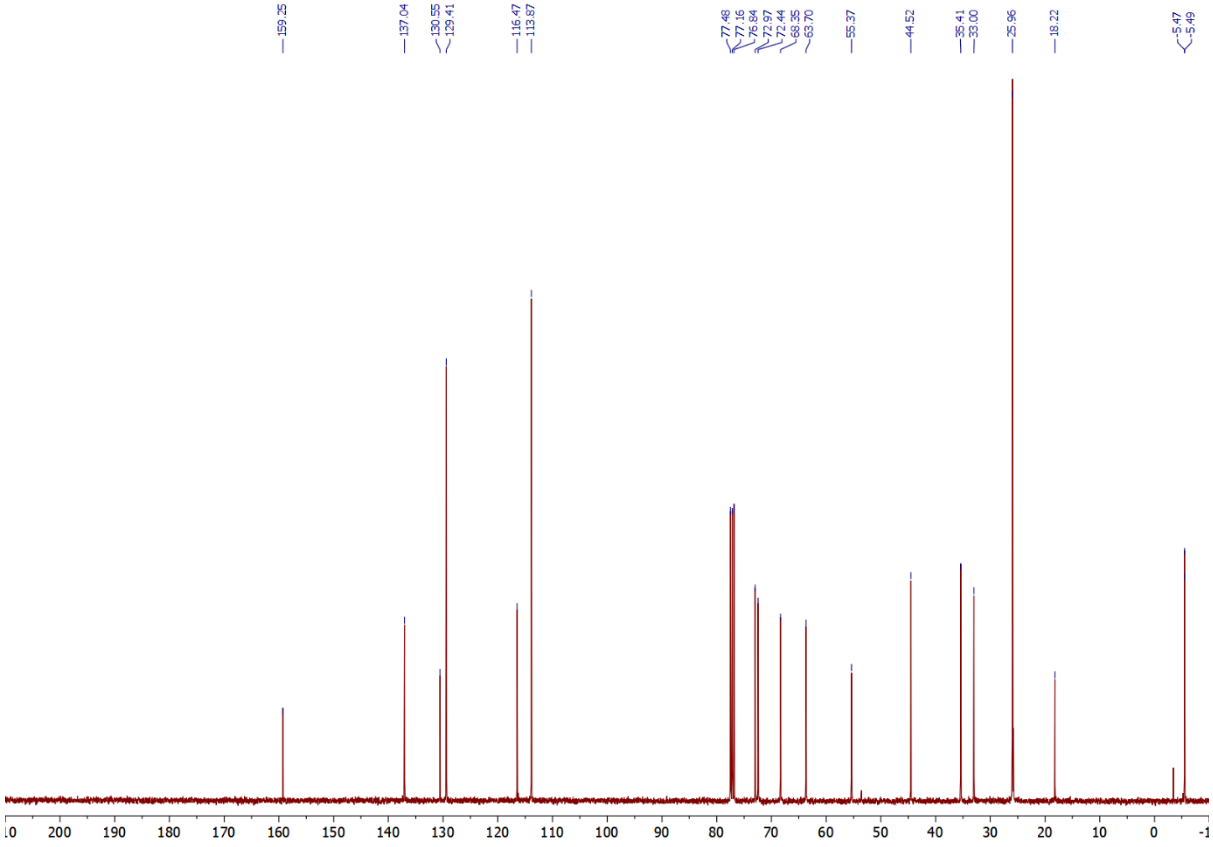

**(4*R*,5*S*)-4-(((*Tert*-butyldimethylsilyl)oxy)methyl)-5-(2-((4-methoxybenzyl)oxy)ethyl) dihydro furan-2(3*H*)-one, 21**

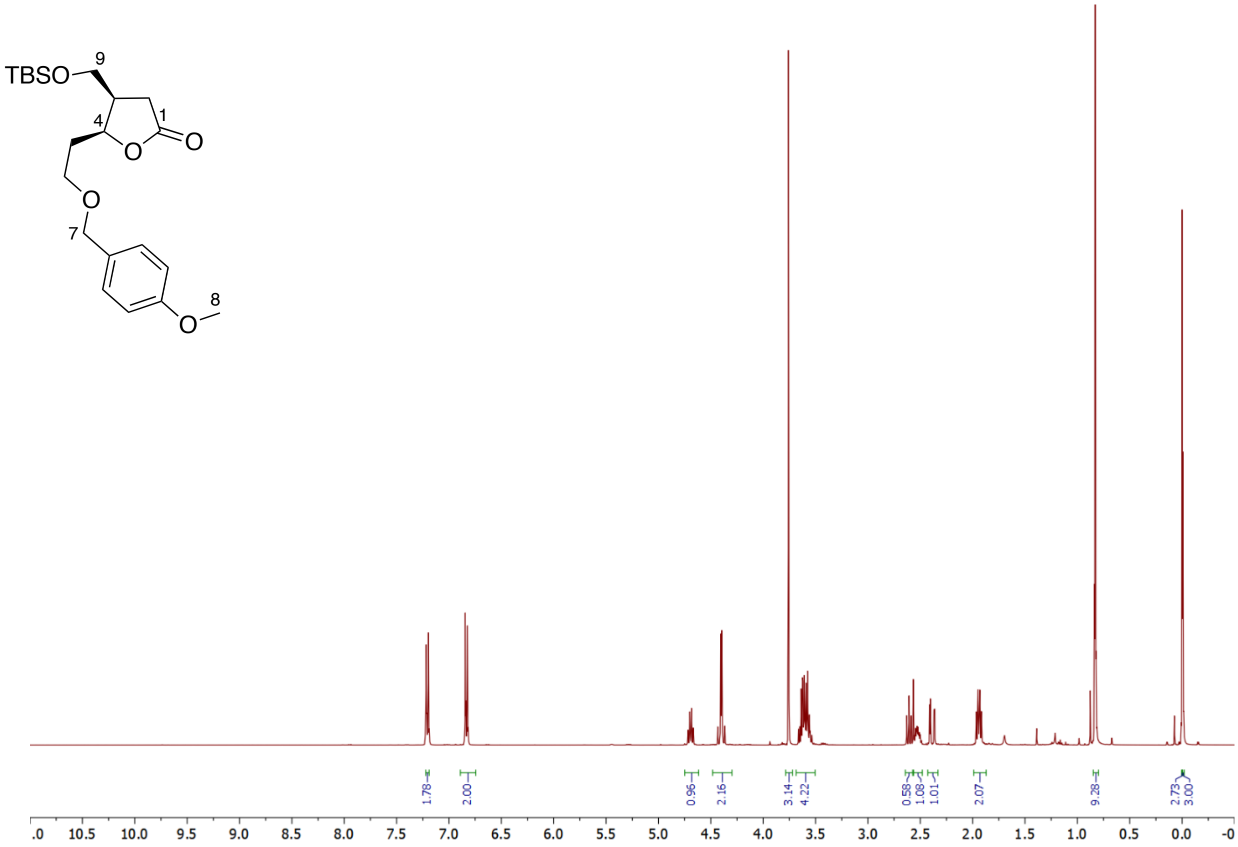


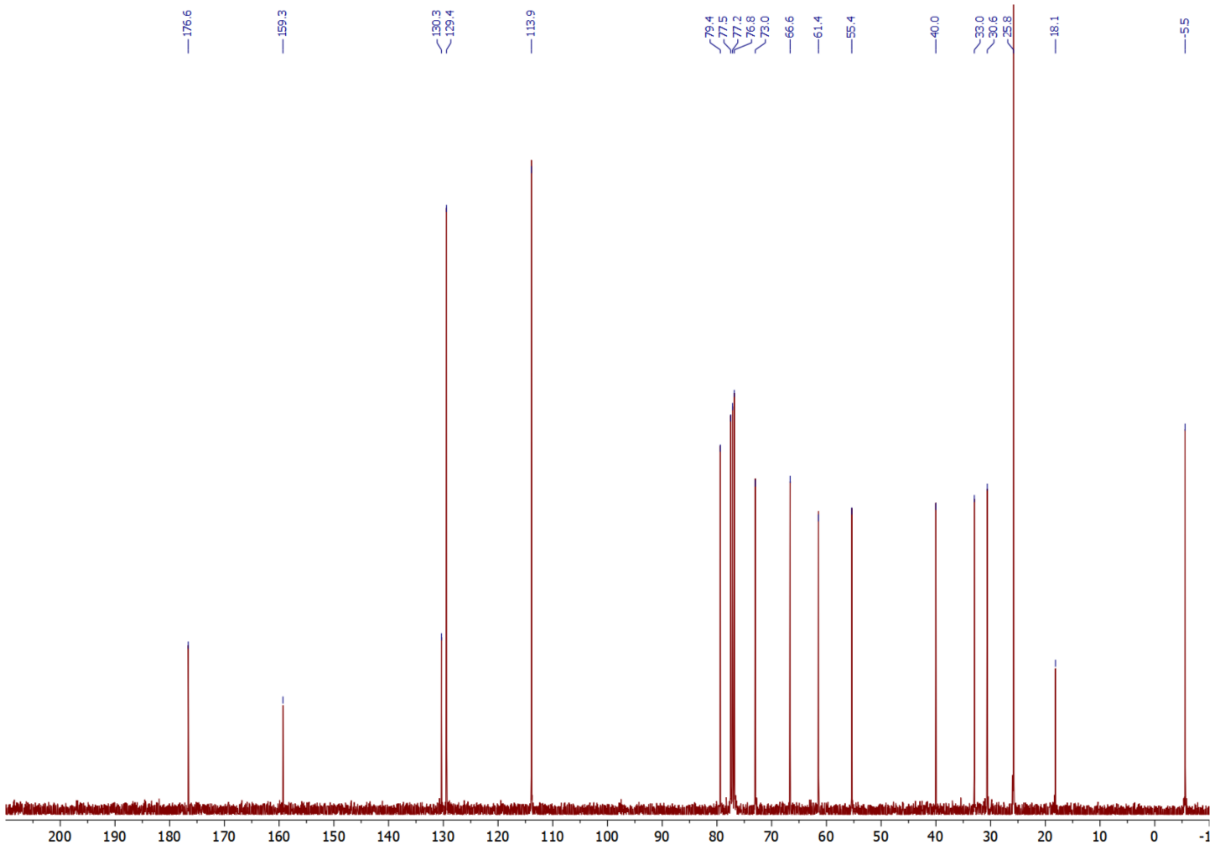

**(3*S*,4*R*,5*S*)-4-(((*Tert*-butyldimethylsilyl)oxy)methyl)-5-(2-((4-methoxybenzyl)oxy)ethyl)-3-methyldihydrofuran-2(3*H*)-one, 22**

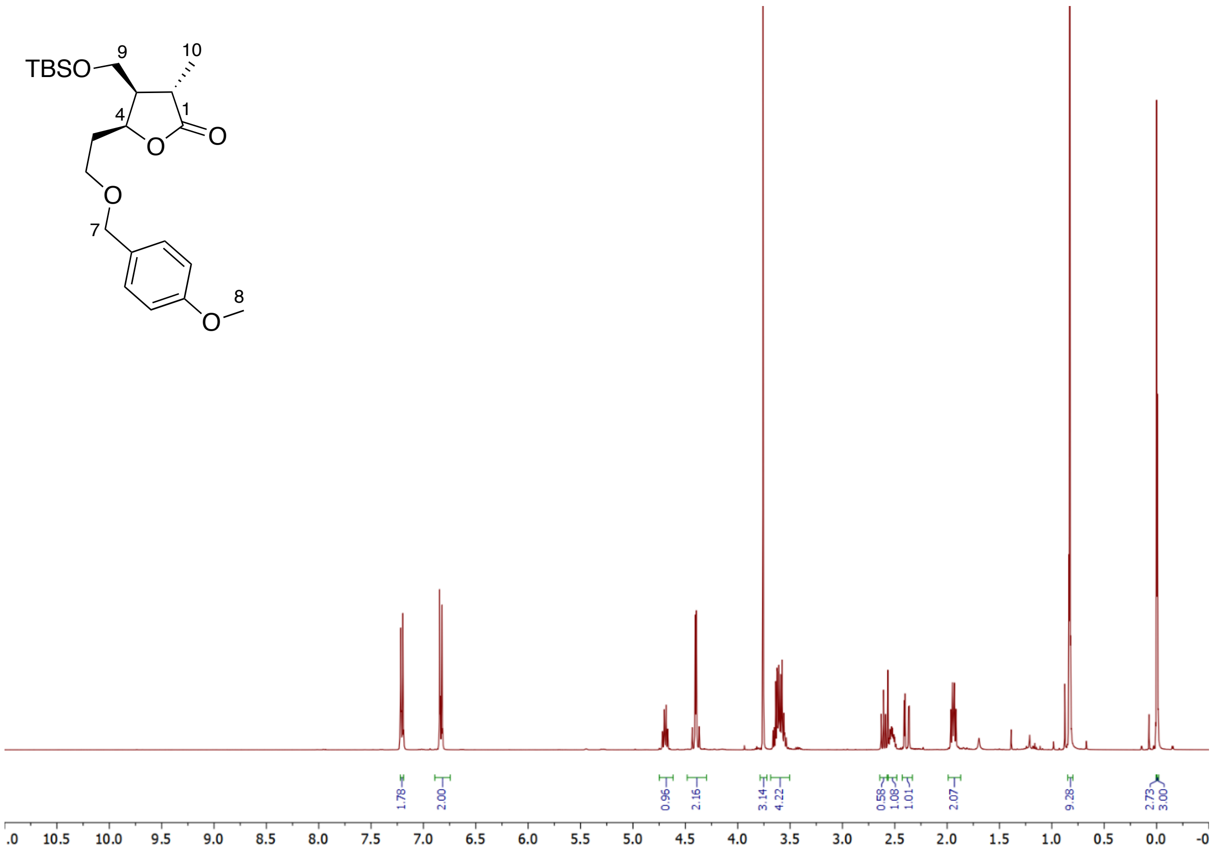


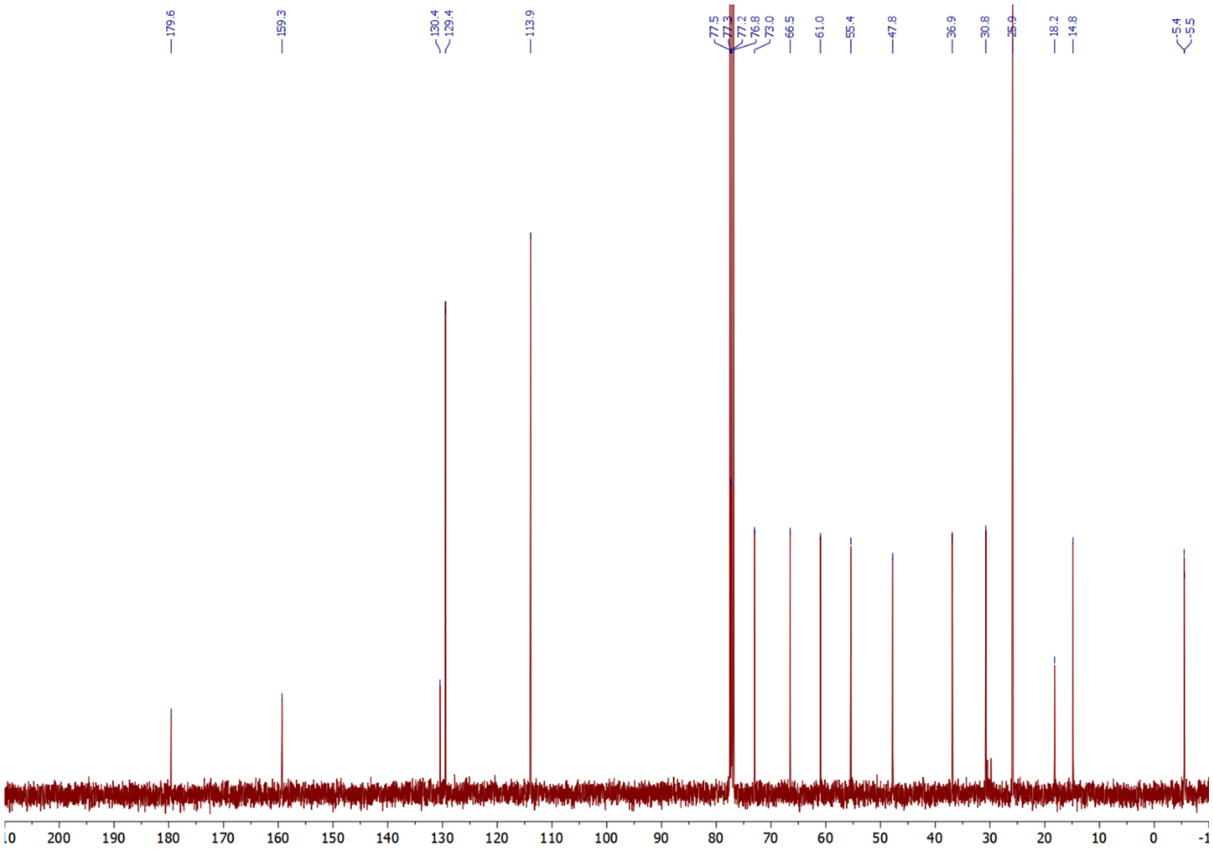

**((2*S*,3*R*,4*S*)-5-Methoxy-2-(2-((4-methoxybenzyl)oxy)ethyl)-4-methyltetrahydrofuran-3-yl)methanol, 23**

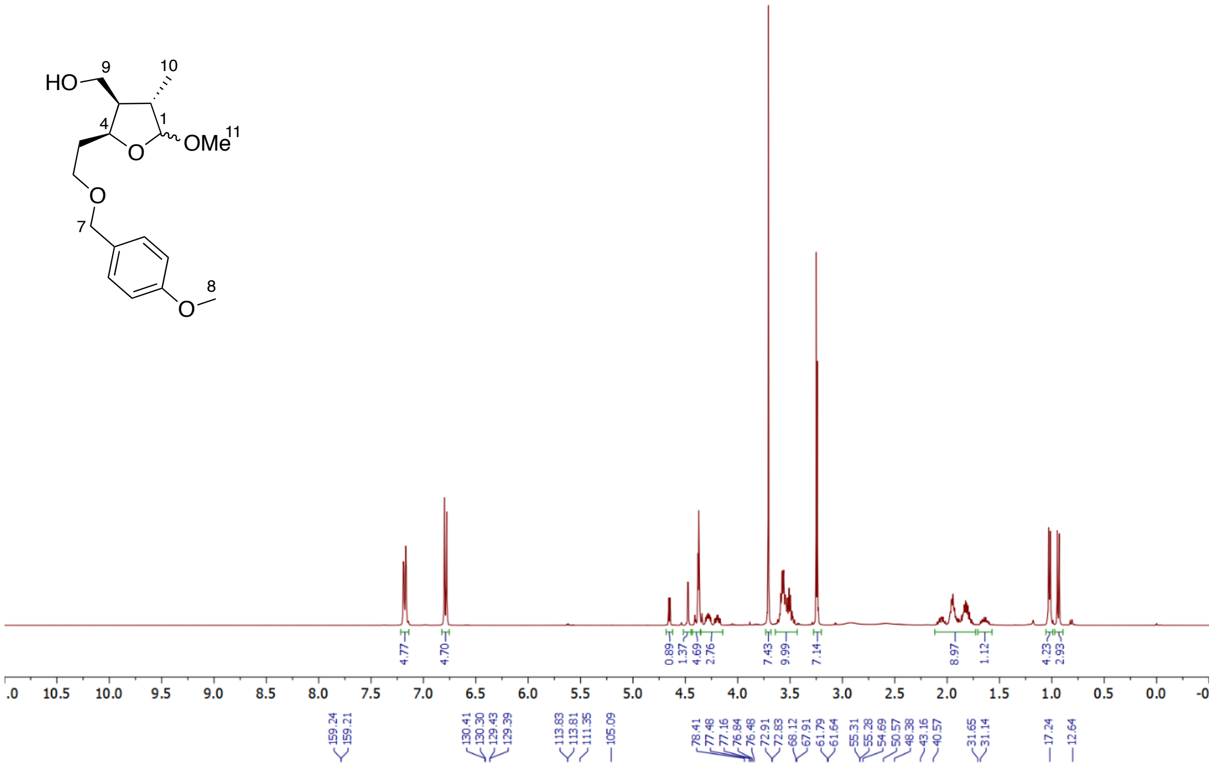


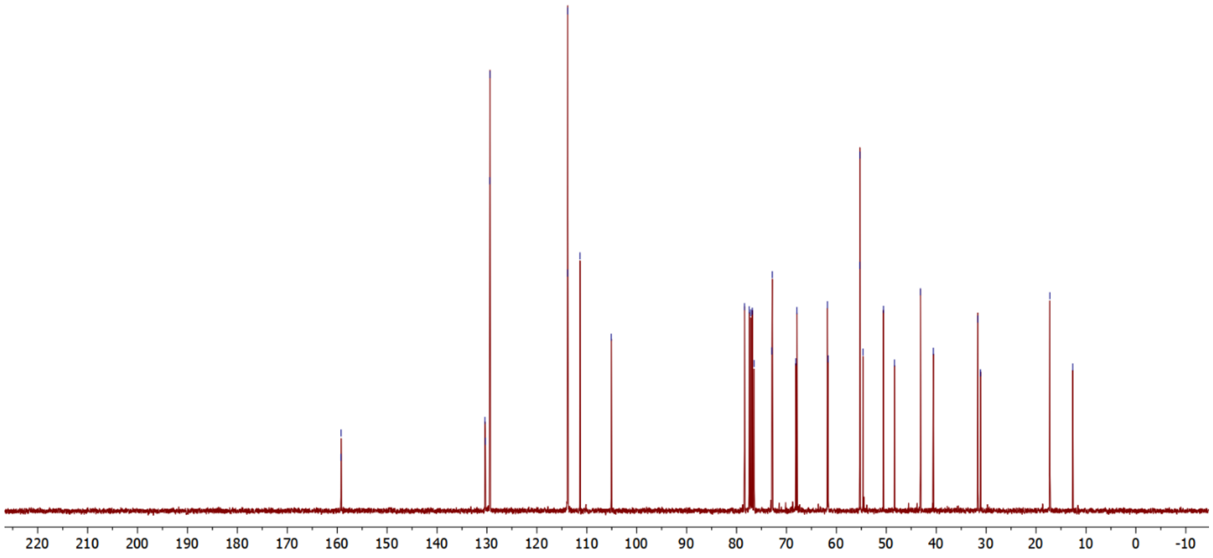

**(2S,3R,4S)-3-Ethynyl-5-methoxy-2-(2-((4-methoxybenzyl)oxy)ethyl)-4-methyltetrahydrofuran: S12**

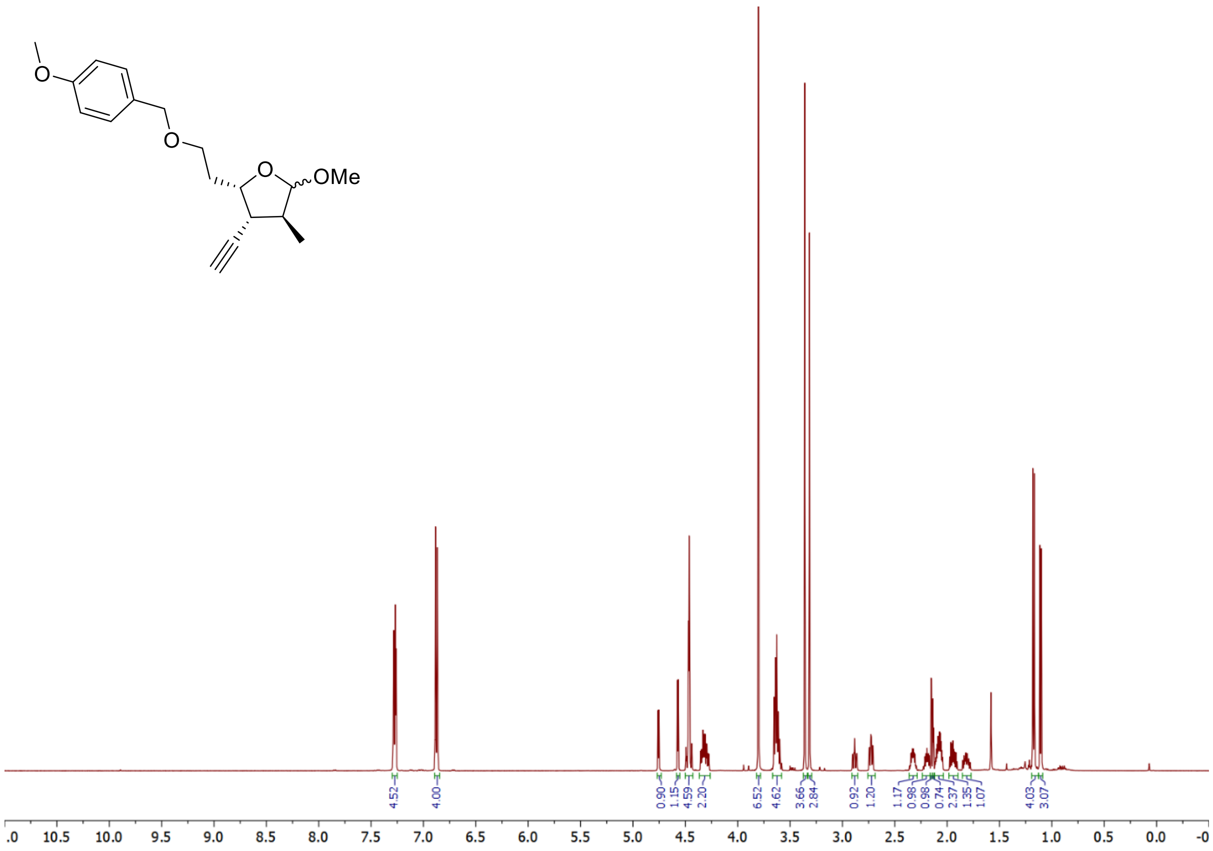


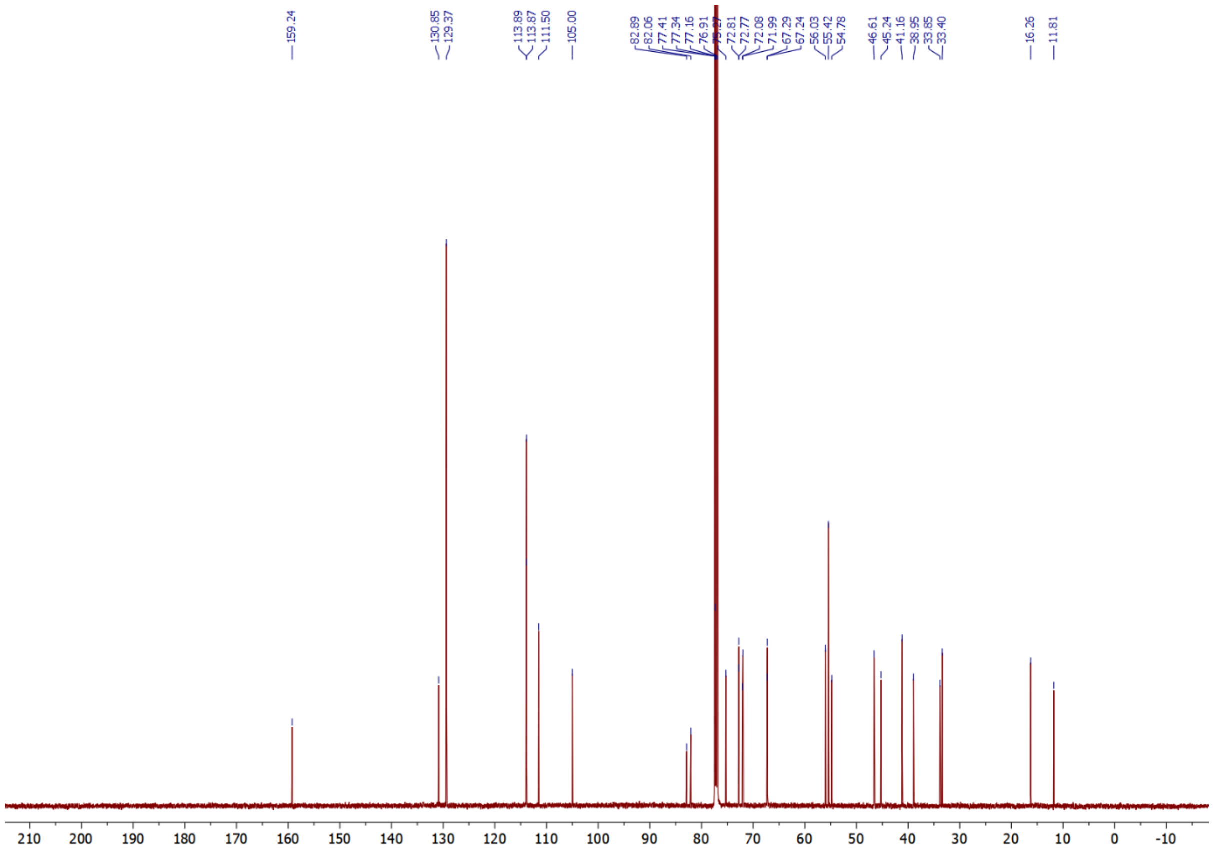

**(((2*S*,3*R*,4*S*)-5-Methoxy-2-(2-((4-methoxybenzyl)oxy)ethyl)-4-methyltetrahydrofuran-3-yl)ethynyl)trimethylsilane, S13**

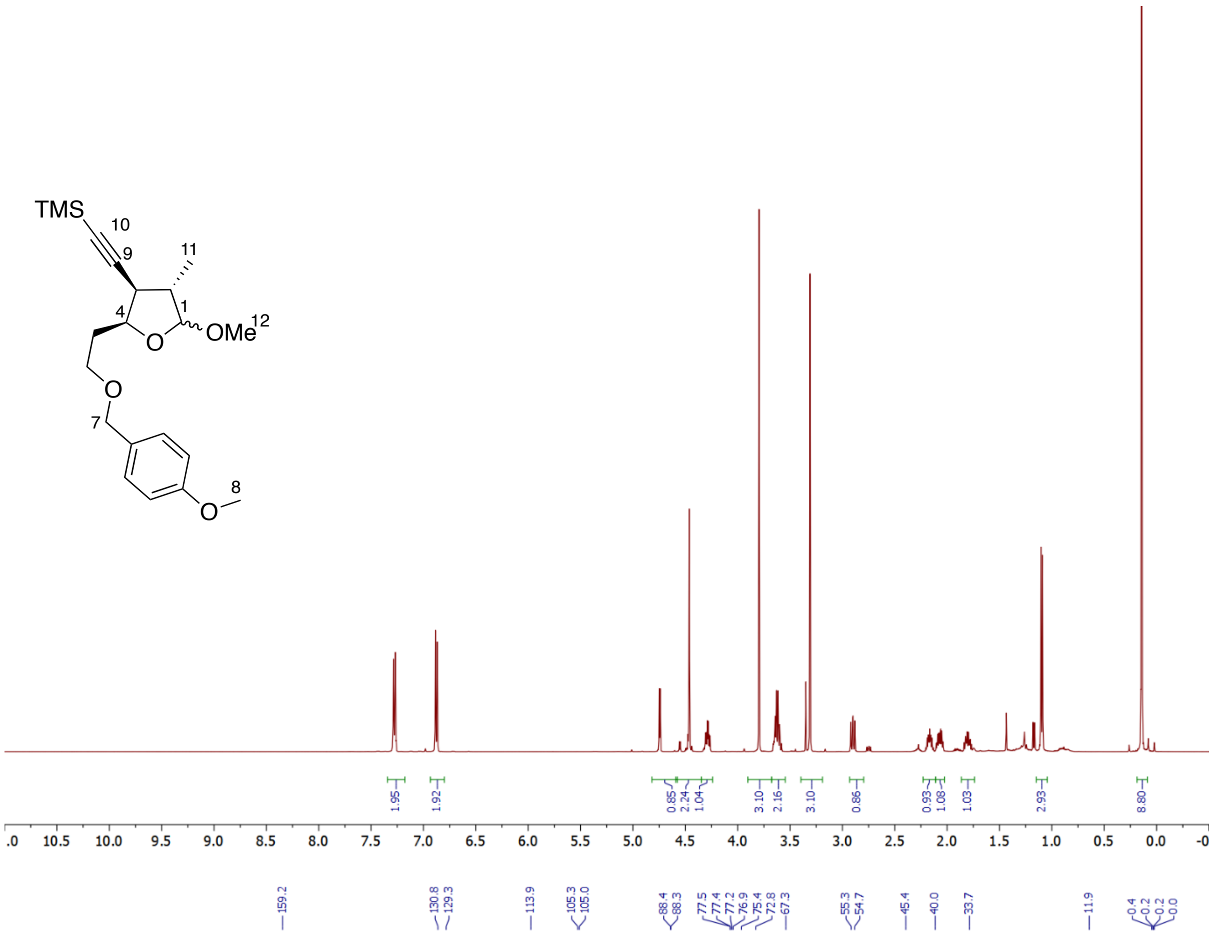


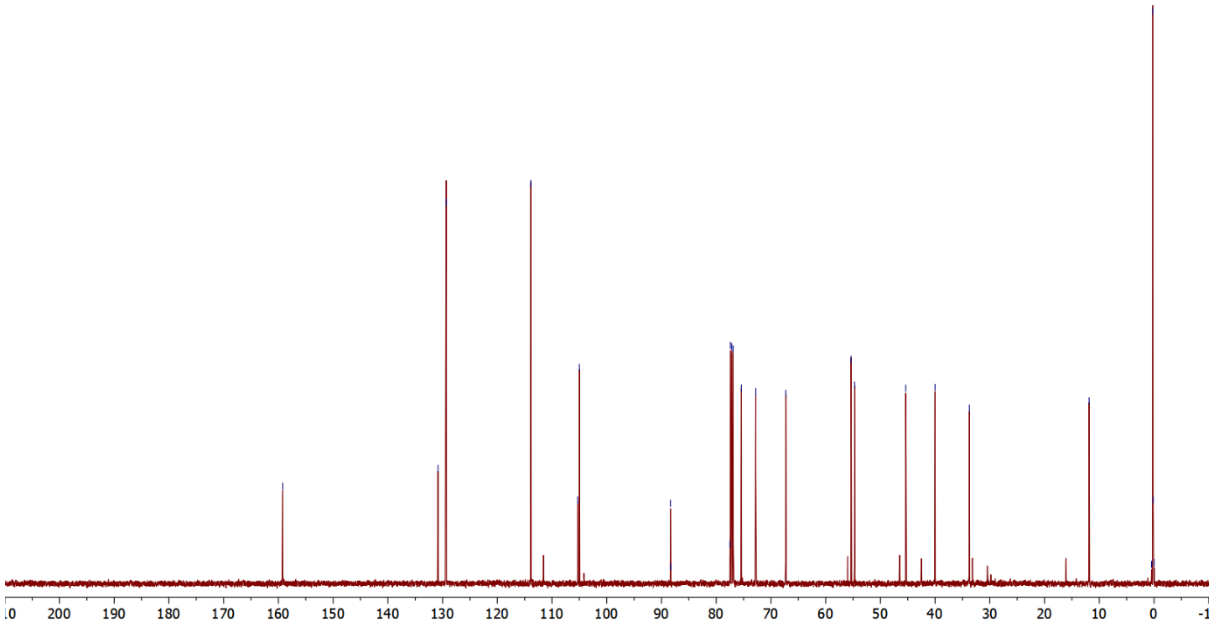

**(((2*S*,3*R*,4*S*)-5-methoxy-4-methyl-2-(prop-2-yn-1-yl)tetrahydrofuran-3-yl)ethynyl)trimethyl-silane, 25**

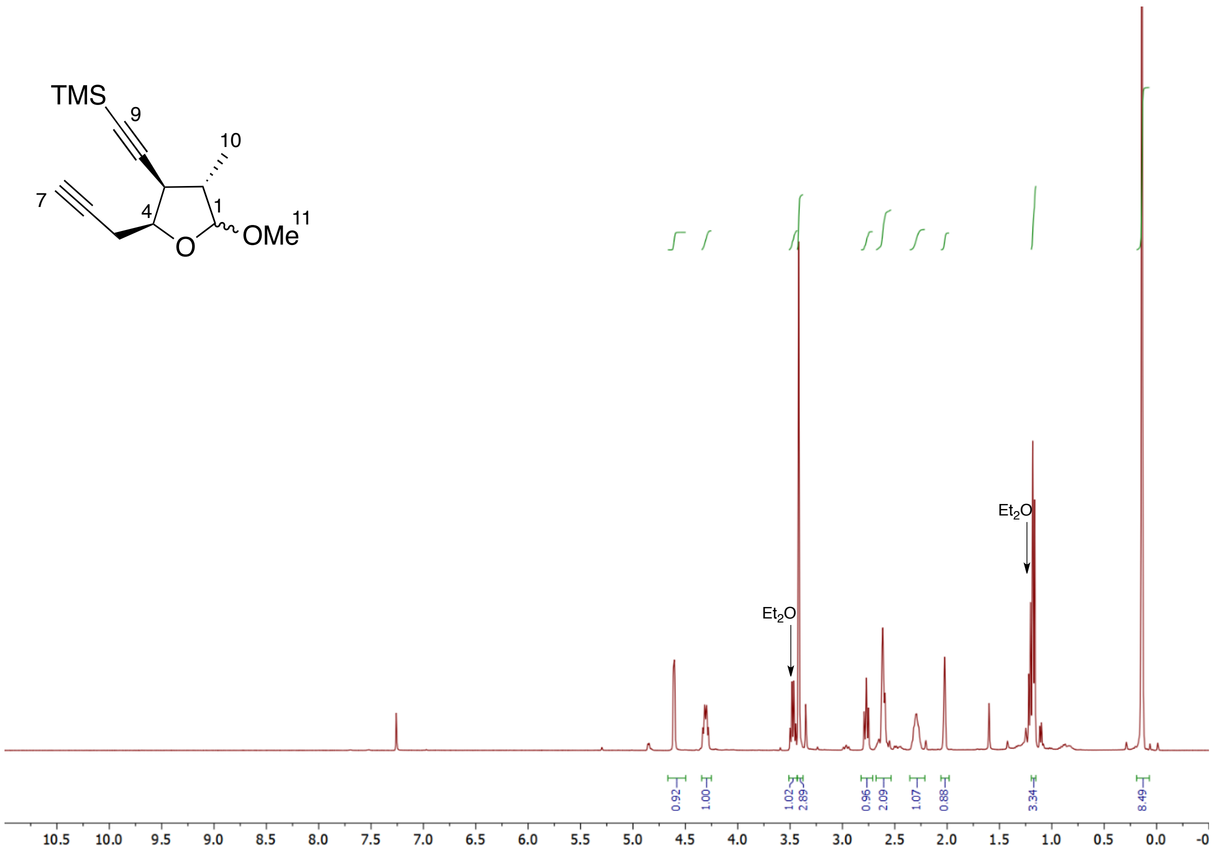


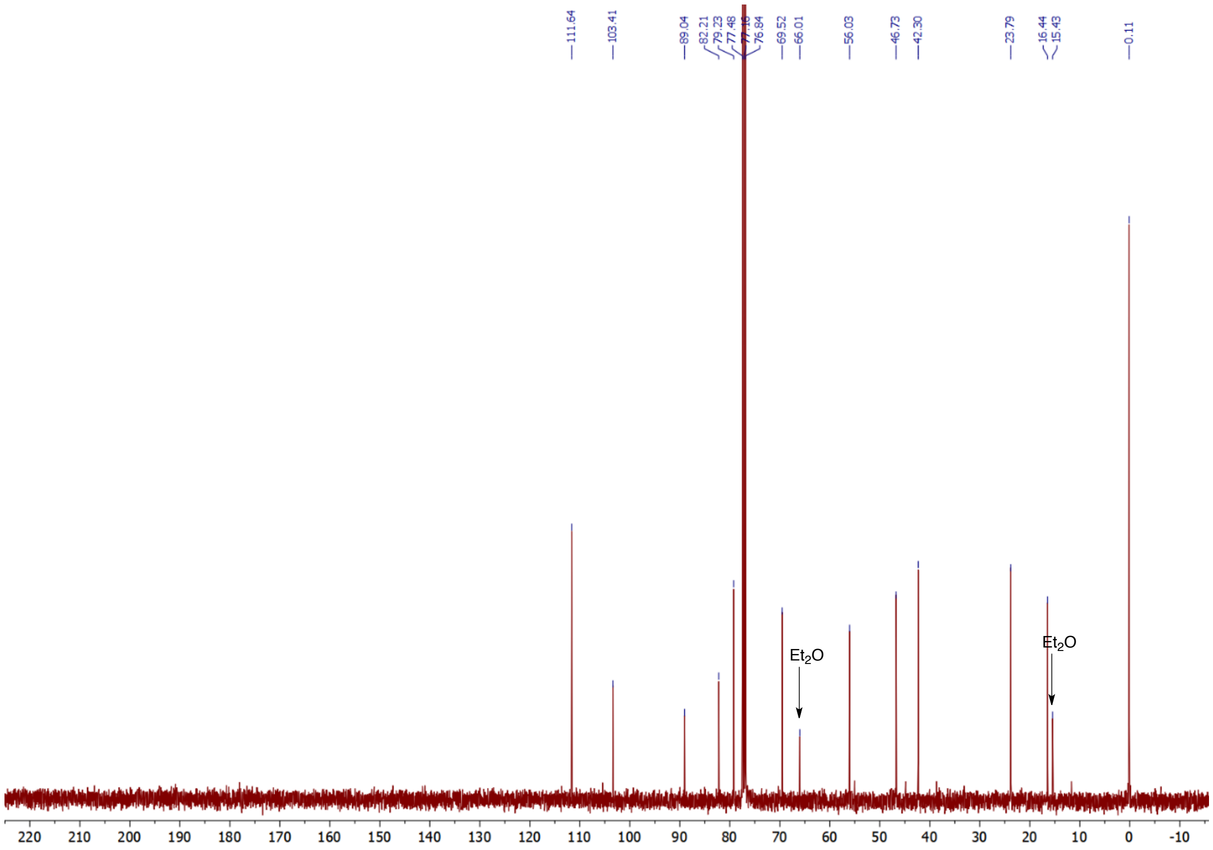

**(3*S*,4*R*,5*S*)-3-Methyl-5-(prop-2-yn-1-yl)-4-((trimethylsilyl)ethynyl)tetrahydrofuran-2-yl acetate, S14**

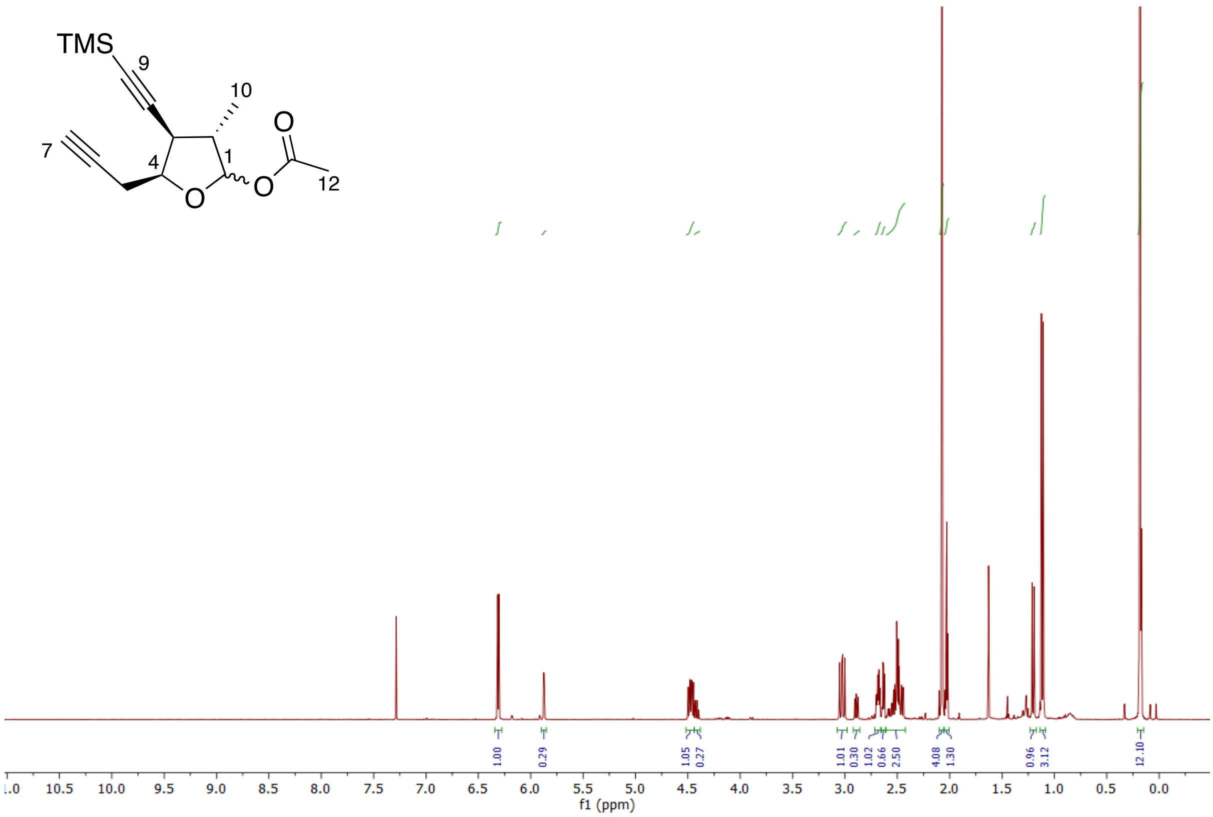


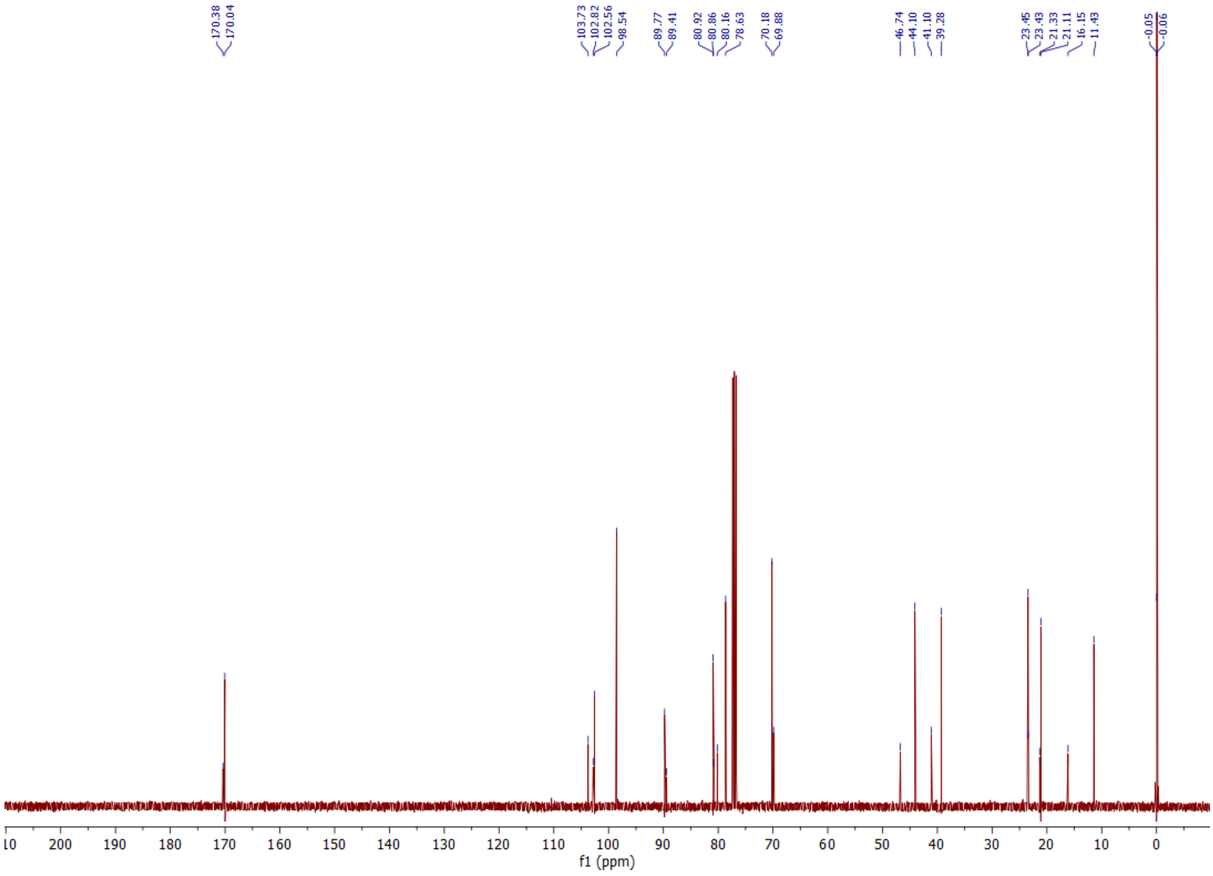

**(3*S*,4*R*,5*S*)-5-(3-Iodoprop-2-yn-1-yl)-3-methyl-4-((trimethylsilyl)ethynyl)tetrahydrofuran-2-yl acetate, 26**

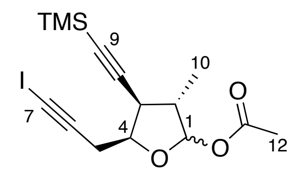


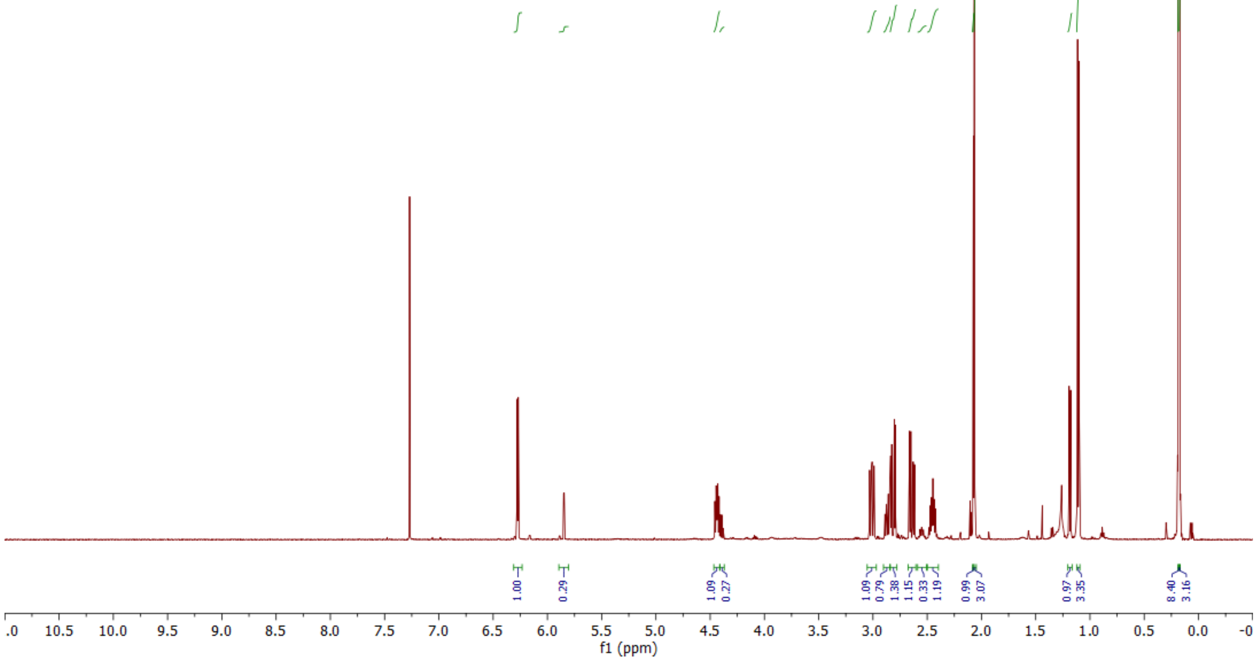


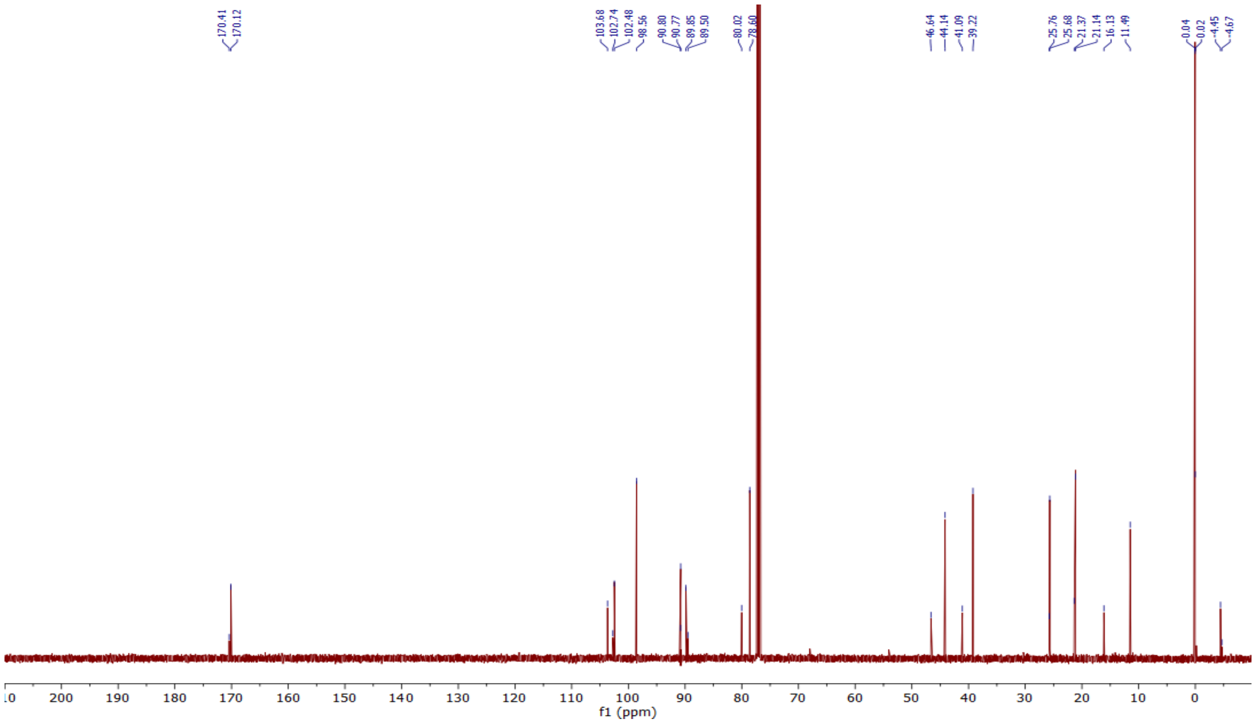

**5-((3*S*,4*R*,5*S*)-5-(3-Iodoprop-2-yn-1-yl)-3-methyl-4-((trimethylsilyl)ethynyl)-tetrahydrofuran-2-yl)-3-methylfuran-2(5*H*)-one, 8a/8d**

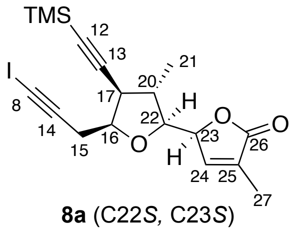


**8a** (C22*S*, C23*S*)

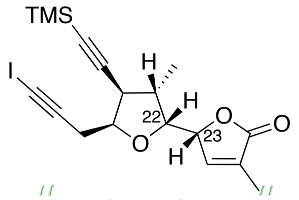


**8d** (C22*R*, C23*R*)

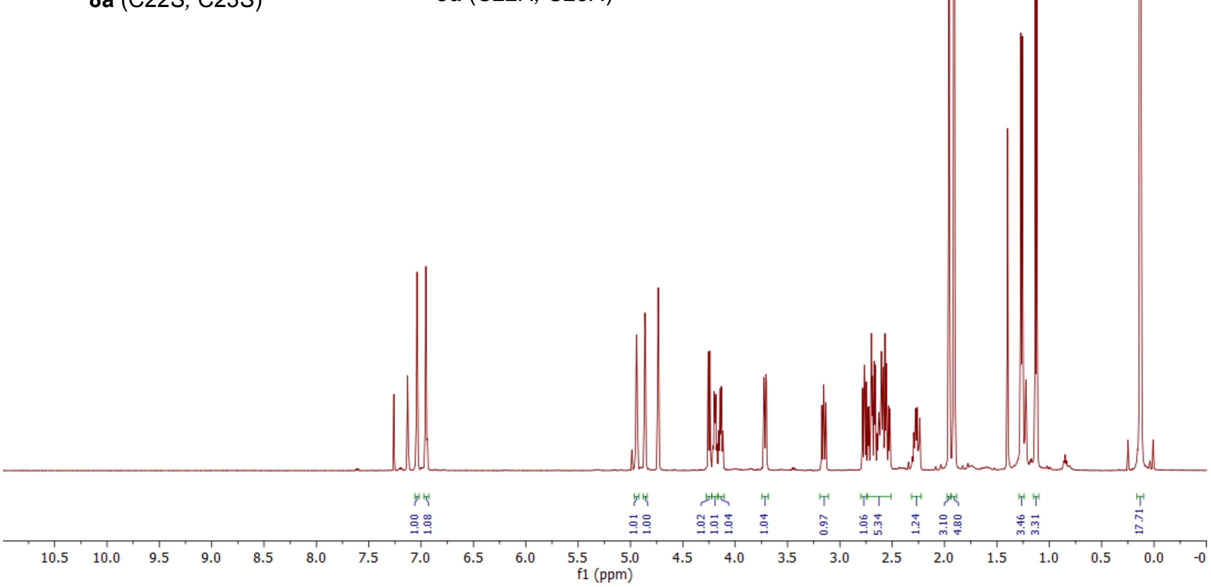


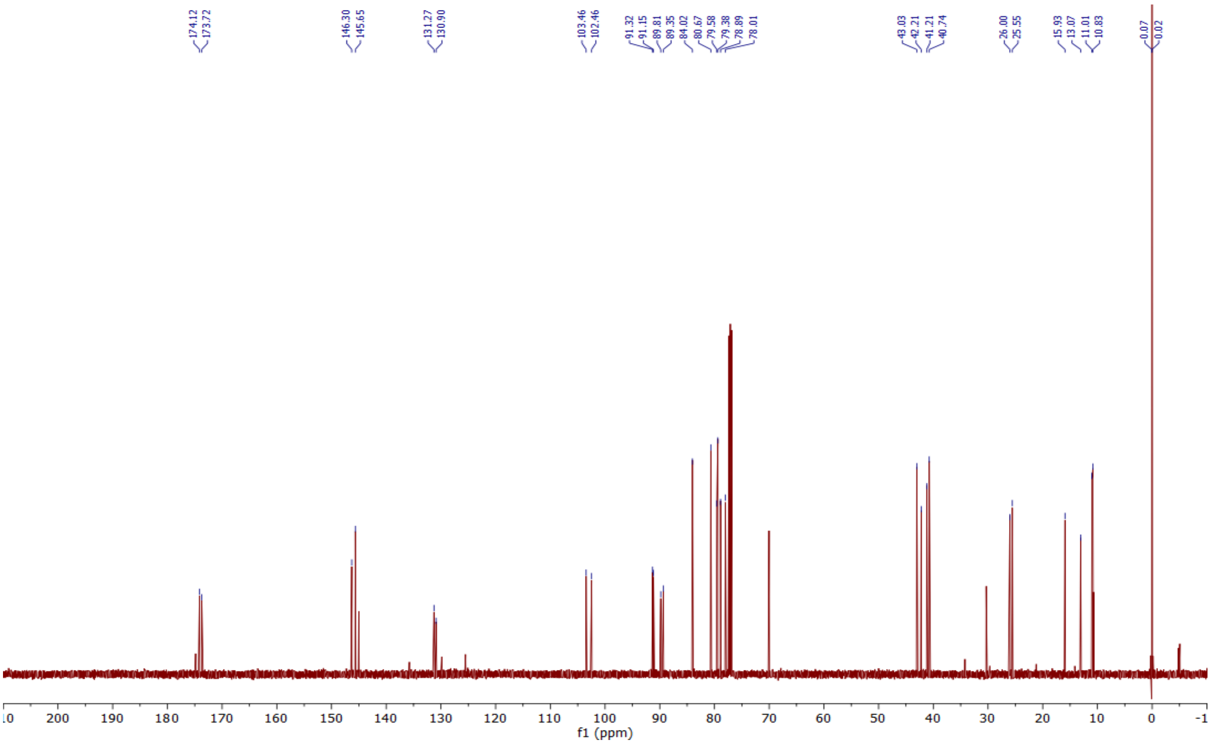

$^{1}H–^{1}H$ NOESY ($CDCl_3$)

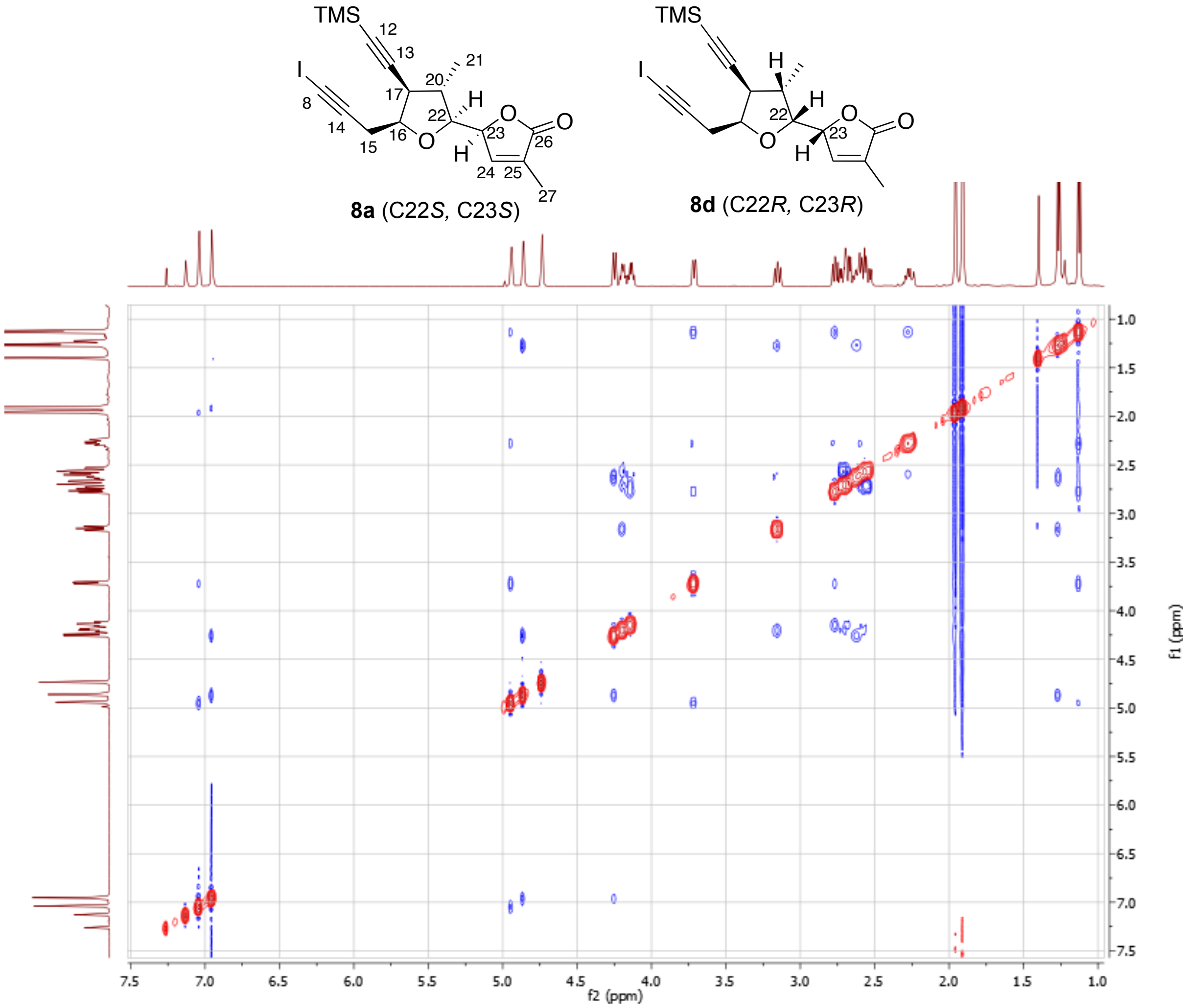

**(*S*)-5-((2*S*,3*S*,4*R*,5*S*)-5-(3-Iodoprop-2-yn-1-yl)-3-methyl-4-((trimethylsilyl)ethynyl)-tetrahydrofuran-2-yl)-3-methylfuran-2(5*H*)-one, 8b**

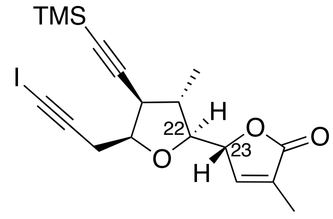


**8b** (C22*S*, C23*R*)

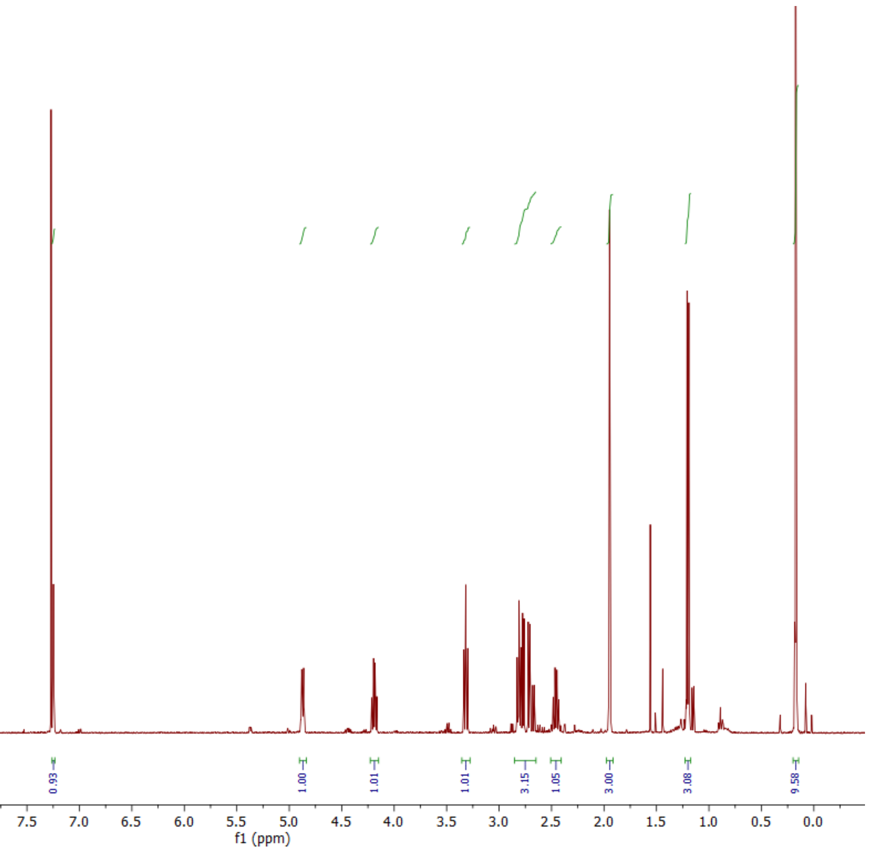


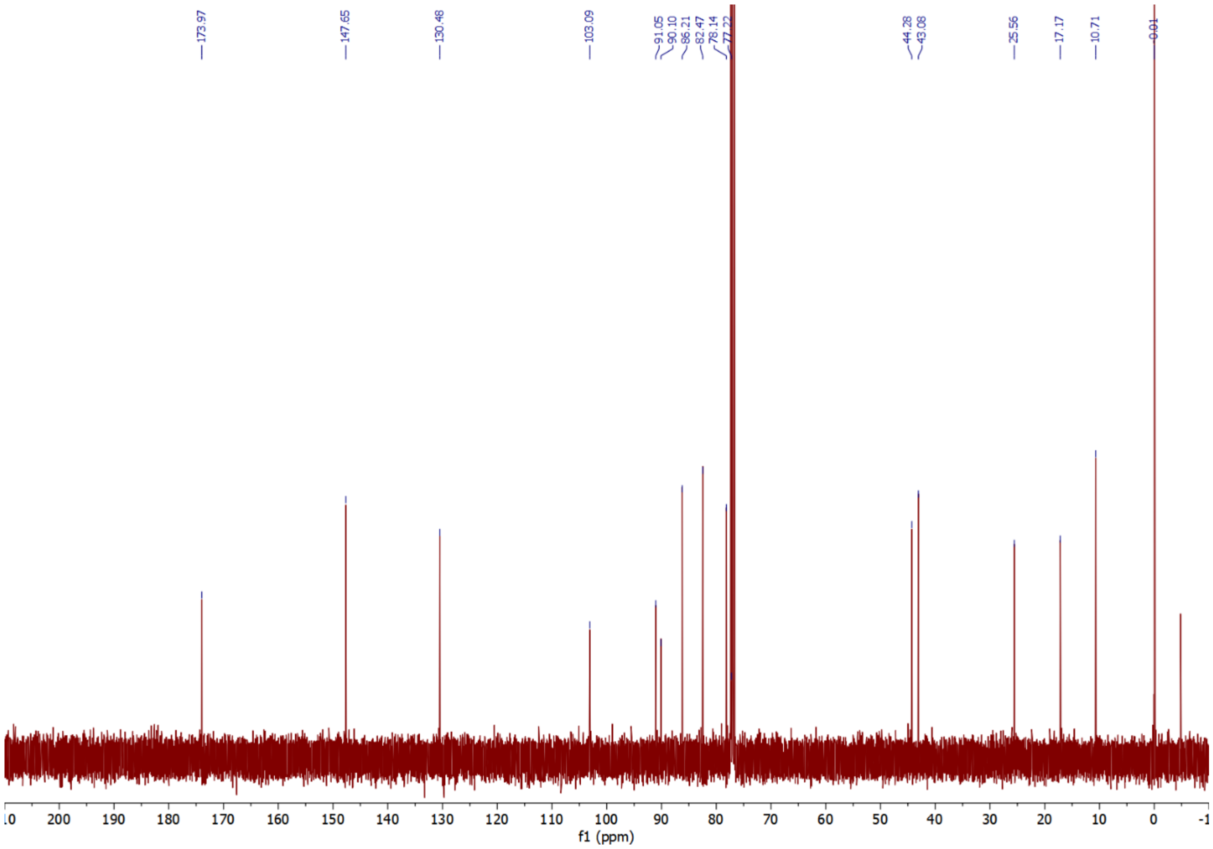

$^1H$–$^1H$ NOESY ($CDCl_3$)

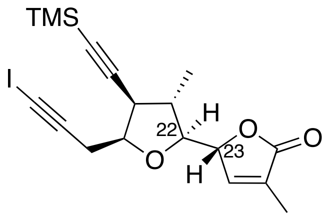


**8b** (C22*S*, C23*R*)

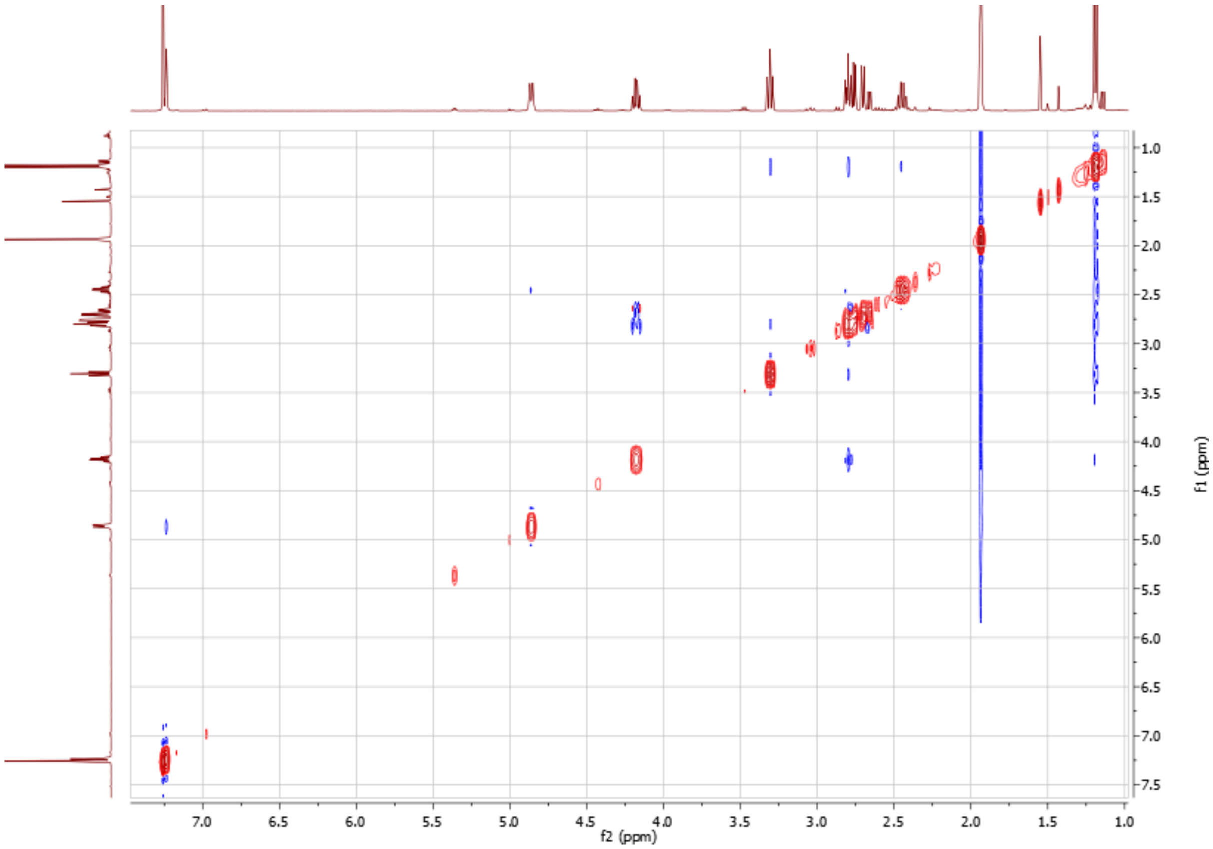

**(*R*)-5-((2*R*,3*S*,4*R*,5*S*)-5-(3-Iodoprop-2-yn-1-yl)-3-methyl-4-((trimethylsilyl)ethynyl)-tetrahydrofuran-2-yl)-3-methylfuran-2(5*H*)-one, 8c**

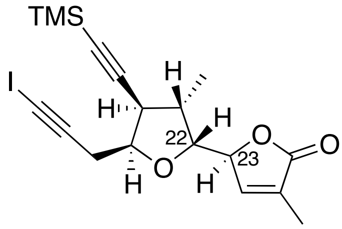


**8c** (C22*R*, C23*S*)

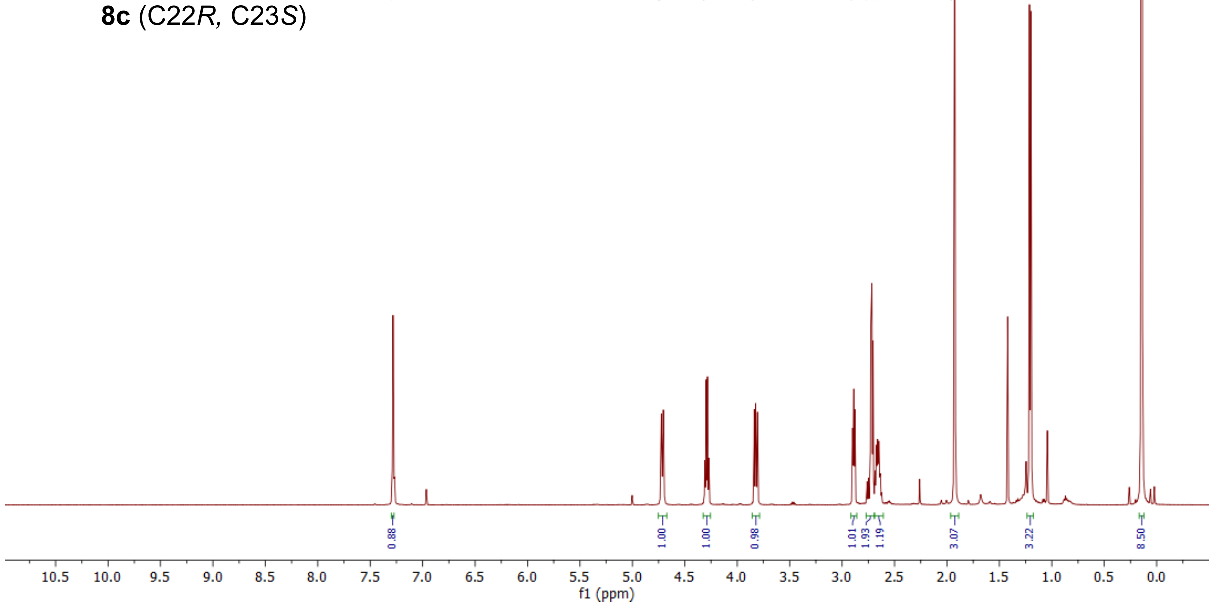


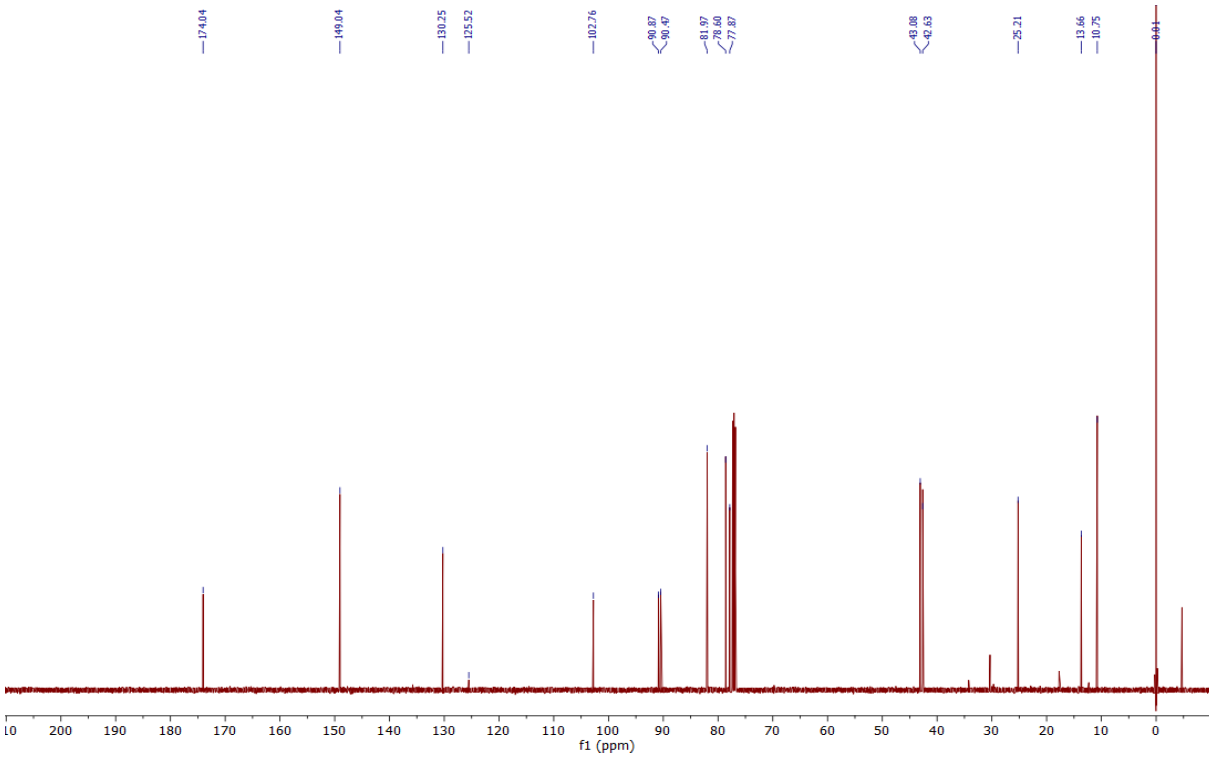

**$^{1}H$–$^{1}H$ NOESY ($CDCl_3$)**

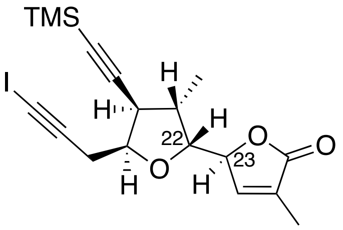


**8c** (C22*R*, C23*S*)

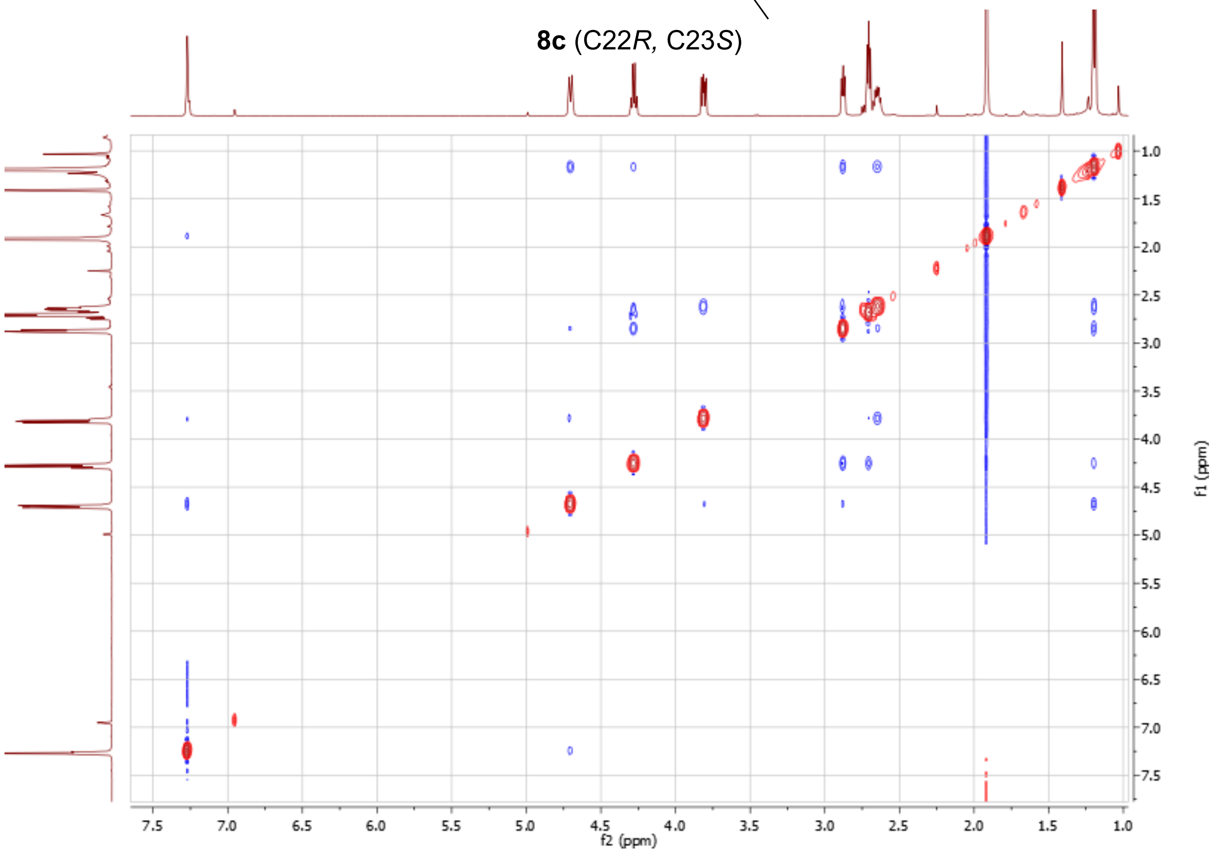

**(2*R*,3*S*,4*S*,5*R*)-5-(4-Hydroxydeca-2,9-diyn-1-yl)-3-methyl-4-((trimethylsilyl)ethynyl)tetrahydro furan-2-yl acetate; 27**

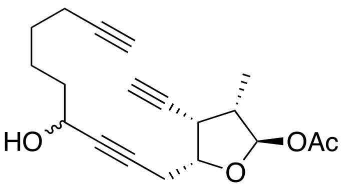


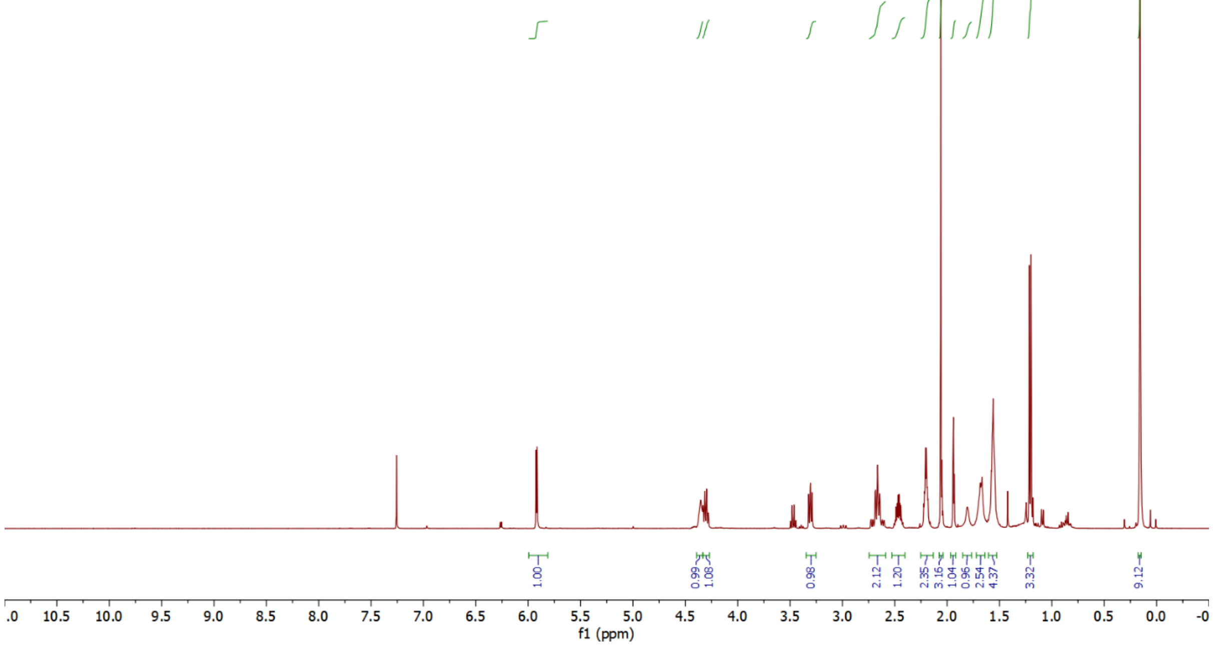


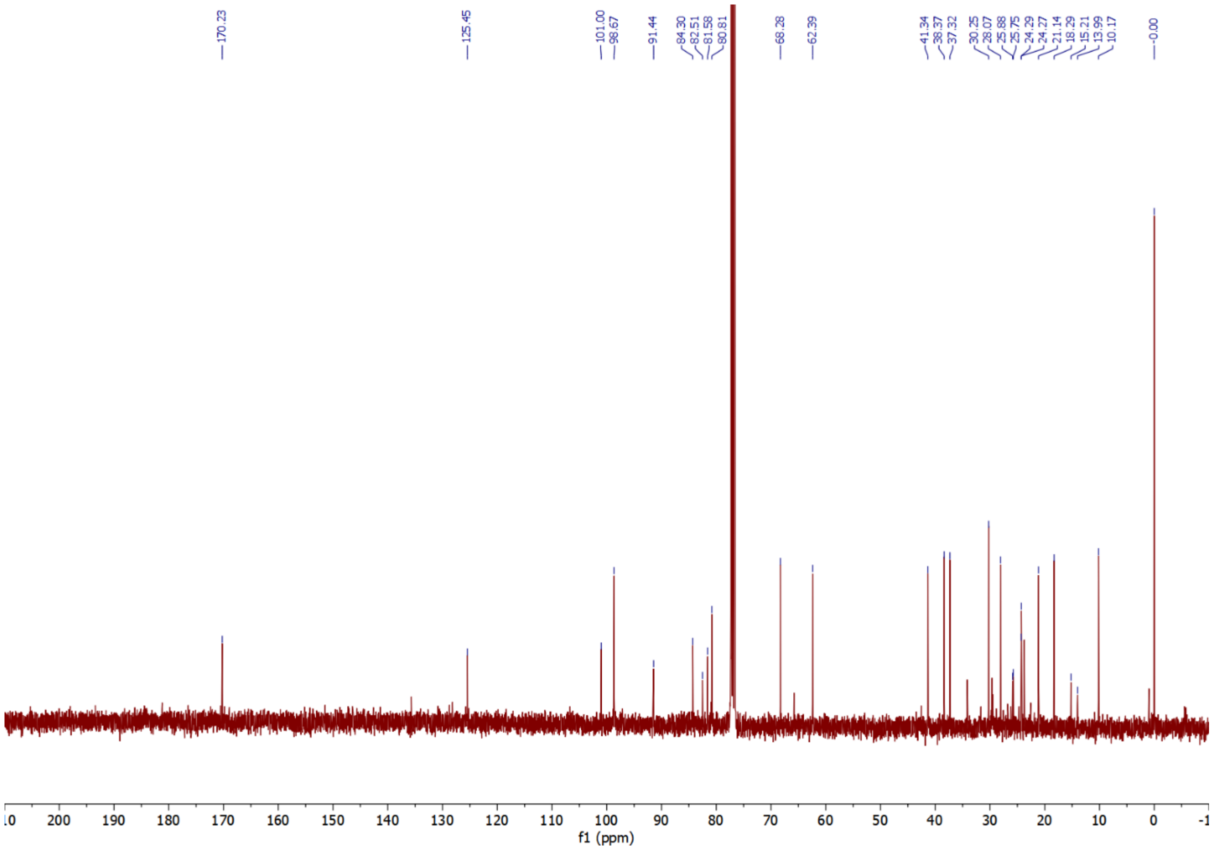

**(7b*S*,8*S*,9*R*,10a*R*)-1-Hydroxy-8-methyl-1,2,3,4,5,7b,8,9,10a,11-decahydrocyclohepta[4,5]indeno-[2,1-b]furan-9-yl acetate, 28**

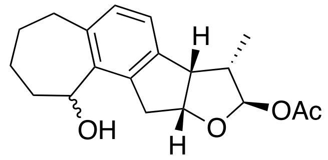


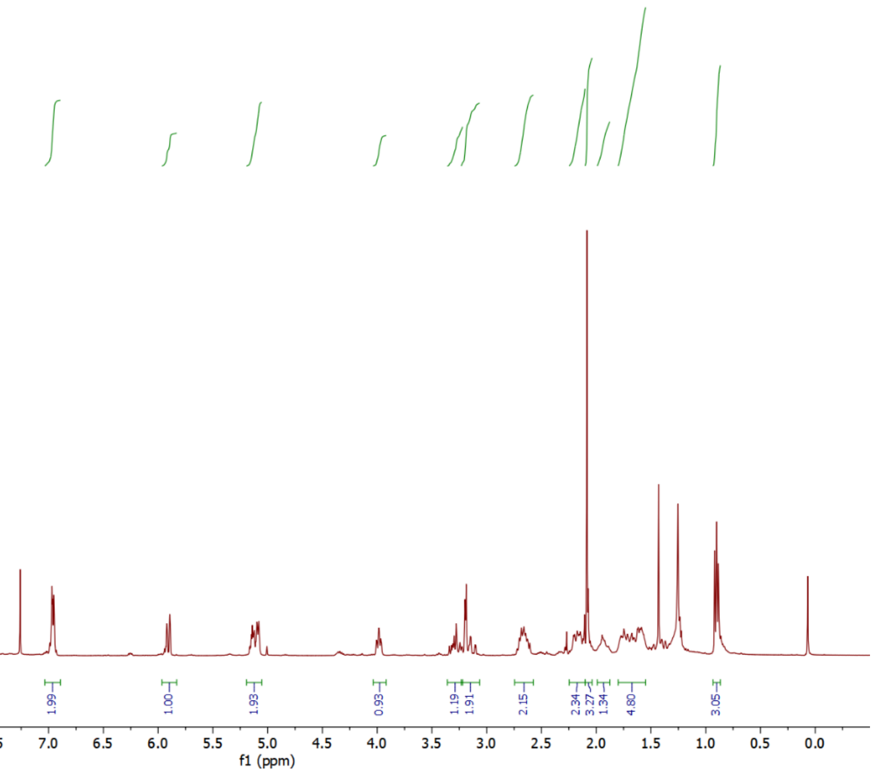


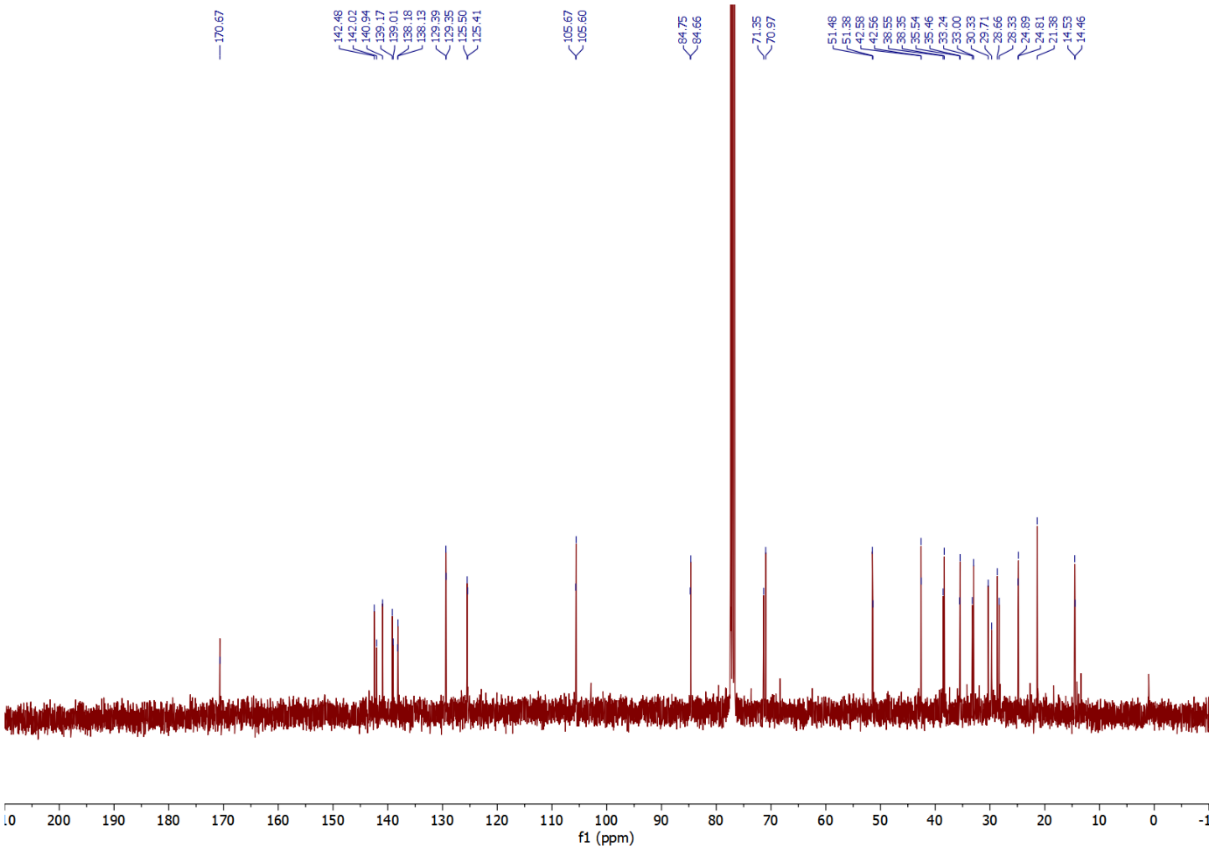

**(7b*S*,8*S*,9*R*,10a*R*)-8-Methyl-3,4,5,7b,8,9,10a,11-octahydrocyclohepta[4,5]indeno[2,1-b]furan-9-yl acetate, 29**

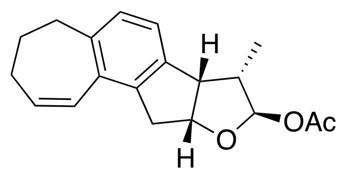


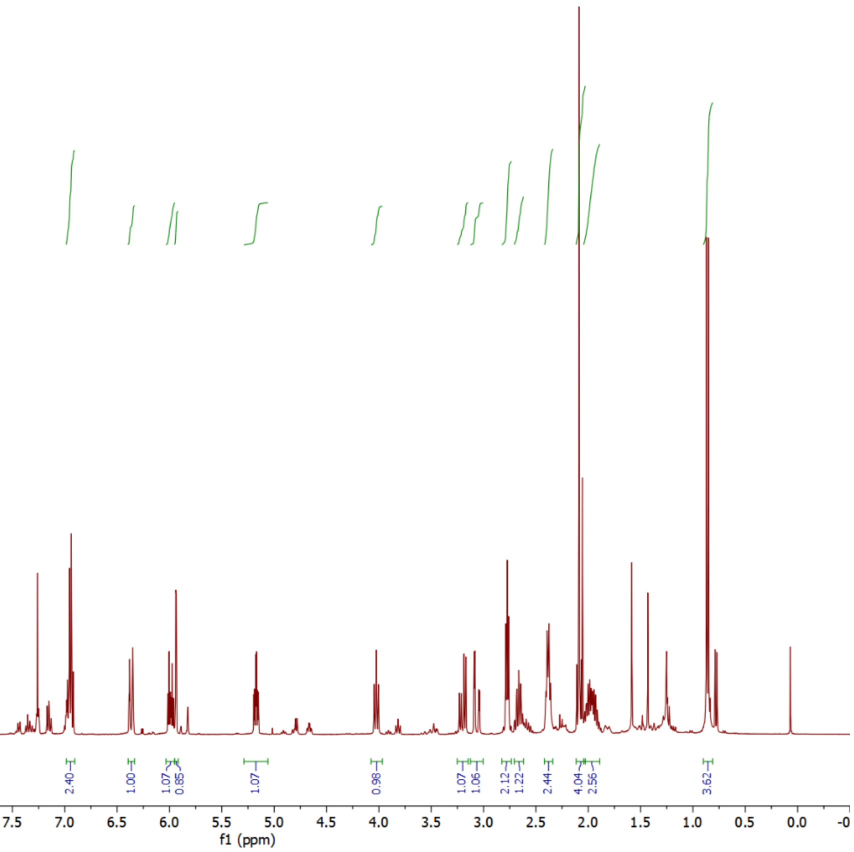

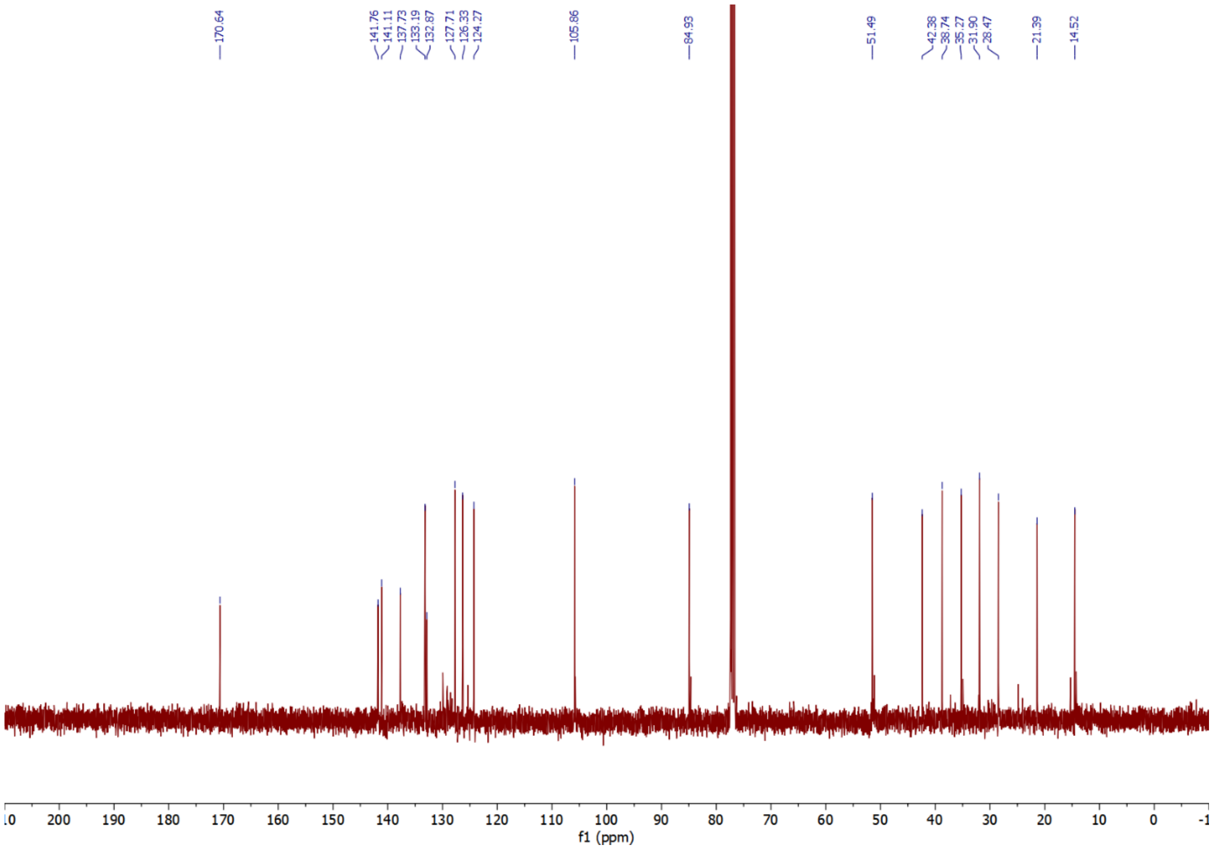

**(*S*)-3-Methyl-5-((7b*S*,8*S*,9*S*,10a*R*)-8-methyl-3,4,5,7b,8,9,10a,11-octahydrocyclohepta[4,5]indeno-[2,1-b]furan-9-yl)furan-2(5*H*)-one, 30**

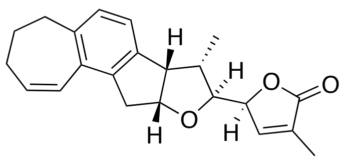

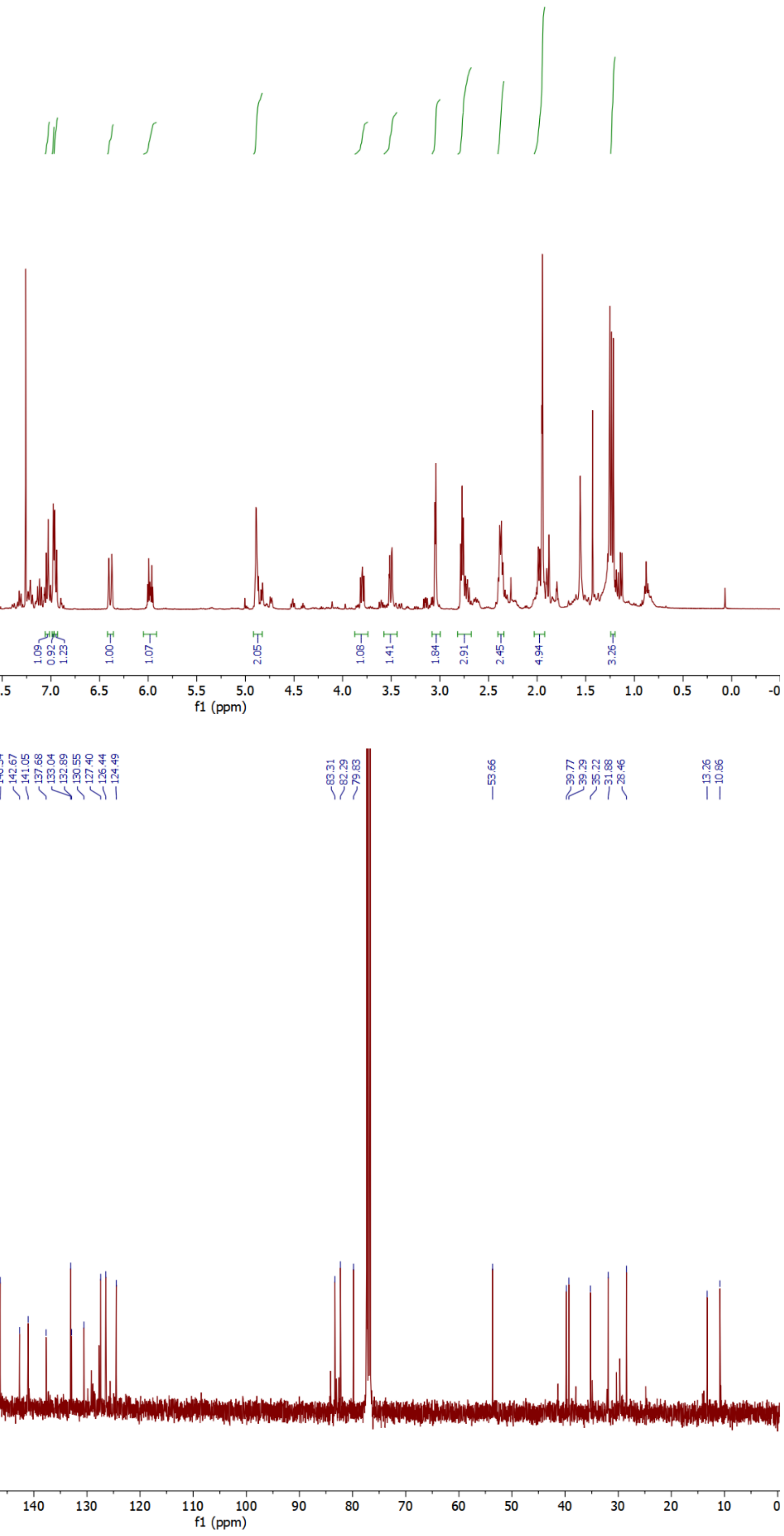

**(2*R*,3*S*,4*S*,5*R*)-5-(5-((3*S*,3a*R*,6a*R*)-2,2-Dimethyl-5-oxo-3a-(prop-2-yn-1-yl)hexahydrofuro[3,2-b]-furan-3-yl)-4-hydroxypent-2-yn-1-yl)-3-methyl-4-((trimethylsilyl)ethynyl)tetrahydrofuran-2-yl acetate, S17**

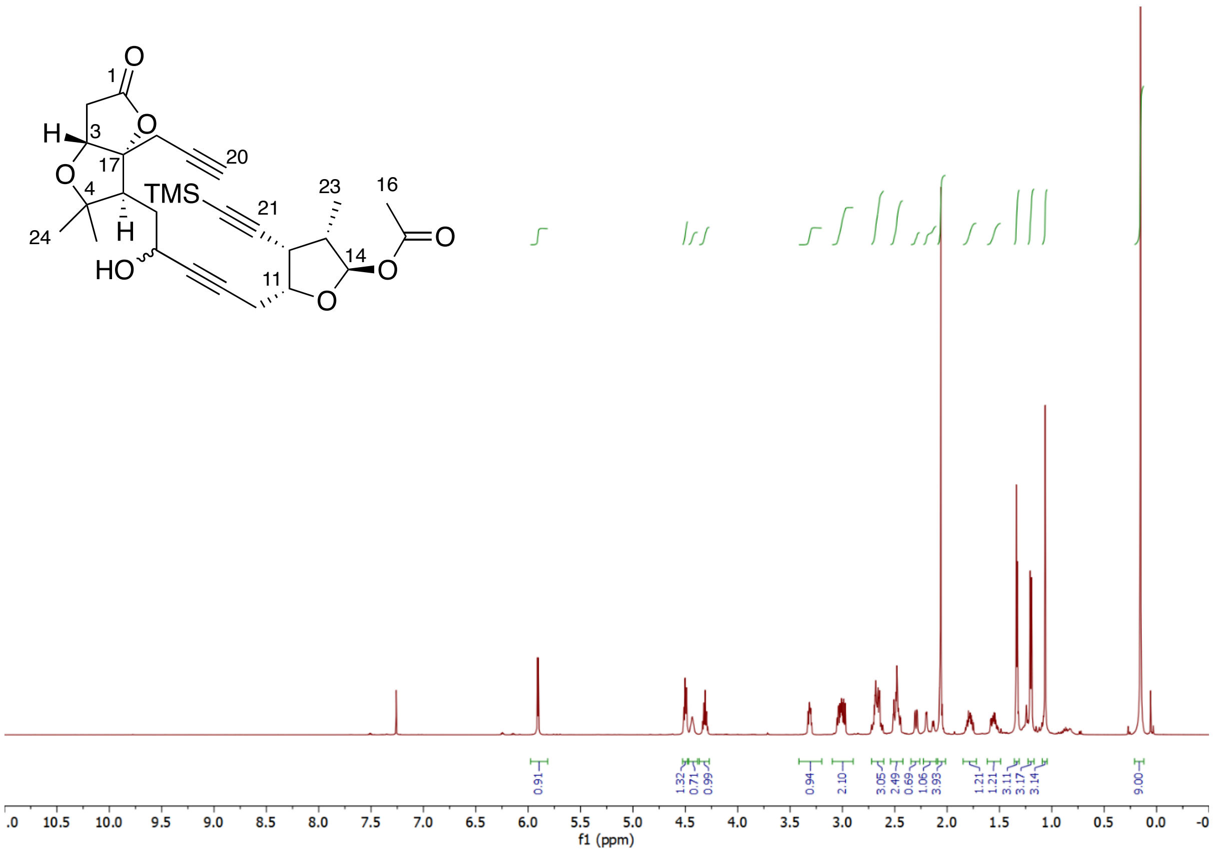


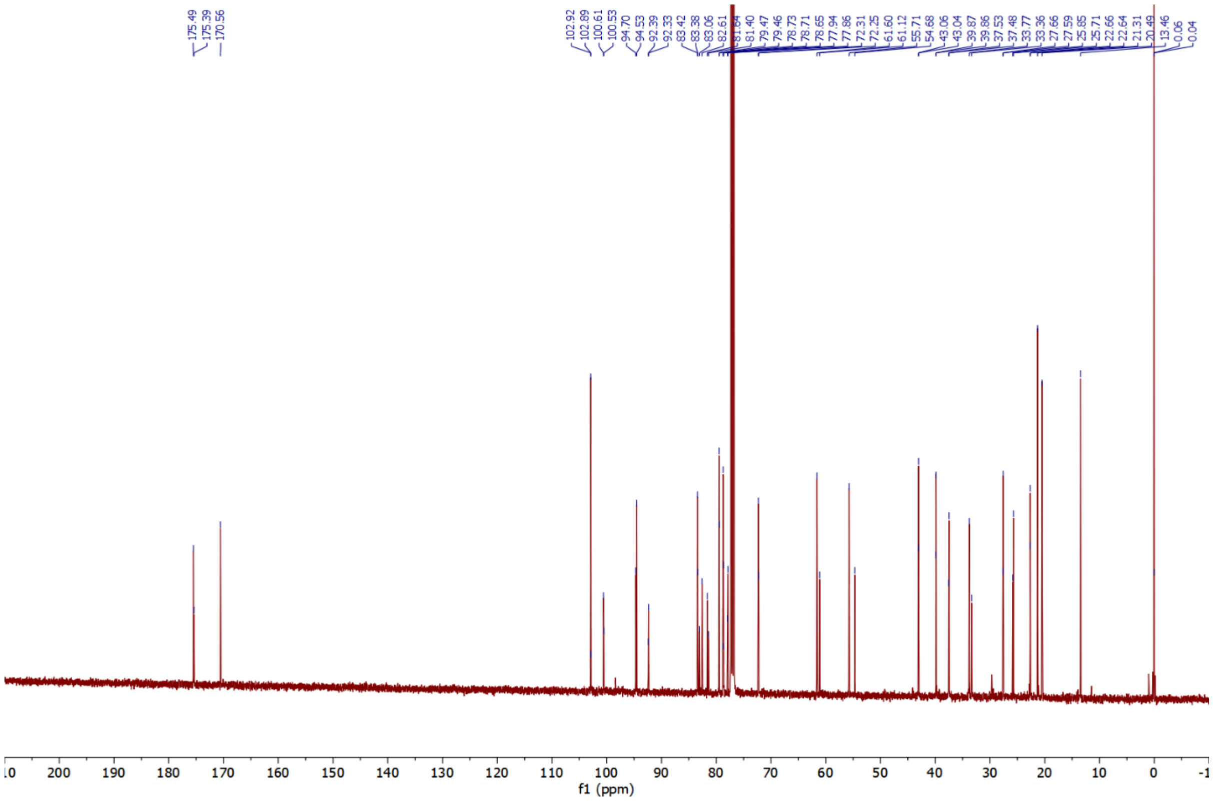

**(2*R*,3*S*,4*S*,5*R*)-5-(5-((3S,3a*R*,6a*R*)-2,2-Dimethyl-5-oxo-3a-(prop-2-yn-1-yl)hexahydrofuro[3,2-b]-furan-3-yl)-4-hydroxypent-2-yn-1-yl)-4-ethynyl-3-methyltetrahydrofuran-2-yl acetate, 31**

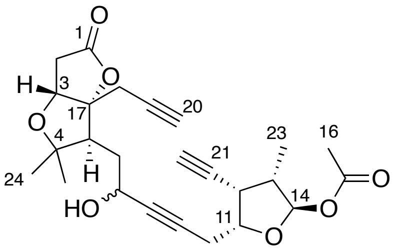

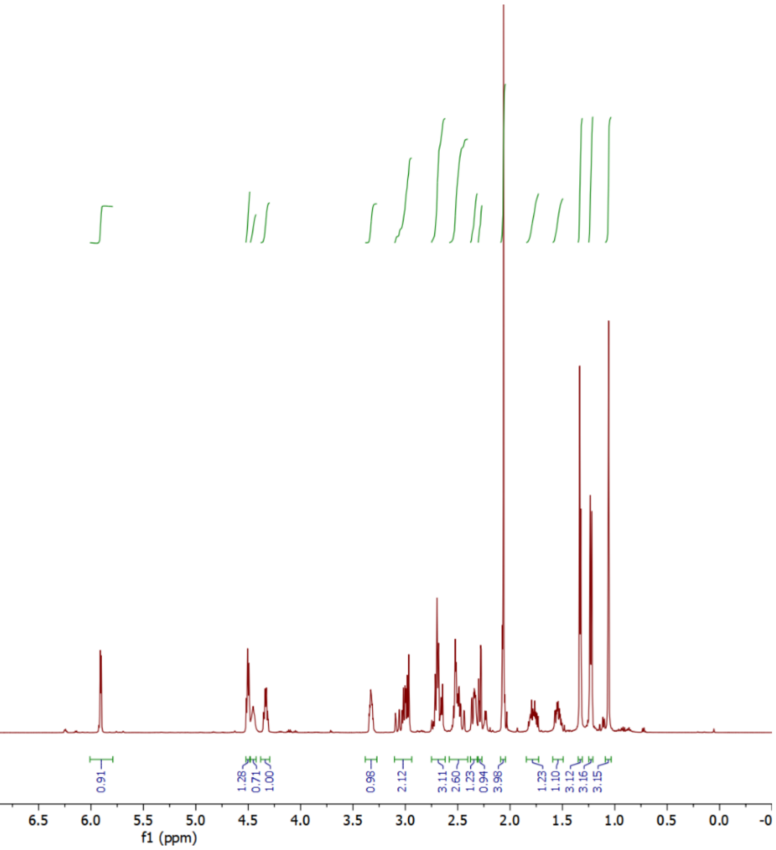

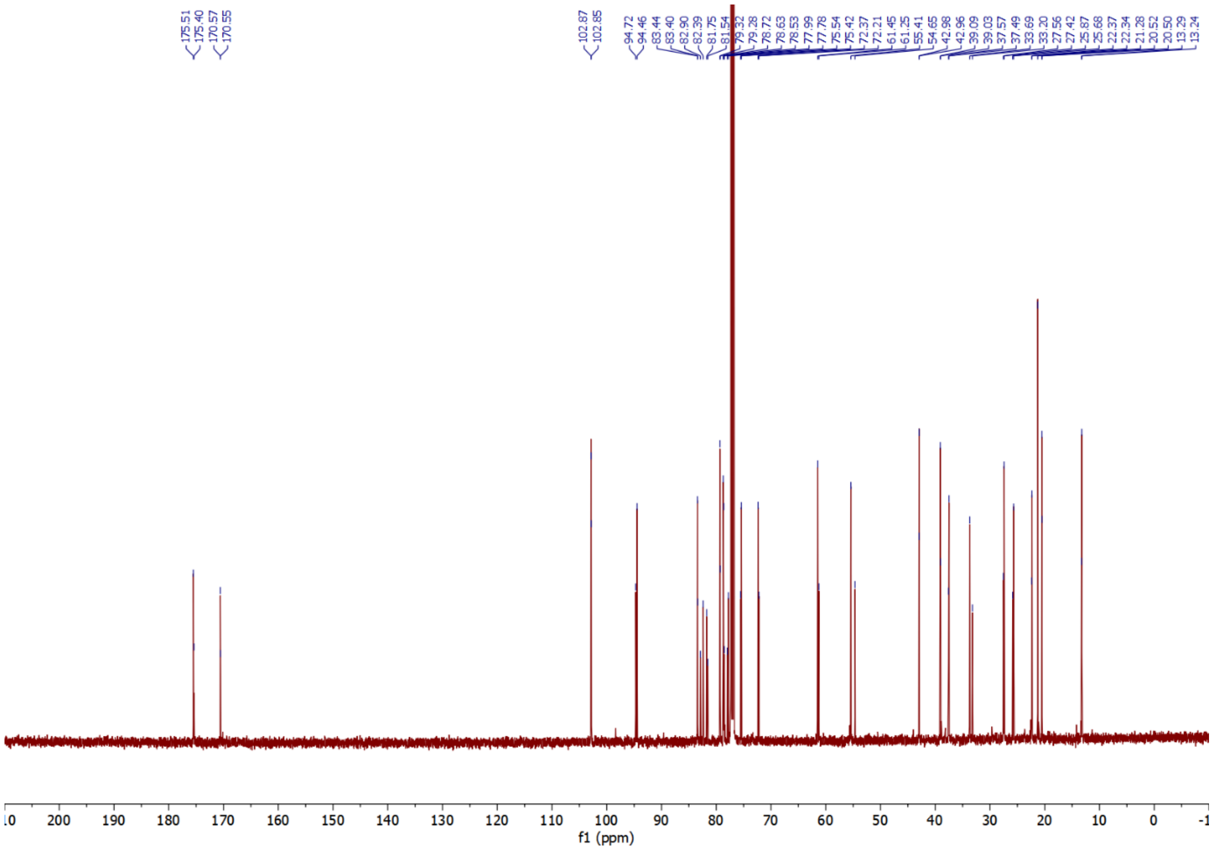

**ABCDEF hexacycle 32**

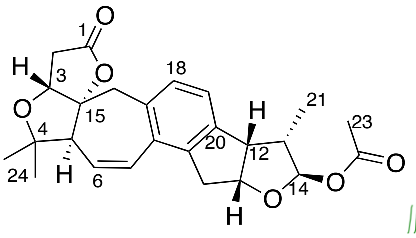


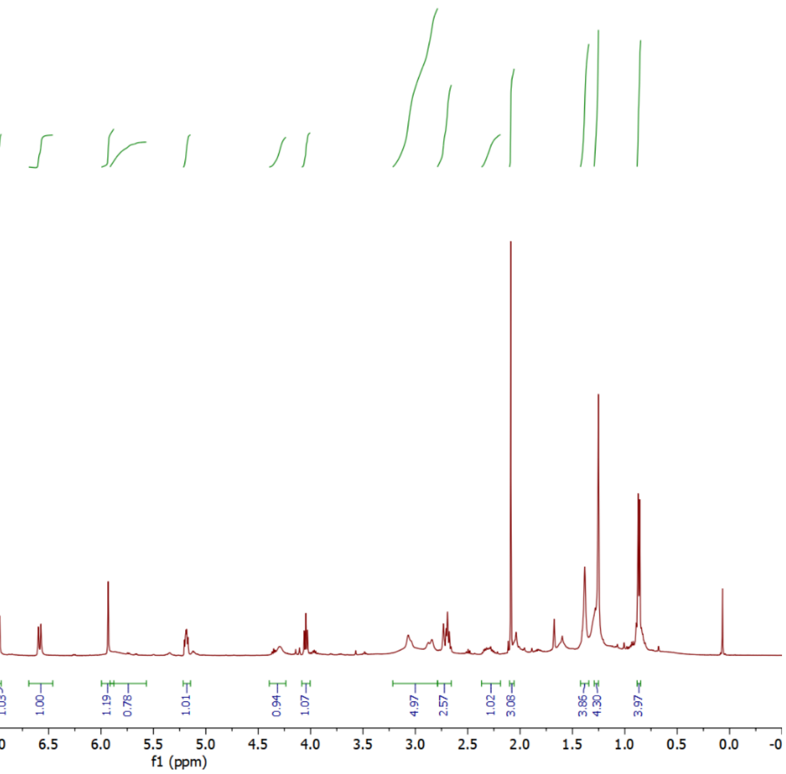


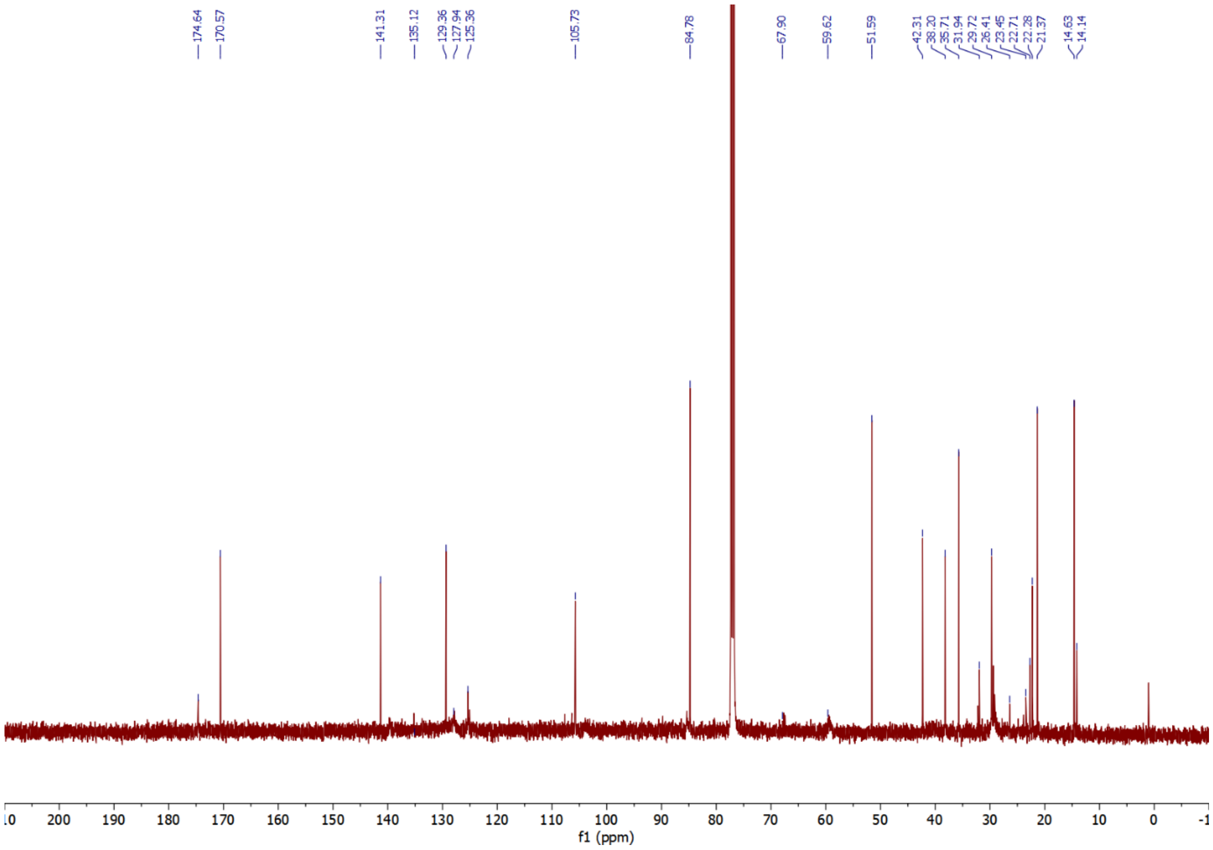

**ABCDEF sulfurane adduct 33**

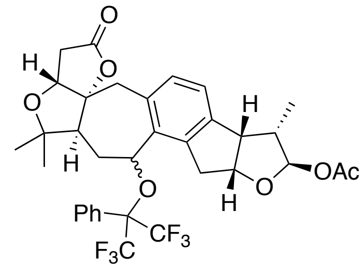


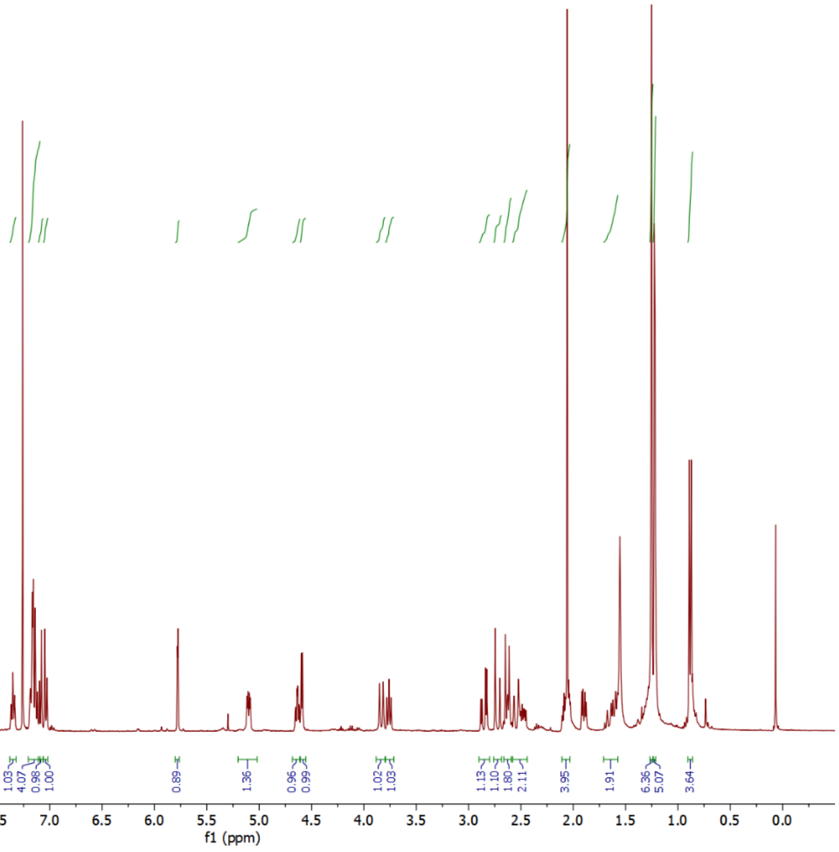


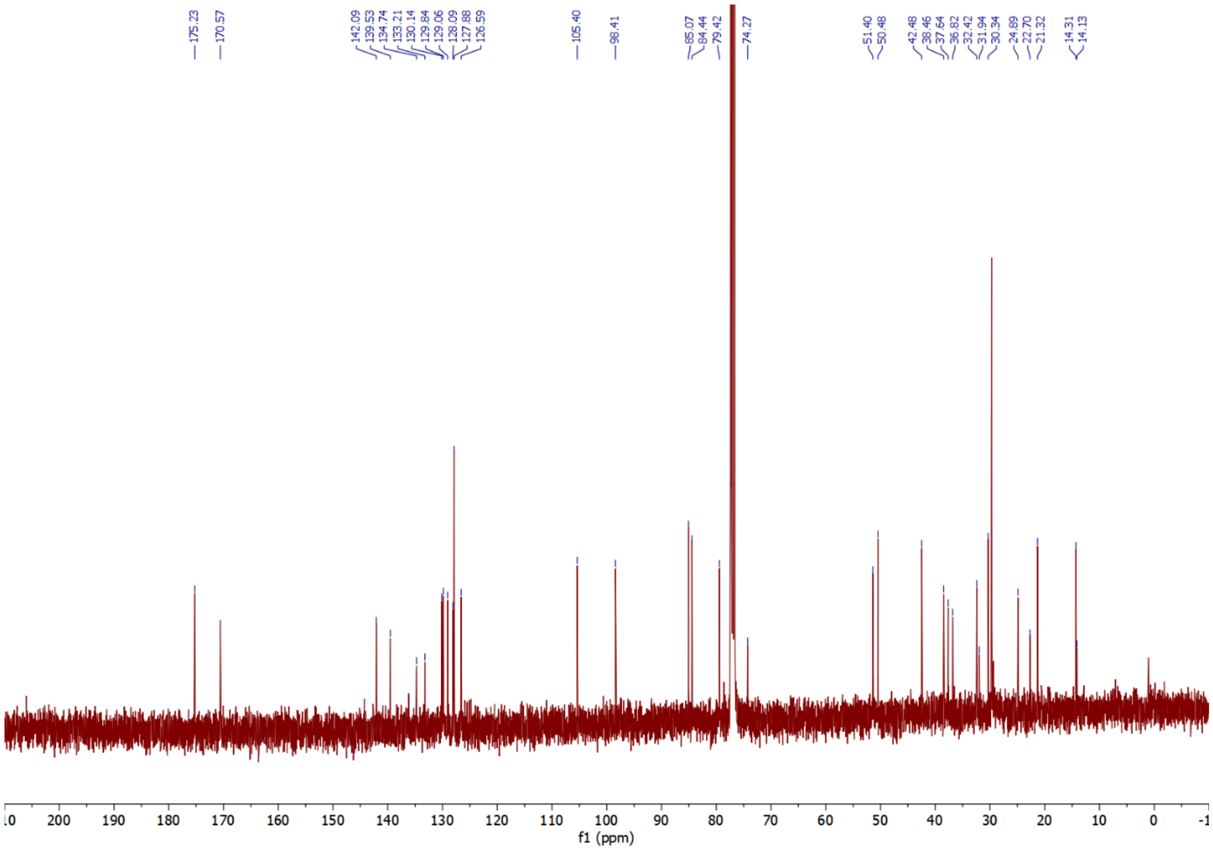

**(3a*R*,6*S*,6a*R*)-6-(2-Hydroxy-5-((2*R*,3*S*,4*S*,5*S*)-4-methyl-5-((*S*)-4-methyl-5-oxo-2,5-dihydrofuran-2-yl)-3-((trimethylsilyl)ethynyl)tetrahydrofuran-2-yl)pent-3-yn-1-yl)-5,5-dimethyl-6a-(prop-2-yn-1-yl)tetrahydrofuro[3,2-b]furan-2(3*H*)-one, S18**

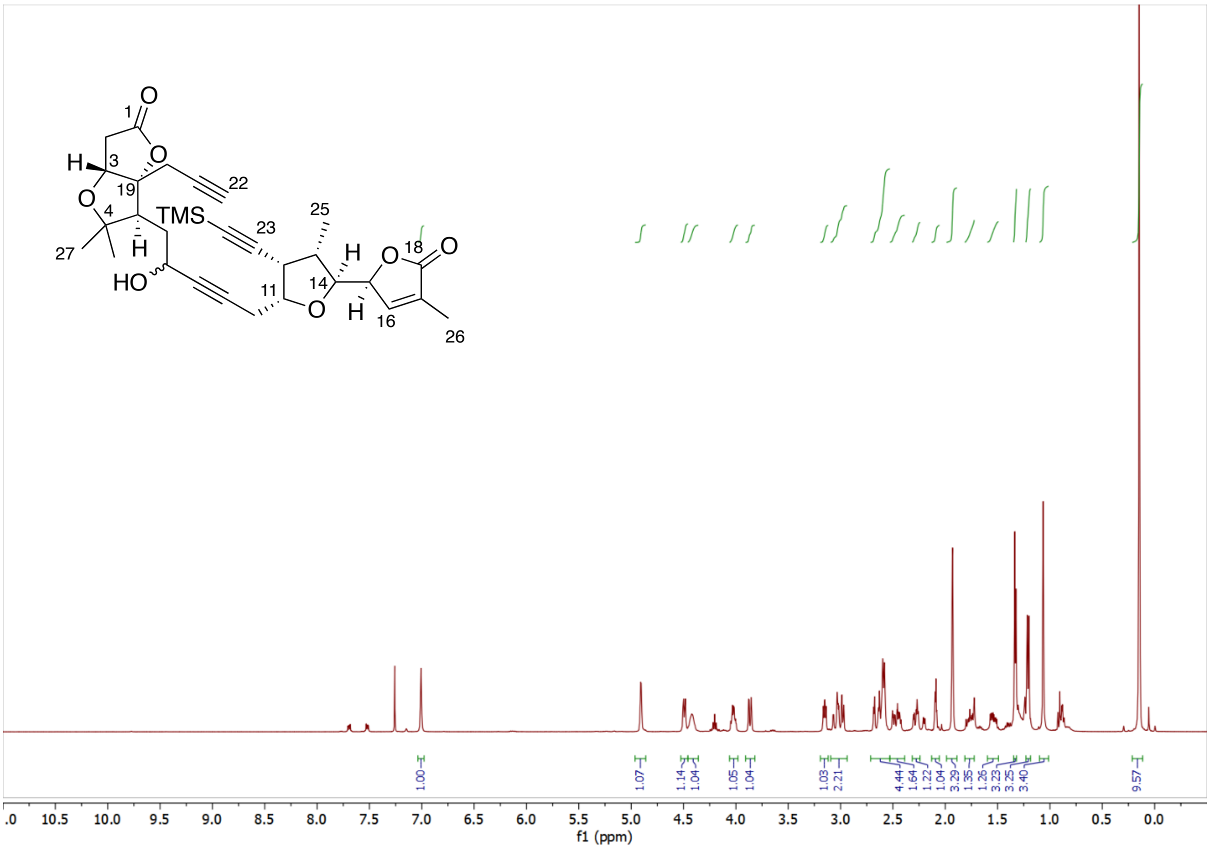


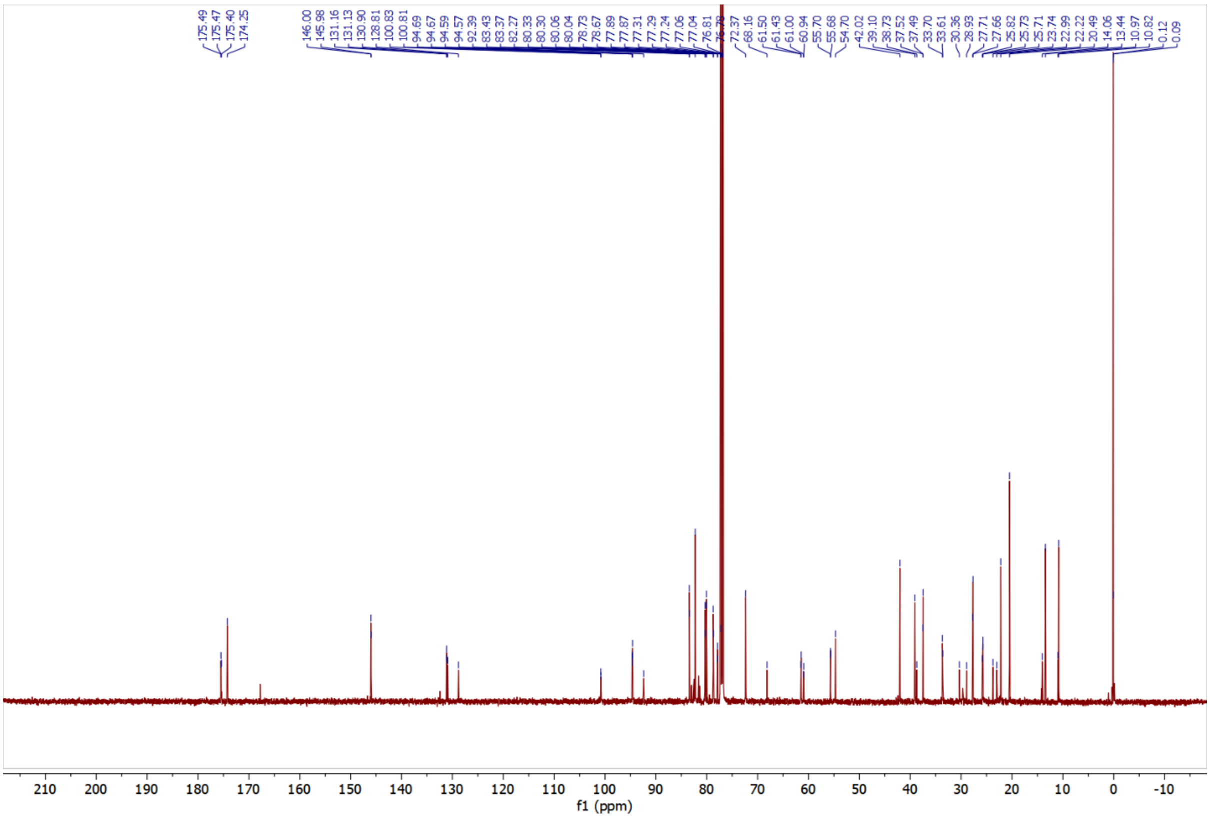

**(3a*R*,6*S*,6a*R*)-6-(5-((2*R*,3*S*,4*S*,5*S*)-3-Ethynyl-4-methyl-5-((*S*)-4-methyl-5-oxo-2,5-dihydrofuran-2-yl)tetrahydrofuran-2-yl)-2-hydroxypent-3-yn-1-yl)-5,5-dimethyl-6a-(prop-2-yn-1-yl)tetrahydro-furo[3,2-b]furan-2(3*H*)-one, 34**

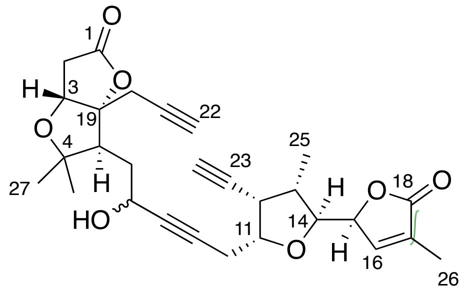

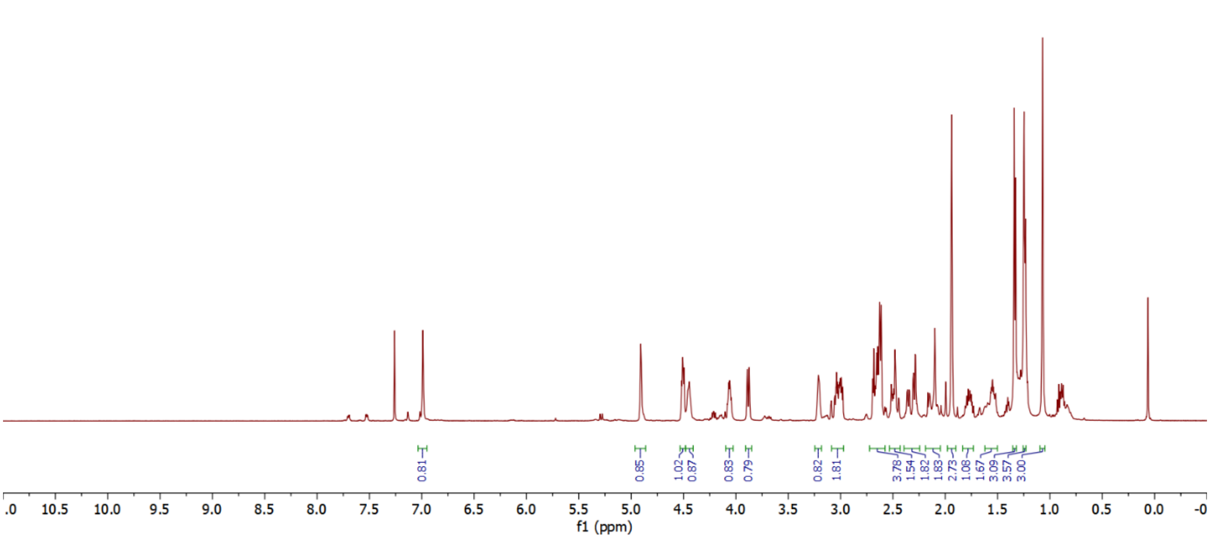

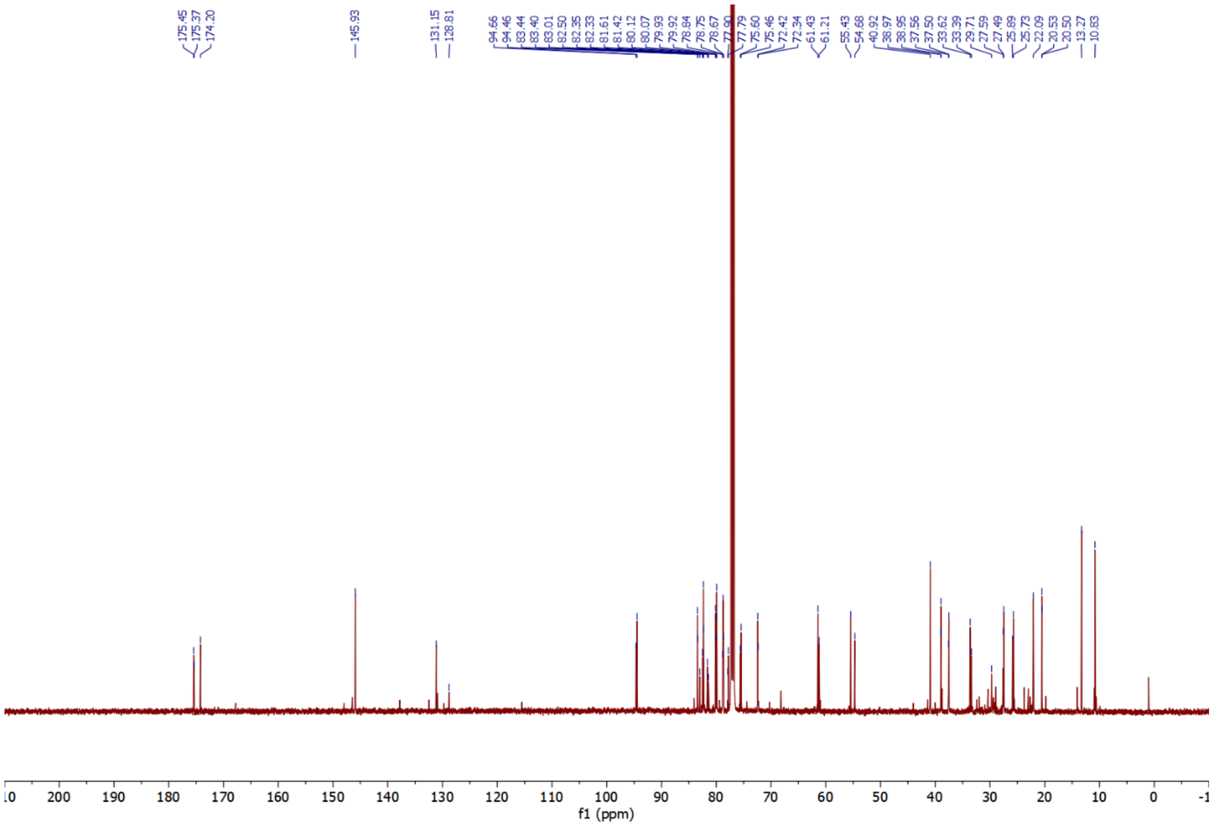

## Rubriflordilactone B, 1

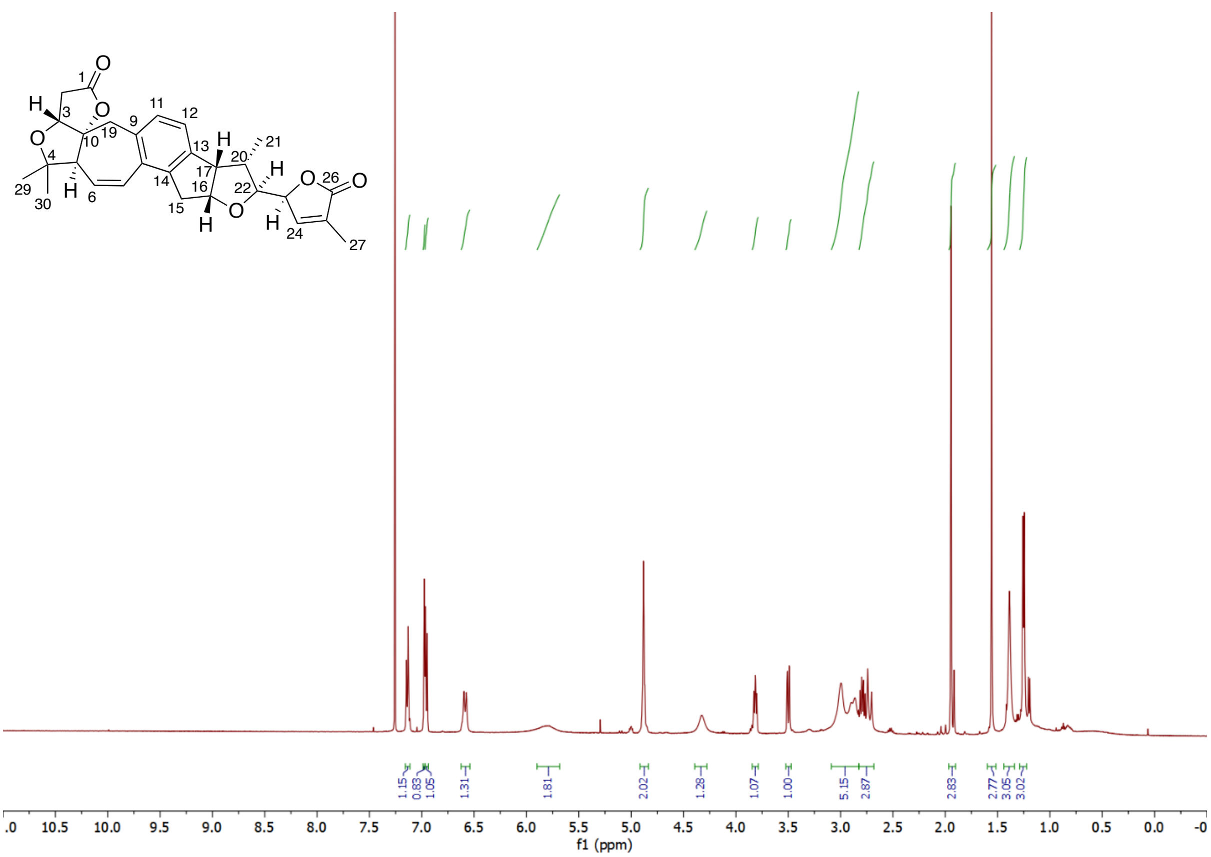

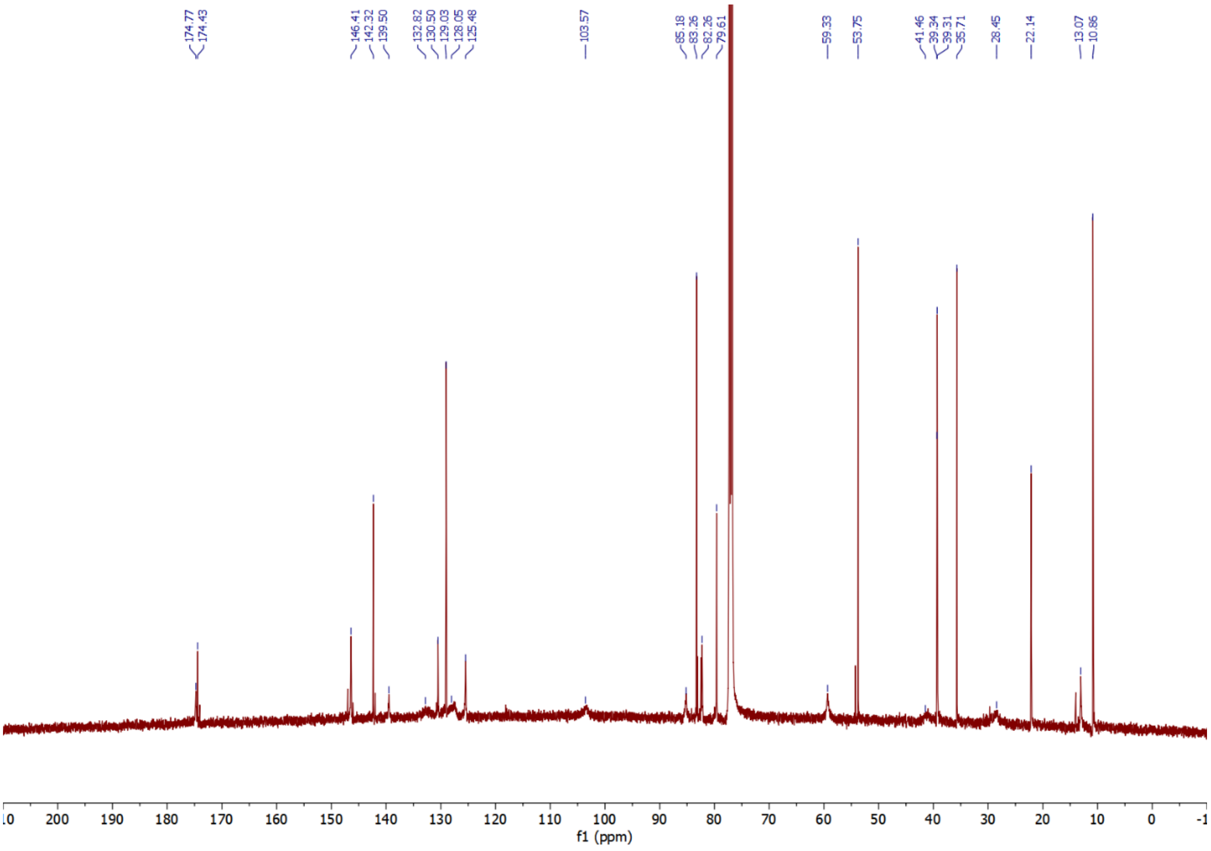

**(3a*R*,6*S*,6a*R*)-6-(2-Hydroxy-5-((2*R*,3*S*,4*S*,5*S*)-4-methyl-5-((*R*)-4-methyl-5-oxo-2,5-dihydrofuran-2-yl)-3-((trimethylsilyl)ethynyl)tetrahydrofuran-2-yl)pent-3-yn-1-yl)-5,5-dimethyl-6a-(prop-2-yn-1-yl)tetrahydrofuro[3,2-b]furan-2(3*H*)-one, S19**

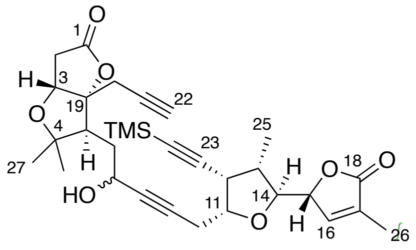

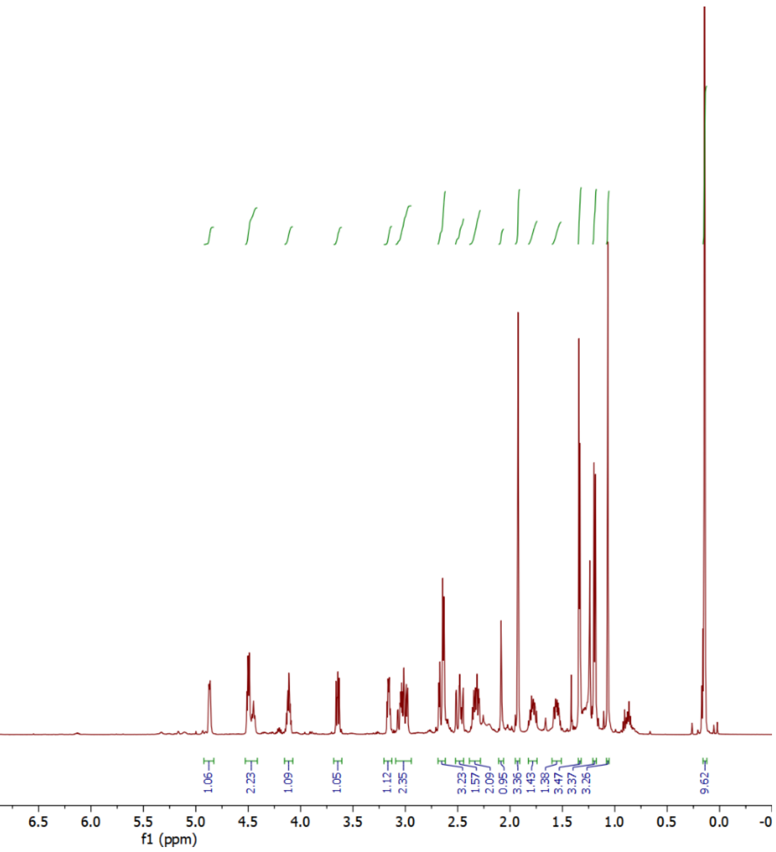

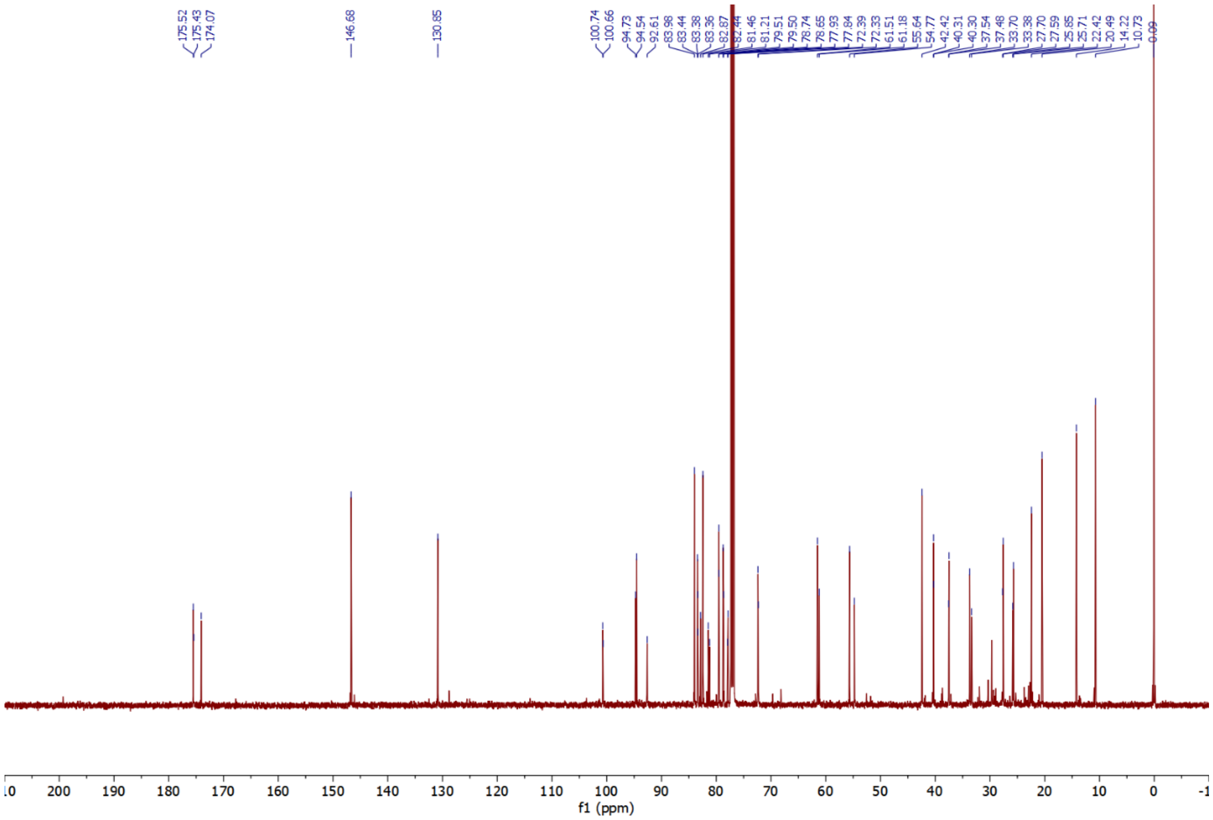

**(3a*R*,6*S*,6a*R*)-6-(5-((2*R*,3*S*,4*S*,5*S*)-3-Ethynyl-4-methyl-5-((*R*)-4-methyl-5-oxo-2,5-dihydrofuran-2-yl)tetrahydrofuran-2-yl)-2-hydroxypent-3-yn-1-yl)-5,5-dimethyl-6a-(prop-2-yn-1-yl)tetrahydrofuro[3,2-b]furan-2(3*H*)-one, 35**

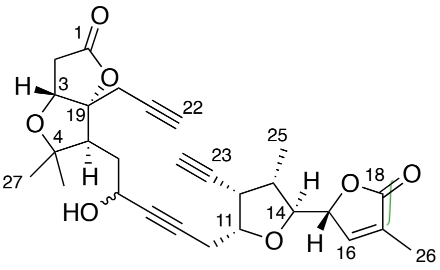

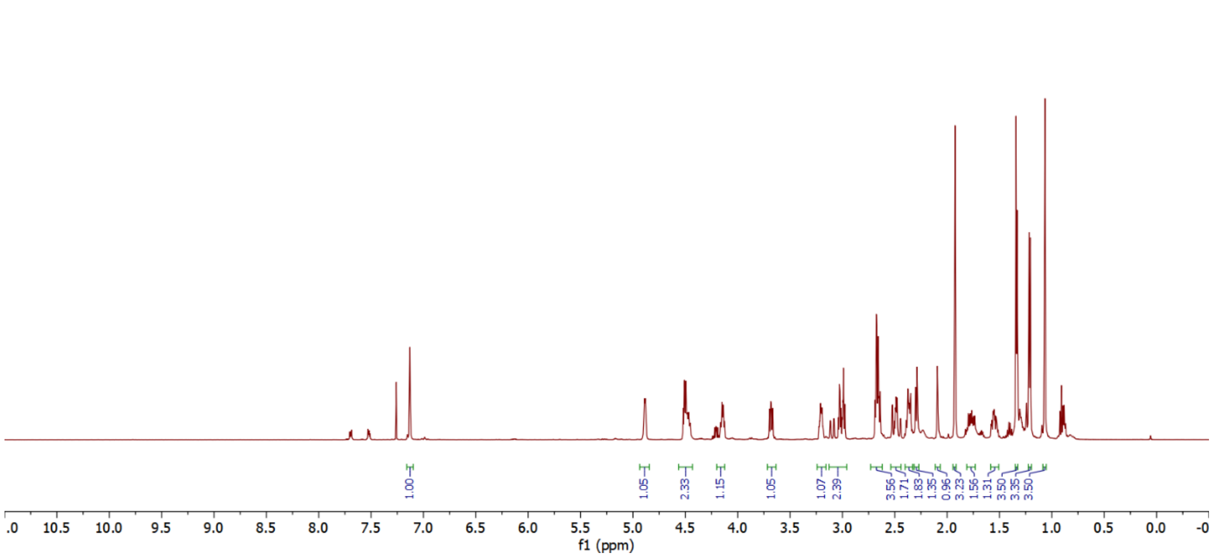


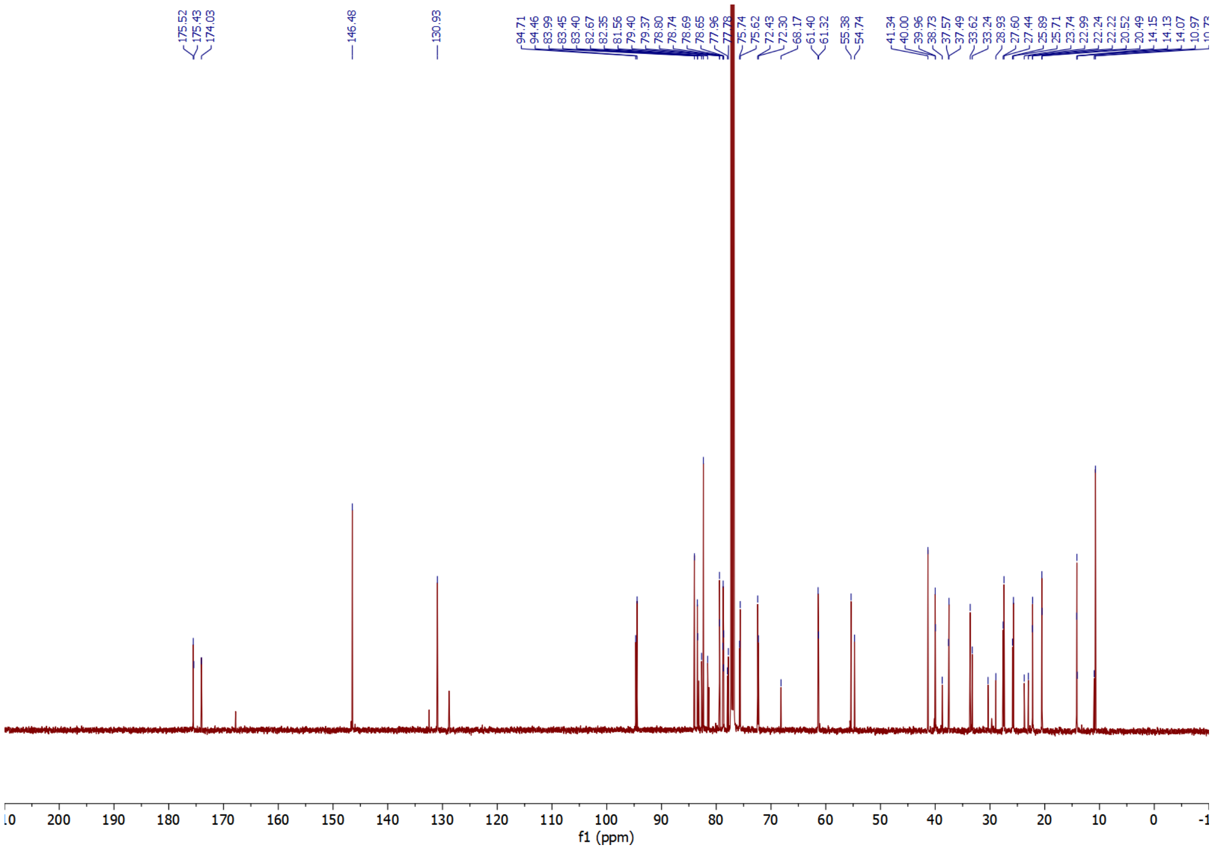

***Epi*-rubriflordilactone B, *epi*-1**

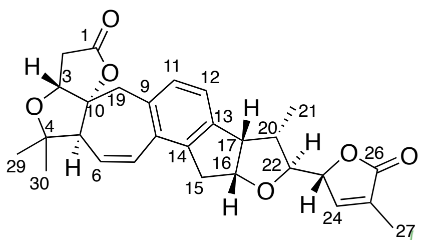

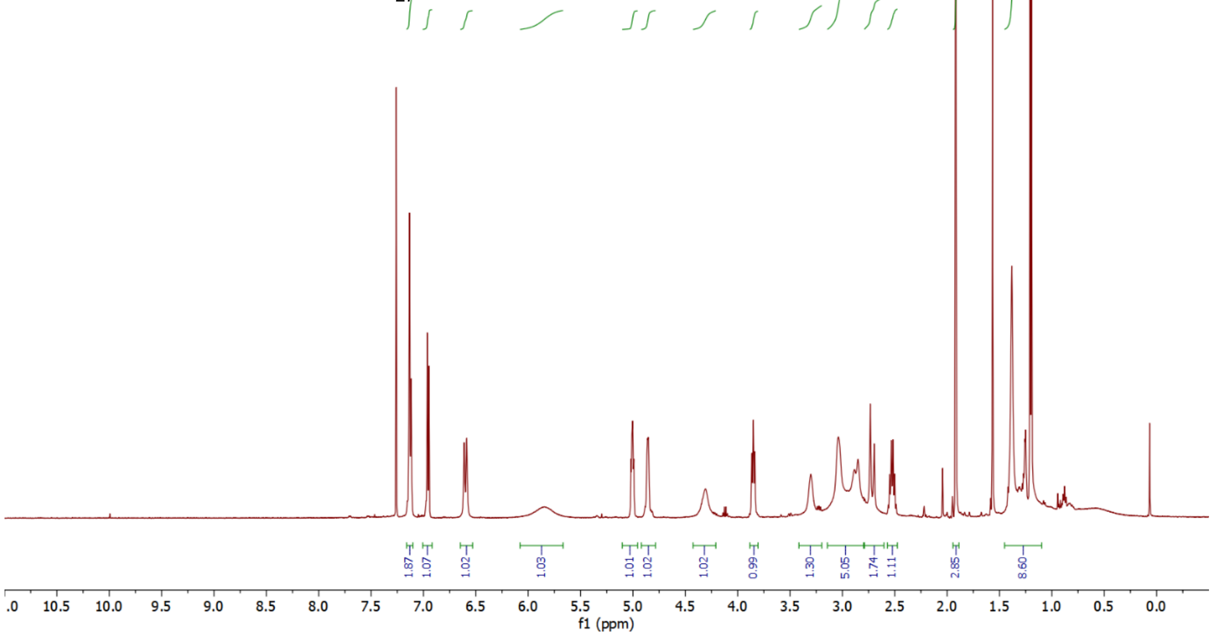


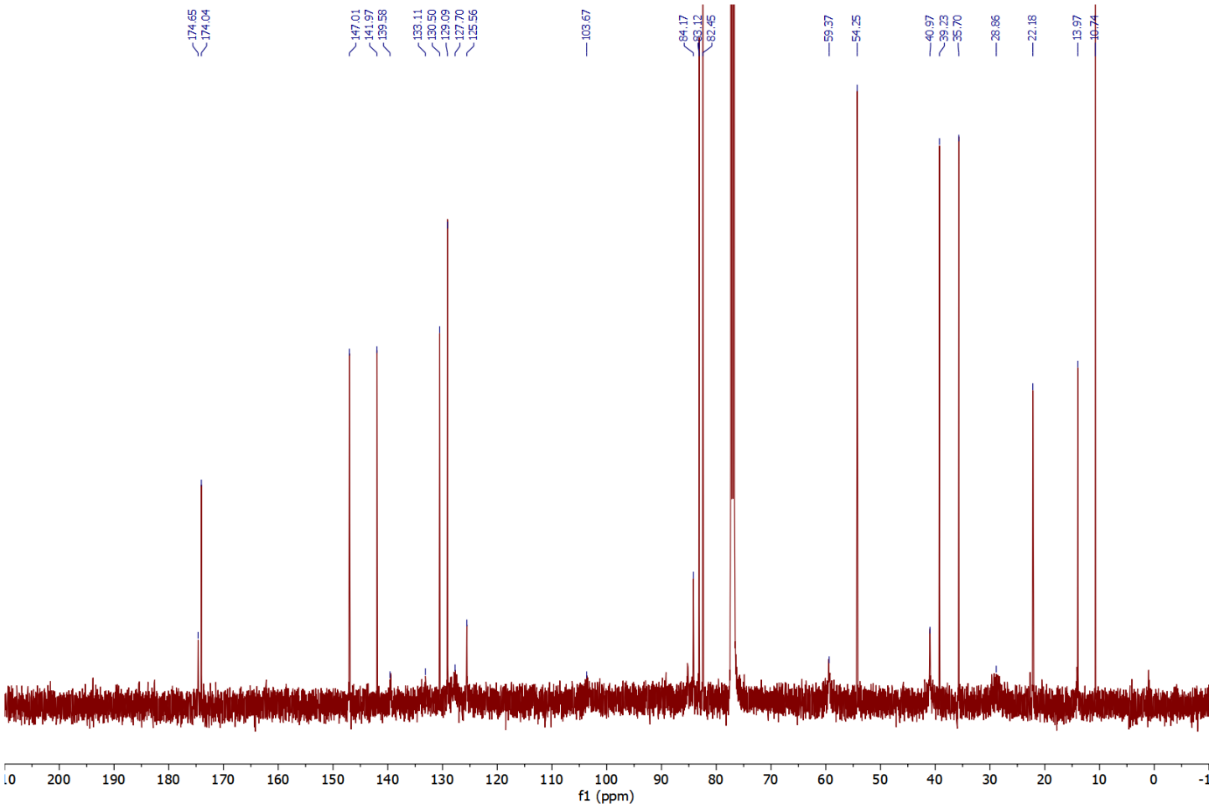

**(3a*R*,6*S*,6a*R*)-6-(2-Hydroxy-5-((2*S*,3*R*,4*S*,5*S*)-4-methyl-5-((*S*)-4-methyl-5-oxo-2,5-dihydrofuran-2-yl)-3-((trimethylsilyl)ethynyl)tetrahydrofuran-2-yl)pent-3-yn-1-yl)-5,5-dimethyl-6a-(prop-2-yn-1-yl)tetrahydrofuro[3,2-b]furan-2(3*H*)-one, S20**

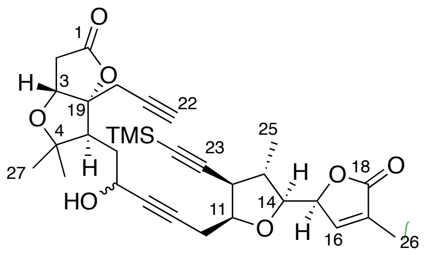

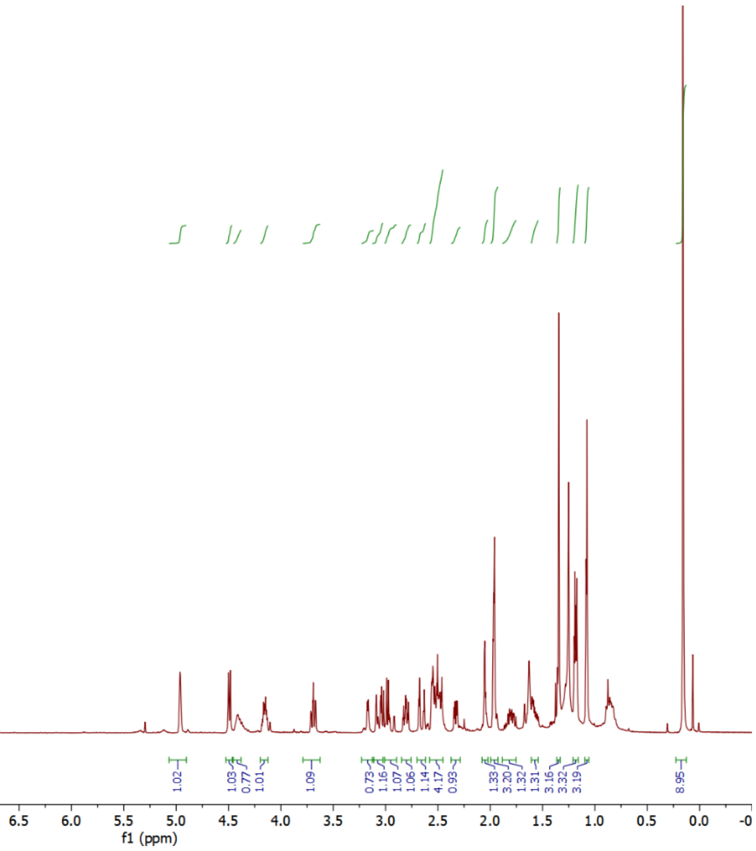


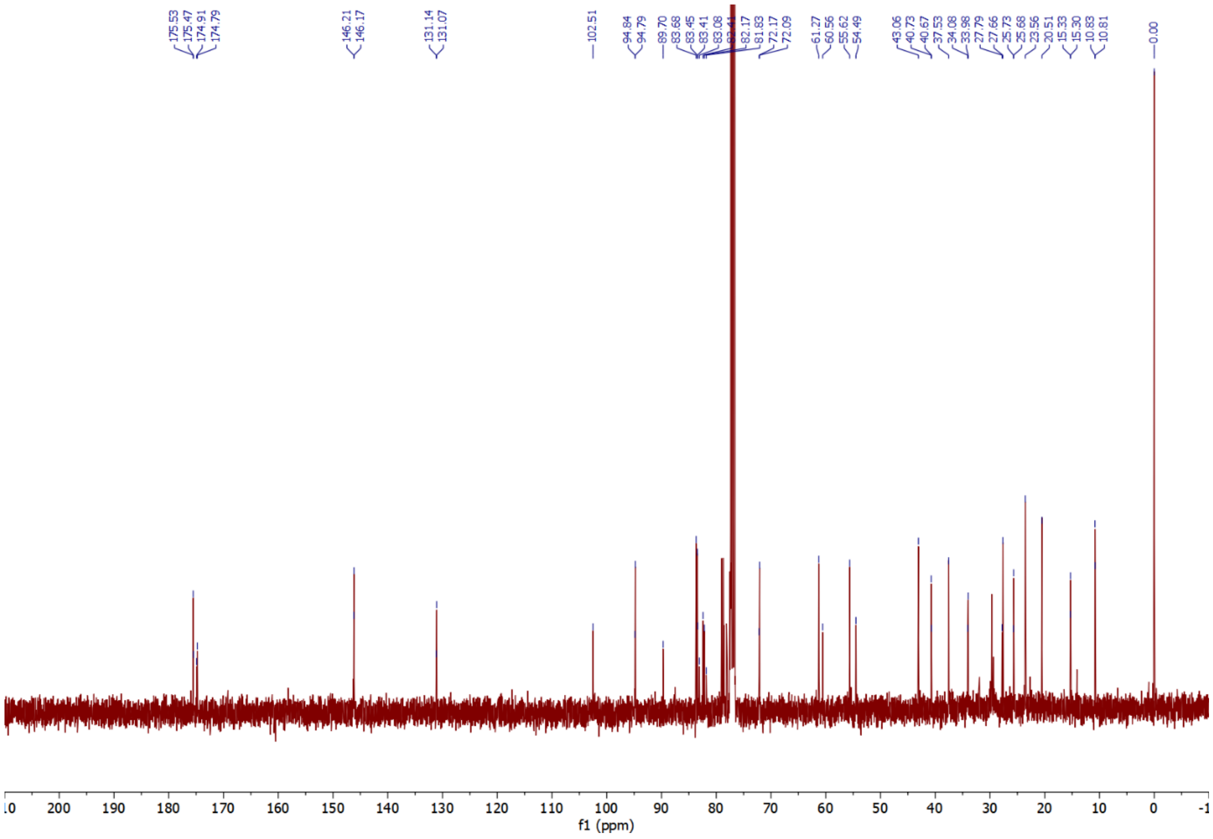

**(3a*R*,6*S*,6a*R*)-6-(5-((2*S*,3*R*,4*S*,5*S*)-3-Ethynyl-4-methyl-5-((*S*)-4-methyl-5-oxo-2,5-dihydrofuran-2-yl)tetrahydrofuran-2-yl)-2-hydroxypent-3-yn-1-yl)-5,5-dimethyl-6a-(prop-2-yn-1-yl)tetrahydrofuro[3,2-b]furan-2(3*H*)-one, 36**

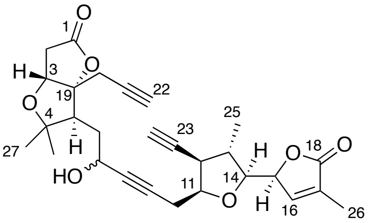

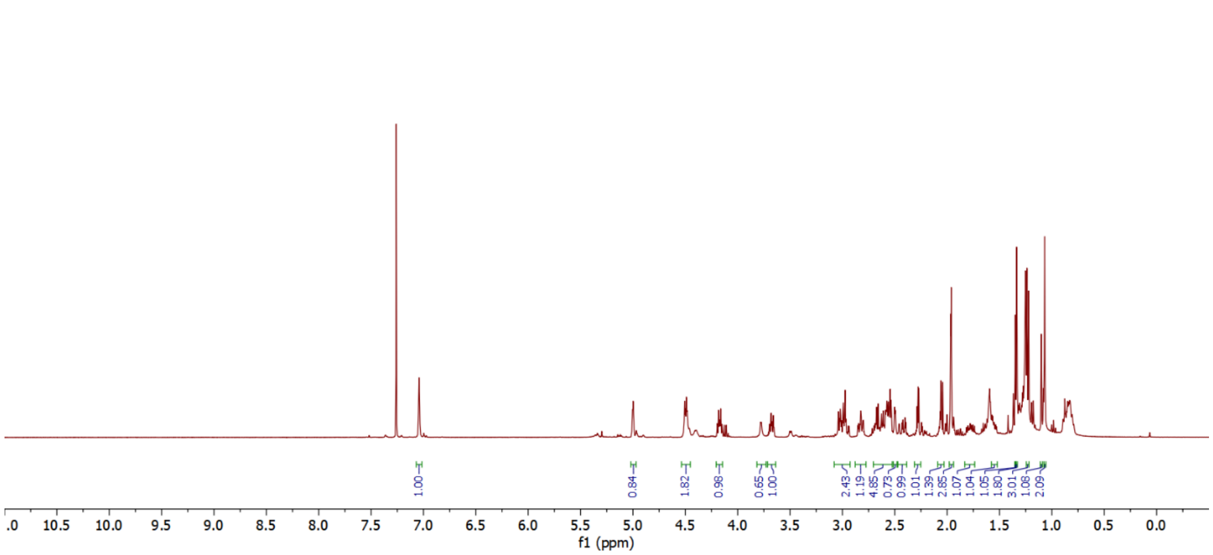

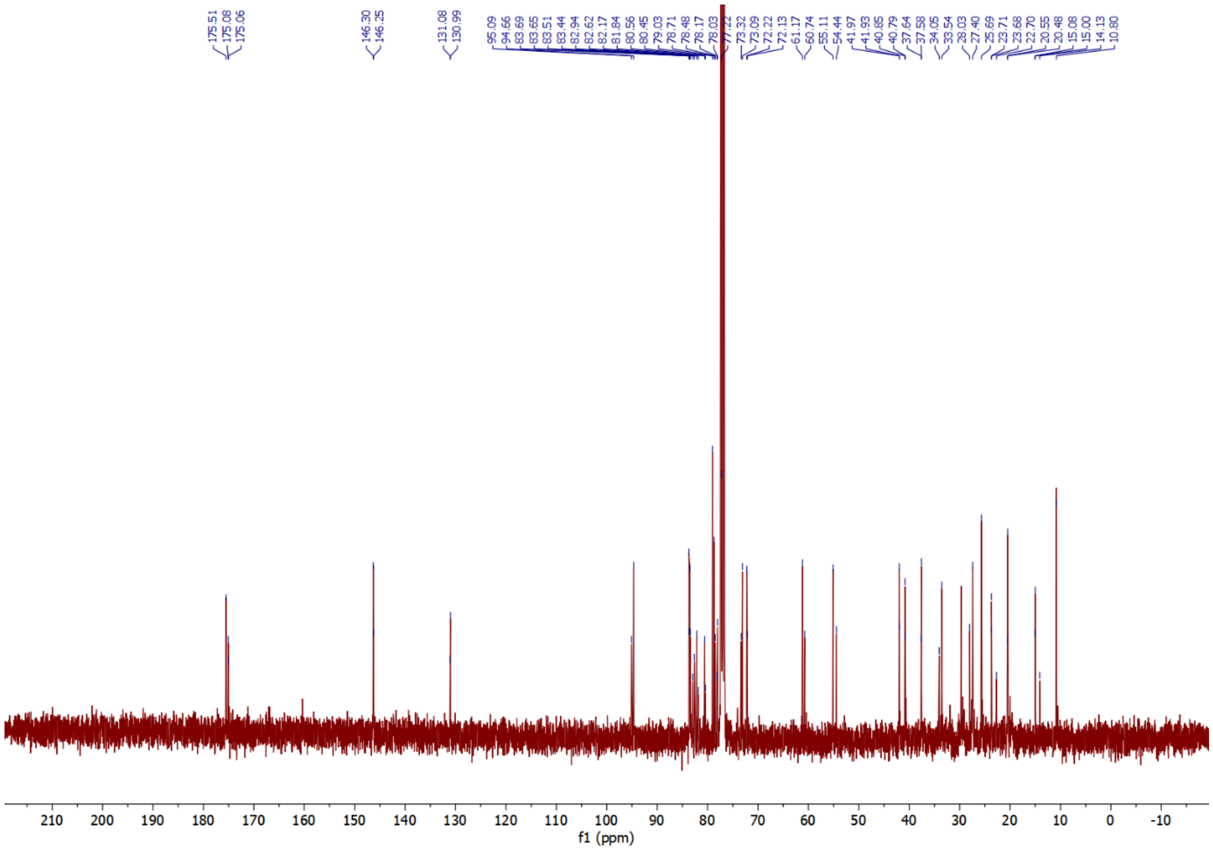

**_Pseudo_-rubriflordilactone B ($CDCl_3$), 2**

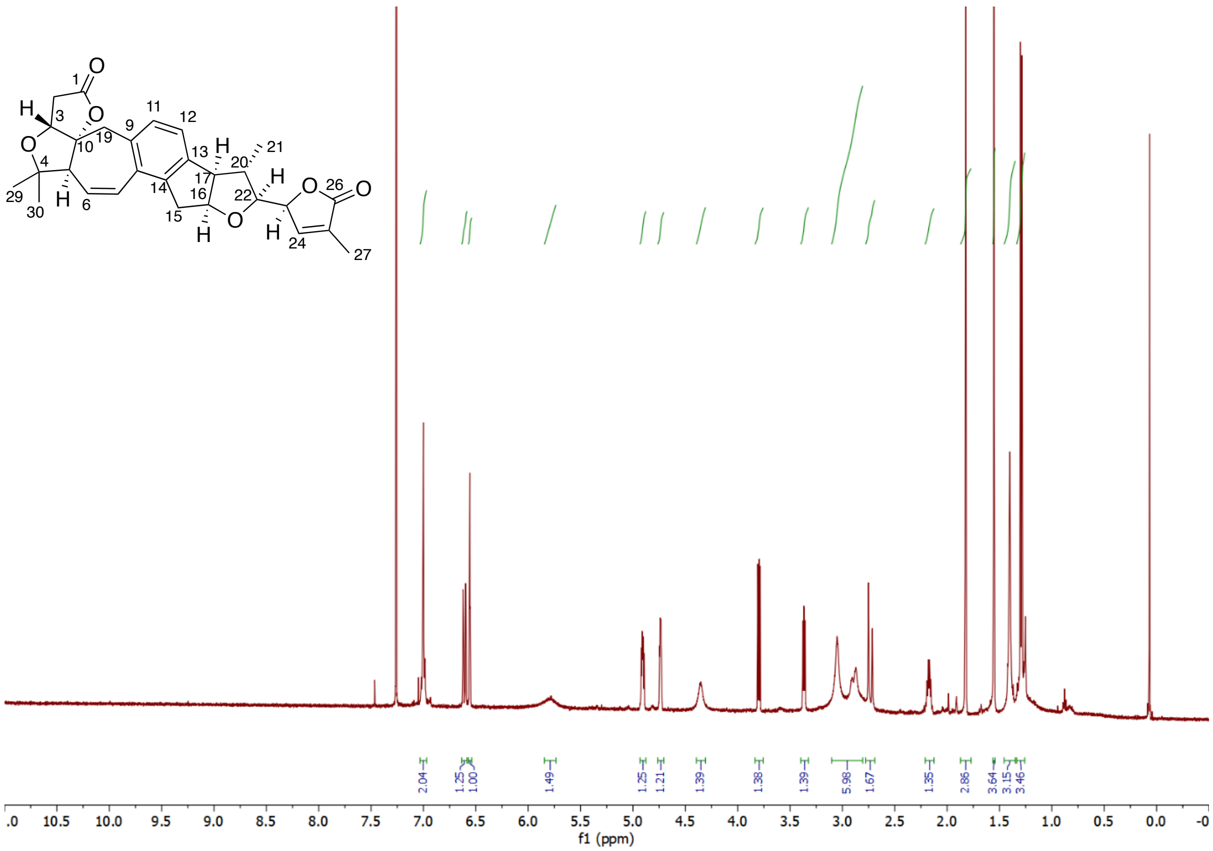


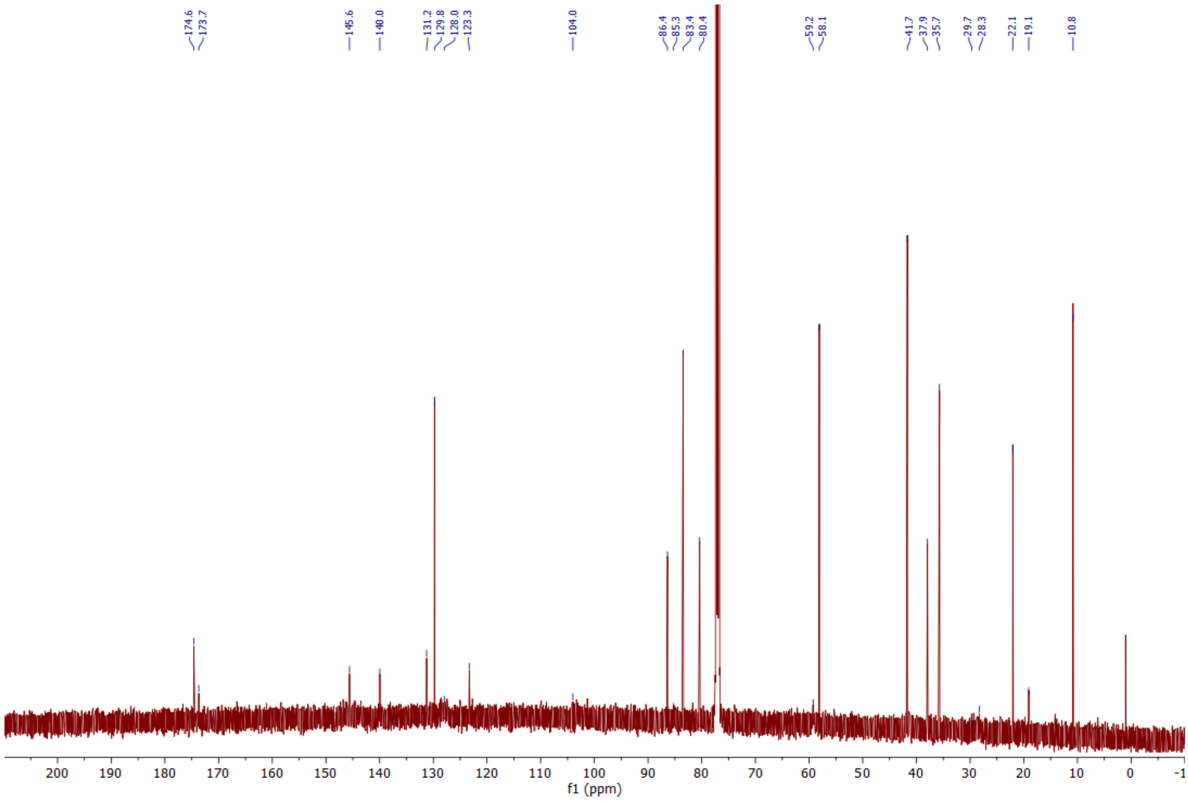

***Pseudo*-rubriflordilactone B (d5-pyridine, 25 °C ), 2**

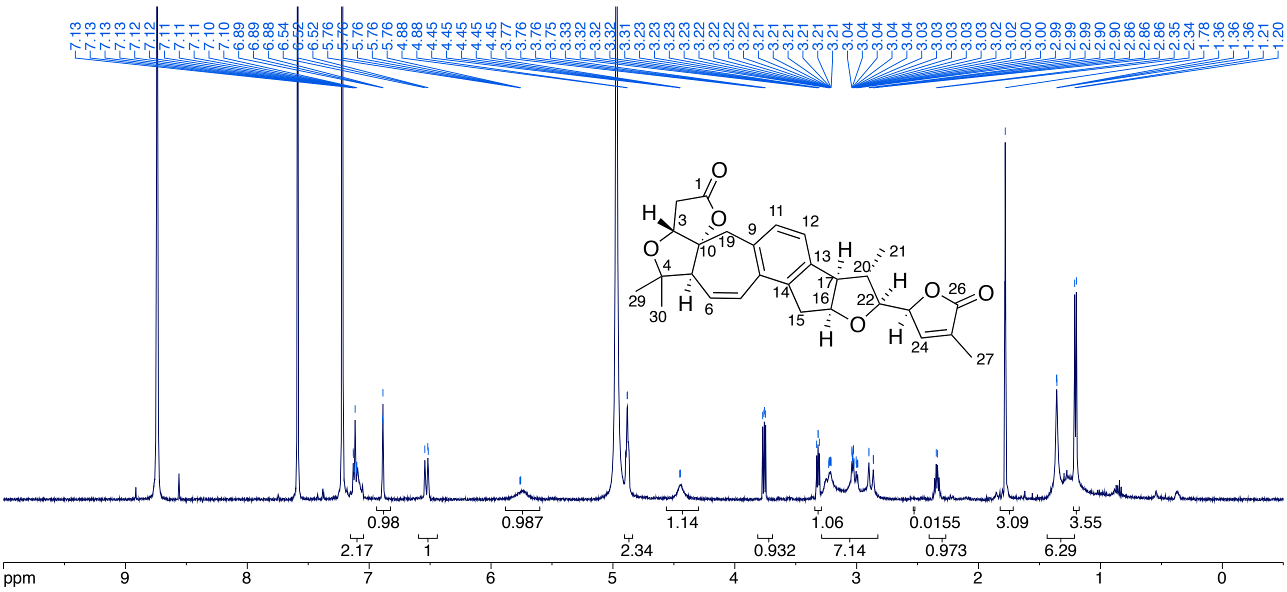


**298 K $^{13}$C NMR** (d5-pyridine)

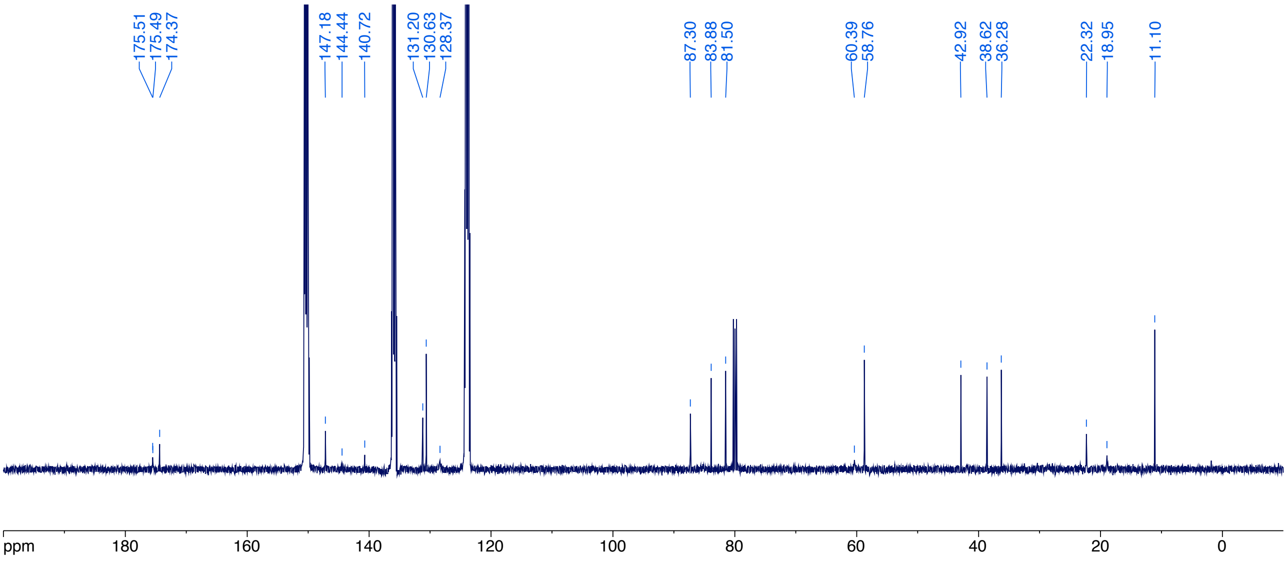


**333 K $^{13}$C NMR** (d5-pyridine)

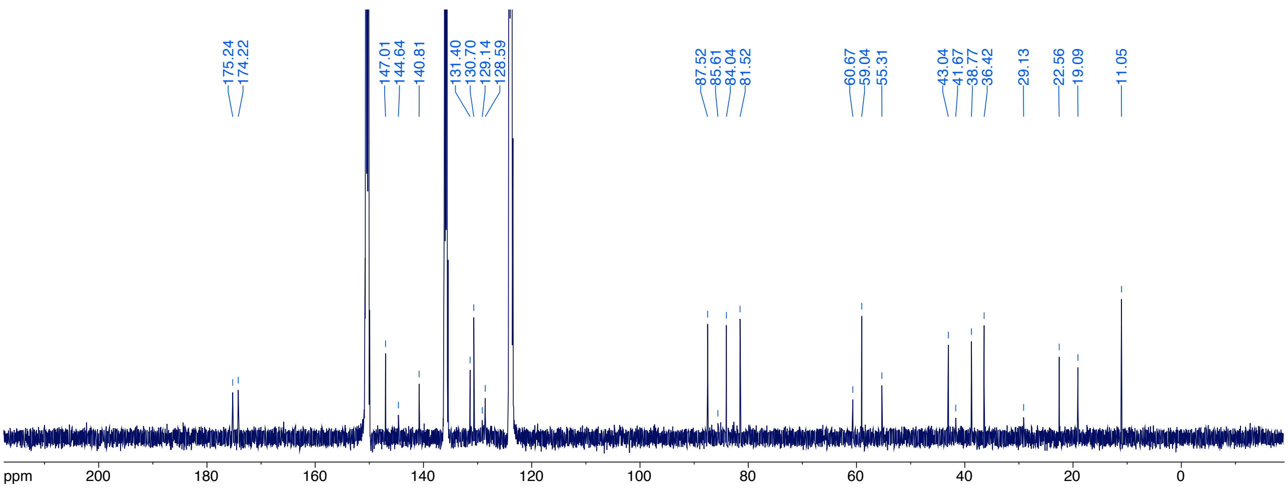

***Pseudo*-rubriflordilactone B (d5-pyridine, 298 K) $^1$H–$^1$H COSY**

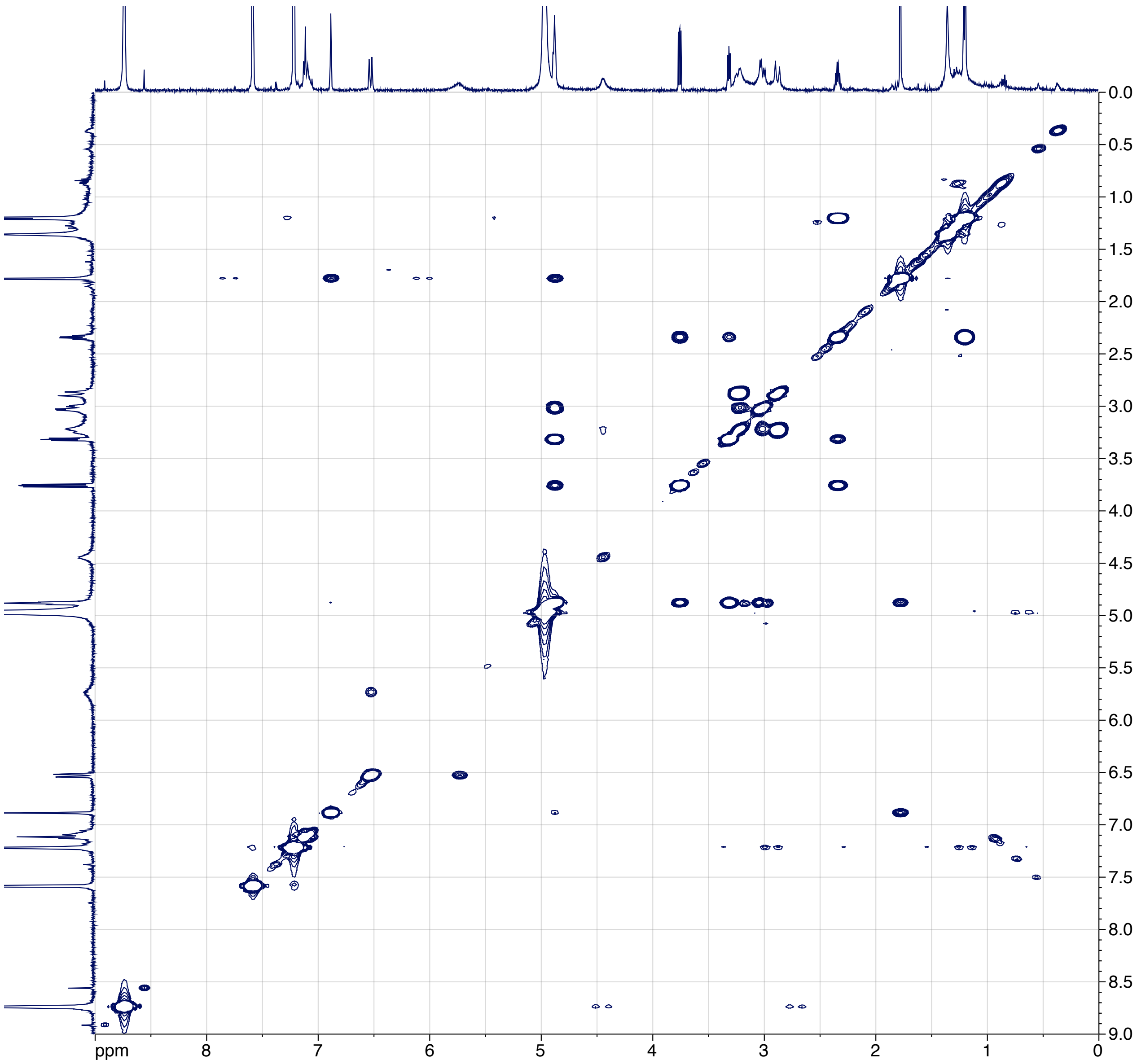

*Pseudo*-rubriflordilactone B (d5-pyridine, 298 K) $^{1}H$–$^{13}C$ HSQC

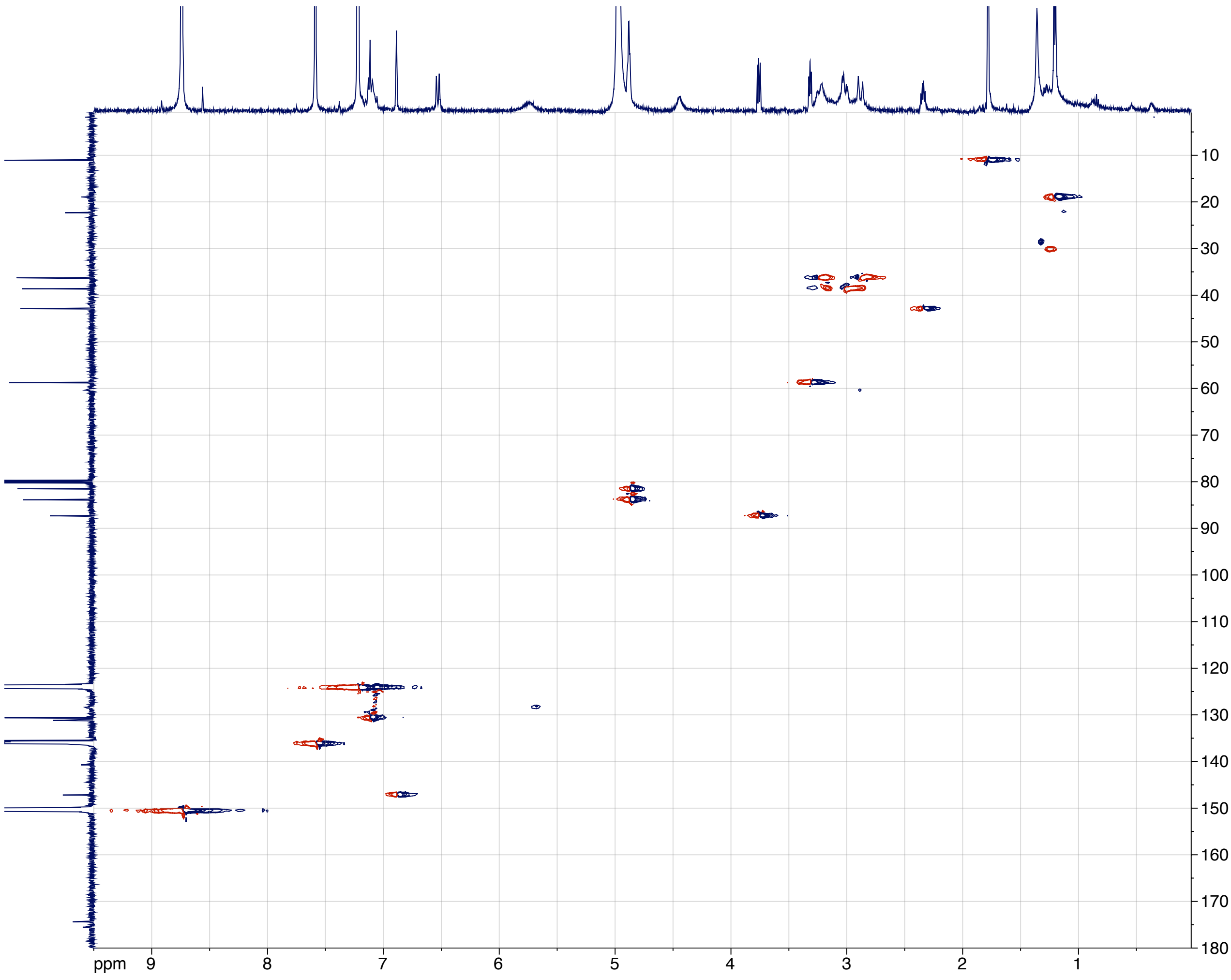

***Pseudo*-rubriflordilactone B (d5-pyridine, 298 K) $^{1}H–^{13}C$ HMBC**

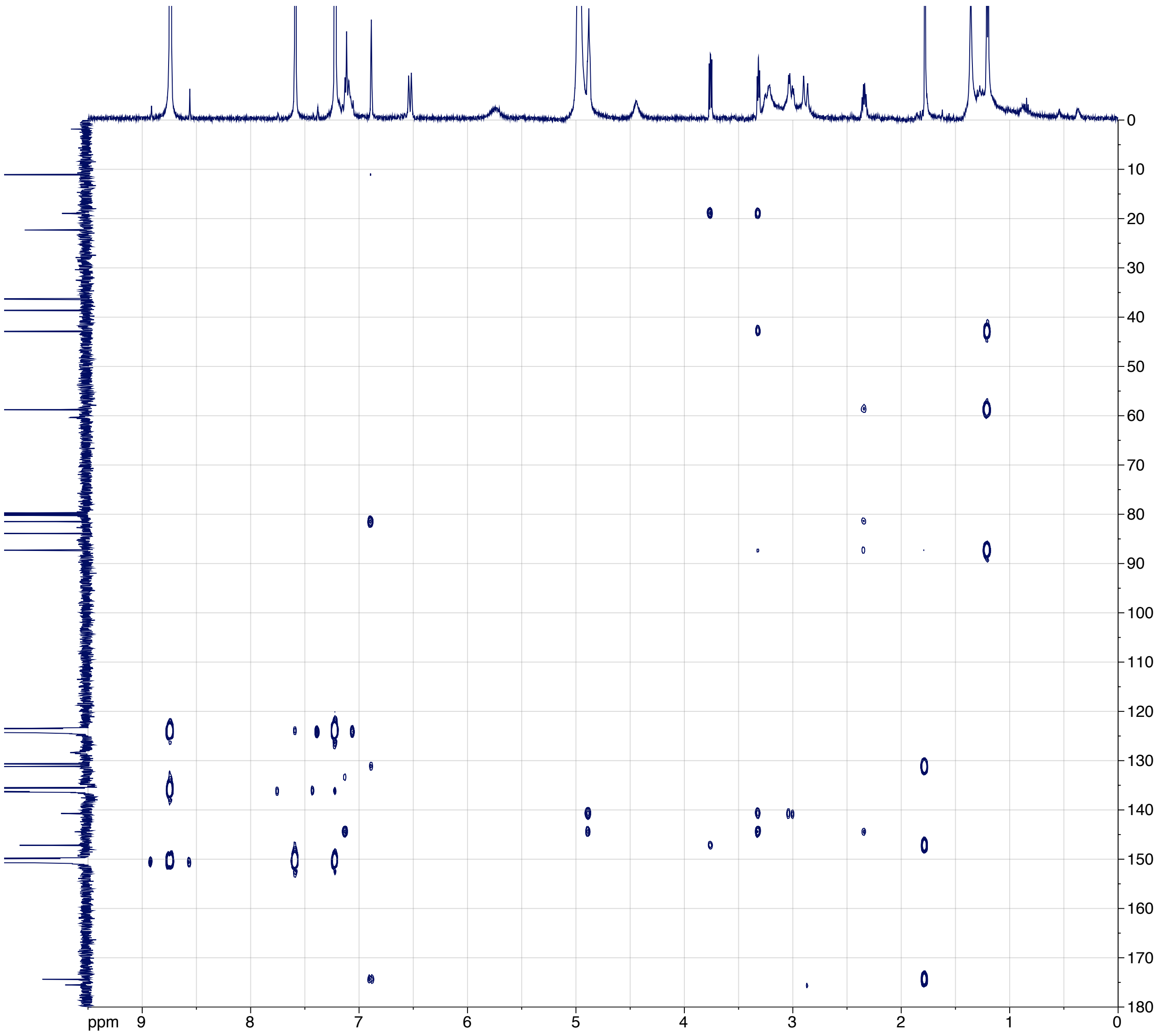

**(3a*R*,6*S*,6a*R*)-6-(2-Hydroxy-5-((2*S*,3*R*,4*S*,5*S*)-4-methyl-5-((*R*)-4-methyl-5-oxo-2,5-dihydrofuran-2-yl)-3-((trimethylsilyl)ethynyl)tetrahydrofuran-2-yl)pent-3-yn-1-yl)-5,5-dimethyl-6a-(prop-2-yn-1-yl)tetrahydrofuro[3,2-b]furan-2(3*H*)-one, S21**

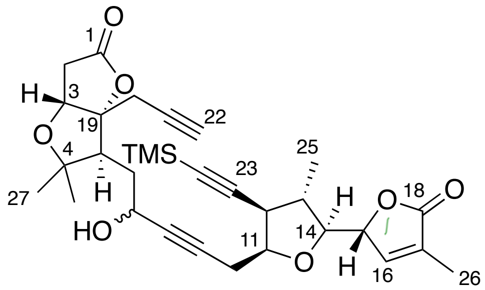


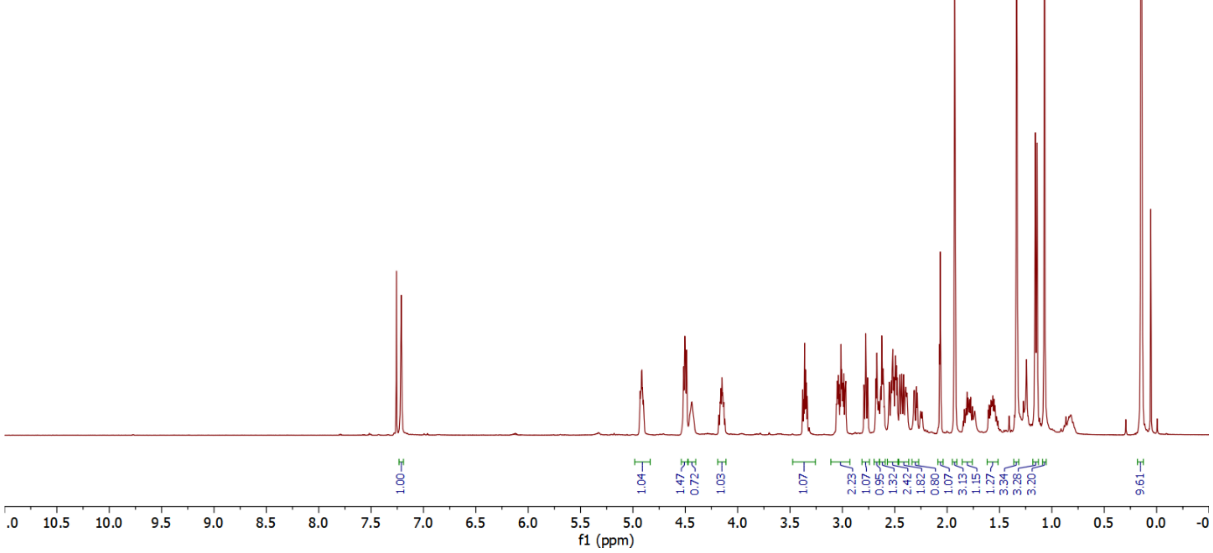


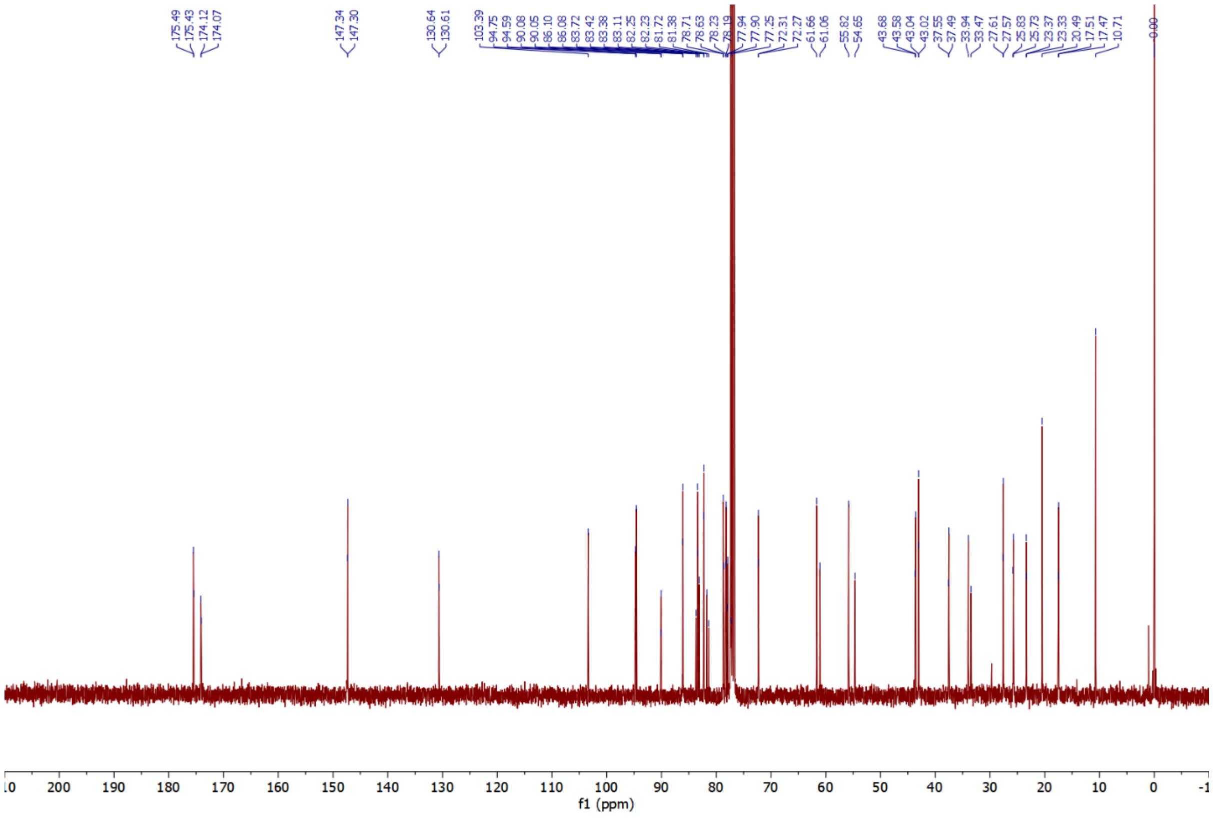

**(3a*R*,6*S*,6a*R*)-6-(5-((2*S*,3*R*,4*S*,5*S*)-3-ethynyl-4-methyl-5-((*S*)-4-methyl-5-oxo-2,5-dihydrofuran-2-yl)tetrahydrofuran-2-yl)-2-hydroxypent-3-yn-1-yl)-5,5-dimethyl-6a-(prop-2-yn-1-yl)tetrahydro-furo[3,2-b]furan-2(3*H*)-one; 37**

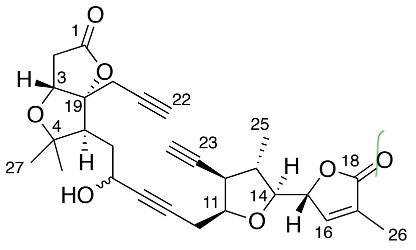

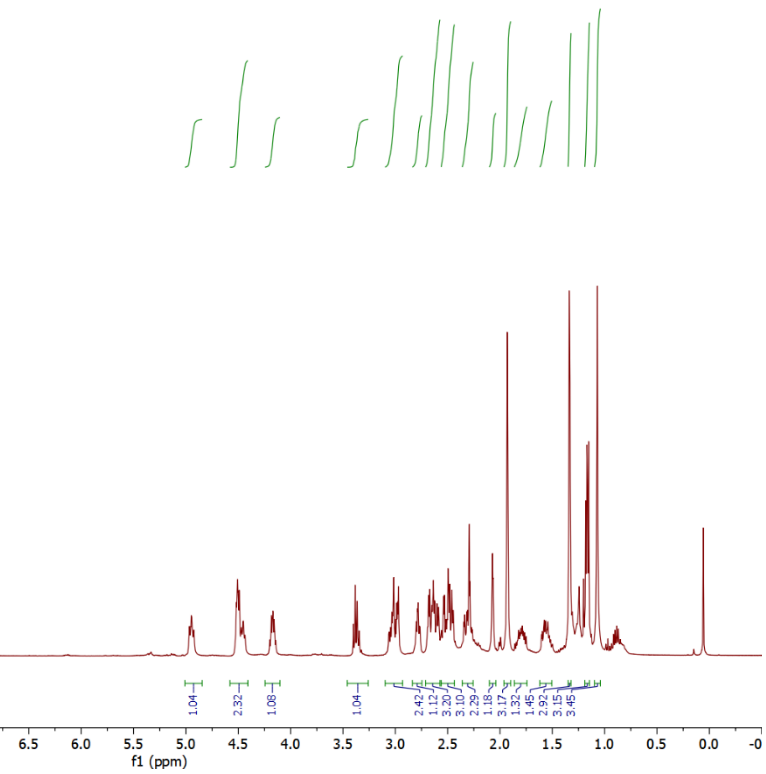

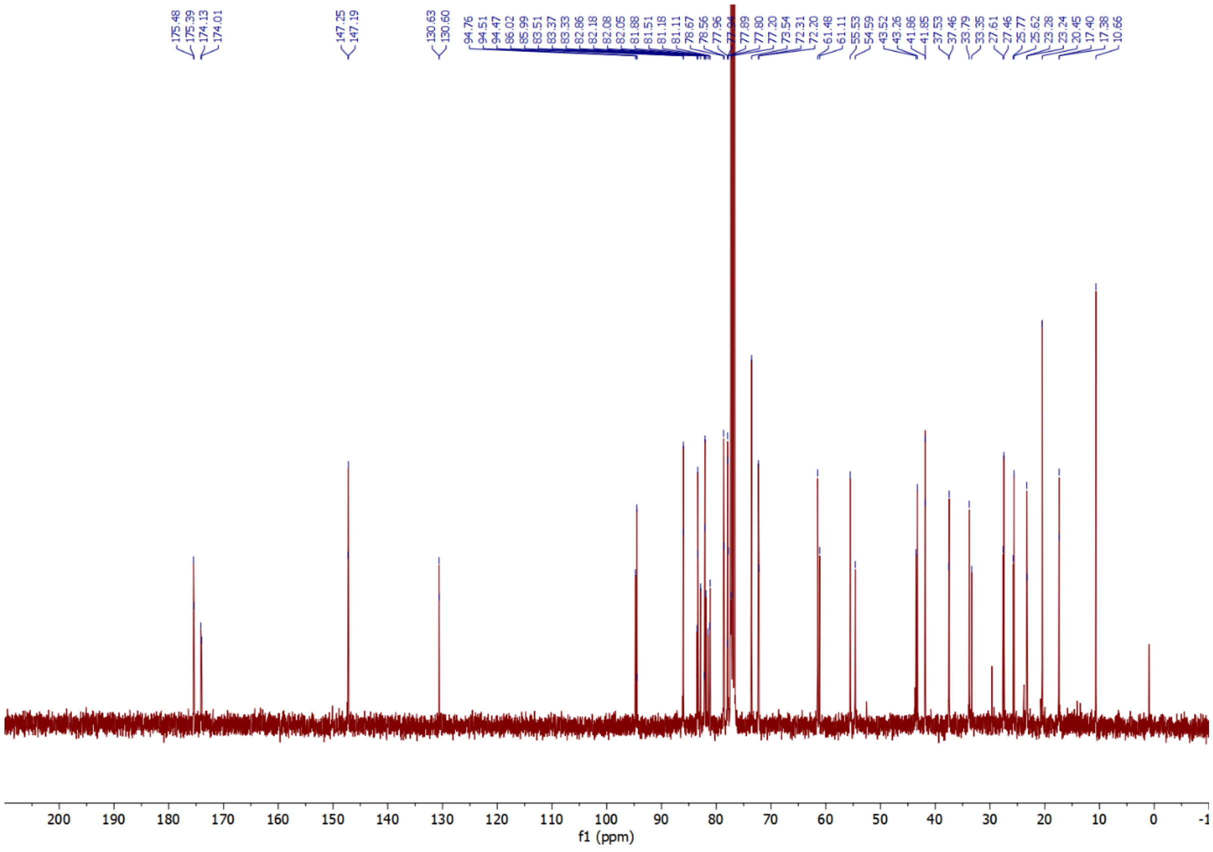

**23-*Epi*-*pseudo*-rubriflordilactone B ($CDCl_3$), *epi*-2**

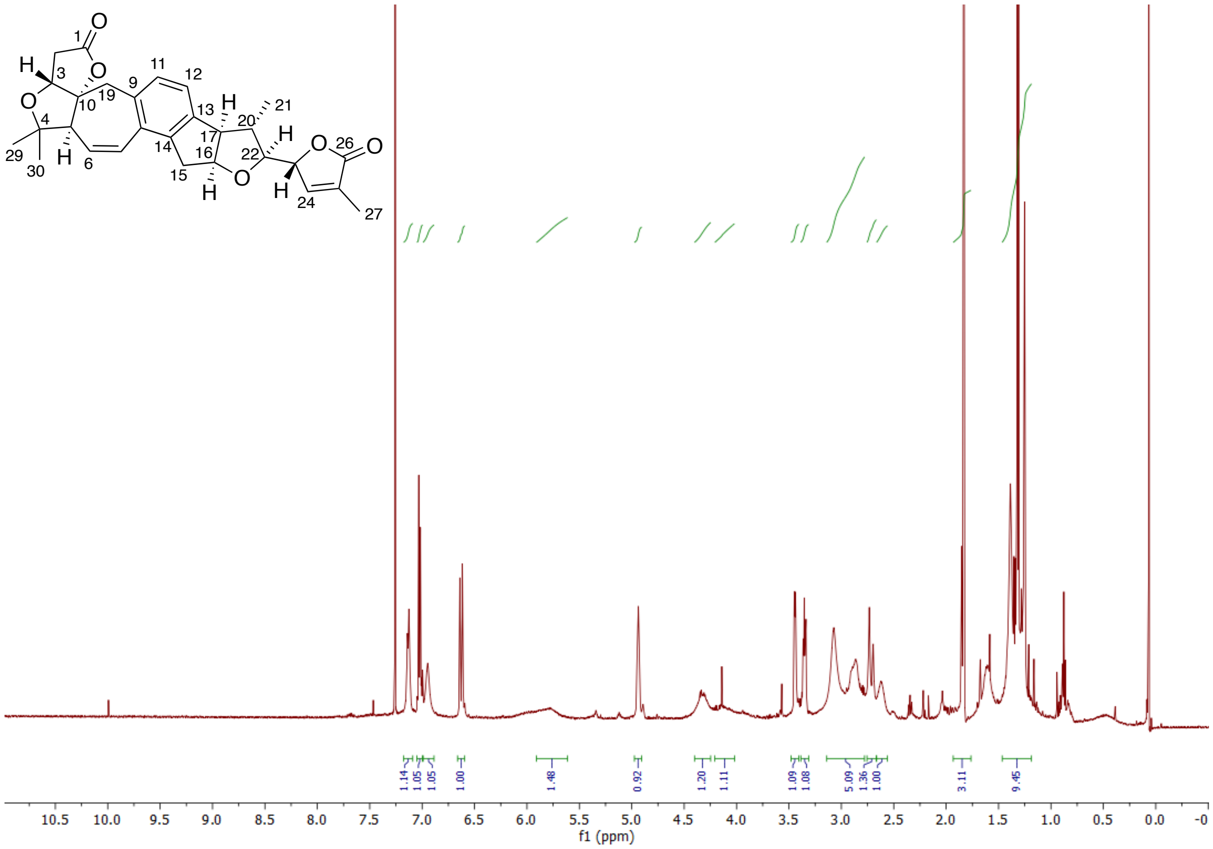


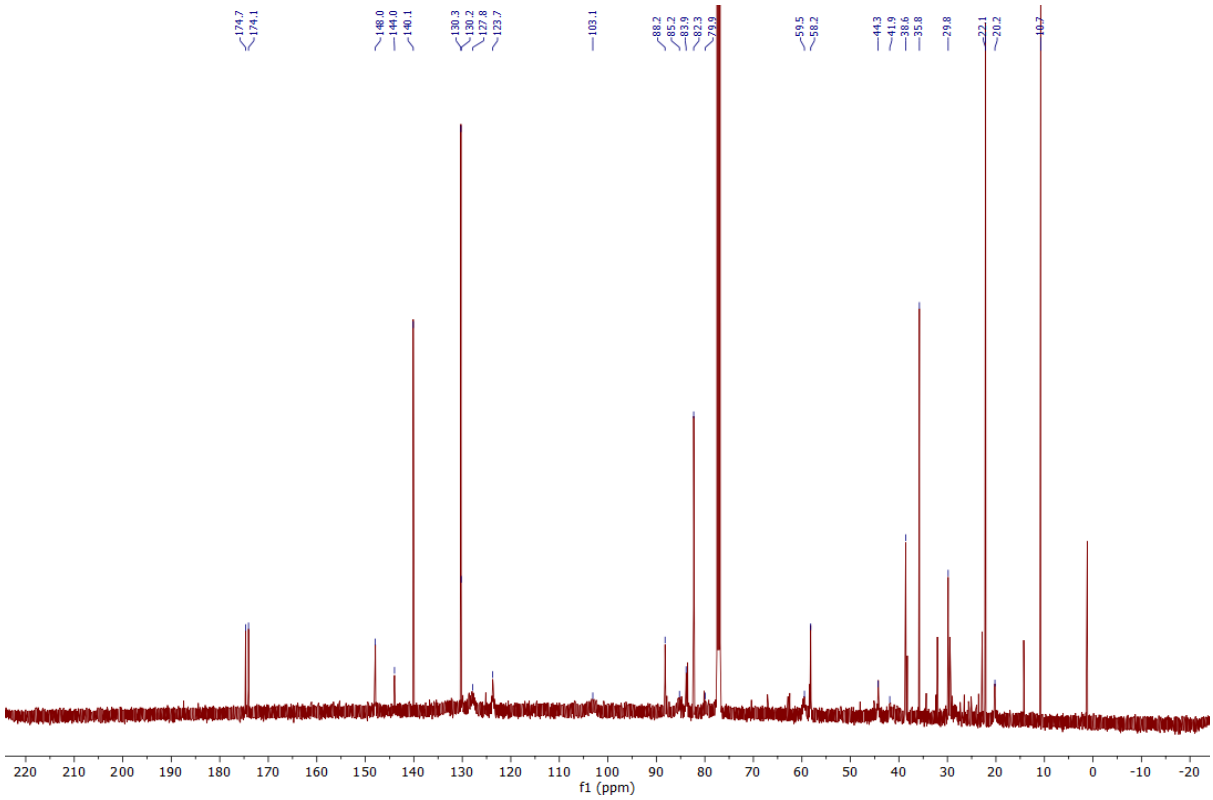

**23-*Epi-pseudo*-rubriflordilactone B (pyridine-d5), *epi*-2**

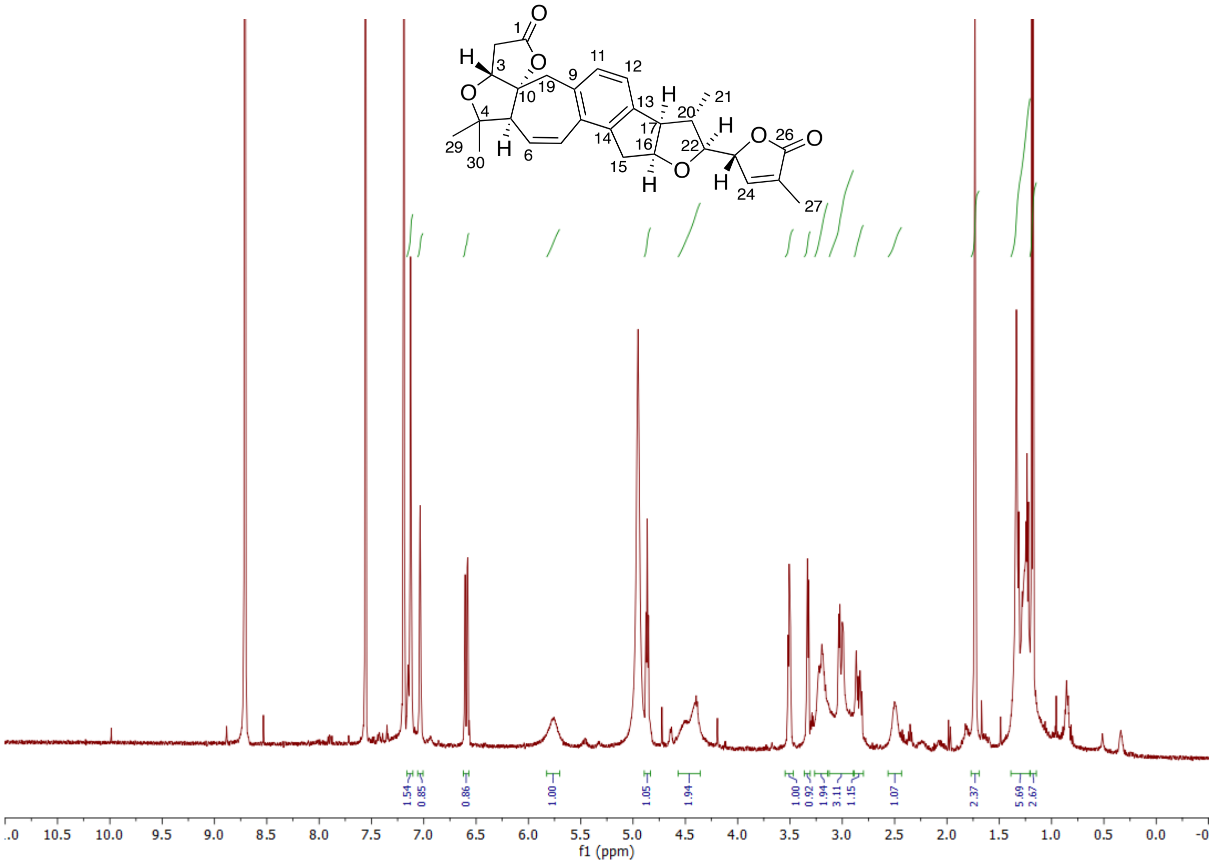


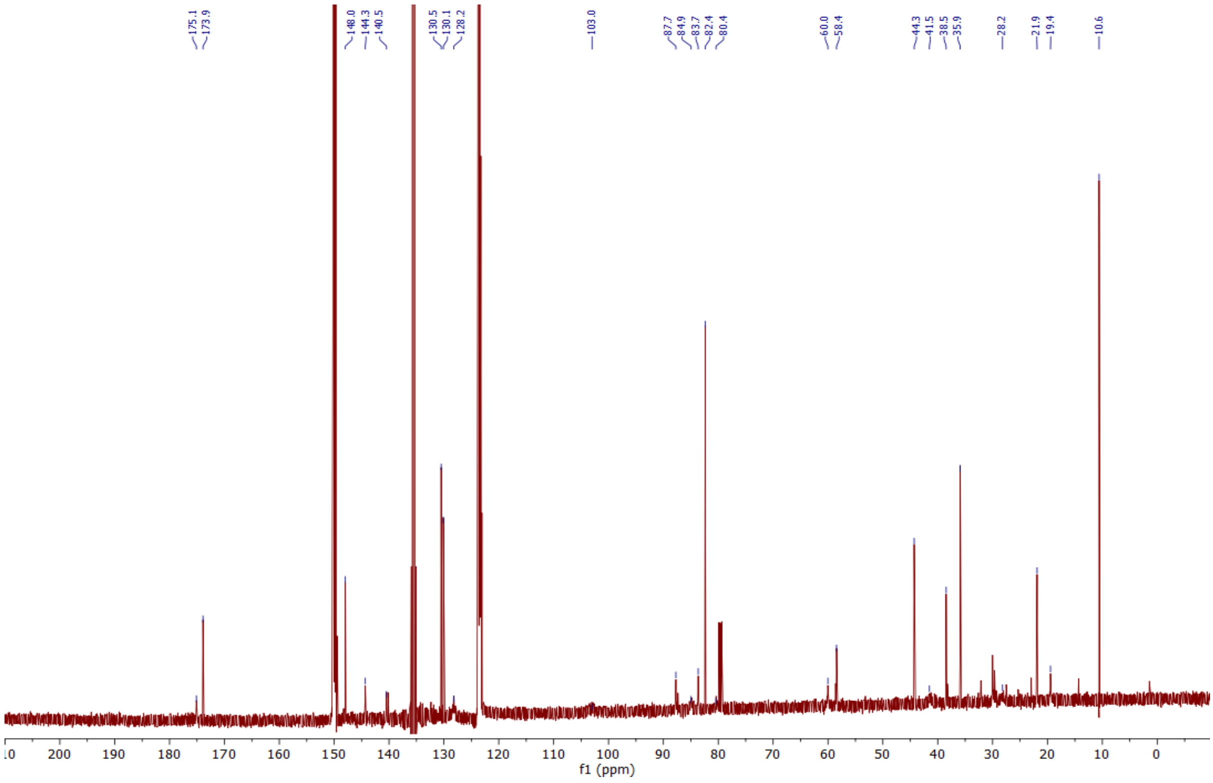

Supplement: Supplementary file 1 — Supplementary [file ANIE-58-18177-s001.pdf]
